# Serum Extracellular Vesicle-Derived microRNAs as Potential Biomarkers for Pleural Mesothelioma in a European Prospective Study

**DOI:** 10.3390/cancers15010125

**Published:** 2022-12-25

**Authors:** Elisabetta Casalone, Giovanni Birolo, Barbara Pardini, Alessandra Allione, Alessia Russo, Chiara Catalano, Manlio Mencoboni, Daniela Ferrante, Corrado Magnani, Marika Sculco, Irma Dianzani, Federica Grosso, Dario Mirabelli, Rosa Angela Filiberti, Ottavio Rena, Carlotta Sacerdote, Miguel Rodriguez-Barranco, Karl Smith-Byrne, Salvatore Panico, Claudia Agnoli, Theron Johnson, Rudolf Kaaks, Rosario Tumino, José María Huerta, Elio Riboli, Alicia K Heath, Camino Trobajo-Sanmartín, Matthias B. Schulze, Calogero Saieva, Pilar Amiano, Antonio Agudo, Elisabete Weiderpass, Paolo Vineis, Giuseppe Matullo

**Affiliations:** 1 Department of Medical Sciences, University of Turin, 10126 Turin, Italy; 2 Italian Institute for Genomic Medicine, IIGM, 10060 Candiolo, Italy; 3 Candiolo Cancer Institute, FPO-IRCCS, 10060 Candiolo, Italy; 4 Medical Oncology, ASL 3 Genovese, Villa Scassi Hospital, 16149 Genoa, Italy; 5 Unit of Medical Statistics, Department of Translational Medicine, University of Eastern Piedmont and Cancer Epidemiology, CPO Piemonte, 28100 Novara, Italy; 6 Department of Health Sciences, University of Eastern Piedmont, 28100 Novara, Italy; 7 Interdepartmental Center for Studies on Asbestos and other Toxic Particulates “G. Scansetti”, University of Turin, 10126 Turin, Italy; 8 Mesothelioma Unit, AO SS. Antonio e Biagio e Cesare Arrigo, 15121 Alessandria, Italy; 9 Unit of Cancer Epidemiology, Città della Salute e della Scienza, University-Hospital and Center for Cancer Prevention (CPO), 10126 Turin, Italy; 10 Clinical Epidemiology, IRCCS AOU San Martino IST, 16100 Genoa, Italy; 11 Unit of Thoracic Surgery, University of Novara, 28100 Novara, Italy; 12 Escuela Andaluza de Salud Pública (EASP), 18012 Granada, Spain; 13 Instituto de Investigación Biosanitaria ibs.GRANADA, 18012 Granada, Spain; 14 Centro de Investigación Biomédica en Red de Epidemiología y Salud Pública (CIBERESP), 28029 Madrid, Spain; 15 Cancer Epidemiology Unit, Nuffield Department of Population Health, University of Oxford, Oxford OX3 7LF, UK; 16 EPIC Centre of Naples, Dipartimento di Medicina Clinica e Chirurgia, Federico II University, 80100 Napoli, Italy; 17 Fondazione IRCCS Istituto Nazionale dei Tumori, 20133 Milano, Italy; 18 Division of Cancer Epidemiology, German Cancer Research Center (DKFZ), 69120 Heidelberg, Germany; 19 German Center for Lung Research (DZL), Translational Lung Research Center (TLRC), 69120 Heidelberg, Germany; 20 Hyblean Association for Epidemiology Research AIRE ONLYS, 97100 Ragusa, Italy; 21 Department of Epidemiology, Murcia Regional Health Council, IMIB-Arrixaca, 30008 Murcia, Spain; 22 Department of Epidemiology and Biostatistics, School of Public Health, Imperial College, London W2 1PG, UK; 23 Navarra Public Health Institute, 31008 Pamplona, Spain; 24 Navarra Institute for Health Research (IdiSNA), 31008 Pamplona, Spain; 25 Department of Molecular Epidemiology, German Institute of Human Nutrition Potsdam-Rehbruecke, 14558 Nuthetal, Germany; 26 Institute of Nutritional Science, University of Potsdam, 14558 Nuthetal, Germany; 27 Cancer Risk Factors and Life-Style Epidemiology Unit, Institute for Cancer Research, Prevention and Clinical Network (ISPRO), 50139 Florence, Italy; 28 Ministry of Health of the Basque Government, Sub Directorate for Public Health and Addictions of Gipuzkoa, 20013 San Sebastian, Spain; 29 Epidemiology of Chronic and Communicable Diseases Group, Biodonostia Health Research Institute, 20014 San Sebastian, Spain; 30 Unit of Nutrition and Cancer, Catalan Institute of Oncology—ICO, 08908 L’Hospitalet de Llobregat, Spain; 31 Nutrition and Cancer Group, Epidemiology, Public Health, Cancer Prevention and Palliative Care Program, Bellvitge Biomedical Research Institute—IDIBELL, 08908 L’Hospitalet de Llobregat, Spain; 32 International Agency for Research on Cancer, World Health Organization, 69372 Lyon, France; 33 MRC Centre for Environment and Health, School of Public Health, Imperial College, London W2 1PG, UK; 34 Medical Genetics Unit, Città della Salute e della Scienza, 10126 Turin, Italy; 35 Department of Medical Sciences, Via Santena 19, 10126 Torino, Italy

**Keywords:** malignant pleural mesothelioma, biomarkers, microRNAs, early changes, next generation sequencing

## Abstract

**Simple Summary:**

Malignant pleural mesothelioma (MPM) is an aggressive and still incurable cancer. There is an urgent need to identify effective and reliable tools for detecting and diagnosing the early onset of MPM. In our study, we investigated the whole miRNAs expression profile from serum extracellular vesicles to identify early changes related to MPM development. miR-11400, miR-148a-3p, and miR-409-3p levels were increased in pre-clinical MPM patients up to five years before their diagnosis. The three-miRNA pattern showed a good discrimination capacity to distinguish pre-clinical MPM from cancer-free controls. The three miRNAs also displayed high diagnostic capabilities for differentiating between MPM patients and controls. This study identified a potential EV miRNA signature in preclinical MPM up to five years before diagnosis and raises the possibility of early intervention.

**Abstract:**

Malignant pleural mesothelioma (MPM) is an aggressive cancer with a dismal prognosis. Early therapeutic interventions could improve patient outcomes. We aimed to identify a pattern of microRNAs (miRNAs) as potential early non-invasive markers of MPM. In a case-control study nested in the European Prospective Investigation into Cancer and Nutrition cohort, we screened the whole miRNome in serum extracellular vesicles (EVs) of preclinical MPM cases. In a subgroup of 20 preclinical samples collected five years prior MPM diagnosis, we observed an upregulation of miR-11400 (fold change (FC) = 2.6, adjusted *p*-value = 0.01), miR-148a-3p (FC = 1.5, *p*-value = 0.001), and miR-409-3p (FC = 1.5, *p*-value = 0.04) relative to matched controls. The 3-miRNA panel showed a good classification capacity with an area under the receiver operating characteristic curve (AUC) of 0.81 (specificity = 0.75, sensitivity = 0.70). The diagnostic ability of the model was also evaluated in an independent retrospective cohort, yielding a higher predictive power (AUC = 0.86). A signature of EV miRNA can be detected up to five years before MPM; moreover, the identified miRNAs could provide functional insights into the molecular changes related to the late carcinogenic process, preceding MPM development.

## 1. Introduction

Malignant pleural mesothelioma (MPM) is the most aggressive asbestos-related disease, characterized by a dismal prognosis with a median survival of 14 months [1].

The hazardous effect of asbestos is globally well recognized and even though it has been banned in several nations, asbestos is still being used in most low- and middle-income countries. Recently, MPM accounted for 30,443 new cancer cases (0.2%) and 25,576 cancer deaths (0.3%) worldwide [2]. A peak incidence and mortality will be expected to occur before 2030, while in resource-limited countries, it will increase in the next decades. For these reasons the role of asbestos in human health still remains a concern.

Despite advances in understanding its pathogenic mechanism, MPM has remained a major clinical challenge. It is often diagnosed at advanced stages and is refractory to current treatments [3,4], but early detection could enable interventions at a potential resectable stage. Previous studies reported that patients treated with a multimodality therapy, when the tumor is limited to parietal or visceral space, have shown a better overall survival [5,6]. In 2020, phase III clinical trials positioned immunotherapy as a promising option for the first- and second-line treatment of MPM and led to a significant improvement of overall survival. However, the 5-year survival rate is still low, with a percentage of 9.6% [7]. 

Multiple approaches have been attempted to identify biomarkers in order to facilitate early non-invasive detection of the disease, with potential application in screening at-risk populations [8]. However, none of the identified markers achived a significant sensitivity and specificity to allow for a translation into clinical settings and the diagnosis is still challenging. Cytological samples obtained by thoracentesis or by fine-needle aspiration biopsy may be inconclusive for a definitive diagnosis [9]. Liquid biomarkers have sparked a lot of interest due to their advantages over a single tissue biopsy. Currently, three blood-based biomarkers have been extensively investigated for MPM for their diagnostic potential, such as soluble mesothelin, fibulin-3, and osteopontin. However, their clinical utility is limited by their poor sensitivity.

A growing number of reports have shown that circulating microRNAs (miRNAs) may have clinical relevance as cancer biomarkers, for their unique expression profiles, depending not only on the type of cancer but also on the disease stage. Most circulating miRNAs are released in the blood stream encapsulated in extracellular vesicles (EVs), lipid bilayer membranes involved in cell-to-cell communication with surrounding and distant cells by shaping their metabolism, and modulating gene expression [10].

EVs can be released from the originating cell either through the outward budding of the plasma membrane or through the inward budding of the endosomal membrane. The most relevant EV subtypes are exosomes (50–150 nm), microvesicles (200 nm–1 µm), and apoptotic bodies, which are all notably involved in physiological processes as well as in pathological development [11].

In addition, miRNAs in EVs are protected from Rnases degradation, making them ideal candidates for liquid biopsies and avoiding the need for invasive procedures to obtain tissue biopsies [12].

Cavalleri et al. identified miR-103a-3p and miR-30e-3p derived from plasma EVs were able to discriminate MPM from asbestos-exposed individuals [9]. However, most of the studies examined circulating cell-free miRNAs [13]. First, among all miR-126-3p was consistently down-regulated both in tissue and serum of MPM patients and could differentiate MPM from NSCLC [14].

Increased circulating expression of miR-625-3p and high levels of miR-34b/c methylation in serum-circulating DNA were associated with MPM diagnosis [15,16].

These results do not allow any conclusion regarding performance of miRNAs for the detection of MPM at early stages, deriving from the analysis of incident cases of MPM and they need to be validated in longitudinal studies.

In the present study, we performed, for the first time, a comprehensive miRNome profile using a Next Generation Sequencing (NGS) approach, of serum circulating EVs in pre-clinical MPM cases, within a case-control study nested in the European Prospective Investigation into Cancer and Nutrition (EPIC) cohort.

## 2. Materials and Methods

### 2.1. Study Cohort

EPIC is a multi-center cohort, involving 23 centers from 10 European countries (Denmark, France, Germany, Greece, Italy, the Netherlands, Norway, Spain, Sweden, and the United Kingdom). The study design has been described previously [17]. 

In the EPIC cohort, 148 participants developed MPM (EPIC-MESO) at the last follow-up (2016), however serum samples were available for 82 pre-clinical participants. Serum was collected at recruitment and, thus, prior to MPM diagnosis, that occurred between 17 days and 18 years after blood collection, with a median time to diagnosis of 8 years. Controls were individuals nested in the EPIC cohort without cancer diagnosis up to and including the last follow-up. One control was randomly selected for each case from the same center and matched for age at blood collection (±1.5 years), sex, and asbestos exposure.

An expert epidemiologist assessed the asbestos exposure as described in Supplementary Methods “Asbestos exposure assesment”.

All participants gave their written informed consent to participate in the EPIC study. Our study complies with the Declaration of Helsinki principles and conforms to ethical requirements.

### 2.2. Isolation of Serum Extracellular Vesicles and RNA Extraction

EVs were isolated from 200 µL of serum using ExoQuick precipitation solution (System Biosciences, Palo Alto, CA, USA) according to manufacturer’s instructions. Briefly, 200 µL of serum was processed with 50.4 µL ExoQuick solution and stored at 4 °C overnight. The EVs pellets were resuspended with 200 µL of nuclease free water and RNA was immediately isolated from the solution.

Total RNA was extracted with miRNeasy serum/plasma kit (Qiagen, Hilden, Germany) using the QIAcube extractor (Qiagen, Germany) according to manufacturer’s instructions. RNA concentration was determined for all samples with Qubit 2.0 Fluorometer with miRNA assay kit (Thermo Fisher Scientific, Waltham, MA, USA). 

### 2.3. Library Preparation and Next Generation Sequencing 

Small non coding RNA libraries were constructed using NEBNext Multiplex Small RNA Library Prep set for Illumina (New England Biolabs, Inc., Ipswich, MA, USA) [18]. The cDNA libraries were purified with the Qiagen PCR Purification kit following the modifications indicated by NEBNext Multiplex Small RNA Library Prep instructions. Six pools of cDNA barcoded samples were prepared and each pool was finally size-selected microRNAs in a 6% PolyAcrylamide Gel (Thermo Fisher Scientific, Waltham, MA, USA). Fragments with an insert of 150 nucleotides were cut out and purified with Qiagen Gel Extraction MiniElute columns (Qiagen, Germany) following the modifications indicated in NEBNext Multiplex Small RNA Library Prep Protocol. A Bioanalyzer 2100 system (Agilent Technologies, Santa Clara, CA, USA) was used to check the size of each cDNA library fragment. Single-end sequencing (75 nucleotides) was performed on NextSeq550 platform (Illumina, San Diego, CA, USA).

### 2.4. Nanoparticle Tracking Analysis and Electron Microscopy

EV concentration was measured in a random pair of samples by Nanosight NS300 (Malvern Instruments Ltd., Malvern, UK) equipped with a 488 nm laser module that utilizes Brownian motion and refraction index. Samples were diluted 1:200 in physiologic solution filtered with 100 nm pore size. For each sample, three videos of 30 s at camera level 15 and threshold 5 were captured. All samples were characterized with Nanoparticle Tracking analysis (NTA).

The presence and integrity of EVs were assessed by Transmission electron microscopy (TEM) as described in Verta et al. [19].

### 2.5. miRNAs Sequencing Data Analysis

The data from miRNA sequencing (miRNA-seq) were computationally processed and analyzed as previously described [18]. Differential expression analysis was performed with the DESeq2 Bioconductor’s package (version 1.22.2). 

We adjusted the analysis for age, gender, and batch effect as these parameters can affect miRNA expression [20,21]. Since the samples were collected in six different countries, we also adjusted the analysis for the country of recruitment, as this could introduce a potent confounding factor into the data. The miRNAs’ profiles could also be affected by asbestos exposure, therefore we also included this variable in the adjustment.

For each tested model, samples with missing covariates were dropped and only miRNAs with a median expression of at least ten reads were included.

MiRNAs obtained from the NGS analysis were considered significantly differentially expressed between pre-diagnostic cases and matched controls if their *p*-value, after adjustment for multiple testing by false discovery rate (FDR), was below the 0.05 threshold.

Logistic regression was used to create a predictive model combining the differentially expressed miRNAs into a single score which allows to detect preclinical cases. The model coefficients were re-estimated on the EPIC data using only the expression of selected miRNAs, without matched variables. Its predictive ability was investigated by the ROC (receiver operating characteristic) curve, and the area under the curve (AUC). Specificity and sensitivity were also computed for a standard cut-off value of 0.5.

### 2.6. Technical Validation by RT-qPCR Analysis

We validated the differentially expressed miRNAs identified in pre-clinical cases recruited up to five years before the diagnosis (20 cases and 20 controls), by quantitative reverse transcription polymerase chain reaction (RT-qPCR). To select the candidate miRNA, we used FDR ≤ 0.05 and |log2FoldChange(FC)| ≥ 0.6 as miRNAs selection criteria; however, due to the small sample study, we also included in the validation analysis de-regulated miRNAs with nominal *p*-value < 0.05, |log2 FC| ≥ 0.6 and read counts greater than 100. The full sequence of miRNA assayed are reported in Supplementary Table S1. Laboratory methods and statistical analysis for technical confirmation of the miRNA-seq results are detailed in Supplementary Materials “RT-qPCR analysis”.

### 2.7. Functional Enrichment Analysis of miRNAs Target Genes

The validated target genes of miR-409-3p and miR-148a-3p were retrieved with the MultiMiR Bioconductor’s package, from Mirecords, Mirtarbase, and Tarbase databases [22]. No available experimental evidence has been reported for miR-11400 targets, therefore potential target genes were predicted by the miRWALK2.0 tool [23]. Only those genes with a binding probability of at least 95% were considered as targets and taken into account for further analysis.

The Kyoto Encyclopedia of Genes and Genomes (KEGG) was used as reference for the pathway enrichment analyses and potential functional roles associated with the genes targeted by miR-11400, miR-409-3p, and miR-148a-3p.

The statistical significance of the enriched pathways was determined using ShinyGO v0.741 [24].

### 2.8. Investigation of Selected miRNAs in a Retrospective Cohort

Since a suitable prospective replication cohort could not be found, we investigated by a NGS approach the whole miRNome in 30 asbestos-exposed patients with a diagnosis of MPM and 20 asbestos-exposed cancer-free controls. Asbestos-exposed cases and controls with a well-characterized occupational and environmental asbestos exposure were recruited from two areas of northern Italy. MPM patients were diagnosed between 2018 and 2019 at the Azienda Ospedaliero-Universitaria Maggiore della Carità (Novara) as previously described in [25]. 

The control group belongs to the study described in Filiberti et al. [26]. The 20 retrieved controls were cancer-free at the follow-up in 2017.

The study protocols were approved by the ethics committee of the participating center and written informed consent was obtained from all individuals before enrolment.

The predictive model, previously fitted on the prospective EPIC samples, was tested for discriminating the MPM cases from the controls, using the same performance metrics previously described.

## 3. Results

We investigated the miRNA expression profile at the whole genome level in serum-derived EVs of 82 individuals who developed MPM in 20 years of follow-up and 82 matched cancer-free controls. 

Based on the occupational history data of each participant, it was possible to perform an assessment of asbestos exposure for 58 cases, and the matched controls had the same asbestos exposure probability (Materials and Methods).

Most of the 164 paired samples were males (72%) and came from the United Kingdom (36.6%). The mean age at recruitment was 57.8 years. The characteristics of the study population are summarized in Table 1. 

The presence of EVs after Exoquick precipitation, were confirmed by TEM and by NanoSight analyses. TEM analysis depicted round-shaped EVs (Supplementary Figure S1) and quantitative size analysis using NTA showed that EVs have a range of 120/150 nm (Supplementary Figure S2). 

Sequencing generated per sample an average of nine million reads, ranging from 2.2–31.6 × 10^6^ reads (Supplementary Table S2). On average, 5.7% of the reads obtained from miRNA-seq aligned on miRNA sequences (miRBase v22). Overall, 211 miRNAs were detected in 164 samples.

After read processing and filtering, four samples (two cases and two controls) were removed from the analysis as outliers.

From the analysis of miRNA-seq data derived from 80 prospectively collected MPM cases and 80 controls adjusted for sequencing run, country of recruitment, sex, and age, we did not identify any significant difference in miRNA expression.

Pre-clinical cases were further divided into two groups according to the time between blood collection and MPM diagnosis (less or more than five years before diagnosis). We did not observe any significant differentially expressed miRNAs between the two groups (Supplementary Table S3). 

**Figure 1:**
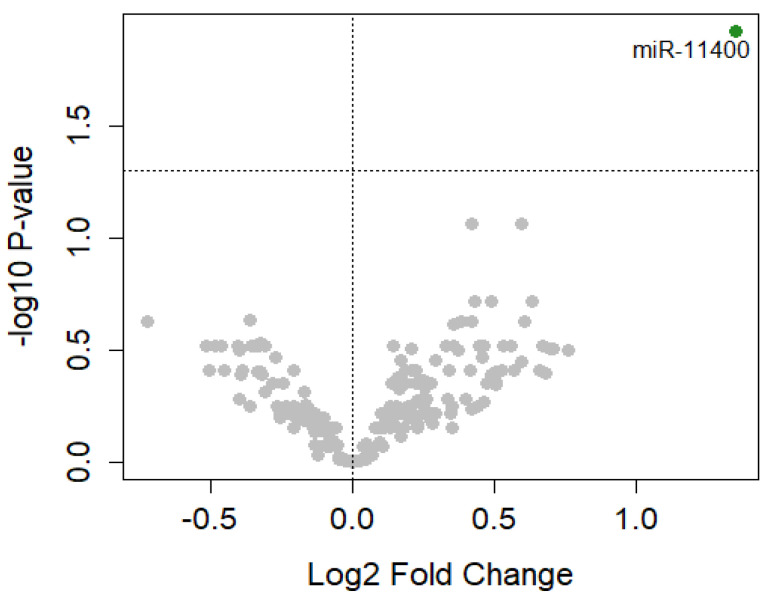
Volcano plot of differentially expressed miRNAs. Each dot represents a miRNA. The green dot represents miR-11400 significantly differentially expressed at FDR ≤ 0.05. The gray dots represent miRNAs that were not significantly differentially expressed at FDR.

**Figure 2:**
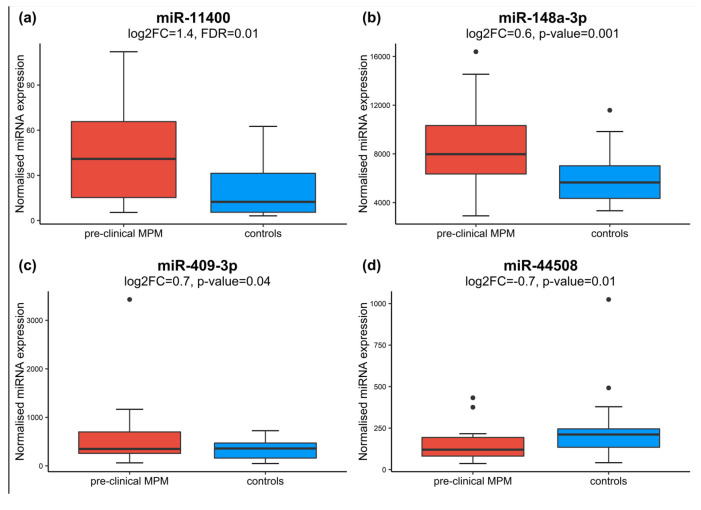
Boxplots show expression levels of miR-11400 (**a**), miR-148a-3p (**b**), miR-409-3p (**c**), and miR-4508 (**d**), in pre-clinical MPM cases collected up to five years before diagnosis and matched controls. Above each boxplot, the *p*-values yielded by DESeq2 after adjustment for multiple testing (FDR) or nominal *p*-value.

In order to observe miRNA dysregulated in relation to the cancer transformation process, we investigated the differentially expressed miRNAs in preclinical cases near to the time of diagnosis (Table 1, EPIC-MESO 5 years). In our dataset, 24 cases developed MPM within five years after the enrolment. Of them, 20 pre-clinical cases and their relative matched cancer-free controls had an assessment of asbestos exposure. We used this subgroup to perform differential expression analysis adjusted for batch effect, age, gender, country, and asbestos exposure. We identified miR-11400 (FDR = 0.01; log_2_FC = 1.4; 95% CI 0.69–2.0) significantly upregulated in cases compared to matched controls (Figure 1, Figure 2a, Supplementary Table S4). 

As described in Material and Methods, we also included for further analysis miR-148a-3p (log_2_FC = 0.6; *p*-value = 0.001; 95% CI 0.2, 0.82) (Figure 2b), miR-409-3p (log2FC = 0.7; *p*-value = 0.04; 95% CI 0.02, 1.3) (Figure 2c), and miR-4508 (log2FC = −0.7; *p*-value = 0.01; 95% CI −1.29, −0.16) (Figure 2d), which were significantly differentially expressed at nominal *p*-value.

In order to evaluate the expression levels of the three-miRNAs signature in MPM and normal tissue, we explored the database of Differentially Expressed MiRNAs in human Cancers (dbDEMC) [27,28]. We compared 25 MPM primary tumors with six normal pleural samples, identifying a significant upregulation in tumor tissue for miR-148a (log_2_FC = 0.4. *p*-value = 9.98 × 10^−3^) and miR-409-3p (log_2_FC = 1.8, *p*-value = 2.11 × 10^−3^).

### 3.1. Validation of miRNA-seq Data by RT-qPCR

The three-miRNA signature (miR-11400, miR-148a-3p, and miR-409-3p) was validated by RT-qPCR in the whole dataset of preclinical cases near to diagnosis and their matched controls (EPIC MESO 5 years). Although miR-4508 was eligible for validation, it was not further investigated due to the failure of TaqMan probe design. The relative expression levels of miR-11400, miR-148a-3p, and miR-409-3p obtained from RT-qPCR agreed with miRNA-seq data based on the expression trends among preclinical cases and controls, however, the differences were not statistically significant (Supplementary Figure S3). 

### 3.2. miRNAs Target Genes and KEGG Enrichment Analysis

Functional enrichment analysis was performed to explore if the identified miRNAs were involved in relevant molecular networks related to the pathogenesis of MPM. MultiMiR Bioconductor’s package was used to retrieve miRNAs validated target genes. We identified 111 experimentally validated targets for miR-148a-3p and miR-409-3p, in particular, 110 were for miR-148a-3p and only one was for miR-409-3p (Supplementary Table S5), while no experimental evidence was available for miR-11400 target genes. 

**Figure 3:**
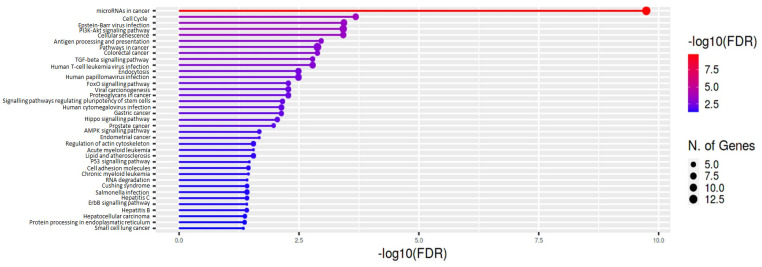
Enrichment analysis of KEGG pathways relative to validated target genes of miR-409-3p and miR-148a-3p. Bar plots show the significantly enriched functional categories ordered by p-value.

The enrichment analysis, carried out considering the validated target genes, revealed a total of 37 significantly enriched terms in KEGG (Figure 3 and Supplementary Table S6). The most significantly altered pathways in KEGG, included “TGF-beta signaling”, “PI3K-Akt signaling”, and “cell cycle pathways”, which have been defined to play essential roles in the development of MPM [29,30].

**Figure 4:**
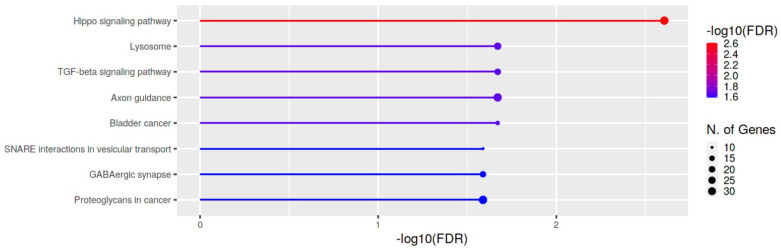
KEGG enrichment analysis of KEGG pathways relative to predicted target genes of miR-11400. Bar plots depict the significantly enriched terms ordered by p-value.

We used miRWalk 2.0 to predict target mRNAs of miR-11400, and we identified 2147 unique target genes (Supplementary Table S7). We also performed an enrichment analysis for these predicted genes and after FDR correction the potential target genes of miR-11400 resulted mainly enriched in lysosome, Hippo signaling pathway and TGF-β signaling pathway (Figure 4 and Supplementary Table S8).

### 3.3. Prediction Model and Validation in a Retrospective Study 

**Figure 5:**
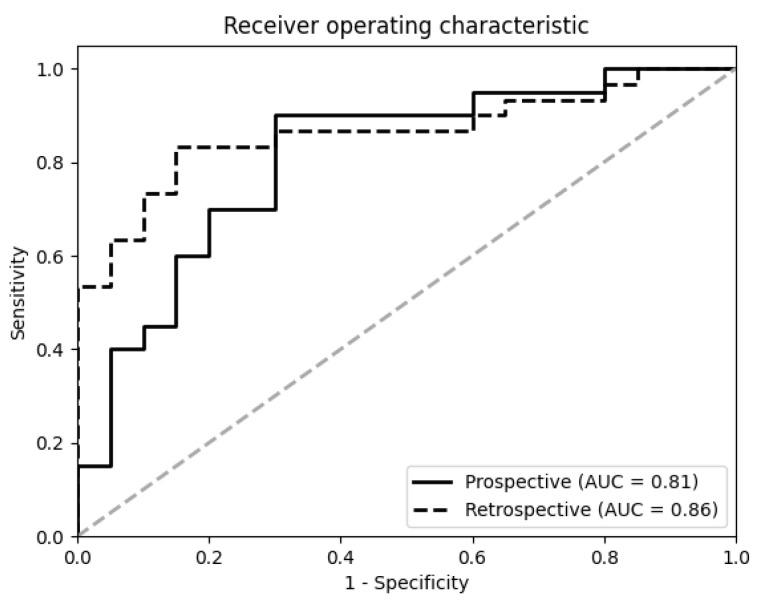
Receiver operating characteristics (ROC) curves of the three miRNAs signature (miR-11400, miR-148a-3p, and miR-409-3p) for the discrimination between preclinical MPM and matched controls (solid line), as well as for discriminating between MPM at diagnosis and healthy controls (dashed line). The area under the curve (AUC) shows the percentage of correct discrimination associating sensitivity (Y) and 1-specificity (X).

To explore the predictive power of the miRNA signature, a multivariate logistic regression model was fitted to distinguish cases diagnosed within five years after baseline and matched cancer-free controls, using the three miRNAs discovered in the previous analyses as features. The model had an AUC of 0.81 (Figure 5), a sensitivity of 75% and a specificity of 70%. The fitted model was validated in an independent retrospective cohort of 30 MPM diagnosed cases and 20 controls (Supplementary Table S9), yielding an AUC of 0.86 with a sensitivity and specificity of 53% and 95%, respectively. Table 2 showed the diagnostic statistics of the three selected miRNAs for MPM prediction.

## 4. Discussion

In the present work, we evaluated if EV-derived miRNAs could serve as reliable tumor biomarkers for early detection of MPM and to gain insight into the molecular mechanisms linked to the late carcinogenic process. By using a NGS approach, we profiled miRNAs expression enclosed in serum EVs of preclinical MPM individuals and cancer-free controls.

We observed a significant upregulation of miR-11400, miR-148a-3p, and miR-409-3p in serum EVs of 20 pre-diagnostic MPM samples collected up to five years before the clinical manifestation of the disease, compared with cancer-free individuals. In the validation phase, miR-11400 and miR-409-3p were found upregulated, while a slight increase of miR-148a-3p expression was observed in preclinical cases.

So far, numerous cell-free miRNAs have been identified as non-invasive biomarkers for MPM, but only miR-126, miR-132-3p, and miR-103a-3p were tested in plasma of preclinical MPM samples, showing that this miRNA pattern was not feasible to detect MPM at early stages [31,32,33,34,35]. Indeed, we did not find any difference in expression levels for the three described miRNAs as reported in Supplementary Table S4. 

In EPIC preclinical MPM cases, a different three-miRNAs panel identified by us could predict MPM onset within five years, yielding an AUC of 0.81 with 75% sensitivity and 70% specificity. In the absence of a prospective validation cohort, we could examine the diagnostic performance of the model in a retrospective study, which confirmed the accuracy and reliability of the three-miRNA signature in distinguishing MPM cases from cancer-free controls even at the time of diagnosis (AUC = 0.86). Although the specificity was even higher than the prospective cohort at 95%, sensitivity dropped to 53% in the retrospective validation study, meaning almost half of the diagnosed cases tested as negative. This suggests that the present miRNA signature may weaken in a number of subjects over time after diagnosis and warrants further investigation. However, it is unlikely to affect undiagnosed cases at earlier stages of MPM and the specificity/sensitivity balance can be adjusted to fine tune the score threshold.

The predictive role of miR-148a-3p has been described in previous cancer studies [36,37,38]. Increasing expression levels of miR-148a-3p have been described as a biomarker for early diagnosis of laryngeal squamous cell carcinoma, and high circulating levels of this miRNA were associated with poor overall and disease-free survival of hepatocellular carcinoma patients after liver transplantation [36,37].

Josson et al. demonstrated that high expression of miR-409 delivered by EVs from prostate stromal fibroblasts promoted tumorigenesis through repression of tumor suppressor genes [39].

Limited data are available in the literature for miR-11400 [40]. The predicted target genes are highly enriched for key regulators of the lysosome, Hippo signaling, and TGF-β pathways. 

The long-term toxicity of asbestos fibers is due to their bio-persistence in cells and their accumulation in lysosomes, causing autophagy as well as lysosomal dysfunctions that result in oxidative stress and inflammation [41]. As it has been demonstrated for nanoparticles, asbestos fibers could also directly affect lysosomal stability by inducing lysosomal oxidative stress, alkalization, osmotic swelling, resulting in lysosomal membrane permeabilization and the subsequent activation of NLRP3 inflammasome [41]. Thus, lysosomal dysfunction provides a mechanism for asbestos-mediated inflammation.

Significant enrichment for Hippo and TGF-β signaling has also been reported for miR-148a-3p-validated target genes. Previous studies have demonstrated a functional interaction between TGF-β and Hippo signaling pathways in MPM cell growth and proliferation [29]. In particular, the inactivation of Hippo signaling components can play a remarkable role in the development and progression of MPM [29,30]. The activated LATS kinase, in association with the tumor-suppressor MOB, predicted target of miR-11400, phosphorylates and inhibits the transcription coactivators TAZ and/or YAP [42]. Therefore, dysregulation of LATS1/2 has been shown to result in constitutive activation of the YAP1/TAZ transcriptional coactivators, conferring malignant phenotypes to mesothelial cells. 

Amongst predicted miR-11400-target genes, the tumor suppressor RASSF1 and the G1-phase cell-cycle regulator CDKN2B are both frequently lost in MPM tissue and implicated in the mechanisms of carcinogenesis [43,44]. Although experimental evidence is needed to validate the functional relationship between miR-11400 and its target genes, these findings suggest its role as a mediator of inflammation and malignant transformation. 

Conversely, further evidence is available for miR-148a-3p. According to pathway enrichment analysis, validated miR-148a-3p target genes were enriched in several MPM-related pathways. We obtained a significant enrichment for FOXO signaling pathway, given by downstream genes mediating the cell cycle and apoptosis. Suzuki and colleagues demonstrated that the up-regulation of miR-182 and miR-183 suppresses the expression of FOXO1, which in turn promotes cell proliferation by acting on its downstream targets CDKN1A, and CDKN1B [45]. Likewise, miR-148a-3p targeted and suppressed cell cycle and apoptotic regulators (BCL2L11, CDKN1A, CDKN1B, S1PR1) likely leading to uncontrolled proliferation [46].

It is well demonstrated that EVs represent an ideal tool used by cells of primary neoplastic lesions to shape local and distant microenvironments promoting optimal conditions for tumor growth and proliferation [47]. 

The levels of serum EV miRNA might reflect the profiles in cancer tissue. By using dbDEMC, we reported that miR-148a-3p and miR-409-3p were upregulated in MPM tissues in agreement with the results in serum EVs, supporting their potential mechanisms in tumor-related biological processes and potentially reflecting the MPM progression [27,28]. The expression changes in miR-11400 levels were not detected since the authors used a microarray not including miR-11400 among the probes.

Our study presents several limitations. First, the small number of prospectively collected cases limits the robustness of our results. In addition, EPIC did not include multiple sampling during follow-up; as a result, a serum sample preceding diagnosis no more than five years (i.e., presumably during the MPM pre-clinical phase) was available for only part of all EPIC cases, entailing further limitation of the study dimension. MPM is a rare malignancy and obtaining pre-clinical samples is challenging. Settings where members of high-risk groups might be invited to participate to studies of pre-clinical MPM markers are medical surveillance programs for former asbestos workers; no such program, however, is active in Italy. A validation prospective cohort was not, thus, available to us; we tested, therefore, the identified mi-RNAs panel in a case-control setting, which had the disadvantage of evaluating sensitivity and specificity at the time of MPM diagnosis, rather than during its pre-clinical phase.

More importantly, it might be questionable whether research into pre-clinical MPM markers is justified on clinical as well as public health grounds, given the current limitations of the options that might be offered to suspect pre-clinical MPM cases: the diagnostic work-up is invasive and associated with non-negligible risks, while treatment offers only limited hopes of disease eradication. However, both diagnostic approaches and treatment might evolve if a shift might be obtained towards a larger proportion of MPM cases in potentially resectable disease stages. It also has to be mentioned that the same markers may prove useful as ancillary diagnostic tools for a non-negligible proportion of cases, such as those for whom thoracoscopy is contraindicated or where microscopic samples turn out difficult to be interpreted.

Besides the potential role of miRNAs, it would be worth identifying and validating other biomarkers of different molecular sources (e.g. proteins, DNA methylation), that in combination with small non coding RNAs might improve the diagnostic performance of MPM detection. As in other tumors, it is well known that the use of circulating biomarkers in pre-diagnostic samples may be a meaningful approach for detection at early stages [48,49]. So far, the combination of CALRETININ and MESOTHELIN has been showed to have the better diagnosis performance in plasma of preclinical asbestos exposed individuals, up to about one year before diagnosis [50]. However, several confounding factors may influence their plasma concentration and the study of additional markers that can decrease the false positive tests is warranted [51,52].

## 5. Conclusions

Most of the MPM group receives a diagnosis in their advanced tumor stage, which makes therapeutic approaches unfeasible, entailing a worse survival. A timely diagnosis may significantly improve the efficacy of the current therapeutic options and the overall survival. 

This study identified a potential EV miRNA signature in preclinical MPM up to five years before diagnosis and raises the possibility of early intervention. If further replicated in other prospective cohorts, EV miRNAs in combination with other biomarkers may significantly impact on the management of this deadly tumor in the future.

## Figures and Tables

**Table 1:** Descriptive table of the samples included in the EPIC-MESO study at recruitment. ALL EPIC-MESO indicates the sequenced pre-MPM cases and matched controls. EPIC-MESO 5 years includes the subgroup of preclinical MPM collected within five years prior to MPM diagnosis and their matched controls.

	ALL EPIC-MESO (*n* = 164)		EPIC-MESO 5 Years (*n* = 48)	
	CA = 82	CO = 82	CA = 24	CO = 24
**GENDER**				
M (%)	59 (72)	59 (72)	20 (83.3)	20(83.3)
F (%)	23 (28)	23 (28)	4 (16.7)	4(16.7)
**AGE** (mean ± sd)	57.7 ± 8.1	57.8±8.1	60.4 ± 7.6	60.4 ± 7.6
**ASBESTOS EXPOSURE**				
unexposed (%)	18 (22)	18 (22)	3 (12.5)	3 (12.5)
exposed (%)	40 (48.7)	40 (48.7)	17 (70.8)	17 (70.8)
NA	24 (29.3)	24 (29.3)	4(16.7)	4(16.7)
**COUNTRY**				
Germany (%)	12 (14.6)	12 (14.6)	9 (37.5)	9 (37.5)
Spain (%)	10 (12.2)	10 (12.2)	1 (4.2)	1 (4.2)
France (%)	1 (1.2)	1 (1.2)	1 (4.2)	1 (4.2)
Italy (%)	21 (25.6)	21 (25.6)	4 (16.6)	4 (16.6)
The Netherlands (%)	8 (9.8)	8 (9.8)	1 (4.2)	1 (4.2)
United Kingdom (%)	30 (36.6)	30 (36.6)	8 (33.3)	8 (33.3)

CA: preclinical cases; CO: controls; M: male; F: female.

**Table 2:** Diagnostic accuracy of miR-11400, miR-148-3p, and miR-409.

	Prospective	Retrospective
AUC	81%	86%
Sensitivity	75%	53%
Specificity	70%	95%
PPV	71%	94%
NPV	74 %	57%
Accuracy	73%	70%

AUC, area under curve; PPV, positive predictive value; NPV, negative predictive value.

## Data Availability

The data presented in this study are available on request from the corresponding author upon request and approval by the Data Access Committee.

## Supplementary Materials

The following supporting information can be downloaded at: https://www.mdpi.com/article/10.3390/cancers15010125/s1. Supplementary Methods: Asbestos exposure assessment, RT-qPCR analysis. Figure S1: Representative micrograph of transmission electron microscopy of EVs isolated from 200 µL of serum; Figure S2: Representative nanoparticle tracking analysis of serum EVs showing the EVs size distribution; Figure S3: Results of RT-qPCR validation of miR-11400 (a), miR-148a-3p (b), and miR-409-3p (c) in 19 pre-clinical MPM samples and 19 matched controls. Table S1: Primary sequence of miRNAs analyzed by RT-qPCR; Table S2: MiRNA NGS pre-processing statistics; Table S3: Differentially expressed miRNAs between 80 pre-diagnostic MPM cases and 80 matched controls from NGS; Table S4: Differentially expressed miRNAs between 20 pre-diagnostic MPM cases and 20 matched controls from NGS; Table S5: Validated miRNA-target interaction results from MultiMiR Bioconductor’s package. Table S6: KEGG pathway enrichment analysis for validated target genes of miR-409-3p and miR-148a; Table S7: miRWalk 2.0 consensus target predictions for miR-11400; Table S8: KEGG pathway enrichment analysis for miR-11400 predicted target genes; Table S9: Characteristics of the retrospective study used in the validation analysis.
