# Supplementary material for: Serum Extracellular Vesicle-Derived microRNAs as Potential Biomarkers for Pleural Mesothelioma in a European Prospective Study
Source: Cancers (Basel). 2022 Dec 25;15(1):125. doi: 10.3390/cancers15010125 (PMC9817828; doi:10.3390/cancers15010125)
Supplement: Supplementary file 1 [file cancers-15-00125-s001.zip › cancers-1979232-supplementary.pdf]

Supplementary Methods.

Asbestos exposure assessment

Occupational information was available from the baseline EPIC questionnaire. It included the occupation at enrolment and data on ever working up to the time of enrolment in 52 at-risk occupations. No information was available on duration of employment and time of first employment. A semi-quantitative job-exposure matrix (JEM) was developed by expert epidemiologists as previously described [1], assigning to each occupation an "exposure probability" and an "exposure intensity" based on occupational history data (occupational categories of the questionnaire 1-52). The "exposure probability" and "exposure intensity" were coded as: 0 = no probability/intensity; 1 = low probability/intensity; 2 = intermediate probability/intensity; 3 = high probability/intensity.

The two values (probability and intensity) are then used to build up, for each occupation, an "Exposure Index" given by "Probability of exposure" * "intensity of exposure". The Exposure Index (EI) may thus assume the following values: 0, 1, 2, 3, 4, 6, 9. Each occupation has its own EI, which may be assigned to each individual as his/her own Exposure Index. If a participant has several occupations, a "cumulative exposure index" is computed. The cumulative exposure index is the sum of all the EIs (one for each occupation) of that individual. For participants with just one occupation the EI and the Cumulative Exposure Index coincide.

Derivative Exposure Indexes: the Exposure Index assigned to each individual according to his/her occupation (or the Cumulative Exposure Index in the case of multiple occupations) is used to build up derivative exposure indexes: a "Binary Exposure Index", coded as: 0 = no exposure (if the Exposure Index, or cum. index in the case of multiple jobs, is < 3); 1 = exposed (if the Exposure Index, or cumulative index in the case of multiple jobs, is ≥ 3). The table below shows the 52 occupational categories and exposure matrix.

	EPIC list of occupations	Probability of exposure	Intensity of Exposure	Exposure index	Binary exposure index
1	1.1 Livestock breeding	0	0	0	0
2	1.2 Agriculture	0	0	0	0
3	2 mines or quarries	0	0	0	0
4	3 Foundry	1	2	2	0
5	- Steel	1	2	2	0
6	- Special alloys	1	2	2	0
7	4 Galvanic	0	0	0	0
8	5 Chemical Industry	1	2	2	0
9	- Refinery	1	2	2	0
10	- Dyes Production	1	2	2	0
11	- Chemical Laboratory	0	0	0	0
12	6 Rubber Industry	1	1	1	0
13	7 Textile Industry	1	1	1	0
14	- Of tissues Dyeing	2	2	4	1
15	- Weaving	1	2	2	0
16	8 Processing and tanning	0	0	0	0
17	9 Production of shoes and leather	0	0	0	0
18	10 Woodworking	0	0	0	0
19	- Production of furniture	0	0	0	0
20	11 Metalworking	1	1	1	0
21	- Turning, drilling, milling etc..	0	0	0	0
22	- Welding	2	2	4	1
23	- Painting	0	0	0	0
24	12 Boatyard	2	3	6	1
25	13 Electrical and Electronics Industry	1	1	1	0
26	14 Glass Industry	1	2	2	0
27	15 Typography	0	0	0	0
28	16 Construction	1	2	2	0
29	- Roof Waterproofing	2	2	4	1
30	- Asphalt	0	0	0	0
31	- Demolition	2	2	4	1
32	17 Transport	1	1	1	0
33	- Truck driver	1	2	2	0
34	- Driver	0	0	0	0
35	- Taxi driver	0	0	0	0
36	18 Nuclear Industry	1	2	2	0
37	19 Production of paper or cellulose	1	2	2	0
38	20 Production of asbestos and asbestos-cement	3	3	9	1
39	21 Worker with asbestos insulation	3	3	9	1
40	22 Production of cement	0	0	0	0
41	23 Production of ceramics	0	0	0	0
42	24 Butcher	0	0	0	0
43	25 Painter, painter	1	1	1	0
44	26 Welder	2	2	4	1
45	27 Hairdresser	0	0	0	0
46	28 Gas Station	0	0	0	0
47	29 Auto Mechanic	1	1	1	0
48	30 Bartender	0	0	0	0
49	31 Warden restaurant	0	0	0	0
50	32 Medical and Health Services	0	0	0	0
51	33 Electrician	2	2	4	1
52	34 Other	0	0	0	0

RT-qPCR analysis

For technical confirmation of the miRNA-seq results, the RT-qPCR was performed according to TaqMan® MicroRNA Assays with custom RT pools and custom preamplification pools protocol (Thermo Fisher Scientific, USA). Briefly, four µl of total RNA was used for RT using TaqMan miRNA Reverse Transcription Kit (Life Technologies, USA) with custom RT primer pool. Two samples (a case and its matched control) were excluded due to insufficient RNA amount, therefore RT-qPCR analysis was performed on 38 samples.

The RT product (3.5 µl) was preamplified with TaqMan PreAmp Master Mix and a custom PreAmp primer pool. PreAmp product was diluted with 0.1 xTE (10mM TrisHCl, 1mM disodium EDTA pH8.0) to final volume of 200µl, then 0.5 µl of pre-amplified cDNA was used for PCR reaction with TaqMan Fast Advanced master mix. Real-time PCR was performed on CFX96 Real-Time PCR machine (BioRad, USA) according to the manufacturer's protocol. Relative gene expression was calculated using the $2^{-\Delta\Delta Ct}$ method and normalised to the endogenous control miR-92b-3p [2]. The differences in the expression of the analysed miRNAs between pre-clinical cases and cancer-free controls were assessed by the Wilcoxon test.

Supplementary Figures

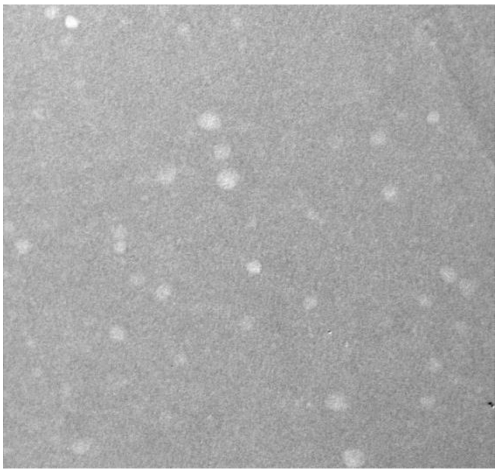

Supplementary Figure S1. Representative micrograph of transmission electron microscopy of EVs isolated from 200 ul of serum.

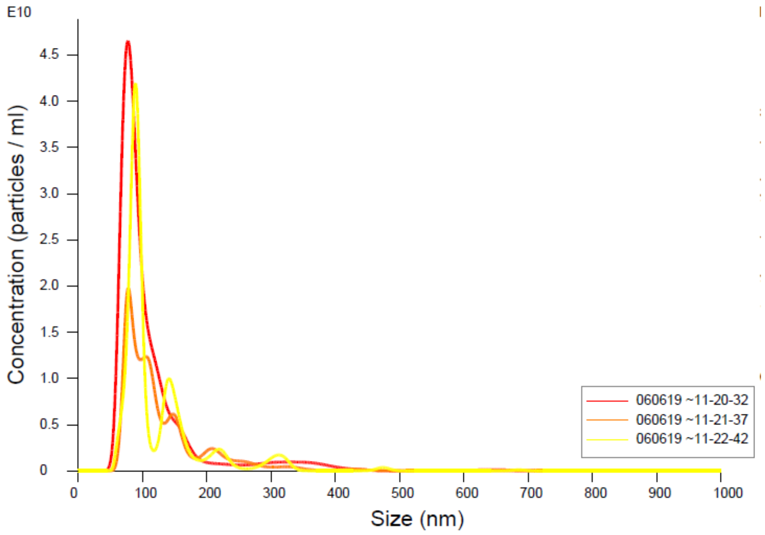

Supplementary Figure S2. Representative nanoparticle tracking analysis of serum EVs showing the EVs size distribution.

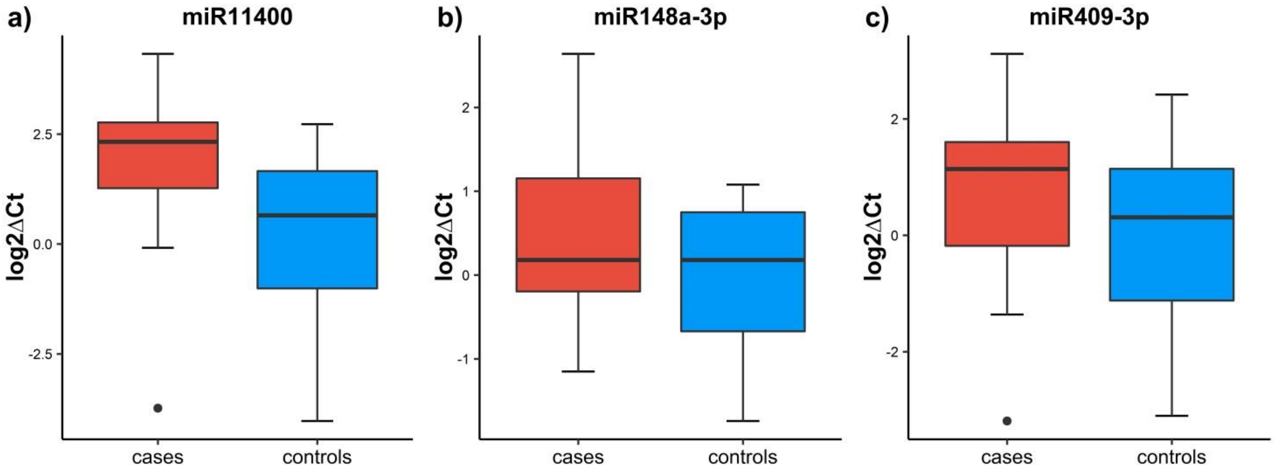

Supplementary Figure S3. Results of RT-qPCR validation of miR-11400 (a), miR-148a-3p (b) and miR-409-3p (c) in 19 pre-clinical MPM samples and 19 matched controls. Box-and-whiskers plots present the first quartile, median and third quartile, minimum and maximum values and outliers that are marked outside the whiskers range. Wilcoxon's test revealed no significant difference between cases and controls.

Supplementary Tables

Supplementary Table S1. Primary sequence of miRNAs analysed by RT-qPCR.

miR-92b-3p	5'-UAUUGCACUCGUCCCGGCCUGU-3'
miR-148a	5'-UCAGUGCACUACAGAACUUUGU-3'
miR-409-3p	5'-GAAUGUUGCUCGGUGAACCCCU-3'
miR-11400	5'-UCGGCUGUGUAUCUCUGUGUC-3'

Supplementary Table S2. MiRNA NGS pre-processing statistics.

SAMPLE	raw reads[a]	cutadapt short reads[b]	cutadapt short reads/raw reads	cutapdapt adapter reads[c]	cutapdapt adapter reads/raw reads	clean reads[d]	clean reads/raw reads	aligned[e]	aligned/clean reads	aligned/raw reads	well aligned[f]	well aligned/aligned	well aligned/raw reads
EPIC_1	1E+07	3320936	0.321	10299067	0.996	7019306	0.679	3326816	0.474	0.322	651035	0.196	0.063
EPIC_2	1E+07	3534463	0.347	10175702	0.999	6655746	0.653	2629276	0.395	0.258	745546	0.284	0.073
EPIC_3	8E+06	2787601	0.358	7757391	0.997	4995032	0.642	2056570	0.412	0.264	252970	0.123	0.033
EPIC_4	1E+07	5263965	0.379	13846188	0.997	8628392	0.621	2923891	0.339	0.210	139768	0.048	0.010
EPIC_5	1E+07	3373152	0.346	9729351	0.999	6370430	0.654	2392638	0.376	0.246	477563	0.200	0.049
EPIC_6	2E+07	4458847	0.270	16440279	0.996	12048500	0.730	5550276	0.461	0.336	843778	0.152	0.051
EPIC_7	4E+06	2080166	0.465	4456365	0.997	2389116	0.535	1146159	0.480	0.256	173250	0.151	0.039
EPIC_8	2E+07	4658859	0.303	15336691	0.996	10736170	0.697	5255760	0.490	0.341	1143626	0.218	0.074
EPIC_9	1E+07	3584813	0.315	11336144	0.997	7784812	0.685	2953060	0.379	0.260	187221	0.063	0.016
EPIC_10	1E+07	4644433	0.350	13170678	0.993	8616495	0.650	4055435	0.471	0.306	439099	0.108	0.033
EPIC_11	1E+07	1677671	0.171	9792948	0.997	8146003	0.829	4266639	0.524	0.434	1190653	0.279	0.121
EPIC_12	6E+06	2232965	0.370	6018288	0.998	3794539	0.630	1396413	0.368	0.232	146040	0.105	0.024
EPIC_13	7E+06	1162702	0.160	7228287	0.996	6091739	0.840	3446020	0.566	0.475	782467	0.227	0.108

SAMPLE	raw reads[a]	cutadapt short reads[b]	cutadapt short reads/raw reads	cutapdapt adapter reads[c]	cutapdapt adapter reads/raw reads	clean reads[d]	clean reads/raw reads	aligned[e]	aligned/clean reads	aligned/raw reads	well aligned[f]	well aligned/aligned	well aligned/raw reads
EPIC_14	7E+06	1308083	0.191	6830754	0.997	5541298	0.809	2692535	0.486	0.393	640383	0.238	0.093
EPIC_15	1E+07	6052235	0.561	10754157	0.997	4735100	0.439	2750800	0.581	0.255	923881	0.336	0.086
EPIC_16	3E+06	1005379	0.336	2971919	0.995	1982521	0.664	795750	0.401	0.266	24251	0.030	0.008
EPIC_17	5E+06	1750707	0.374	4681043	0.999	2935307	0.626	865542	0.295	0.185	39420	0.046	0.008
EPIC_18	1E+07	4243148	0.337	12563505	0.997	8357012	0.663	3317082	0.397	0.263	369236	0.111	0.029
EPIC_19	3E+06	452188	0.150	3004579	0.999	2556003	0.850	1364957	0.534	0.454	308142	0.226	0.102
EPIC_20	4E+06	1028379	0.274	3743489	0.999	2720034	0.726	1346696	0.495	0.359	192797	0.143	0.051
EPIC_21	7E+06	2577526	0.377	6828867	0.998	4265209	0.623	1447496	0.339	0.212	204893	0.142	0.030
EPIC_22	3E+07	15807681	0.515	30496418	0.994	14873539	0.485	5794051	0.390	0.189	793810	0.137	0.026
EPIC_23	3E+06	859931	0.279	3072285	0.997	2220406	0.721	1337711	0.602	0.434	648586	0.485	0.211
EPIC_24	1E+07	2037833	0.183	11116727	0.996	9122129	0.817	4992865	0.547	0.447	1537314	0.308	0.138
EPIC_25	7E+06	1303320	0.190	6826254	0.997	5543149	0.810	2490455	0.449	0.364	313871	0.126	0.046
EPIC_26	1E+07	4236576	0.339	12452959	0.998	8246555	0.661	2982299	0.362	0.239	251956	0.084	0.020
EPIC_27	8E+06	2661497	0.320	8291691	0.996	5661079	0.680	2254894	0.398	0.271	244768	0.109	0.029
EPIC_28	5E+06	1966658	0.374	5246241	0.997	3295037	0.626	1232845	0.374	0.234	126261	0.102	0.024

SAMPLE	raw reads[a]	cutadapt short reads[b]	cutadapt short reads /raw reads	cutapdapt adapter reads[c]	cutapdapt adapter reads/raw reads	clean reads[d]	clean reads/raw reads	aligned[e]	aligned/clean reads	aligned/raw reads	well aligned[f]	well aligned/aligned	well aligned/raw reads
EPIC_29	6E+06	1217687	0.189	6440452	0.998	5234843	0.811	2813848	0.538	0.436	508151	0.181	0.079
EPIC_30	1E+07	3742213	0.325	11502248	0.999	7775404	0.675	2719547	0.350	0.236	274288	0.101	0.024
EPIC_31	1E+07	4087246	0.382	10666362	0.997	6607108	0.618	2304639	0.349	0.216	161093	0.070	0.015
EPIC_32	4E+06	629254	0.156	4029376	0.999	3404952	0.844	1648963	0.484	0.409	224328	0.136	0.056
EPIC_33	2E+07	4947964	0.320	15324390	0.992	10507536	0.680	5280924	0.503	0.342	1093390	0.207	0.071
EPIC_34	9E+06	1275809	0.146	8678217	0.996	7434412	0.854	4533297	0.610	0.520	1559515	0.344	0.179
EPIC_35	2E+07	5791072	0.309	18671884	0.996	12958233	0.691	6157193	0.475	0.328	870199	0.141	0.046
EPIC_36	1E+07	2009857	0.183	10931243	0.997	8956444	0.817	4157716	0.464	0.379	738241	0.178	0.067
EPIC_37	5E+06	1807507	0.337	5352958	0.999	3552460	0.663	1356746	0.382	0.253	249487	0.184	0.047
EPIC_38	2E+07	3996814	0.207	19159453	0.994	15283810	0.793	7641547	0.500	0.396	1853999	0.243	0.096
EPIC_39	1E+07	3371192	0.337	9998778	0.998	6643136	0.663	3488054	0.525	0.348	769264	0.221	0.077
EPIC_40	1E+07	4161786	0.306	13560426	0.996	9451059	0.694	4463967	0.472	0.328	1118638	0.251	0.082
EPIC_41	1E+07	2741423	0.286	9564739	0.997	6848659	0.714	2643886	0.386	0.276	227285	0.086	0.024
EPIC_42	1E+07	3912251	0.361	10808756	0.998	6915098	0.639	2929727	0.424	0.271	564509	0.193	0.052
EPIC_43	1E+07	2152472	0.166	12919556	0.996	10824363	0.834	6288403	0.581	0.485	1665209	0.265	0.128

SAMPLE	raw reads[a]	cutadapt short reads[b]	cutadapt short reads /raw reads	cutapdapt adapter reads[c]	cutapdapt adapter reads/raw reads	clean reads[d]	clean reads/raw reads	aligned[e]	aligned/clean reads	aligned/raw reads	well aligned[f]	well aligned/aligned	well aligned/raw reads
EPIC_44	6E+06	1471603	0.242	6070527	0.999	4606546	0.758	2144691	0.466	0.353	356293	0.166	0.059
EPIC_45	1E+07	4134486	0.355	11592972	0.996	7503987	0.645	2937807	0.391	0.252	402245	0.137	0.035
EPIC_46	2E+07	5221036	0.289	18003476	0.997	12829765	0.711	5072030	0.395	0.281	347989	0.069	0.019
EPIC_47	9E+06	2792040	0.322	8647123	0.999	5866061	0.678	2589914	0.442	0.299	564706	0.218	0.065
EPIC_48	2E+07	4161158	0.241	17187633	0.997	13069968	0.759	5570858	0.426	0.323	512999	0.092	0.030
EPIC_49	1E+07	4640829	0.331	14008753	0.998	9395180	0.669	4338882	0.462	0.309	799378	0.184	0.057
EPIC_50	7E+06	2258026	0.324	6958257	0.999	4708970	0.676	1639754	0.348	0.235	253354	0.155	0.036
EPIC_51	8E+06	2293348	0.303	7554373	0.999	5269998	0.697	2145240	0.407	0.284	158710	0.074	0.021
EPIC_52	4E+06	739276	0.167	4429919	0.999	3696781	0.833	2037162	0.551	0.459	537431	0.264	0.121
EPIC_53	6E+06	2047254	0.354	5778640	0.999	3739376	0.646	1315733	0.352	0.227	156104	0.119	0.027
EPIC_54	7E+06	2195801	0.330	6649502	0.998	4464395	0.670	1786150	0.400	0.268	189549	0.106	0.028
EPIC_55	6E+06	965860	0.175	5515272	0.998	4561232	0.825	2554351	0.560	0.462	462131	0.181	0.084
EPIC_56	4E+06	670441	0.156	4285746	0.998	3621981	0.844	1927428	0.532	0.449	374944	0.195	0.087
EPIC_57	8E+06	2218284	0.262	8418268	0.994	6253892	0.738	3614618	0.578	0.427	518623	0.143	0.061
EPIC_58	5E+06	1563015	0.301	5157276	0.993	3631947	0.699	2483146	0.684	0.478	564108	0.227	0.109

SAMPLE	raw reads[a]	cutadapt short reads[b]	cutadapt short reads /raw reads	cutapdapt adapter reads[c]	cutapdapt adapter reads/raw reads	clean reads[d]	clean reads/raw reads	aligned[e]	aligned/clean reads	aligned/raw reads	well aligned[f]	well aligned/aligned	well aligned/raw reads
EPIC_59	9E+06	2005135	0.232	8638047	0.999	6644587	0.768	3832487	0.577	0.443	1808053	0.472	0.209
EPIC_60	3E+06	1216912	0.350	3453196	0.993	2261701	0.650	1113146	0.492	0.320	143767	0.129	0.041
EPIC_61	6E+06	2402735	0.392	6088155	0.993	3727962	0.608	2370053	0.636	0.387	719016	0.303	0.117
EPIC_62	6E+06	2403773	0.410	5815637	0.991	3464635	0.590	1791192	0.517	0.305	274490	0.153	0.047
EPIC_63	1E+07	3238751	0.289	11210919	0.999	7986029	0.711	3061064	0.383	0.273	560448	0.183	0.050
EPIC_64	1E+07	3846000	0.326	11738299	0.996	7942484	0.674	3879887	0.488	0.329	374578	0.097	0.032
EPIC_65	6E+06	1911299	0.302	6315738	0.999	4412346	0.698	1423636	0.323	0.225	90066	0.063	0.014
EPIC_66	1E+07	5192903	0.382	13495162	0.992	8405050	0.618	4458624	0.530	0.328	879894	0.197	0.065
EPIC_67	1E+07	4984008	0.368	13482096	0.995	8562833	0.632	3817258	0.446	0.282	295092	0.077	0.022
EPIC_68	1E+07	4534946	0.362	12461193	0.996	7981016	0.638	4136268	0.518	0.330	504220	0.122	0.040
EPIC_69	2E+07	5906164	0.367	15983860	0.994	10167766	0.633	4494821	0.442	0.280	393017	0.087	0.024
EPIC_70	3E+06	1328350	0.388	3405137	0.995	2095117	0.612	866145	0.413	0.253	30898	0.036	0.009
EPIC_71	2E+07	4434147	0.239	18508259	0.997	14133071	0.761	5375023	0.380	0.289	306902	0.057	0.017
EPIC_72	2E+07	4749690	0.235	20108115	0.996	15447565	0.765	7022266	0.455	0.348	1041865	0.148	0.052
EPIC_73	1E+07	1819125	0.191	9469308	0.995	7695502	0.809	4088975	0.531	0.430	774725	0.189	0.081

SAMPLE	raw reads[a]	cutadapt short reads[b]	cutadapt short reads/raw reads	cutapdapt adapter reads[c]	cutapdapt adapter reads/raw reads	clean reads[d]	clean reads/raw reads	aligned[e]	aligned/clean reads	aligned/raw reads	well aligned[f]	well aligned/aligned	well aligned/raw reads
EPIC_74	5E+06	926899	0.200	4627952	0.999	3707562	0.800	1859019	0.501	0.401	349537	0.188	0.075
EPIC_75	1E+07	1738448	0.156	11108236	0.996	9413456	0.844	5054657	0.537	0.453	990747	0.196	0.089
EPIC_76	5E+06	857991	0.176	4868172	0.998	4017646	0.824	2300615	0.573	0.472	398025	0.173	0.082
EPIC_77	3E+06	470228	0.162	2904177	0.999	2437575	0.838	1136535	0.466	0.391	154675	0.136	0.053
EPIC_78	8E+06	2565135	0.315	8132764	0.998	5581168	0.685	2068944	0.371	0.254	106427	0.051	0.013
EPIC_79	8E+06	2795948	0.354	7873266	0.997	5099281	0.646	2012937	0.395	0.255	246634	0.123	0.031
EPIC_80	2E+07	5206572	0.267	19462850	0.997	14321688	0.733	8165669	0.570	0.418	2856284	0.350	0.146
EPIC_81	1E+07	4454262	0.352	12590754	0.995	8203681	0.648	3070512	0.374	0.243	255999	0.083	0.020
EPIC_82	2E+06	1027265	0.427	2402837	0.998	1380695	0.573	592117	0.429	0.246	68423	0.116	0.028
EPIC_83	1E+07	3696701	0.263	13971543	0.995	10344744	0.737	5485371	0.530	0.391	1087071	0.198	0.077
EPIC_84	1E+07	4281379	0.360	11857828	0.998	7595456	0.640	2551803	0.336	0.215	406499	0.159	0.034
EPIC_85	2E+07	5334623	0.337	15791276	0.997	10504412	0.663	3632625	0.346	0.229	159601	0.044	0.010
EPIC_86	8E+06	2550739	0.317	7999704	0.995	5491514	0.683	1977481	0.360	0.246	58964	0.030	0.007
EPIC_87	3E+06	1112692	0.352	3159661	0.999	2051364	0.648	635002	0.310	0.201	43024	0.068	0.014
EPIC_88	1E+07	2836598	0.197	14343684	0.997	11555916	0.803	5803740	0.502	0.403	1305966	0.225	0.091

SAMPLE	raw reads[a]	cutadapt short reads[b]	cutadapt short reads /raw reads	cutapdapt adapter reads[c]	cutapdapt adapter reads/raw reads	clean reads[d]	clean reads/raw reads	aligned[e]	aligned/clean reads	aligned/raw reads	well aligned[f]	well aligned/aligned	well aligned/raw reads
EPIC_89	6E+06	3023492	0.505	5967267	0.997	2960202	0.495	1232493	0.416	0.206	105351	0.085	0.018
EPIC_90	2E+07	4820613	0.310	15498018	0.997	10719911	0.690	4768806	0.445	0.307	619922	0.130	0.040
EPIC_91	1E+07	3308849	0.342	9641756	0.996	6367151	0.658	2443780	0.384	0.253	177130	0.072	0.018
EPIC_92	1E+07	4512293	0.335	13425528	0.996	8966746	0.665	4245206	0.473	0.315	350026	0.082	0.026
EPIC_93	1E+07	2694604	0.263	10180669	0.995	7539129	0.737	3100818	0.411	0.303	430244	0.139	0.042
EPIC_94	9E+06	3234614	0.369	8750103	0.998	5528666	0.631	2369784	0.429	0.270	518034	0.219	0.059
EPIC_95	6E+06	1069547	0.189	5647135	0.997	4593934	0.811	2298219	0.500	0.406	580110	0.252	0.102
EPIC_96	1E+07	3616234	0.278	12977671	0.997	9403455	0.722	4624862	0.492	0.355	1536281	0.332	0.118
EPIC_97	1E+07	8965724	0.699	12786732	0.997	3864677	0.301	1745407	0.452	0.136	357938	0.205	0.028
EPIC_98	3E+06	1012975	0.357	2822664	0.996	1821565	0.643	743671	0.408	0.262	34583	0.047	0.012
EPIC_99	4E+06	1320673	0.332	3969649	0.999	2653804	0.668	861550	0.325	0.217	92449	0.107	0.023
EPIC_100	1E+07	4262288	0.403	10538305	0.997	6306020	0.597	2456900	0.390	0.232	255352	0.104	0.024
EPIC_101	5E+06	1876284	0.381	4915152	0.998	3050003	0.619	1513495	0.496	0.307	324685	0.215	0.066
EPIC_102	4E+06	882253	0.220	4009913	0.998	3134690	0.780	1622445	0.518	0.404	339012	0.209	0.084
EPIC_103	3E+06	894889	0.322	2777701	0.999	1886056	0.678	824881	0.437	0.297	236696	0.287	0.085

SAMPLE	raw reads[a]	cutadapt short reads[b]	cutadapt short reads/raw reads	cutapdapt adapter reads[c]	cutapdapt adapter reads/raw reads	clean reads[d]	clean reads/raw reads	aligned[e]	aligned/clean reads	aligned/raw reads	well aligned[f]	well aligned/aligned	well aligned/raw reads
EPIC_104	1E+07	5317006	0.396	13332308	0.994	8098530	0.604	3324844	0.411	0.248	202588	0.061	0.015
EPIC_105	4E+06	1528459	0.354	4312898	0.999	2789779	0.646	1131601	0.406	0.262	366560	0.324	0.085
EPIC_106	1E+07	1941858	0.182	10645521	0.996	8748607	0.818	4796647	0.548	0.449	1342226	0.280	0.126
EPIC_107	1E+07	1629878	0.165	9820782	0.997	8225295	0.835	4376395	0.532	0.444	845687	0.193	0.086
EPIC_108	1E+07	5203015	0.381	13605040	0.997	8441336	0.619	3047301	0.361	0.223	272210	0.089	0.020
EPIC_109	6E+06	2108152	0.382	5504353	0.997	3412528	0.618	1315029	0.385	0.238	161137	0.123	0.029
EPIC_110	1E+07	5174619	0.409	12608993	0.998	7461937	0.591	2547802	0.341	0.202	129012	0.051	0.010
EPIC_111	6E+06	980806	0.156	6286791	0.999	5314846	0.844	3058169	0.575	0.486	766009	0.250	0.122
EPIC_112	1E+07	5611098	0.388	14421525	0.998	8833829	0.612	2917083	0.330	0.202	413345	0.142	0.029
EPIC_113	2E+07	5189191	0.319	16231411	0.997	11088963	0.681	3914732	0.353	0.240	203259	0.052	0.012
EPIC_114	3E+06	752064	0.229	3280308	0.998	2533590	0.771	1332464	0.526	0.406	321631	0.241	0.098
EPIC_115	2E+07	1971023	0.105	18615810	0.996	16724426	0.895	11530627	0.689	0.617	2995420	0.260	0.160
EPIC_116	1E+07	2302308	0.230	9946251	0.994	7699302	0.770	4674233	0.607	0.467	1195407	0.256	0.120
EPIC_117	2E+06	614690	0.283	2166323	0.996	1559748	0.717	645624	0.414	0.297	120251	0.186	0.055
EPIC_118	2E+07	2967234	0.151	19628818	0.996	16742600	0.849	9966139	0.595	0.506	2267630	0.228	0.115

SAMPLE	raw reads[a]	cutadapt short reads[b]	cutadapt short reads/raw reads	cutapdapt adapter reads[c]	cutapdapt adapter reads/raw reads	clean reads[d]	clean reads/raw reads	aligned[e]	aligned/clean reads	aligned/raw reads	well aligned[f]	well aligned/aligned	well aligned/raw reads
EPIC_119	7E+06	2592389	0.356	7271216	0.998	4692057	0.644	1921317	0.409	0.264	517447	0.269	0.071
EPIC_120	2E+07	3207703	0.154	20794026	0.995	17684735	0.846	10103684	0.571	0.484	2040840	0.202	0.098
EPIC_121	1E+07	4722427	0.328	14390597	0.999	9689042	0.672	4153721	0.429	0.288	570443	0.137	0.040
EPIC_122	1E+07	3248736	0.318	10172768	0.997	6953391	0.682	2801713	0.403	0.275	490301	0.175	0.048
EPIC_123	2E+07	4916453	0.277	17687947	0.997	12820911	0.723	4915659	0.383	0.277	279734	0.057	0.016
EPIC_124	1E+07	3555403	0.329	10778104	0.998	7239531	0.671	3170005	0.438	0.294	483653	0.153	0.045
EPIC_125	1E+07	1520074	0.103	14650745	0.995	13207873	0.897	9460077	0.716	0.642	2994167	0.317	0.203
EPIC_126	5E+06	1141640	0.220	5191131	0.998	4058656	0.780	2164085	0.533	0.416	374754	0.173	0.072
EPIC_127	2E+07	5651169	0.306	18436740	0.998	12831390	0.694	4779265	0.372	0.259	297224	0.062	0.016
EPIC_128	6E+06	2093095	0.334	6254225	0.997	4182019	0.666	1524921	0.365	0.243	134641	0.088	0.021
EPIC_129	7E+06	2598073	0.359	7229256	0.998	4642437	0.641	1897362	0.409	0.262	300036	0.158	0.041
EPIC_130	7E+06	2016460	0.274	7345436	0.997	5347694	0.726	2161394	0.404	0.294	265154	0.123	0.036
EPIC_131	2E+07	4386928	0.274	15996695	0.998	11642579	0.726	4665573	0.401	0.291	453031	0.097	0.028
EPIC_132	9E+06	3100529	0.345	8981914	0.999	5893052	0.655	2116957	0.359	0.235	294512	0.139	0.033
EPIC_133	2E+06	415170	0.184	2256180	0.999	1844290	0.816	879473	0.477	0.389	128899	0.147	0.057

SAMPLE	raw reads[a]	cutadapt short reads[b]	cutadapt short reads /raw reads	cutapdapt adapter reads[c]	cutapdapt adapter reads/raw reads	clean reads[d]	clean reads/raw reads	aligned[e]	aligned/clean reads	aligned/raw reads	well aligned[f]	well aligned/aligned	well aligned/raw reads
EPIC_134	5E+06	1294148	0.239	5405534	0.998	4120424	0.761	2410896	0.585	0.445	697956	0.290	0.129
EPIC_135	1E+07	4887339	0.355	13732827	0.998	8871279	0.645	2811015	0.317	0.204	257721	0.092	0.019
EPIC_136	6E+06	1504031	0.246	6102361	0.998	4608141	0.754	2466007	0.535	0.403	288042	0.117	0.047
EPIC_137	7E+06	1242652	0.166	7477256	0.998	6248411	0.834	3901316	0.624	0.521	778727	0.200	0.104
EPIC_138	3E+06	406151	0.127	3185285	0.999	2783744	0.873	1641513	0.590	0.515	269341	0.164	0.084
EPIC_139	2E+07	4610898	0.305	15053524	0.994	10527540	0.695	4824770	0.458	0.319	480055	0.099	0.032
EPIC_140	1E+07	4636035	0.440	10464704	0.994	5889442	0.560	2864349	0.486	0.272	416727	0.145	0.040
EPIC_141	4E+06	1439179	0.335	4287041	0.999	2854116	0.665	945990	0.331	0.220	102023	0.108	0.024
EPIC_142	4E+06	1464977	0.352	4133009	0.993	2696746	0.648	1761596	0.653	0.423	429876	0.244	0.103
EPIC_143	2E+07	4858343	0.299	16165724	0.994	11399897	0.701	7982761	0.700	0.491	3749986	0.470	0.231
EPIC_144	6E+06	1946955	0.328	5892044	0.992	3991515	0.672	2532454	0.634	0.426	616456	0.243	0.104
EPIC_145	7E+06	2306528	0.319	7213176	0.998	4917702	0.681	1616881	0.329	0.224	132944	0.082	0.018
EPIC_146	1E+07	5024980	0.366	13667418	0.995	8705312	0.634	3962344	0.455	0.289	380419	0.096	0.028
EPIC_147	5E+06	1451986	0.292	4963252	0.999	3517547	0.708	1125832	0.320	0.227	85773	0.076	0.017
EPIC_148	2E+07	5391913	0.317	16920040	0.996	11600741	0.683	5069160	0.437	0.298	314835	0.062	0.019

SAMPLE	raw reads[a]	cutadapt short reads[b]	cutadapt short reads /raw reads	cutapdapt adapter reads[c]	cutapdapt adapter reads/raw reads	clean reads[d]	clean reads/raw reads	aligned[e]	aligned/clean reads	aligned/raw reads	well aligned[f]	well aligned/aligned	well aligned/raw reads
EPIC_149	3E+07	8689487	0.301	28757292	0.995	20222698	0.699	8937345	0.442	0.309	509129	0.057	0.018
EPIC_150	1E+07	3658669	0.282	12918065	0.996	9307606	0.718	4255712	0.457	0.328	293304	0.069	0.023
EPIC_151	3E+06	1284465	0.385	3317513	0.994	2053957	0.615	841638	0.410	0.252	28499	0.034	0.009
EPIC_152	1E+07	4563988	0.327	13743700	0.983	9411194	0.673	4368239	0.464	0.313	269566	0.062	0.019
EPIC_153	1E+07	3032798	0.217	13936867	0.996	10953447	0.783	4618496	0.422	0.330	449406	0.097	0.032
EPIC_154	3E+07	5975301	0.189	31487308	0.996	25646962	0.811	10533441	0.411	0.333	783265	0.074	0.025
EPIC_155	1E+07	2257018	0.222	10112936	0.995	7904927	0.778	3693792	0.467	0.363	511321	0.138	0.050
EPIC_156	6E+06	1903065	0.324	5870435	0.998	3977867	0.676	2171297	0.546	0.369	251496	0.116	0.043
EPIC_157	4E+06	801045	0.192	4156760	0.996	3370724	0.808	1472527	0.437	0.353	171205	0.116	0.041
EPIC_158	5E+06	939043	0.179	5237576	0.999	4305304	0.821	2081080	0.483	0.397	251237	0.121	0.048
EPIC_159	4E+06	746623	0.200	3730780	0.999	2988749	0.800	1358564	0.455	0.364	143572	0.106	0.038
EPIC_160	9E+06	2656563	0.303	8758322	0.998	6115061	0.697	2394741	0.392	0.273	161789	0.068	0.018
EPIC_161	1E+07	4338398	0.395	10944937	0.997	6638862	0.605	2288499	0.345	0.208	92382	0.040	0.008
EPIC_162	1E+07	4948621	0.342	14359990	0.993	9519371	0.658	4388820	0.461	0.303	821649	0.187	0.057
EPIC_163	1E+07	4202704	0.363	11539231	0.997	7370965	0.637	2595354	0.352	0.224	165139	0.064	0.014

**SAMPLE**	**raw reads**[a]	**cutadapt short reads**[b]	**cutadapt short reads /raw reads**	**cutapdapt adapter reads**[c]	**cutapdapt adapter reads/raw reads**	**clean reads**[d]	**clean reads/raw reads**	**aligned**[e]	**aligned/clean reads**	**aligned/raw reads**	**well aligned**[f]	**well aligned/aligned**	**well aligned/raw reads**
EPIC_164	4E+06	821755	0.188	4357654	0.998	3542572	0.812	1683672	0.475	0.386	190997	0.113	0.044

[a]raw reads
[b]cutadapt short reads
[c]cutapdapt adapter reads

[d]clean reads

[e]aligned

[f]well aligned

Supplementary Table S3. Differentially expressed miRNAs between 80 pre-diagnostic MPM cases and 80 matched controls from NGS. Table shows the average expression for all identified miRNAs (base Mean), the differential expression reported as log2 fold change between cases and controls (Log2FC), the p-value from statistical analysis (p-value) and the p-value adjusted for multiple testing by FDR (p-adj).

ID	baseMean	log2FoldChange	p-value	FDR
**hsa-let-7a-3p**	45.58	-0.045	0.607	0.96
**hsa-miR-323b-3p**	27.87	0.138	0.573	0.96
**hsa-miR-330-3p**	59.49	0.224	0.103	0.96
**hsa-miR-335-3p**	22.61	0.256	0.144	0.96
**hsa-miR-335-5p**	21.58	0.111	0.399	0.96
**hsa-miR-339-3p**	22.85	-0.051	0.680	0.96
**hsa-miR-339-5p**	29.33	0.057	0.695	0.96
**hsa-miR-340-5p**	116.36	0.065	0.443	0.96
**hsa-miR-342-3p**	26.65	0.132	0.362	0.96
**hsa-miR-342-5p**	101.32	0.066	0.585	0.96
**hsa-miR-345-5p**	18.94	-0.091	0.508	0.96
**hsa-miR-361-3p**	100.87	0.078	0.392	0.96
**hsa-miR-361-5p**	17.17	0.076	0.580	0.96
**hsa-miR-3613-5p**	34.39	0.110	0.477	0.96
**hsa-miR-3615**	606.27	-0.049	0.646	0.96
**hsa-miR-363-3p**	376.84	-0.106	0.333	0.96
**hsa-miR-370-3p**	90.41	-0.122	0.555	0.96
**hsa-miR-374b-5p**	13.02	0.190	0.264	0.96
**hsa-miR-375-3p**	149.22	-0.194	0.320	0.96
**hsa-miR-378a-3p**	461.55	-0.066	0.487	0.96
**hsa-miR-320d**	58.31	-0.065	0.691	0.96
**hsa-miR-378c**	84.17	-0.041	0.698	0.96
**hsa-miR-320b**	1092.46	-0.039	0.685	0.96
**hsa-miR-32-5p**	29.01	-0.175	0.113	0.96
**hsa-miR-222-3p**	287.01	0.052	0.426	0.96
**hsa-miR-223-3p**	103.13	0.053	0.715	0.96
**hsa-miR-223-5p**	507.44	-0.093	0.448	0.96
**hsa-miR-224-5p**	94.85	0.157	0.399	0.96
**hsa-miR-23a-3p**	82.02	0.104	0.424	0.96
**hsa-miR-24-2-5p**	20.91	0.202	0.072	0.96
**hsa-miR-24-3p**	1768.35	-0.089	0.154	0.96
**hsa-miR-25-3p**	3748.06	-0.135	0.248	0.96
**hsa-miR-26a-5p**	9889.16	0.129	0.229	0.96
**hsa-miR-26b-5p**	610.44	0.070	0.355	0.96
**hsa-miR-27a-3p**	1085.40	-0.094	0.404	0.96
**hsa-miR-99a-5p**	1578.46	0.084	0.524	0.96
**hsa-miR-28-3p**	240.54	0.078	0.370	0.96
**hsa-miR-28-5p**	28.68	0.140	0.333	0.96
**hsa-miR-30b-5p**	15.56	0.161	0.219	0.96
**hsa-miR-30c-5p**	336.54	0.034	0.709	0.96
**hsa-miR-30e-3p**	129.71	0.037	0.728	0.96
**hsa-miR-30e-5p**	939.50	-0.058	0.482	0.96

**hsa-miR-3158-3p**	36.77	-0.310	0.085	0.96
**hsa-miR-320a-3p**	4702.58	-0.093	0.300	0.96
**hsa-miR-221-3p**	394.60	0.105	0.203	0.96
**hsa-miR-382-5p**	71.99	-0.071	0.719	0.96
**hsa-miR-423-5p**	13919.98	-0.075	0.442	0.96
**hsa-miR-505-5p**	22.41	0.144	0.229	0.96
**hsa-miR-511-5p**	19.90	0.121	0.531	0.96
**hsa-miR-532-5p**	302.61	-0.108	0.309	0.96
**hsa-miR-550a-5p**	12.35	-0.342	0.038	0.96
**hsa-miR-625-3p**	143.63	-0.068	0.618	0.96
**hsa-miR-628-3p**	19.74	0.098	0.439	0.96
**hsa-miR-629-5p**	188.28	-0.134	0.220	0.96
**hsa-miR-652-3p**	29.48	0.174	0.228	0.96
**hsa-miR-654-3p**	29.65	-0.192	0.368	0.96
**hsa-miR-654-5p**	14.43	-0.095	0.698	0.96
**hsa-miR-671-3p**	30.26	0.202	0.212	0.96
**hsa-miR-6842-3p**	19.13	0.158	0.267	0.96
**hsa-miR-7-5p**	305.55	-0.263	0.082	0.96
**hsa-miR-92a-3p**	12475.53	-0.119	0.236	0.96
**hsa-miR-92b-3p**	96.77	-0.107	0.278	0.96
**hsa-miR-93-5p**	186.41	-0.146	0.154	0.96
**hsa-miR-941**	82.27	0.120	0.340	0.96
**hsa-miR-942-5p**	17.65	-0.222	0.102	0.96
**hsa-miR-98-5p**	193.20	0.047	0.692	0.96
**hsa-miR-502-3p**	14.40	0.181	0.279	0.96
**hsa-miR-423-3p**	1750.59	-0.074	0.309	0.96
**hsa-miR-501-3p**	78.42	0.118	0.431	0.96
**hsa-miR-493-3p**	26.52	-0.122	0.613	0.96
**hsa-miR-425-3p**	15.86	-0.239	0.078	0.96
**hsa-miR-425-5p**	256.94	0.031	0.666	0.96
**hsa-miR-432-5p**	53.99	-0.206	0.354	0.96
**hsa-miR-433-3p**	25.38	-0.170	0.474	0.96
**hsa-miR-4433b-3p**	48.98	-0.126	0.654	0.96
**hsa-miR-4433b-5p**	88.06	-0.142	0.433	0.96
**hsa-miR-4443**	99.33	0.164	0.334	0.96
**hsa-miR-4446-3p**	29.19	0.070	0.704	0.96
**hsa-miR-4488**	17.05	-0.182	0.416	0.96
**hsa-miR-4497**	26.35	-0.279	0.205	0.96
**hsa-miR-4508**	223.96	0.114	0.606	0.96
**hsa-miR-4516**	23.95	0.096	0.660	0.96
**hsa-miR-451a**	45496.02	-0.077	0.659	0.96
**hsa-miR-4732-3p**	38.06	-0.074	0.686	0.96
**hsa-miR-4732-5p**	49.97	-0.101	0.541	0.96
**hsa-miR-483-5p**	39.18	0.095	0.658	0.96
**hsa-miR-485-5p**	33.79	-0.079	0.710	0.96
**hsa-miR-486-3p**	159.19	-0.160	0.232	0.96
**hsa-miR-486-5p**	52351.70	-0.066	0.674	0.96

**hsa-miR-495-3p**	22.27	-0.118	0.581	0.96
**hsa-miR-22-5p**	77.20	-0.107	0.278	0.96
**hsa-miR-27a-5p**	14.48	-0.154	0.462	0.96
**hsa-miR-215-5p**	50.65	-0.180	0.268	0.96
**hsa-miR-126-5p**	124.33	0.031	0.725	0.96
**hsa-miR-1301-3p**	65.63	0.083	0.498	0.96
**hsa-miR-1307-3p**	456.22	-0.054	0.541	0.96
**hsa-miR-130b-5p**	34.99	-0.058	0.700	0.96
**hsa-miR-139-3p**	40.96	0.073	0.608	0.96
**hsa-miR-139-5p**	191.66	0.213	0.023	0.96
**hsa-miR-140-3p**	679.74	-0.112	0.261	0.96
**hsa-miR-140-5p**	46.37	-0.095	0.338	0.96
**hsa-miR-126-3p**	7395.61	0.041	0.624	0.96
**hsa-miR-142-3p**	145.27	0.056	0.570	0.96
**hsa-miR-144-3p**	180.26	-0.057	0.668	0.96
**hsa-miR-144-5p**	74.53	-0.086	0.532	0.96
**hsa-miR-145-3p**	30.83	-0.109	0.439	0.96
**hsa-miR-146b-5p**	628.56	0.102	0.188	0.96
**hsa-miR-148a-3p**	8221.05	-0.084	0.450	0.96
**hsa-miR-148b-3p**	333.13	0.044	0.547	0.96
**hsa-miR-150-5p**	156.62	0.158	0.283	0.96
**hsa-miR-151a-5p**	18.01	0.247	0.156	0.96
**hsa-miR-142-5p**	1451.60	-0.099	0.356	0.96
**hsa-miR-155-5p**	51.98	0.101	0.315	0.96
**hsa-miR-125b-5p**	105.84	0.056	0.667	0.96
**hsa-miR-1228-5p**	26.42	-0.132	0.579	0.96
**hsa-let-7a-5p**	4650.26	0.027	0.691	0.96
**hsa-let-7b-3p**	14.01	-0.118	0.417	0.96
**hsa-let-7b-5p**	8901.25	-0.074	0.506	0.96
**hsa-let-7c-5p**	656.98	-0.044	0.595	0.96
**hsa-let-7d-5p**	686.33	0.057	0.428	0.96
**hsa-let-7e-5p**	109.79	0.121	0.382	0.96
**hsa-let-7f-5p**	5222.16	0.055	0.505	0.96
**hsa-let-7g-5p**	2514.28	0.027	0.650	0.96
**hsa-miR-125a-5p**	321.45	0.151	0.138	0.96
**hsa-miR-100-5p**	271.77	-0.469	0.039	0.96
**hsa-miR-103a-3p**	356.30	0.029	0.713	0.96
**hsa-miR-106b-3p**	195.74	-0.106	0.184	0.96
**hsa-miR-106b-5p**	37.43	-0.168	0.278	0.96
**hsa-miR-107**	105.85	-0.046	0.711	0.96
**hsa-miR-10a-5p**	1095.94	-0.069	0.586	0.96
**hsa-miR-10b-5p**	1834.93	0.093	0.467	0.96
**hsa-miR-11400**	29.02	-0.105	0.673	0.96
**hsa-miR-122-5p**	15710.86	0.081	0.728	0.96
**hsa-miR-101-3p**	1427.82	-0.167	0.170	0.96
**hsa-miR-15a-5p**	14.37	0.115	0.566	0.96
**hsa-miR-99b-5p**	392.23	0.148	0.156	0.96

**hsa-miR-191-5p**	1382.79	0.032	0.666	0.96
**hsa-miR-181d-5p**	13.10	0.085	0.579	0.96
**hsa-miR-1843**	25.29	-0.041	0.700	0.96
**hsa-miR-185-5p**	480.24	-0.144	0.167	0.96
**hsa-miR-186-5p**	293.01	-0.101	0.330	0.96
**hsa-miR-1908-5p**	39.87	-0.083	0.565	0.96
**hsa-miR-15b-3p**	19.26	-0.136	0.383	0.96
**hsa-miR-192-5p**	372.46	-0.085	0.611	0.96
**hsa-miR-181a-5p**	538.68	-0.056	0.496	0.96
**hsa-miR-194-5p**	157.00	0.068	0.638	0.96
**hsa-miR-197-3p**	49.58	-0.082	0.534	0.96
**hsa-miR-19b-3p**	37.48	-0.086	0.567	0.96
**hsa-miR-200c-3p**	30.86	0.597	0.019	0.96
**hsa-miR-17-5p**	122.06	-0.115	0.189	0.96
**hsa-miR-203a-3p**	212.98	0.636	0.020	0.96
**hsa-miR-206**	23.70	-0.225	0.397	0.96
**hsa-miR-16-5p**	518.60	-0.114	0.428	0.96
**hsa-miR-20a-5p**	194.05	-0.162	0.109	0.96
**hsa-miR-16-2-3p**	687.23	-0.126	0.326	0.96
**hsa-miR-20b-5p**	23.66	-0.199	0.217	0.96
**hsa-miR-21-5p**	6737.31	0.114	0.125	0.96
**hsa-miR-2110**	62.74	-0.135	0.159	0.96
**hsa-miR-183-5p**	130.69	-0.064	0.675	0.96
**hsa-miR-182-5p**	191.27	-0.074	0.596	0.96
**hsa-miR-409-3p**	439.35	-0.071	0.735	0.96
**hsa-miR-152-3p**	69.76	0.034	0.747	0.97
**hsa-miR-150-3p**	23.20	-0.061	0.746	0.97
**hsa-miR-130a-3p**	12.92	0.043	0.774	0.97
**hsa-miR-340-3p**	44.76	0.044	0.775	0.97
**hsa-miR-193a-5p**	89.26	-0.046	0.770	0.97
**hsa-miR-484**	384.77	-0.027	0.772	0.97
**hsa-miR-30a-3p**	28.21	0.035	0.771	0.97
**hsa-miR-181a-2-3p**	15.74	-0.030	0.791	0.98
**hsa-miR-7704**	19.37	-0.065	0.782	0.98
**hsa-miR-589-5p**	12.10	-0.040	0.789	0.98
**hsa-miR-199a-3p**	194.99	-0.024	0.804	0.98
**hsa-miR-146a-5p**	1248.55	0.021	0.810	0.98
**hsa-miR-338-5p**	20.65	-0.040	0.809	0.98
**hsa-miR-760**	20.68	0.033	0.813	0.98
**hsa-miR-664a-5p**	21.45	0.026	0.832	0.98
**hsa-miR-379-5p**	56.62	0.051	0.820	0.98
**hsa-miR-22-3p**	1734.89	-0.019	0.841	0.98
**hsa-miR-485-3p**	21.86	-0.057	0.827	0.98
**hsa-miR-30a-5p**	169.15	0.023	0.839	0.98
**hsa-miR-1246**	508.63	-0.033	0.834	0.98
**hsa-miR-7706**	20.11	0.029	0.862	0.99
**hsa-miR-128-3p**	1199.82	-0.012	0.863	0.99

**hsa-miR-151a-3p**	3782.82	0.016	0.861	0.99
**hsa-miR-1273h-3p**	17.24	-0.026	0.868	0.99
**hsa-miR-744-5p**	367.70	-0.005	0.962	0.99
**hsa-let-7d-3p**	624.70	0.007	0.944	0.99
**hsa-let-7i-5p**	11247.66	-0.009	0.899	0.99
**hsa-miR-1-3p**	465.67	-0.174	0.888	0.99
**hsa-miR-199a-5p**	218.23	0.018	0.900	0.99
**hsa-miR-134-5p**	89.07	0.017	0.934	0.99
**hsa-miR-493-5p**	20.59	-0.015	0.952	0.99
**hsa-miR-323a-3p**	14.81	0.029	0.905	0.99
**hsa-miR-320c**	150.00	0.017	0.881	0.99
**hsa-miR-543**	148.91	-0.025	0.896	0.99
**hsa-miR-27b-3p**	1024.36	0.004	0.953	0.99
**hsa-miR-381-3p**	50.61	-0.022	0.916	0.99
**hsa-miR-29a-3p**	114.08	0.009	0.943	0.99
**hsa-miR-181b-5p**	52.17	0.008	0.945	0.99
**hsa-miR-1180-3p**	94.29	-0.009	0.956	0.99
**hsa-miR-584-5p**	282.55	-0.009	0.925	0.99
**hsa-miR-328-3p**	275.39	0.005	0.960	0.99
**hsa-miR-30d-5p**	4600.44	0.003	0.947	0.99
**hsa-miR-424-3p**	46.42	0.011	0.924	0.99
**hsa-miR-19a-3p**	15.36	-0.005	0.977	1.00
**hsa-miR-23b-3p**	44.68	-0.005	0.970	1.00
**hsa-miR-143-3p**	541.09	-0.005	0.973	1.00
**hsa-miR-127-3p**	76.87	-0.001	0.998	1.00
**hsa-miR-374a-5p**	30.44	-0.002	0.990	1.00
**hsa-miR-576-3p**	16.32	-0.0002	0.998	1.00
**hsa-miR-15b-5p**	49.49	0.003	0.987	1.00

Supplementary Table S4. Differentially expressed miRNAs between 20 pre-diagnostic MPM cases and 20 matched controls from NGS. Table shows the average expression for all identified miRNAs (base Mean), the differential expression reported as log2 fold change between cases and controls (Log2FC), the p-value from statistical analysis (p-value) and the p-value adjusted for multiple testing by FDR (padj). miRNAs investigated for validation are denoted in bold.

**ID**	**baseMean**	**log2FoldChange**	**p-value**	**FDR[a]**
**hsa-miR-11400**	**33.60**	**1.35**	**0.00**	**0.01**
hsa-miR-361-3p	91.52	0.42	0.00	0.09
**hsa-miR-148a-3p**	**7287.60**	**0.61**	**0.00**	**0.09**
hsa-miR-671-3p	31.15	0.63	0.01	0.19
hsa-miR-139-3p	41.24	0.49	0.00	0.19
hsa-miR-151a-3p	3669.19	0.43	0.00	0.19
hsa-miR-107	93.16	-0.36	0.01	0.23
hsa-miR-584-5p	270.28	0.39	0.01	0.24
hsa-miR-744-5p	337.03	0.42	0.01	0.24
**hsa-miR-4508**	**194.01**	**-0.72**	**0.01**	**0.24**
hsa-miR-4446-3p	26.94	0.61	0.01	0.24
hsa-miR-152-3p	71.68	0.36	0.01	0.24
hsa-miR-363-3p	334.43	-0.32	0.02	0.29
hsa-miR-340-3p	43.00	0.53	0.03	0.30
hsa-miR-505-5p	21.16	-0.35	0.03	0.30
hsa-miR-576-3p	14.32	-0.46	0.02	0.30
hsa-miR-3613-5p	33.56	-0.51	0.03	0.30
hsa-miR-30e-3p	138.17	0.33	0.03	0.30
hsa-miR-144-3p	157.88	-0.40	0.03	0.30
hsa-miR-1908-5p	39.12	0.56	0.03	0.30
hsa-miR-142-5p	1268.35	-0.33	0.03	0.30
hsa-miR-16-2-3p	585.25	-0.35	0.02	0.30
hsa-miR-223-5p	446.51	0.36	0.03	0.30
hsa-miR-30d-5p	4309.93	0.14	0.03	0.30
hsa-miR-186-5p	249.03	-0.31	0.04	0.31
hsa-miR-150-5p	154.68	-0.46	0.04	0.31
hsa-miR-15b-5p	45.01	-0.48	0.04	0.31
hsa-miR-130b-5p	33.33	0.45	0.04	0.31
hsa-miR-23a-3p	69.97	0.36	0.04	0.31
**hsa-miR-409-3p**	**475.07**	**0.67**	**0.04**	**0.31**
hsa-miR-6842-3p	18.40	0.46	0.04	0.31
hsa-miR-128-3p	1147.03	0.21	0.05	0.31
hsa-miR-433-3p	23.27	0.70	0.05	0.31
hsa-miR-379-5p	68.61	0.71	0.05	0.31
hsa-miR-1843	24.13	0.37	0.06	0.32
hsa-miR-493-5p	25.62	0.76	0.05	0.32
hsa-miR-1180-3p	79.86	-0.40	0.06	0.32
hsa-miR-20a-5p	166.86	-0.27	0.06	0.34
hsa-miR-1273h-3p	17.25	0.46	0.06	0.34
hsa-miR-223-3p	94.66	0.29	0.07	0.35

ID	baseMean	log2FoldChange	p-value	FDR[a]
hsa-miR-222-3p	278.25	0.17	0.07	0.35
hsa-miR-122-5p	18547.91	0.60	0.07	0.36
hsa-miR-330-3p	59.75	0.34	0.08	0.39
hsa-miR-502-3p	12.62	-0.45	0.10	0.39
hsa-miR-148b-3p	324.52	0.23	0.08	0.39
hsa-miR-370-3p	88.88	0.53	0.09	0.39
hsa-miR-485-5p	37.96	0.57	0.08	0.39
hsa-miR-335-3p	21.84	0.42	0.10	0.39
hsa-miR-15a-5p	13.35	-0.51	0.09	0.39
hsa-miR-30e-5p	841.81	-0.21	0.09	0.39
hsa-miR-493-3p	28.98	0.66	0.09	0.39
hsa-miR-30c-5p	330.70	0.21	0.09	0.39
hsa-miR-221-3p	377.15	0.19	0.10	0.39
hsa-miR-106b-5p	35.12	-0.39	0.10	0.39
hsa-miR-144-5p	72.05	-0.34	0.11	0.40
hsa-miR-654-3p	29.29	0.51	0.10	0.40
hsa-miR-16-5p	456.04	-0.33	0.11	0.40
hsa-miR-323a-3p	15.49	0.69	0.11	0.40
hsa-miR-501-3p	71.36	-0.32	0.11	0.41
hsa-miR-4732-3p	33.33	-0.39	0.12	0.41
hsa-miR-382-5p	76.12	0.49	0.12	0.41
hsa-miR-423-3p	1681.47	0.17	0.12	0.41
hsa-let-7i-5p	10754.75	0.16	0.13	0.42
hsa-miR-328-3p	261.94	0.25	0.13	0.43
hsa-miR-432-5p	59.49	0.51	0.14	0.44
hsa-miR-98-5p	194.93	0.28	0.14	0.45
hsa-miR-194-5p	146.29	-0.28	0.16	0.45
hsa-miR-197-3p	41.56	0.25	0.16	0.45
hsa-miR-339-3p	21.04	-0.24	0.15	0.45
hsa-miR-2110	51.83	0.19	0.14	0.45
hsa-miR-24-3p	1626.25	0.14	0.15	0.45
hsa-miR-28-3p	232.08	0.18	0.16	0.45
hsa-miR-381-3p	52.41	0.48	0.16	0.45
hsa-miR-378a-3p	432.30	0.23	0.16	0.45
hsa-miR-323b-3p	34.39	0.51	0.16	0.45
hsa-miR-23b-3p	39.88	0.26	0.17	0.47
hsa-miR-126-3p	7402.44	0.17	0.17	0.48
hsa-miR-26b-5p	588.51	-0.17	0.18	0.49
hsa-miR-20b-5p	20.25	-0.31	0.18	0.49
hsa-miR-375-3p	156.71	0.40	0.20	0.52
hsa-miR-628-3p	17.67	0.27	0.21	0.53
hsa-miR-1301-3p	64.02	0.25	0.21	0.53
hsa-miR-4488	18.00	-0.40	0.21	0.53
hsa-miR-224-5p	80.78	0.34	0.21	0.53
hsa-miR-22-5p	73.85	0.26	0.22	0.53
hsa-miR-425-3p	13.54	0.23	0.22	0.53

ID	baseMean	log2FoldChange	p-value	FDR[a]
hsa-miR-654-5p	15.71	0.46	0.22	0.54
hsa-miR-93-5p	156.44	-0.17	0.23	0.55
hsa-miR-486-5p	42289.42	-0.23	0.26	0.57
hsa-miR-7704	17.85	-0.36	0.24	0.57
hsa-let-7f-5p	5440.84	0.16	0.25	0.57
hsa-miR-664a-5p	20.36	0.20	0.26	0.57
hsa-miR-101-3p	1199.58	-0.21	0.25	0.57
hsa-miR-106b-3p	171.05	0.13	0.25	0.57
hsa-miR-451a	38231.09	-0.27	0.26	0.57
hsa-miR-10a-5p	1117.19	0.21	0.26	0.57
hsa-miR-127-3p	100.09	0.44	0.24	0.57
hsa-miR-134-5p	95.53	0.36	0.25	0.57
hsa-let-7a-3p	43.29	-0.15	0.28	0.58
hsa-miR-340-5p	112.61	0.15	0.27	0.58
hsa-miR-199a-3p	192.25	0.18	0.27	0.58
hsa-miR-155-5p	51.69	-0.19	0.28	0.58
hsa-miR-625-3p	138.96	0.25	0.29	0.58
hsa-miR-125a-5p	335.82	-0.16	0.29	0.58
hsa-miR-206	27.07	0.42	0.29	0.58
hsa-miR-543	155.62	0.29	0.33	0.60
hsa-let-7a-5p	4633.63	0.10	0.32	0.60
hsa-miR-532-5p	256.93	-0.13	0.33	0.60
hsa-let-7e-5p	113.00	0.21	0.32	0.60
hsa-miR-130a-3p	11.11	0.28	0.32	0.60
hsa-miR-342-3p	25.91	-0.22	0.30	0.60
hsa-miR-126-5p	126.19	-0.15	0.31	0.60
hsa-miR-199a-5p	208.53	0.21	0.32	0.60
hsa-miR-143-3p	498.08	0.17	0.31	0.60
hsa-miR-495-3p	20.19	0.35	0.33	0.61
hsa-miR-19b-3p	33.81	-0.25	0.34	0.61
hsa-miR-4732-5p	39.45	-0.23	0.34	0.61
hsa-miR-589-5p	11.80	0.26	0.34	0.61
hsa-miR-21-5p	6734.84	0.11	0.34	0.61
hsa-miR-193a-5p	84.19	0.22	0.36	0.63
hsa-miR-26a-5p	9981.66	0.14	0.36	0.63
hsa-miR-19a-3p	16.17	-0.25	0.37	0.63
hsa-miR-145-3p	29.16	0.23	0.37	0.63
hsa-miR-942-5p	15.09	-0.20	0.37	0.63
hsa-miR-146a-5p	1173.34	0.13	0.37	0.63
hsa-miR-4497	30.47	-0.25	0.39	0.64
hsa-miR-425-5p	234.96	-0.10	0.39	0.64
hsa-miR-511-5p	22.47	0.28	0.39	0.64
hsa-miR-99a-5p	1691.55	0.20	0.39	0.64
hsa-miR-99b-5p	397.93	0.12	0.39	0.64
hsa-miR-92b-3p	86.43	-0.12	0.40	0.64
hsa-miR-424-3p	42.76	-0.14	0.40	0.64

ID	baseMean	log2FoldChange	p-value	FDR[a]
hsa-miR-652-3p	24.28	-0.17	0.41	0.65
hsa-miR-185-5p	392.59	-0.13	0.42	0.66
hsa-miR-27b-3p	1012.14	0.11	0.43	0.67
hsa-miR-151a-5p	21.75	0.23	0.43	0.67
hsa-miR-485-3p	23.56	0.28	0.44	0.68
hsa-miR-4433b-3p	55.55	0.35	0.46	0.71
hsa-miR-339-5p	26.41	0.16	0.47	0.71
hsa-miR-629-5p	162.93	-0.12	0.47	0.71
hsa-miR-181a-2-3p	16.21	0.13	0.51	0.71
hsa-miR-181a-5p	516.08	-0.07	0.52	0.71
hsa-miR-139-5p	188.41	0.10	0.48	0.71
hsa-miR-1307-3p	432.02	0.09	0.48	0.71
hsa-miR-22-3p	1624.64	-0.10	0.50	0.71
hsa-miR-1228-5p	30.99	0.23	0.51	0.71
hsa-miR-142-3p	143.38	-0.10	0.50	0.71
hsa-miR-140-3p	571.04	0.08	0.52	0.71
hsa-miR-484	328.63	-0.08	0.52	0.71
hsa-miR-941	67.25	0.10	0.52	0.71
hsa-miR-100-5p	251.30	-0.20	0.49	0.71
hsa-miR-342-5p	99.90	-0.13	0.48	0.71
hsa-miR-361-5p	17.05	0.13	0.52	0.71
hsa-let-7g-5p	2365.49	-0.06	0.52	0.71
hsa-miR-29a-3p	115.96	-0.14	0.49	0.71
hsa-miR-3158-3p	28.10	0.18	0.50	0.71
hsa-miR-760	19.52	0.11	0.53	0.71
hsa-miR-378c	77.71	-0.11	0.53	0.71
hsa-miR-320b	1009.68	-0.09	0.55	0.73
hsa-miR-28-5p	28.77	-0.13	0.56	0.74
hsa-miR-483-5p	49.10	0.17	0.59	0.78
hsa-miR-92a-3p	11188.77	-0.07	0.62	0.80
hsa-miR-182-5p	171.03	0.10	0.63	0.82
hsa-miR-1246	467.43	0.10	0.65	0.83
hsa-miR-25-3p	3017.87	-0.07	0.65	0.83
hsa-miR-103a-3p	326.05	0.05	0.65	0.83
hsa-let-7c-5p	661.96	-0.05	0.66	0.84
hsa-miR-140-5p	41.62	-0.07	0.67	0.84
hsa-miR-1-3p	241.39	-0.13	0.68	0.84
hsa-miR-215-5p	47.90	-0.09	0.68	0.84
hsa-miR-345-5p	16.36	0.09	0.69	0.85
hsa-miR-27a-5p	12.17	0.11	0.69	0.85
hsa-miR-181d-5p	12.38	-0.09	0.72	0.86
hsa-miR-191-5p	1250.05	0.04	0.71	0.86
hsa-let-7b-5p	8648.82	0.06	0.71	0.86
hsa-miR-4516	28.86	-0.11	0.72	0.86
hsa-miR-15b-3p	16.33	-0.08	0.72	0.86
hsa-miR-203a-3p	344.32	-0.12	0.79	0.93

ID	baseMean	log2FoldChange	p-value	FDR[a]
hsa-miR-4443	106.52	0.07	0.79	0.93
hsa-miR-30a-3p	28.86	-0.05	0.81	0.95
hsa-miR-181b-5p	55.57	-0.04	0.83	0.97
hsa-miR-192-5p	295.15	-0.03	0.83	0.97
hsa-miR-200c-3p	17.35	-0.04	0.84	0.97
hsa-miR-320d	57.11	0.05	0.84	0.97
hsa-miR-4433b-5p	83.24	0.05	0.85	0.97
hsa-miR-550a-5p	10.24	-0.05	0.86	0.97
hsa-miR-24-2-5p	20.39	0.03	0.86	0.97
hsa-miR-3615	517.34	-0.03	0.87	0.98
hsa-let-7d-5p	649.43	0.01	0.92	0.99
hsa-let-7d-3p	598.68	0.01	0.92	0.99
hsa-let-7b-3p	12.73	-0.03	0.92	0.99
hsa-miR-32-5p	25.52	-0.01	0.94	0.99
hsa-miR-7-5p	229.50	0.01	0.94	0.99
hsa-miR-320a-3p	3948.70	-0.01	0.92	0.99
hsa-miR-320c	153.77	-0.01	0.97	0.99
hsa-miR-7706	17.62	0.02	0.95	0.99
hsa-miR-30b-5p	16.47	0.02	0.92	0.99
hsa-miR-335-5p	19.16	0.01	0.96	0.99
hsa-miR-374b-5p	13.63	0.01	0.98	0.99
hsa-miR-30a-5p	172.51	-0.01	0.98	0.99
hsa-miR-10b-5p	1964.83	0.01	0.97	0.99
hsa-miR-338-5p	19.03	0.01	0.97	0.99
hsa-miR-423-5p	12999.68	0.01	0.97	0.99
hsa-miR-125b-5p	112.46	-0.01	0.96	0.99
hsa-miR-150-3p	21.82	-0.03	0.92	0.99
hsa-miR-17-5p	107.51	-0.01	0.97	0.99
hsa-miR-183-5p	112.04	0.03	0.90	0.99
hsa-miR-486-3p	126.47	-0.02	0.91	0.99
hsa-miR-374a-5p	31.06	0.02	0.95	0.99
hsa-miR-27a-3p	988.26	0.00	0.99	0.99
hsa-miR-146b-5p	650.36	0.00	1.00	1.00

[a]adjustment for multiple testing by false discovery rate

Supplementary Table S5. Validated miRNA-target interaction results from MultiMiR Bioconductor's package.

mature_mirna_acc	mature_mirna_id	target_symbol	target_entrez	target_ensembl	mirecords	mirtarbase	tarbase	validated.sum
MIMAT0000243	hsa-miR-148a-3p	DNMT1	1786	ENSG00000130816	2	7	1	3
MIMAT0000243	hsa-miR-148a-3p	ACVR1	90	ENSG00000115170	0	2	1	2
MIMAT0000243	hsa-miR-148a-3p	AGO2	27161	ENSG00000123908	0	2	1	2
MIMAT0000243	hsa-miR-148a-3p	ALCAM	214	ENSG00000170017	0	1	1	2
MIMAT0000243	hsa-miR-148a-3p	ANP32A	8125	ENSG00000140350	0	1	1	2
MIMAT0000243	hsa-miR-148a-3p	APC	324	ENSG00000134982	0	1	1	2
MIMAT0000243	hsa-miR-148a-3p	APLP2	334	ENSG00000084234	0	1	1	2
MIMAT0000243	hsa-miR-148a-3p	APPBP2	10513	ENSG00000062725	0	1	1	2
MIMAT0000243	hsa-miR-148a-3p	ARID3A	1820	ENSG00000116017	0	1	1	2
MIMAT0000243	hsa-miR-148a-3p	ARL6IP1	23204	ENSG00000170540	0	1	1	2
MIMAT0000243	hsa-miR-148a-3p	ARL8B	55207	ENSG00000134108	0	1	1	2
MIMAT0000243	hsa-miR-148a-3p	ARRDC3	57561	ENSG00000113369	0	1	1	2
MIMAT0000243	hsa-miR-148a-3p	AURKB	9212	ENSG00000178999	0	1	1	2
MIMAT0000243	hsa-miR-148a-3p	BAZ2B	29994	ENSG00000123636	0	1	1	2
MIMAT0000243	hsa-miR-148a-3p	BCL2L11	10018	ENSG00000153094	0	1	1	2
MIMAT0000243	hsa-miR-148a-3p	BTBD3	22903	ENSG00000132640	0	7	1	2
MIMAT0000243	hsa-miR-148a-3p	CBX3	11335	ENSG00000122565	0	1	1	2
MIMAT0000243	hsa-miR-148a-3p	CCKBR	887	ENSG00000110148	0	4	1	2
MIMAT0000243	hsa-miR-148a-3p	CCNA2	890	ENSG00000145386	0	3	1	2
MIMAT0000243	hsa-miR-148a-3p	CCNI	10983	ENSG00000118816	0	1	1	2
MIMAT0000243	hsa-miR-148a-3p	CCT6A	908	ENSG00000146731	0	1	1	2
MIMAT0000243	hsa-miR-148a-3p	CDC25B	994	ENSG00000101224	0	1	1	2
MIMAT0000243	hsa-miR-148a-3p	CDK19	23097	ENSG00000155111	0	2	1	2
MIMAT0000243	hsa-miR-148a-3p	CDKN1A	1026	ENSG00000124762	0	1	1	2
MIMAT0000243	hsa-miR-148a-3p	CDKN1B	1027	ENSG00000111276	0	4	1	2
MIMAT0000243	hsa-miR-148a-3p	CEBPG	1054	ENSG00000153879	0	1	1	2
MIMAT0000243	hsa-miR-148a-3p	CNOT4	4850	ENSG00000080802	0	4	1	2
MIMAT0000243	hsa-miR-148a-3p	DDX6	1656	ENSG00000110367	0	2	1	2
MIMAT0000243	hsa-miR-148a-3p	DICER1	23405	ENSG00000100697	0	2	1	2

MIMAT0000243	hsa-miR-148a-3p	DNMT3B	1789	ENSG00000088305	4	2	0	2
MIMAT0000243	hsa-miR-148a-3p	DSTYK	25778	ENSG00000133059	0	1	1	2
MIMAT0000243	hsa-miR-148a-3p	DYNLL2	140735	ENSG00000264364	0	1	1	2
MIMAT0000243	hsa-miR-148a-3p	DYRK1A	1859	ENSG00000157540	0	1	1	2
MIMAT0000243	hsa-miR-148a-3p	EOGT	285203	ENSG00000163378	0	3	1	2
MIMAT0000243	hsa-miR-148a-3p	ERRFI1	54206	ENSG00000116285	0	1	1	2
MIMAT0000243	hsa-miR-148a-3p	FAM104A	84923	ENSG00000133193	0	1	1	2
MIMAT0000243	hsa-miR-148a-3p	FURIN	5045	ENSG00000140564	0	1	1	2
MIMAT0000243	hsa-miR-148a-3p	FXR1	8087	ENSG00000114416	0	1	1	2
MIMAT0000243	hsa-miR-148a-3p	GAS1	2619	ENSG00000180447	0	3	1	2
MIMAT0000243	hsa-miR-148a-3p	GLRX5	51218	ENSG00000182512	0	1	1	2
MIMAT0000243	hsa-miR-148a-3p	GPRC5A	9052	ENSG00000013588	0	2	1	2
MIMAT0000243	hsa-miR-148a-3p	HCCS	3052	ENSG00000004961	0	2	1	2
MIMAT0000243	hsa-miR-148a-3p	HLA-A	3105	ENSG00000206503	0	2	1	2
MIMAT0000243	hsa-miR-148a-3p	HLA-C	3107	ENSG00000204525	0	2	1	2
MIMAT0000243	hsa-miR-148a-3p	HMGB1	3146	ENSG00000189403	0	1	1	2
MIMAT0000243	hsa-miR-148a-3p	HSP90AA1	3320	ENSG00000080824	0	1	1	2
MIMAT0000243	hsa-miR-148a-3p	HSP90B1	7184	ENSG00000166598	0	2	1	2
MIMAT0000243	hsa-miR-148a-3p	HSPA4	3308	ENSG00000170606	0	1	1	2
MIMAT0000243	hsa-miR-148a-3p	INO80	54617	ENSG00000128908	0	1	1	2
MIMAT0000243	hsa-miR-148a-3p	IRS1	3667	ENSG00000169047	0	1	1	2
MIMAT0000243	hsa-miR-148a-3p	ITGA5	3678	ENSG00000161638	0	1	1	2
MIMAT0000243	hsa-miR-148a-3p	ITGB8	3696	ENSG00000105855	0	2	1	2
MIMAT0000243	hsa-miR-148a-3p	JARID2	3720	ENSG00000008083	0	1	1	2
MIMAT0000243	hsa-miR-148a-3p	KANSL1	284058	ENSG00000120071	0	1	1	2
MIMAT0000243	hsa-miR-148a-3p	KIF2C	11004	ENSG00000142945	0	1	1	2
MIMAT0000243	hsa-miR-148a-3p	LBR	3930	ENSG00000143815	0	1	1	2
MIMAT0000243	hsa-miR-148a-3p	LDLR	3949	ENSG00000130164	0	1	1	2
MIMAT0000243	hsa-miR-148a-3p	LNPEP	4012	ENSG00000113441	0	2	1	2
MIMAT0000243	hsa-miR-148a-3p	MAP3K9	4293	ENSG00000006432	0	2	1	2
MIMAT0000243	hsa-miR-148a-3p	MRPS27	23107	ENSG00000113048	0	1	1	2
MIMAT0000243	hsa-miR-148a-3p	MYC	4609	ENSG00000136997	0	1	1	2

MIMAT0000243	hsa-miR-148a-3p	NPTX1	4884	ENSG00000171246	0	1	1	2
MIMAT0000243	hsa-miR-148a-3p	NR1I2	8856	ENSG00000144852	1	1	0	2
MIMAT0000243	hsa-miR-148a-3p	NRP1	8829	ENSG00000099250	0	1	1	2
MIMAT0000243	hsa-miR-148a-3p	OBI1	79596	ENSG00000152193	0	1	1	2
MIMAT0000243	hsa-miR-148a-3p	OTUD4	54726	ENSG00000164164	0	1	1	2
MIMAT0000243	hsa-miR-148a-3p	PATL1	219988	ENSG00000166889	0	1	1	2
MIMAT0000243	hsa-miR-148a-3p	PBXIP1	57326	ENSG00000163346	0	2	1	2
MIMAT0000243	hsa-miR-148a-3p	PDIA3	2923	ENSG00000167004	0	1	1	2
MIMAT0000243	hsa-miR-148a-3p	PGAP4	84302	ENSG00000165152	0	1	1	2
MIMAT0000243	hsa-miR-148a-3p	PPARD	5467	ENSG00000112033	0	1	1	2
MIMAT0000243	hsa-miR-148a-3p	PPP6R1	22870	ENSG00000105063	0	2	1	2
MIMAT0000243	hsa-miR-148a-3p	PRNP	5621	ENSG00000171867	0	1	1	2
MIMAT0000243	hsa-miR-148a-3p	PTPN23	25930	ENSG00000076201	0	1	1	2
MIMAT0000243	hsa-miR-148a-3p	QKI	9444	ENSG00000112531	0	1	1	2
MIMAT0000243	hsa-miR-148a-3p	RAB10	10890	ENSG00000084733	0	1	1	2
MIMAT0000243	hsa-miR-148a-3p	RAB14	51552	ENSG00000119396	0	1	1	2
MIMAT0000243	hsa-miR-148a-3p	RAB1B	81876	ENSG00000174903	0	2	1	2
MIMAT0000243	hsa-miR-148a-3p	RAB34	83871	ENSG00000109113	0	2	1	2
MIMAT0000243	hsa-miR-148a-3p	RALY	22913	ENSG00000125970	0	1	1	2
MIMAT0000243	hsa-miR-148a-3p	RASSF8	11228	ENSG00000123094	0	1	1	2
MIMAT0000243	hsa-miR-148a-3p	RBM23	55147	ENSG00000100461	0	1	1	2
MIMAT0000243	hsa-miR-148a-3p	RBM38	55544	ENSG00000132819	0	1	1	2
MIMAT0000243	hsa-miR-148a-3p	RCC2	55920	ENSG00000179051	0	1	1	2
MIMAT0000243	hsa-miR-148a-3p	ROCK1	6093	ENSG00000067900	0	2	1	2
MIMAT0000243	hsa-miR-148a-3p	S1PR1	1901	ENSG00000170989	0	3	1	2
MIMAT0000243	hsa-miR-148a-3p	SECISBP2L	9728	ENSG00000138593	0	5	1	2
MIMAT0000243	hsa-miR-148a-3p	SERPINE1	5054	ENSG00000106366	0	1	1	2
MIMAT0000243	hsa-miR-148a-3p	SESN3	143686	ENSG00000149212	0	1	1	2
MIMAT0000243	hsa-miR-148a-3p	SESTD1	91404	ENSG00000187231	0	1	1	2
MIMAT0000243	hsa-miR-148a-3p	SIK1	150094	ENSG00000142178	0	3	1	2
MIMAT0000243	hsa-miR-148a-3p	SLC25A3	5250	ENSG00000075415	0	1	1	2
MIMAT0000243	hsa-miR-148a-3p	SLC38A2	54407	ENSG00000134294	0	2	1	2

MIMAT0000243	hsa-miR-148a-3p	SMAD2	4087	ENSG00000175387	0	4	1	2
MIMAT0000243	hsa-miR-148a-3p	SPRY2	10253	ENSG00000136158	0	1	1	2
MIMAT0000243	hsa-miR-148a-3p	STX6	10228	ENSG00000135823	0	1	1	2
MIMAT0000243	hsa-miR-148a-3p	TGIF2	60436	ENSG00000118707	0	1	1	2
MIMAT0000243	hsa-miR-148a-3p	TMED7	51014	ENSG00000134970	0	1	1	2
MIMAT0000243	hsa-miR-148a-3p	TMEM9B	56674	ENSG00000175348	0	1	1	2
MIMAT0000243	hsa-miR-148a-3p	TNRC6A	27327	ENSG00000090905	0	2	1	2
MIMAT0000243	hsa-miR-148a-3p	TNRC6B	23112	ENSG00000100354	0	1	1	2
MIMAT0000243	hsa-miR-148a-3p	TRIM59	286827	ENSG00000213186	0	1	1	2
MIMAT0000243	hsa-miR-148a-3p	TXNIP	10628	ENSG00000265972	0	2	1	2
MIMAT0000243	hsa-miR-148a-3p	UBE2D3	7323	ENSG00000109332	0	1	1	2
MIMAT0000243	hsa-miR-148a-3p	UQCRQ	27089	ENSG00000164405	0	1	1	2
MIMAT0000243	hsa-miR-148a-3p	VAV2	7410	ENSG00000160293	0	1	1	2
MIMAT0000243	hsa-miR-148a-3p	VPS37A	137492	ENSG00000155975	0	1	1	2
MIMAT0000243	hsa-miR-148a-3p	VPS37B	79720	ENSG00000139722	0	1	1	2
MIMAT0000243	hsa-miR-148a-3p	YWHAB	7529	ENSG00000166913	0	1	1	2
MIMAT0000243	hsa-miR-148a-3p	ZFYVE26	23503	ENSG00000072121	0	2	1	2
MIMAT0001639	hsa-miR-409-3p	FGB	2244	ENSG00000171564	0	1	1	2

Supplementary Table S6. KEGG pathway enrichment analysis for validated target genes of miR-409-3p and miR-148a.

nGenes[a]	Enrichment FDR [b]	Pathway Genes	Fold Enrichment [c]	Pathway	Genes
13	1.81E-10	161	16.6	MicroRNAs in cancer	BCL2L11 SPRY2 CDKN1A CDKN1B DNMT1 DNMT3B DICER1 APC IRS1 ITGA5 MYC ROCK1 CDC25B
7	0.000207161	124	11.6	Cell cycle	CDKN1A CDKN1B SMAD2 MYC HEL-S-1 CCNA2 CDC25B
8	0.00036598	202	8.1	Epstein-Barr virus infection	BCL2L11 CDKN1A CDKN1B HEL-S-269 HLA-A HLA-Cw MYC CCNA2
10	0.000377112	354	5.8	PI3K-Akt signaling pathway	BCL2L11 CDKN1A CDKN1B HSP90AA1 IRS1 ITGA5 ITGB8 MYC HEL-S-125m HEL-S-1
7	0.000377112	156	9.2	Cellular senescence	CDKN1A HLA-A HLA-Cw SMAD2 MYC SERPINE1 CCNA2
5	0.001087109	78	13.2	Antigen processing and presentation	HEL-S-269 HLA-A HLA-Cw HSPA4 HSP90AA1
11	0.001304125	530	4.3	Pathways in cancer	BCL2L11 CDKN1A CDKN1B APC HSP90AA1 SMAD2 MYC NR1C2 ROCK1 HEL-S-125m CCNA2
5	0.001304125	86	11.9	Colorectal cancer	BCL2L11 CDKN1A APC SMAD2 MYC
5	0.00164067	93	11.0	TGF-beta signaling pathway	SMAD2 MYC TGIF2 ROCK1 ACVR1
7	0.00164067	219	6.6	Human T-cell leukemia virus 1 infection	CDKN1A HLA-A HLA-Cw SMAD2 MYC NRP1 CCNA2
7	0.003244572	252	5.7	Endocytosis	RAB10 VPS37A HLA-A HLA-Cw LDLR SMAD2 VPS37B
8	0.003244572	331	5.0	Human papillomavirus infection	CDKN1A CDKN1B HLA-A HLA-Cw APC ITGA5 ITGB8 CCNA2
5	0.005278394	131	7.8	FoxO signaling pathway	BCL2L11 CDKN1A CDKN1B S1PR1 IRS1
6	0.005278394	203	6.1	Viral carcinogenesis	CDKN1A CDKN1B HLA-A HLA-Cw HEL-S-1 CCNA2
6	0.005278394	202	6.1	Proteoglycans in cancer	CDKN1A ITGA5 SMAD2 MYC ROCK1 VAV2

nGenes[a]	Enrichment FDR [b]	Pathway Genes	Fold Enrichment [c]	Pathway	Genes
5	0.006980585	143	7.2	Signaling pathways regulating pluripotency of stem cells	APC JARID2 SMAD2 MYC ACVR1
6	0.007365432	224	5.5	Human cytomegalovirus infection	CDKN1A HEL-S-269 HLA-A HLA-Cw MYC ROCK1
5	0.007365432	148	6.9	Gastric cancer	CDKN1A CDKN1B APC SMAD2 MYC
5	0.008943497	157	6.5	Hippo signaling pathway	APC SMAD2 MYC SERPINE1 HEL-S-1
4	0.010681789	97	8.5	Prostate cancer	CDKN1A CDKN1B HSP90AA1 HEL-S-125m
4	0.021272746	120	6.8	AMPK signaling pathway	RAB10 IRS1 RAB14 CCNA2
3	0.021272746	58	10.6	Endometrial cancer	CDKN1A APC MYC
5	0.028158182	217	4.7	Regulation of actin cytoskeleton	APC ITGA5 ITGB8 ROCK1 VAV2
3	0.028158182	67	9.2	Acute myeloid leukemia	MYC NR1C2 CCNA2
5	0.028158182	214	4.8	Lipid and atherosclerosis	HSPA4 HSP90AA1 LDLR HEL-S-125m VAV2
3	0.034419624	73	8.4	P53 signaling pathway	CDKN1A SESN3 SERPINE1
4	0.035815824	149	5.5	Cell adhesion molecules	ALCAM HLA-A HLA-Cw ITGB8
3	0.035815824	76	8.1	Chronic myeloid leukemia	CDKN1A CDKN1B MYC
3	0.038350195	79	7.8	RNA degradation	DDX6 PATL1 CNOT4
4	0.038350195	155	5.3	Cushing syndrome	CDKN1A CDKN1B APC LDLR
5	0.038409225	249	4.1	Salmonella infection	DYNLL2 HSP90AA1 MYC ARL8B HEL-S-125m
4	0.038409225	157	5.2	Hepatitis C	CDKN1A LDLR MYC HEL-S-1
3	0.038799015	84	7.3	ErbB signaling pathway	CDKN1A CDKN1B MYC
4	0.038799015	161	5.1	Hepatitis B	CDKN1A MYC HEL-S-1 CCNA2
4	0.04249957	167	4.9	Hepatocellular carcinoma	CDKN1A APC SMAD2 MYC
4	0.043034962	169	4.9	Protein processing in endoplasmic reticulum	HEL-S-269 HSP90AA1 HEL-S-125m UBE2D3
3	0.045693219	92	6.7	Small cell lung cancer	CDKN1A CDKN1B MYC

[a] number of genes enriched in the pathwa[y]

[b] FDR is adjusted from the hypergeometric test. Fold Enrichment indicates how drastically genes of a certain pathway is overrepresented.

nGenes[a]	Enrichment FDR [b]	Pathway Genes	Fold Enrichment [c]	Pathway	Genes

[c] Fold Enrichment is defined as the percentage of genes in the list belonging to a pathway, divided by the corresponding percentage in the background

Supplementary Table S7. miRWalk 2.0 consensus target predictions for miR-11400.

mirnaid	refseqid	genesymbol	start	end	bindingp	seed	phylopstem	phylopflank	binding_region_length	longest_consecutive_pairings	position
hsa-miR-11400	NM_012154	AGO2	9415	9431	1	1	0.62	0.09	16	15	3UTR
hsa-miR-11400	NM_001164623	AGO2	9313	9329	1	1	0.49	0.06	16	15	3UTR
hsa-miR-11400	NM_177422	AGO3	7056	7075	1	1	0.41	0.40	19	10	3UTR
hsa-miR-11400	NM_024852	AGO3	7314	7333	1	1	0.41	0.40	19	10	3UTR
hsa-miR-11400	NM_017629	AGO4	3705	3723	1	1	1.98	1.78	18	9	3UTR
hsa-miR-11400	NM_001013630	AADACL4	1768	1807	0.974359	1	-0.79	-0.85	39	8	3UTR
hsa-miR-11400	NM_001271885	AAGAB	3061	3080	1	1	0.00	0.00	19	10	3UTR
hsa-miR-11400	NM_001271886	AAGAB	3247	3266	1	1	0.10	1.21	19	10	3UTR
hsa-miR-11400	NM_014911	AAK1	8625	8646	1	1	1.24	0.86	21	10	3UTR
hsa-miR-11400	NM_001371575	AAK1	8507	8528	1	1	1.10	1.67	21	10	3UTR
hsa-miR-11400	NM_020686	ABAT	2444	2465	1	1	-0.44	-0.20	21	8	3UTR
hsa-miR-11400	NM_000663	ABAT	3246	3267	1	1	-0.44	-0.20	21	8	3UTR
hsa-miR-11400	NM_001127448	ABAT	2571	2592	1	1	-0.44	-0.20	21	8	3UTR
hsa-miR-11400	NM_033450	ABCC10	4808	4827	1	1	-0.20	-0.24	19	18	3UTR

mirnaid	refseqid	genesymbol	start	end	bindingp	seed	phylopstem	phylopflank	binding_region_length	longest_consecutive_pairings	position
hsa-miR-11400	NM_001198934	ABCC10	4761	4780	1	1	-0.20	-0.24	19	18	3UTR
hsa-miR-11400	NM_001301829	ABCC4	4192	4207	1	1	4.11	3.67	15	14	3UTR
hsa-miR-11400	NM_005845	ABCC4	4333	4348	1	1	4.11	3.67	15	14	3UTR
hsa-miR-11400	NM_001079528	ABCC6	400	425	1	1	-0.04	0.29	25	8	3UTR
hsa-miR-11400	NM_005164	ABCD2	2495	2517	1	1	0.43	0.18	22	9	3UTR
hsa-miR-11400	NM_005164	ABCD2	3184	3200	1	1	0.16	0.47	16	10	3UTR
hsa-miR-11400	NM_022169	ABCG4	3745	3764	1	1	1.22	1.66	19	18	3UTR
hsa-miR-11400	NM_001142505	ABCG4	3533	3552	1	1	1.22	1.66	19	18	3UTR
hsa-miR-11400	NM_022437	ABCG8	6905	6928	1	1	0.44	-0.08	21	8	3UTR
hsa-miR-11400	NM_016006	ABHD5	4800	4823	1	1	0.09	-0.12	23	10	3UTR
hsa-miR-11400	NM_001355186	ABHD5	1328	1350	1	1	-0.49	-0.08	22	7	3UTR
hsa-miR-11400	NM_001365649	ABHD5	5110	5133	1	1	0.09	-0.12	23	10	3UTR
hsa-miR-11400	NM_001365650	ABHD5	4681	4704	1	1	0.09	-0.12	23	10	3UTR
hsa-miR-11400	NM_001135186	ABI3	1411	1429	0.961538	1	0.00	0.00	18	10	3UTR
hsa-miR-11400	NM_005158	ABL2	7147	7175	1	1	-0.77	0.38	19	14	3UTR
hsa-miR-11400	NM_007314	ABL2	7440	7468	1	1	0.51	0.17	19	14	3UTR
hsa-miR-11400	NM_001136000	ABL2	6838	6866	1	1	0.93	0.16	19	14	3UTR

mirnaid	refseqid	genesymbol	start	end	bindingp	seed	phylopstem	phylopflank	binding_region_length	longest_consecutive_pairings	position
hsa-miR-11400	NM_001168236	ABL2	7377	7405	1	1	-0.06	0.12	19	14	3UTR
hsa-miR-11400	NM_001168237	ABL2	7131	7159	1	1	0.73	0.33	19	14	3UTR
hsa-miR-11400	NM_001168238	ABL2	7068	7096	1	1	0.43	0.74	19	14	3UTR
hsa-miR-11400	NM_001168239	ABL2	6775	6803	1	1	0.67	0.20	19	14	3UTR
hsa-miR-11400	NM_020469	ABO	2257	2276	1	1	0.00	0.04	19	7	3UTR
hsa-miR-11400	XM_005267418	ACIN1	2432	2454	1	1	0.00	0.00	22	11	3UTR
hsa-miR-11400	NM_014977	ACIN1	4410	4431	1	1	3.47	2.74	21	8	3UTR
hsa-miR-11400	NM_001164814	ACIN1	4371	4392	1	1	3.47	2.74	21	8	3UTR
hsa-miR-11400	NM_001164815	ACIN1	4290	4311	1	1	3.47	2.74	21	8	3UTR
hsa-miR-11400	NM_001302490	ACP2	1586	1613	1	1	3.42	2.87	20	8	3UTR
hsa-miR-11400	NM_001111035	ACP5	1389	1406	1	1	-0.89	-0.08	17	10	3UTR
hsa-miR-11400	NM_016361	ACP6	2159	2179	1	1	0.12	0.08	20	8	3UTR
hsa-miR-11400	NM_015162	ACSBG1	2874	2927	1	1	-0.23	-0.10	23	9	3UTR
hsa-miR-11400	XM_017022923	ACSM2A	2802	2825	1	1	0.00	0.00	20	12	3UTR
hsa-miR-11400	NM_001105069	ACSM2B	2773	2796	1	1	0.30	0.04	20	12	3UTR
hsa-miR-11400	NM_001199954	ACTG1	1322	1342	0.974359	1	0.00	0.00	20	11	3UTR
hsa-miR-11400	NM_005735	ACTR1B	1862	1882	1	1	5.52	5.34	20	12	3UTR

mirnaid	refseqid	genesymbol	start	end	bindingp	seed	phylopstem	phylopflank	binding_region_length	longest_consecutive_pairings	position
hsa-miR-11400	NM_001282227	ADA2	3296	3318	1	1	1.90	1.71	22	7	3UTR
hsa-miR-11400	NM_001190956	ADAM18	924	950	1	1	0.31	-0.09	12	10	3UTR
hsa-miR-11400	NM_016351	ADAM22	4169	4219	1	1	0.01	0.13	42	11	3UTR
hsa-miR-11400	NM_021722	ADAM22	4256	4306	1	1	0.01	0.13	42	11	3UTR
hsa-miR-11400	NM_021723	ADAM22	4277	4327	1	1	0.01	0.13	42	11	3UTR
hsa-miR-11400	NM_145004	ADAM32	2476	2491	1	1	0.42	0.45	15	12	3UTR
hsa-miR-11400	NM_030955	ADAMTS12	5575	5594	1	1	2.88	3.27	19	9	3UTR
hsa-miR-11400	NM_001324512	ADAMTS12	5320	5339	1	1	0.27	0.13	19	9	3UTR
hsa-miR-11400	NM_014243	ADAMTS3	5495	5528	1	1	3.81	3.26	33	9	3UTR
hsa-miR-11400	NM_182920	ADAMTS9	7084	7106	1	1	3.17	2.94	22	16	3UTR
hsa-miR-11400	NM_001318781	ADAMTS9	7000	7022	1	1	3.17	2.94	22	16	3UTR
hsa-miR-11400	XM_011521825	ADAMTSL3	4470	4491	0.961538	1	0.00	0.00	21	12	3UTR
hsa-miR-11400	NM_001281768	ADCY1	1681	1701	1	1	0.00	0.00	20	9	3UTR
hsa-miR-11400	NM_001118	ADCYAP1R1	1891	1913	1	1	0.76	0.94	22	11	3UTR
hsa-miR-11400	NM_001199635	ADCYAP1R1	1975	1997	1	1	-0.03	0.06	22	11	3UTR
hsa-miR-11400	NM_001199637	ADCYAP1R1	1828	1850	1	1	0.36	0.67	22	11	3UTR
hsa-miR-11400	NM_153840	ADGRF1	4496	4540	1	1	1.11	1.01	23	16	3UTR

mirnaid	refseqid	genesymbol	start	end	bindingp	seed	phylopstem	phylopflank	binding_region_length	longest_consecutive_pairings	position
hsa-miR-11400	XM_011514469	ADGRF1	4183	4227	1	1	0.00	0.00	23	16	3UTR
hsa-miR-11400	NM_153839	ADGRF2	4803	4822	1	1	-0.06	0.04	19	13	3UTR
hsa-miR-11400	NM_005756	ADGRG2	3820	3839	1	1	0.30	0.64	19	8	3UTR
hsa-miR-11400	NM_001079859	ADGRG2	3787	3806	1	1	0.64	0.55	19	8	3UTR
hsa-miR-11400	NM_001079860	ADGRG2	3763	3782	1	1	0.74	0.57	19	8	3UTR
hsa-miR-11400	NM_001184833	ADGRG2	3781	3800	1	1	0.55	0.67	19	8	3UTR
hsa-miR-11400	NM_001184836	ADGRG2	3757	3776	1	1	0.33	0.57	19	8	3UTR
hsa-miR-11400	NM_001184837	ADGRG2	3739	3758	1	1	0.39	0.48	19	8	3UTR
hsa-miR-11400	NM_001308360	ADGRG3	2117	2134	1	1	-0.10	-0.18	17	8	3UTR
hsa-miR-11400	NM_001297704	ADGRL2	4621	4635	1	1	4.65	4.45	14	13	3UTR
hsa-miR-11400	NM_001297705	ADGRL2	4762	4776	1	1	4.65	4.45	14	13	3UTR
hsa-miR-11400	NM_001297706	ADGRL2	4559	4573	1	1	4.65	4.45	14	13	3UTR
hsa-miR-11400	XM_005270668	ADGRL2	4802	4816	1	1	0.00	0.00	14	13	3UTR
hsa-miR-11400	XM_017000784	ADGRL2	4745	4759	1	1	0.00	0.00	14	13	3UTR
hsa-miR-11400	XM_017000789	ADGRL2	4726	4740	1	1	0.00	0.00	14	13	3UTR
hsa-miR-11400	XM_024454350	ADGRL2	5523	5537	1	1	0.00	0.00	14	13	3UTR
hsa-miR-11400	XM_024454355	ADGRL2	4636	4650	1	1	0.00	0.00	14	13	3UTR

mirnaid	refseqid	genesymbol	start	end	bindingp	seed	phylopstem	phylopflank	binding_region_length	longest_consecutive_pairings	position
hsa-miR-11400	NM_012302	ADGRL2	4656	4670	1	1	4.65	4.45	14	13	3UTR
hsa-miR-11400	NM_001330645	ADGRL2	4695	4709	1	1	4.65	4.45	14	13	3UTR
hsa-miR-11400	NM_001350699	ADGRL2	4726	4740	1	1	4.65	4.45	14	13	3UTR
hsa-miR-11400	NM_001366002	ADGRL2	4756	4770	1	1	4.65	4.45	14	13	3UTR
hsa-miR-11400	NM_001366009	ADGRL2	4767	4781	1	1	4.65	4.45	14	13	3UTR
hsa-miR-11400	NM_001286650	ADH1B	3751	3791	1	1	2.61	1.76	27	10	3UTR
hsa-miR-11400	NM_000668	ADH1B	3643	3669	1	1	0.20	1.29	26	10	3UTR
hsa-miR-11400	NM_004797	ADIPOQ	3491	3517	0.961538	1	0.33	0.29	19	10	3UTR
hsa-miR-11400	NM_001177800	ADIPOQ	3542	3568	0.961538	1	0.33	0.29	19	10	3UTR
hsa-miR-11400	NM_181442	ADNP	5059	5077	1	1	5.18	5.00	18	14	3UTR
hsa-miR-11400	NM_015339	ADNP	5234	5252	1	1	5.76	4.76	18	14	3UTR
hsa-miR-11400	NM_000676	ADORA2B	1358	1396	1	1	0.05	0.43	21	15	3UTR
hsa-miR-11400	NM_020233	ADPRM	1329	1349	1	1	-0.17	0.14	20	16	3UTR
hsa-miR-11400	NM_001267043	AEBP2	3382	3425	0.980769	1	0.01	0.15	20	12	3UTR
hsa-miR-11400	NM_198595	AFAP1	4717	4746	1	1	-0.73	-0.36	29	10	3UTR
hsa-miR-11400	NM_001134647	AFAP1	4969	4998	1	1	-0.39	-0.09	29	10	3UTR
hsa-miR-11400	NM_001313959	AFF1	5882	5901	1	1	-0.10	-0.11	19	8	3UTR

mirnaid	refseqid	genesymbol	start	end	bindingp	seed	phylopstem	phylopflank	binding_region_length	longest_consecutive_pairings	position
hsa-miR-11400	NM_005935	AFF1	5866	5885	1	1	-0.10	-0.11	19	8	3UTR
hsa-miR-11400	NM_001170628	AFF2	8062	8081	1	1	0.00	0.00	19	8	3UTR
hsa-miR-11400	NM_001386135	AFF3	9091	9115	1	1	0.00	0.00	24	9	3UTR
hsa-miR-11400	NM_006796	AFG3L2	2693	2714	1	1	0.20	2.14	21	13	3UTR
hsa-miR-11400	XM_011510549	AGAP1	10481	10533	1	1	0.00	0.00	33	11	3UTR
hsa-miR-11400	NM_004504	AGFG1	5242	5263	1	1	-0.13	-0.24	21	7	3UTR
hsa-miR-11400	NM_001135187	AGFG1	5308	5329	1	1	0.00	0.00	21	7	3UTR
hsa-miR-11400	NM_001135188	AGFG1	5236	5257	1	1	0.00	0.00	21	7	3UTR
hsa-miR-11400	NM_001135189	AGFG1	5122	5143	1	1	-0.13	-0.24	21	7	3UTR
hsa-miR-11400	XM_017012114	AGFG2	3919	3937	0.961538	1	0.00	0.00	18	8	3UTR
hsa-miR-11400	XM_024446835	AGK	4713	4740	1	1	0.00	0.00	21	8	3UTR
hsa-miR-11400	XM_005261160	AGPAT3	3534	3552	1	1	0.00	0.00	18	17	3UTR
hsa-miR-11400	XM_006724030	AGPAT3	3430	3448	1	1	0.00	0.00	18	17	3UTR
hsa-miR-11400	NM_020133	AGPAT4	2645	2660	1	1	-0.61	-0.79	15	14	3UTR
hsa-miR-11400	XM_011512041	AGPS	7212	7229	1	1	0.00	0.00	17	13	3UTR
hsa-miR-11400	NM_003659	AGPS	7400	7417	1	1	0.60	1.03	17	13	3UTR
hsa-miR-11400	XM_011541802	AGTRAP	1142	1170	1	1	0.00	0.00	22	10	3UTR

mirnaid	refseqid	genesymbol	start	end	bindingp	seed	phylopstem	phylopflank	binding_region_length	longest_consecutive_pairings	position
hsa-miR-11400	NM_001354571	AHSG	1217	1236	1	1	0.53	0.68	19	8	3UTR
hsa-miR-11400	NM_001622	AHSG	1214	1233	1	1	0.53	0.68	19	8	3UTR
hsa-miR-11400	NM_174858	AK5	2417	2434	1	1	-0.43	-0.12	17	16	3UTR
hsa-miR-11400	NM_012093	AK5	2585	2602	1	1	-0.43	-0.12	17	16	3UTR
hsa-miR-11400	NM_001317950	AKNA	5022	5044	1	1	0.10	-0.06	22	10	3UTR
hsa-miR-11400	NM_001317952	AKNA	4561	4583	1	1	-0.57	0.06	22	10	3UTR
hsa-miR-11400	NM_030767	AKNA	4948	4970	1	1	1.56	0.19	22	10	3UTR
hsa-miR-11400	XM_017012224	AKR1B15	1272	1290	1	1	0.00	0.00	18	8	3UTR
hsa-miR-11400	NM_005989	AKR1D1	1472	1485	1	1	0.02	0.03	13	12	3UTR
hsa-miR-11400	NM_001190906	AKR1D1	1349	1362	1	1	0.02	0.03	13	12	3UTR
hsa-miR-11400	NM_001190907	AKR1D1	1389	1402	1	1	0.02	0.03	13	12	3UTR
hsa-miR-11400	NM_001320979	AKR7A2	1705	1731	1	1	4.38	3.22	21	6	3UTR
hsa-miR-11400	XM_011526614	AKT2	1559	1580	1	1	0.00	0.00	21	14	3UTR
hsa-miR-11400	XM_024451417	AKT2	1574	1595	1	1	0.00	0.00	21	14	3UTR
hsa-miR-11400	NM_002860	ALDH18A1	3116	3155	1	1	1.01	2.02	39	8	3UTR
hsa-miR-11400	NM_001017423	ALDH18A1	3110	3149	1	1	1.01	2.02	39	8	3UTR
hsa-miR-11400	NM_001034173	ALDH1L2	4536	4556	1	1	-0.22	-0.44	20	10	3UTR

mirnaid	refseqid	genesymbol	start	end	bindingp	seed	phylopstem	phylopflank	binding_region_length	longest_consecutive_pairings	position
hsa-miR-11400	NM_000690	ALDH2	4099	4121	1	1	-0.05	0.07	22	9	3UTR
hsa-miR-11400	NM_021926	ALX4	4435	4455	1	1	5.39	3.97	20	6	3UTR
hsa-miR-11400	NM_001164690	AMHR2	1546	1566	1	1	2.98	1.94	20	10	3UTR
hsa-miR-11400	NM_198722	AMIGO3	2513	2538	1	1	1.78	0.40	25	11	3UTR
hsa-miR-11400	NM_015365	AMMECR1	3958	3978	1	1	-0.07	1.42	20	9	3UTR
hsa-miR-11400	NM_001025580	AMMECR1	3847	3867	1	1	0.32	1.24	20	9	3UTR
hsa-miR-11400	NM_133265	AMOT	4128	4150	1	1	3.66	2.90	22	11	3UTR
hsa-miR-11400	NM_001113490	AMOT	4543	4565	1	1	0.00	0.00	22	11	3UTR
hsa-miR-11400	NM_001301007	AMOTL1	7116	7135	1	1	0.29	-0.19	19	8	3UTR
hsa-miR-11400	NM_130847	AMOTL1	7266	7285	1	1	0.29	-0.19	19	8	3UTR
hsa-miR-11400	NM_001278685	AMOTL2	3062	3078	0.961538	1	2.35	2.46	16	8	3UTR
hsa-miR-11400	NM_001278683	AMOTL2	3192	3208	1	1	5.25	4.21	16	8	3UTR
hsa-miR-11400	XM_006713654	AMOTL2	3159	3175	1	1	0.00	0.00	16	8	3UTR
hsa-miR-11400	NM_001256708	ANAPC10	1480	1499	1	1	0.08	1.04	19	10	3UTR
hsa-miR-11400	NM_001278485	ANAPC15	596	612	1	1	3.75	4.43	16	15	3UTR
hsa-miR-11400	NM_001278489	ANAPC15	554	570	1	1	4.22	4.20	16	15	3UTR
hsa-miR-11400	NM_001278490	ANAPC15	499	515	1	1	2.77	4.14	16	15	3UTR

mirnaid	refseqid	genesymbol	start	end	bindingp	seed	phylopstem	phylopflank	binding_region_length	longest_consecutive_pairings	position
hsa-miR-11400	NM_001278492	ANAPC15	590	606	1	1	3.40	4.39	16	15	3UTR
hsa-miR-11400	NM_014042	ANAPC15	584	600	1	1	3.75	4.43	16	15	3UTR
hsa-miR-11400	NM_001330321	ANAPC15	1358	1407	1	1	2.72	4.26	25	10	3UTR
hsa-miR-11400	NM_173473	ANAPC16	546	563	1	1	0.39	0.34	17	9	3UTR
hsa-miR-11400	NM_001242546	ANAPC16	888	907	1	1	4.35	4.35	19	7	3UTR
hsa-miR-11400	NM_001242548	ANAPC16	405	422	1	1	0.00	0.00	17	9	3UTR
hsa-miR-11400	NM_004673	ANGPTL1	2976	2992	1	1	0.77	1.65	16	15	3UTR
hsa-miR-11400	NM_012098	ANGPTL2	2979	3008	1	1	2.23	2.50	29	10	3UTR
hsa-miR-11400	NM_001204403	ANK3	7861	7881	1	1	-0.12	-0.06	20	8	3UTR
hsa-miR-11400	NM_001358683	ANKRD40CL	731	756	1	1	-0.12	0.10	25	8	3UTR
hsa-miR-11400	NM_145865	ANKS4B	2751	2793	1	1	-0.30	-0.13	42	9	3UTR
hsa-miR-11400	NM_001144960	ANKUB1	2069	2089	1	1	0.15	0.26	20	7	3UTR
hsa-miR-11400	NM_020959	ANO8	3929	3945	1	1	0.84	0.65	16	15	3UTR
hsa-miR-11400	NM_032208	ANTXR1	3564	3582	1	1	0.12	0.12	18	11	3UTR
hsa-miR-11400	NM_004034	ANXA7	2081	2101	1	1	2.49	1.99	14	12	3UTR
hsa-miR-11400	NM_001156	ANXA7	2015	2035	1	1	2.49	1.99	14	12	3UTR
hsa-miR-11400	XM_011510600	AP1S3	1764	1785	1	1	0.00	0.00	21	9	3UTR

mirnaid	refseqid	genesymbol	start	end	bindingp	seed	phylopstem	phylopflank	binding_region_length	longest_consecutive_pairings	position
hsa-miR-11400	NM_001039569	AP1S3	1902	1923	1	1	-0.18	-0.01	21	9	3UTR
hsa-miR-11400	NM_001242837	AP2A2	3439	3456	1	1	0.00	0.00	17	10	3UTR
hsa-miR-11400	NM_207012	AP3M1	2019	2036	1	1	0.03	-0.01	17	11	3UTR
hsa-miR-11400	NM_005829	AP3S2	2381	2399	0.980769	1	0.43	-0.17	18	11	3UTR
hsa-miR-11400	NM_153360	APCDD1L	2296	2318	1	1	4.21	2.65	22	11	3UTR
hsa-miR-11400	NM_031301	APH1B	3595	3616	1	1	0.13	-0.13	21	11	3UTR
hsa-miR-11400	XM_024450085	APH1B	3600	3621	1	1	0.00	0.00	21	11	3UTR
hsa-miR-11400	NM_001145646	APH1B	3472	3493	1	1	0.27	0.12	21	11	3UTR
hsa-miR-11400	NM_001642	APLP2	3104	3119	0.974359	1	0.68	0.15	15	14	3UTR
hsa-miR-11400	NM_001142276	APLP2	3068	3083	0.974359	1	0.68	0.15	15	14	3UTR
hsa-miR-11400	NM_001142277	APLP2	2900	2915	0.974359	1	0.68	0.15	15	14	3UTR
hsa-miR-11400	NM_001142278	APLP2	2381	2396	0.974359	1	0.68	0.15	15	14	3UTR
hsa-miR-11400	NM_001243299	APLP2	3059	3074	0.974359	1	0.68	0.15	15	14	3UTR
hsa-miR-11400	XM_011529992	APOBEC3H	1109	1138	1	1	0.00	0.00	21	7	3UTR
hsa-miR-11400	NM_198450	APOOL	5596	5616	1	1	2.55	1.46	15	13	3UTR
hsa-miR-11400	NM_198450	APOOL	847	871	1	1	0.26	2.09	19	9	3UTR
hsa-miR-11400	NM_001251905	APPL2	2866	2885	1	1	5.36	4.60	19	10	3UTR

mirnaid	refseqid	genesymbol	start	end	bindingp	seed	phylopstem	phylopflank	binding_region_length	longest_consecutive_pairings	position
hsa-miR-11400	NM_001317384	AQP4	1413	1432	1	1	0.44	0.06	19	7	3UTR
hsa-miR-11400	NM_001317387	AQP4	1332	1351	1	1	-0.32	0.11	19	7	3UTR
hsa-miR-11400	NM_001318156	AQP7	2078	2101	1	1	0.00	0.00	23	13	3UTR
hsa-miR-11400	XM_017014700	AQP7	2570	2593	1	1	0.00	0.00	23	13	3UTR
hsa-miR-11400	XM_024447539	AQP7	1895	1918	1	1	0.00	0.00	23	13	3UTR
hsa-miR-11400	NM_014691	AQR	5265	5283	1	1	1.61	2.20	18	6	3UTR
hsa-miR-11400	NM_020340	ARFGEF3	7401	7421	1	1	0.37	0.27	20	12	3UTR
hsa-miR-11400	NM_001287431	ARFIP1	1770	1788	1	1	1.19	1.68	18	9	3UTR
hsa-miR-11400	NM_014447	ARFIP1	1674	1692	1	1	1.19	1.68	18	9	3UTR
hsa-miR-11400	NM_001025593	ARFIP1	1809	1827	1	1	1.19	1.68	18	9	3UTR
hsa-miR-11400	NM_001025595	ARFIP1	1905	1923	1	1	0.77	1.61	18	9	3UTR
hsa-miR-11400	NM_001242854	ARFIP2	1300	1320	1	1	2.84	1.61	20	6	3UTR
hsa-miR-11400	NM_001270696	ARHGAP12	3965	3982	1	1	5.03	3.78	17	16	3UTR
hsa-miR-11400	NM_018287	ARHGAP12	4055	4072	1	1	5.03	3.78	17	16	3UTR
hsa-miR-11400	NM_001204300	ARHGAP19	4020	4051	1	1	0.00	0.00	31	11	3UTR
hsa-miR-11400	NM_001287602	ARHGAP30	3415	3434	1	1	1.12	2.08	19	8	3UTR
hsa-miR-11400	NM_004309	ARHGDIA	1087	1107	1	1	0.81	1.04	15	13	3UTR

mirnaid	refseqid	genesymbol	start	end	bindingp	seed	phylopstem	phylopflank	binding_region_length	longest_consecutive_pairings	position
hsa-miR-11400	NM_001185078	ARHGDIA	955	975	1	1	0.95	0.42	15	13	3UTR
hsa-miR-11400	NM_173728	ARHGEF15	2723	2744	1	1	-0.16	0.46	21	10	3UTR
hsa-miR-11400	NM_173728	ARHGEF15	2893	2921	1	1	0.53	0.14	28	6	3UTR
hsa-miR-11400	NM_025014	ARHGEF15	2662	2683	1	1	-0.16	0.46	21	10	3UTR
hsa-miR-11400	NM_025014	ARHGEF15	2832	2860	1	1	0.53	0.14	28	6	3UTR
hsa-miR-11400	NM_015595	ARHGEF26	2965	2988	1	1	-0.20	0.00	23	9	3UTR
hsa-miR-11400	NM_001251962	ARHGEF26	3068	3091	1	1	0.00	0.00	23	9	3UTR
hsa-miR-11400	NM_001251963	ARHGEF26	2900	2923	1	1	-0.20	0.00	23	9	3UTR
hsa-miR-11400	NM_001289698	ARHGEF3	2069	2090	0.980769	1	0.91	3.23	21	8	3UTR
hsa-miR-11400	NM_001320854	ARHGEF7	3542	3572	1	1	0.02	0.14	30	9	3UTR
hsa-miR-11400	NM_005224	ARID3A	5364	5390	1	1	0.16	0.07	20	18	3UTR
hsa-miR-11400	NM_005744	ARIH1	9331	9358	1	1	-0.19	-0.02	27	8	3UTR
hsa-miR-11400	NM_001317333	ARIH2	2513	2531	1	1	0.12	0.24	18	11	3UTR
hsa-miR-11400	NM_006321	ARIH2	2391	2409	1	1	0.12	0.24	18	11	3UTR
hsa-miR-11400	NM_019087	ARL15	1893	1916	1	1	-0.07	0.30	23	11	3UTR
hsa-miR-11400	NM_001113738	ARL17A	2853	2887	1	1	-0.08	-0.16	22	9	3UTR
hsa-miR-11400	NM_001039083	ARL17B	2853	2887	1	1	0.20	-0.10	22	9	3UTR

mirnaid	refseqid	genesymbol	start	end	bindingp	seed	phylopstem	phylopflank	binding_region_length	longest_consecutive_pairings	position
hsa-miR-11400	NM_001195396	ARL4A	1044	1061	1	1	3.84	4.39	17	16	3UTR
hsa-miR-11400	NM_177985	ARL5A	3417	3437	1	1	0.83	0.38	20	7	3UTR
hsa-miR-11400	NM_012097	ARL5A	3675	3695	1	1	0.39	0.43	20	7	3UTR
hsa-miR-11400	XM_005260157	ARMC6	2055	2074	1	1	0.00	0.00	19	7	3UTR
hsa-miR-11400	NM_001267041	ARMC8	2989	3010	1	1	1.46	1.28	18	7	3UTR
hsa-miR-11400	NM_001267042	ARMC8	2598	2619	1	1	1.46	1.28	18	7	3UTR
hsa-miR-11400	NM_001282342	ARMC8	2629	2650	1	1	1.46	1.28	18	7	3UTR
hsa-miR-11400	NM_213654	ARMC8	1746	1775	1	1	2.50	2.26	21	9	3UTR
hsa-miR-11400	NM_015396	ARMC8	2875	2896	1	1	1.46	1.28	18	7	3UTR
hsa-miR-11400	NM_014154	ARMC8	1692	1721	1	1	2.50	2.26	21	9	3UTR
hsa-miR-11400	NM_001363941	ARMC8	3029	3050	1	1	1.46	1.28	18	7	3UTR
hsa-miR-11400	XM_006719112	ARNTL2	2643	2675	1	1	0.00	0.00	32	8	3UTR
hsa-miR-11400	XM_011520769	ARNTL2	2583	2615	1	1	0.00	0.00	32	8	3UTR
hsa-miR-11400	NM_020183	ARNTL2	2702	2734	1	1	0.30	0.20	32	8	3UTR
hsa-miR-11400	NM_001248002	ARNTL2	2660	2692	1	1	0.00	0.00	32	8	3UTR
hsa-miR-11400	NM_001248003	ARNTL2	2591	2623	1	1	0.00	0.00	32	8	3UTR
hsa-miR-11400	NM_001248004	ARNTL2	2558	2590	1	1	0.30	0.20	32	8	3UTR

mirnaid	refseqid	genesymbol	start	end	bindingp	seed	phylopstem	phylopflank	binding_region_length	longest_consecutive_pairings	position
hsa-miR-11400	NM_001248005	ARNTL2	2482	2514	1	1	0.30	0.20	32	8	3UTR
hsa-miR-11400	NM_001198793	ARPC4-TTLL3	2580	2599	1	1	0.19	0.49	19	8	3UTR
hsa-miR-11400	NM_001270439	ARPC5	4687	4713	0.980769	1	-0.06	-0.30	21	12	3UTR
hsa-miR-11400	NM_005717	ARPC5	4678	4704	0.980769	1	0.09	-0.14	21	12	3UTR
hsa-miR-11400	NM_001270439	ARPC5	6234	6251	1	1	2.59	1.17	17	16	3UTR
hsa-miR-11400	NM_005717	ARPC5	6225	6242	1	1	0.97	0.96	17	16	3UTR
hsa-miR-11400	NM_182616	ARPIN	2220	2256	1	1	-0.19	0.08	20	8	3UTR
hsa-miR-11400	NM_001199058	ARPIN-AP3S2	3056	3074	0.980769	1	-0.20	-0.20	18	11	3UTR
hsa-miR-11400	NM_001267618	ARPP21	1710	1757	1	1	0.03	0.52	20	7	3UTR
hsa-miR-11400	NM_198399	ARPP21	1604	1651	1	1	0.03	0.52	20	7	3UTR
hsa-miR-11400	NM_001025068	ARPP21	1616	1663	1	1	0.03	0.52	20	7	3UTR
hsa-miR-11400	NM_001025069	ARPP21	1625	1672	1	1	0.03	0.52	20	7	3UTR
hsa-miR-11400	NM_020801	ARRDC3	3849	3876	0.961538	1	4.85	5.74	27	8	3UTR
hsa-miR-11400	NM_001247996	ASAP1	3799	3821	0.961538	1	5.14	5.23	22	11	3UTR
hsa-miR-11400	XM_017013467	ASAP1	3530	3552	1	1	0.00	0.00	22	11	3UTR
hsa-miR-11400	NM_018482	ASAP1	3697	3719	1	1	4.81	3.81	22	11	3UTR
hsa-miR-11400	NM_001012428	ASB11	1976	1994	1	1	0.00	0.00	18	10	3UTR

mirnaid	refseqid	genesymbol	start	end	bindingp	seed	phylopstem	phylopflank	binding_region_length	longest_consecutive_pairings	position
hsa-miR-11400	NM_001201583	ASB11	1950	1968	1	1	0.00	0.00	18	10	3UTR
hsa-miR-11400	NM_001142733	ASB14	2746	2768	1	1	3.76	3.18	22	9	3UTR
hsa-miR-11400	NM_001319301	ASB8	829	848	0.969231	1	0.40	-0.07	19	9	3UTR
hsa-miR-11400	NM_001369093	ASCC1	1993	2030	1	1	3.86	4.14	37	14	3UTR
hsa-miR-11400	NM_001369108	ASCC1	1768	1805	1	1	3.76	3.70	37	14	3UTR
hsa-miR-11400	NM_001369111	ASCC1	1907	1944	1	1	3.86	4.14	37	14	3UTR
hsa-miR-11400	NM_001369112	ASCC1	1682	1719	1	1	3.76	3.70	37	14	3UTR
hsa-miR-11400	NM_001198799	ASCC1	1323	1342	1	1	3.42	2.22	19	16	3UTR
hsa-miR-11400	NM_001198800	ASCC1	1888	1925	1	1	3.76	3.70	37	14	3UTR
hsa-miR-11400	NM_032468	ASPH	1049	1066	1	1	0.56	1.68	17	10	3UTR
hsa-miR-11400	NM_032468	ASPH	1508	1529	1	1	0.81	0.72	21	8	3UTR
hsa-miR-11400	NM_001164751	ASPH	1046	1063	1	1	0.49	1.72	17	10	3UTR
hsa-miR-11400	NM_001164751	ASPH	2421	2438	1	0	2.20	2.00	17	11	3UTR
hsa-miR-11400	NM_001164751	ASPH	1505	1526	1	1	0.57	0.62	21	8	3UTR
hsa-miR-11400	NM_001164753	ASPH	1379	1400	1	1	0.95	0.34	21	8	3UTR
hsa-miR-11400	NM_032810	ATAD1	4431	4451	1	1	0.86	0.52	20	13	3UTR
hsa-miR-11400	NM_033064	ATCAY	4036	4061	1	1	-0.17	-0.23	25	8	3UTR

mirnaid	refseqid	genesymbol	start	end	bindingp	seed	phylopstem	phylopflank	binding_region_length	longest_consecutive_pairings	position
hsa-miR-11400	NM_012068	ATF5	1820	1852	1	1	-0.05	0.00	19	8	3UTR
hsa-miR-11400	NM_001193646	ATF5	1615	1647	1	1	-0.05	0.00	19	8	3UTR
hsa-miR-11400	NM_031482	ATG10	1148	1175	1	1	0.06	0.02	27	10	3UTR
hsa-miR-11400	XM_005248611	ATG10	991	1018	1	1	0.00	0.00	27	10	3UTR
hsa-miR-11400	NM_001131028	ATG10	1283	1310	1	1	0.06	0.02	27	10	3UTR
hsa-miR-11400	NM_014924	ATG14	2491	2536	1	1	1.30	0.58	22	9	3UTR
hsa-miR-11400	NM_001278712	ATG3	2626	2648	1	1	0.79	2.01	22	11	3UTR
hsa-miR-11400	NM_001308076	ATL2	2562	2580	1	1	4.39	3.64	18	8	3UTR
hsa-miR-11400	NM_022374	ATL2	2755	2773	1	1	4.25	4.44	18	8	3UTR
hsa-miR-11400	NM_001330458	ATL2	2033	2051	1	1	4.60	3.57	18	8	3UTR
hsa-miR-11400	NM_001135673	ATL2	2278	2296	1	1	4.25	4.44	18	8	3UTR
hsa-miR-11400	NM_012069	ATP1B4	1230	1263	1	1	1.23	0.98	33	8	3UTR
hsa-miR-11400	NM_001142447	ATP1B4	1242	1275	1	1	1.23	0.98	33	8	3UTR
hsa-miR-11400	NM_000704	ATP4A	3396	3429	1	1	1.09	2.85	20	13	3UTR
hsa-miR-11400	NM_001689	ATP5MC3	1914	1942	1	1	3.20	3.33	22	7	3UTR
hsa-miR-11400	NM_005765	ATP6AP2	1693	1708	1	1	0.75	0.44	15	14	3UTR
hsa-miR-11400	NM_000052	ATP7A	6969	6986	1	1	0.19	0.08	17	10	3UTR

mirnaid	refseqid	genesymbol	start	end	bindingp	seed	phylopstem	phylopflank	binding_region_length	longest_consecutive_pairings	position
hsa-miR-11400	XM_005266419	ATP8A2	9066	9089	1	1	0.00	0.00	23	10	3UTR
hsa-miR-11400	NM_016529	ATP8A2	9349	9372	1	1	1.80	1.56	23	10	3UTR
hsa-miR-11400	NM_138813	ATP8B3	4456	4476	1	1	1.30	2.03	20	9	3UTR
hsa-miR-11400	NM_001178002	ATP8B3	4404	4424	1	1	2.56	2.84	20	9	3UTR
hsa-miR-11400	NM_006045	ATP9A	5968	5988	1	1	4.50	4.91	20	13	3UTR
hsa-miR-11400	NM_001256418	ATPAF1	1640	1660	1	1	0.07	-0.08	20	7	3UTR
hsa-miR-11400	NM_001243728	ATPAF1	1549	1569	1	1	0.07	-0.08	20	7	3UTR
hsa-miR-11400	NM_001128164	ATXN1	7139	7158	1	1	3.75	3.45	19	12	3UTR
hsa-miR-11400	NM_001136262	ATXN7L3B	785	800	1	1	-0.08	0.18	15	9	3UTR
hsa-miR-11400	NM_024037	AUNIP	1417	1439	1	1	0.00	0.44	22	7	3UTR
hsa-miR-11400	NM_001127232	AUTS2	2886	2908	1	1	0.28	0.17	22	10	3UTR
hsa-miR-11400	NM_000706	AVPR1A	2657	2679	1	1	0.03	0.13	22	8	3UTR
hsa-miR-11400	XM_005265392	AZI2	3278	3316	1	1	0.00	0.00	21	12	3UTR
hsa-miR-11400	NM_022461	AZI2	3895	3925	1	1	5.44	3.15	22	7	3UTR
hsa-miR-11400	NM_001277155	B3GALNT2	3142	3167	1	1	1.44	1.34	25	8	3UTR
hsa-miR-11400	NM_003783	B3GALT2	2986	3008	1	1	2.99	2.95	22	7	3UTR
hsa-miR-11400	NM_054025	B3GAT1	2197	2213	1	1	1.08	0.95	16	15	3UTR

mirnaid	refseqid	genesymbol	start	end	bindingp	seed	phylopstem	phylopflank	binding_region_length	longest_consecutive_pairings	position
hsa-miR-11400	NM_194318	B3GLCT	3019	3056	1	1	0.55	0.05	19	8	3UTR
hsa-miR-11400	NM_001320743	B3GNTL1	2499	2521	1	1	3.29	2.65	22	10	3UTR
hsa-miR-11400	NM_001276468	B4GALNT1	3422	3440	1	1	1.98	1.38	18	9	3UTR
hsa-miR-11400	XM_005268773	B4GALNT1	3911	3929	1	1	0.00	0.00	18	9	3UTR
hsa-miR-11400	NM_004776	B4GALT5	1425	1449	1	1	1.22	1.74	24	8	3UTR
hsa-miR-11400	NM_015681	B9D1	775	792	0.980769	1	4.86	3.40	17	9	3UTR
hsa-miR-11400	NM_001321214	B9D1	864	881	0.980769	1	4.86	3.40	17	9	3UTR
hsa-miR-11400	NM_001321215	B9D1	3028	3046	1	1	4.33	4.21	18	9	3UTR
hsa-miR-11400	NM_004282	BAG2	3397	3414	1	1	-0.02	-0.12	17	14	3UTR
hsa-miR-11400	NM_017450	BAIAP2	2362	2380	1	1	-0.19	-0.13	18	8	3UTR
hsa-miR-11400	NM_001385146	BAIAP2	2355	2373	1	1	-0.19	-0.13	18	8	3UTR
hsa-miR-11400	NM_001286464	BAIAP3	4422	4440	1	1	-0.41	-0.55	18	17	3UTR
hsa-miR-11400	NM_003933	BAIAP3	4519	4537	1	1	-0.41	-0.55	18	17	3UTR
hsa-miR-11400	NM_001199096	BAIAP3	4361	4379	1	1	-0.41	-0.55	18	17	3UTR
hsa-miR-11400	NM_001199097	BAIAP3	4371	4389	1	1	-0.41	-0.55	18	17	3UTR
hsa-miR-11400	NM_001199098	BAIAP3	4302	4320	1	1	-0.41	-0.55	18	17	3UTR
hsa-miR-11400	NM_001199099	BAIAP3	4287	4305	1	1	-0.41	-0.55	18	17	3UTR

mirnaid	refseqid	genesymbol	start	end	bindingp	seed	phylopstem	phylopflank	binding_region_length	longest_consecutive_pairings	position
hsa-miR-11400	NM_001289975	BAZ2B	7032	7049	0.961538	1	2.74	3.06	17	7	3UTR
hsa-miR-11400	NM_014417	BBC3	1085	1106	1	1	0.89	1.11	21	8	3UTR
hsa-miR-11400	NM_001127241	BBC3	678	699	1	1	0.40	1.38	21	8	3UTR
hsa-miR-11400	NM_198428	BBS9	3334	3353	1	1	-0.04	0.12	19	8	3UTR
hsa-miR-11400	NM_001348036	BBS9	3124	3143	1	1	-0.04	0.12	19	8	3UTR
hsa-miR-11400	NM_001008405	BCAP29	2356	2383	1	1	0.45	0.16	19	10	3UTR
hsa-miR-11400	NM_001178093	BCAT1	4487	4506	1	1	-0.17	0.00	19	7	3UTR
hsa-miR-11400	NM_138622	BCL2L11	904	941	1	1	1.76	0.67	37	8	3UTR
hsa-miR-11400	NM_138623	BCL2L11	724	761	1	1	1.76	0.67	37	8	3UTR
hsa-miR-11400	NM_001204107	BCL2L11	706	743	1	1	1.76	0.67	37	8	3UTR
hsa-miR-11400	NM_001010922	BCL2L15	1885	1904	1	1	0.17	0.29	19	8	3UTR
hsa-miR-11400	NM_181844	BCL6B	1961	1978	1	1	2.76	0.69	17	8	3UTR
hsa-miR-11400	NM_001363659	BCLAF1	3843	3859	1	1	0.16	0.34	16	7	3UTR
hsa-miR-11400	NM_001136106	BEAN1	961	990	1	1	0.03	0.02	22	15	3UTR
hsa-miR-11400	NM_001178020	BEAN1	1068	1097	1	1	0.03	0.02	22	15	3UTR
hsa-miR-11400	NM_001367314	BEND3	4632	4670	1	1	4.20	3.27	38	10	3UTR
hsa-miR-11400	NM_001080450	BEND3	4845	4883	1	1	2.45	3.19	38	10	3UTR

mirnaid	refseqid	genesymbol	start	end	bindingp	seed	phylopstem	phylopflank	binding_region_length	longest_consecutive_pairings	position
hsa-miR-11400	NM_001711	BGN	1292	1306	1	1	-0.44	-0.08	14	13	3UTR
hsa-miR-11400	NM_001080512	BICC1	3239	3264	1	1	1.46	1.85	25	7	3UTR
hsa-miR-11400	NM_207311	BICDL1	2862	2898	1	1	1.26	1.74	24	14	3UTR
hsa-miR-11400	NM_182962	BIRC3	3267	3286	1	1	0.32	0.06	19	13	3UTR
hsa-miR-11400	NM_001165	BIRC3	5827	5846	1	1	0.32	0.06	19	13	3UTR
hsa-miR-11400	NM_001282436	BLOC1S2	1431	1444	1	1	0.34	0.21	13	6	3UTR
hsa-miR-11400	NM_173809	BLOC1S2	1323	1336	1	1	-0.49	0.04	13	6	3UTR
hsa-miR-11400	XM_017008382	BMP2K	3609	3653	1	1	0.00	0.00	44	8	3UTR
hsa-miR-11400	NM_001201	BMP3	3782	3809	1	1	0.00	0.00	27	10	3UTR
hsa-miR-11400	XM_011540103	BMPR1A	5758	5807	1	1	0.00	0.00	22	6	3UTR
hsa-miR-11400	NM_001204	BMPR2	11520	11545	1	1	0.00	0.00	21	19	3UTR
hsa-miR-11400	NM_004330	BNIP2	5722	5743	1	1	1.94	2.24	21	9	3UTR
hsa-miR-11400	NM_001320675	BNIP2	5731	5752	1	1	1.56	2.33	21	9	3UTR
hsa-miR-11400	NM_001010903	BNIP5	2944	2963	1	1	0.39	-0.38	19	14	3UTR
hsa-miR-11400	NM_138369	BOD1	1131	1149	1	1	-0.25	1.27	18	12	3UTR
hsa-miR-11400	NM_001159651	BOD1	934	952	1	1	-0.20	0.27	18	12	3UTR
hsa-miR-11400	NM_001300742	BORCS5	5819	5836	1	1	0.00	0.00	17	13	3UTR

mirnaid	refseqid	genesymbol	start	end	bindingp	seed	phylopstem	phylopflank	binding_region_length	longest_consecutive_pairings	position
hsa-miR-11400	NM_001300742	BORCS5	1321	1344	1	1	0.00	0.00	23	12	3UTR
hsa-miR-11400	NM_058169	BORCS5	6210	6227	1	1	-0.25	-0.05	17	13	3UTR
hsa-miR-11400	NM_001330356	BORCS5	6066	6083	1	1	-0.25	-0.05	17	13	3UTR
hsa-miR-11400	NM_007299	BRCA1	2659	2678	1	1	1.57	1.82	19	8	3UTR
hsa-miR-11400	NM_007371	BRD3	3340	3359	1	1	3.37	3.16	19	16	3UTR
hsa-miR-11400	NM_023924	BRD9	2161	2187	1	1	3.32	3.27	26	9	3UTR
hsa-miR-11400	NM_023924	BRD9	2240	2266	1	1	3.98	3.46	26	9	3UTR
hsa-miR-11400	NM_001242790	BRF1	1010	1032	1	1	0.00	0.00	22	16	3UTR
hsa-miR-11400	NM_032043	BRIP1	6442	6463	1	1	2.93	3.08	21	8	3UTR
hsa-miR-11400	NM_015399	BRMS1	1155	1197	0.961538	1	1.39	2.75	19	8	3UTR
hsa-miR-11400	NM_001024957	BRMS1	1073	1115	0.961538	1	1.39	2.75	19	8	3UTR
hsa-miR-11400	XM_005249010	BRPF3	3770	3814	1	1	0.00	0.00	44	10	3UTR
hsa-miR-11400	NM_001347943	BTBD11	4198	4220	1	1	2.33	1.52	22	9	3UTR
hsa-miR-11400	NM_001017523	BTBD11	2692	2714	1	1	2.33	1.52	22	9	3UTR
hsa-miR-11400	NM_001018072	BTBD11	4555	4577	1	1	2.33	1.52	22	9	3UTR
hsa-miR-11400	NM_001289133	BTBD7	4075	4095	1	1	0.54	0.74	20	9	3UTR
hsa-miR-11400	NM_015237	BTBD8	4219	4237	1	1	1.74	1.83	18	8	3UTR

mirnaid	refseqid	genesymbol	start	end	bindingp	seed	phylopstem	phylopflank	binding_region_length	longest_consecutive_pairings	position
hsa-miR-11400	NM_001281726	BTD	9875	9897	1	1	0.37	-0.01	22	10	3UTR
hsa-miR-11400	NM_001037637	BTF3	1129	1150	1	1	0.71	1.38	21	9	3UTR
hsa-miR-11400	NM_152265	BTF3L4	2348	2365	1	1	-0.04	0.50	17	9	3UTR
hsa-miR-11400	NM_001136497	BTF3L4	2281	2298	1	1	0.68	1.05	17	9	3UTR
hsa-miR-11400	NM_001243767	BTF3L4	2146	2163	1	1	0.00	0.00	17	9	3UTR
hsa-miR-11400	NM_001256856	BTRC	1912	1935	1	1	2.35	2.75	23	9	3UTR
hsa-miR-11400	NM_033637	BTRC	1990	2013	1	1	2.05	2.07	23	9	3UTR
hsa-miR-11400	NM_003939	BTRC	1882	1905	1	1	2.05	2.07	23	9	3UTR
hsa-miR-11400	NM_153714	C10orf67	2364	2385	1	1	1.29	0.96	21	7	3UTR
hsa-miR-11400	NM_001371909	C10orf67	2170	2190	1	1	0.39	0.86	20	9	3UTR
hsa-miR-11400	NM_001329958	C11orf21	551	572	1	1	-0.52	-0.24	21	10	3UTR
hsa-miR-11400	NM_001142946	C11orf21	636	657	1	1	-0.36	-0.17	21	10	3UTR
hsa-miR-11400	NM_173525	C11orf42	1102	1121	1	1	1.65	2.00	19	18	3UTR
hsa-miR-11400	NM_080659	C11orf52	1054	1077	1	1	0.53	0.16	18	10	3UTR
hsa-miR-11400	NM_031450	C11orf68	1421	1460	1	1	0.58	1.06	39	9	3UTR
hsa-miR-11400	NM_001135635	C11orf68	1424	1463	1	1	0.58	1.03	39	9	3UTR
hsa-miR-11400	NM_207645	C11orf87	5661	5679	0.961538	1	0.91	0.56	18	7	3UTR

mirnaid	refseqid	genesymbol	start	end	bindingp	seed	phylopstem	phylopflank	binding_region_length	longest_consecutive_pairings	position
hsa-miR-11400	NM_152318	C12orf45	21097	21120	1	1	0.08	0.06	23	11	3UTR
hsa-miR-11400	NM_152318	C12orf45	10959	10982	1	1	-0.07	0.01	23	6	3UTR
hsa-miR-11400	XM_011537985	C12orf50	2565	2584	0.961538	1	0.00	0.00	19	13	3UTR
hsa-miR-11400	NM_001099676	C12orf56	2694	2710	1	1	1.38	1.60	16	15	3UTR
hsa-miR-11400	NM_001170633	C12orf56	3174	3190	1	1	2.87	1.96	16	15	3UTR
hsa-miR-11400	XM_017019916	C12orf73	937	954	1	1	0.00	0.00	17	8	3UTR
hsa-miR-11400	XM_017019917	C12orf73	572	589	1	1	0.00	0.00	17	8	3UTR
hsa-miR-11400	NM_001135570	C12orf73	479	496	1	1	0.19	0.02	17	8	3UTR
hsa-miR-11400	NM_001252507	C14orf132	2421	2440	1	1	-0.17	-0.52	19	18	3UTR
hsa-miR-11400	NM_001252507	C14orf132	4703	4732	1	1	-0.27	-0.15	29	11	3UTR
hsa-miR-11400	NM_001282463	C14orf132	2520	2539	1	1	-0.17	-0.52	19	18	3UTR
hsa-miR-11400	NM_001282463	C14orf132	4802	4831	1	1	-0.27	-0.15	29	11	3UTR
hsa-miR-11400	NM_001289139	C14orf132	2514	2533	1	1	-0.17	-0.52	19	18	3UTR
hsa-miR-11400	NM_001289139	C14orf132	4796	4825	1	1	-0.27	-0.15	29	11	3UTR
hsa-miR-11400	NM_025187	C16orf70	1713	1731	1	1	1.56	1.56	18	13	3UTR
hsa-miR-11400	NM_001320542	C16orf70	1584	1602	1	1	1.56	1.56	18	13	3UTR
hsa-miR-11400	NM_014117	C16orf72	2097	2119	1	1	1.01	1.22	22	7	3UTR

mirnaid	refseqid	genesymbol	start	end	bindingp	seed	phylopstem	phylopflank	binding_region_length	longest_consecutive_pairings	position
hsa-miR-11400	NM_001348660	C16orf87	5826	5849	1	1	2.16	2.08	23	8	3UTR
hsa-miR-11400	NM_001105520	C17orf100	841	855	1	1	-0.36	0.45	14	7	3UTR
hsa-miR-11400	NM_152460	C17orf77	2050	2067	1	1	0.07	0.11	17	16	3UTR
hsa-miR-11400	NM_145055	C18orf25	4062	4083	1	1	0.01	0.22	21	8	3UTR
hsa-miR-11400	NM_001008239	C18orf25	3879	3900	1	1	0.12	0.26	21	8	3UTR
hsa-miR-11400	NM_001199346	C18orf32	4248	4297	1	1	-0.15	0.00	26	8	3UTR
hsa-miR-11400	NM_001282929	C19orf12	1873	1892	1	1	-0.33	-0.59	19	12	3UTR
hsa-miR-11400	NM_001282930	C19orf12	1860	1879	1	1	-0.38	-0.46	19	12	3UTR
hsa-miR-11400	NM_001136495	C1orf198	2455	2470	1	1	0.35	0.42	15	8	3UTR
hsa-miR-11400	NM_152374	C1orf216	1302	1325	1	1	0.04	-0.01	23	13	3UTR
hsa-miR-11400	NM_001297717	C1orf43	1213	1234	1	1	5.78	4.62	21	12	3UTR
hsa-miR-11400	NM_001297720	C1orf43	1272	1293	1	1	5.78	4.62	21	12	3UTR
hsa-miR-11400	NM_001297721	C1orf43	1019	1042	1	1	3.75	3.05	23	8	3UTR
hsa-miR-11400	NM_138740	C1orf43	1170	1191	1	1	2.75	3.00	21	12	3UTR
hsa-miR-11400	NM_015449	C1orf43	1224	1245	1	1	2.75	3.86	21	12	3UTR
hsa-miR-11400	NM_001098616	C1orf43	1326	1347	1	1	5.78	4.62	21	12	3UTR
hsa-miR-11400	NM_024097	C1orf50	4019	4041	1	1	0.36	0.03	22	7	3UTR

mirnaid	refseqid	genesymbol	start	end	bindingp	seed	phylopstem	phylopflank	binding_region_length	longest_consecutive_pairings	position
hsa-miR-11400	NM_001297642	C1RL	1215	1250	1	1	0.38	-0.06	35	12	3UTR
hsa-miR-11400	NM_001009984	C20orf194	4770	4789	1	1	3.11	2.50	19	9	3UTR
hsa-miR-11400	XM_024451878	C20orf203	2183	2202	1	1	0.00	0.00	19	9	3UTR
hsa-miR-11400	NM_019596	C21orf62	1932	1954	1	1	-0.03	-0.21	22	9	3UTR
hsa-miR-11400	NM_001162496	C21orf62	1851	1873	1	1	-0.35	0.02	22	9	3UTR
hsa-miR-11400	NM_001136263	C2CD4C	2342	2362	1	1	-0.04	0.95	20	7	3UTR
hsa-miR-11400	NM_001013649	C2orf68	2656	2680	1	1	-0.05	0.16	24	8	3UTR
hsa-miR-11400	NM_001162483	C2orf83	618	654	1	1	-0.12	-0.08	19	7	3UTR
hsa-miR-11400	NM_024616	C3orf52	1082	1117	1	1	-0.07	-0.36	18	12	3UTR
hsa-miR-11400	NM_001171747	C3orf52	901	922	1	1	0.00	0.00	21	9	3UTR
hsa-miR-11400	NM_001171747	C3orf52	829	864	1	1	0.00	0.00	18	12	3UTR
hsa-miR-11400	NM_001351622	C3orf85	809	823	1	1	0.15	0.02	14	13	3UTR
hsa-miR-11400	NM_001271749	C5AR2	1360	1381	1	1	0.27	0.18	21	9	3UTR
hsa-miR-11400	NM_018485	C5AR2	1260	1281	1	1	0.27	0.18	21	9	3UTR
hsa-miR-11400	NM_178569	C5orf38	3904	3934	1	1	0.00	0.00	30	11	3UTR
hsa-miR-11400	NM_175921	C5orf51	3665	3683	1	1	0.13	0.59	18	17	3UTR
hsa-miR-11400	NM_001286635	C6orf89	3845	3864	0.980769	1	-0.55	0.00	19	14	3UTR

mirnaid	refseqid	genesymbol	start	end	bindingp	seed	phylopstem	phylopflank	binding_region_length	longest_consecutive_pairings	position
hsa-miR-11400	NM_001286636	C6orf89	4023	4042	0.980769	1	-0.55	0.00	19	14	3UTR
hsa-miR-11400	NM_152734	C6orf89	3745	3764	0.980769	1	-0.55	0.00	19	14	3UTR
hsa-miR-11400	NM_023080	C8orf33	1887	1920	0.961538	1	0.00	-0.01	21	11	3UTR
hsa-miR-11400	NM_152571	C9orf163	2184	2201	1	1	-0.41	-0.75	17	16	3UTR
hsa-miR-11400	NM_173520	C9orf62	1678	1699	1	1	-0.06	-0.31	21	11	3UTR
hsa-miR-11400	NM_001291968	CA1	562	603	1	1	-0.16	0.14	13	11	3UTR
hsa-miR-11400	NM_001293642	CA12	4511	4534	1	1	0.00	0.00	23	8	3UTR
hsa-miR-11400	NM_206925	CA12	4691	4714	1	1	0.00	0.00	23	8	3UTR
hsa-miR-11400	NM_001218	CA12	4724	4747	1	1	0.00	0.00	23	8	3UTR
hsa-miR-11400	NM_004056	CA8	2757	2773	1	1	-0.69	0.20	16	15	3UTR
hsa-miR-11400	NM_031215	CABLES2	1467	1490	1	1	-0.54	0.01	23	9	3UTR
hsa-miR-11400	XM_005245478	CACNA1S	5764	5806	1	1	0.00	0.00	20	12	3UTR
hsa-miR-11400	XM_005252588	CACNB2	6690	6709	1	1	0.00	0.00	19	10	3UTR
hsa-miR-11400	XM_006717502	CACNB2	6604	6623	1	1	0.00	0.00	19	10	3UTR
hsa-miR-11400	NM_001206915	CACNB3	2358	2382	1	1	0.89	0.46	24	9	3UTR
hsa-miR-11400	NM_001206916	CACNB3	2150	2181	1	1	0.00	0.00	25	9	3UTR
hsa-miR-11400	NM_001330117	CACNB4	6398	6415	0.953846	1	5.24	5.41	17	13	3UTR

mirnaid	refseqid	genesymbol	start	end	bindingp	seed	phylopstem	phylopflank	binding_region_length	longest_consecutive_pairings	position
hsa-miR-11400	NM_000726	CACNB4	5962	5979	0.953846	1	4.86	4.36	17	13	3UTR
hsa-miR-11400	NM_001005746	CACNB4	5989	6006	0.953846	1	5.38	4.49	17	13	3UTR
hsa-miR-11400	NM_001320722	CACNB4	6252	6269	1	1	1.22	2.06	17	13	3UTR
hsa-miR-11400	NM_001330116	CACNB4	6063	6080	1	1	2.87	3.55	17	13	3UTR
hsa-miR-11400	NM_000726	CACNB4	5644	5664	1	1	1.64	1.54	20	7	3UTR
hsa-miR-11400	NM_153810	CACUL1	7767	7813	1	1	0.50	0.98	46	15	3UTR
hsa-miR-11400	NM_153810	CACUL1	6327	6346	1	1	0.45	0.24	19	8	3UTR
hsa-miR-11400	NM_001301044	CADM1	5799	5820	1	1	3.51	2.45	21	13	3UTR
hsa-miR-11400	NM_001098517	CADM1	5682	5703	1	1	0.49	1.14	21	13	3UTR
hsa-miR-11400	NM_153184	CADM2	2698	2716	1	1	4.80	4.19	16	14	3UTR
hsa-miR-11400	NM_007088	CALB2	916	955	1	1	0.76	0.22	24	8	3UTR
hsa-miR-11400	NM_001740	CALB2	1010	1049	1	1	0.75	0.07	24	8	3UTR
hsa-miR-11400	NM_020898	CALCOCO1	2465	2483	0.961538	1	-0.80	-0.39	13	11	3UTR
hsa-miR-11400	NM_001143682	CALCOCO1	2210	2228	0.961538	1	-0.16	-0.02	13	11	3UTR
hsa-miR-11400	XM_017019707	CALCOCO1	2603	2621	1	1	0.00	0.00	13	11	3UTR
hsa-miR-11400	NM_001271751	CALCRL	5571	5589	1	1	1.43	0.81	18	8	3UTR
hsa-miR-11400	NM_001369434	CALCRL	5804	5822	1	1	1.81	0.69	18	8	3UTR

mirnaid	refseqid	genesymbol	start	end	bindingp	seed	phylopstem	phylopflank	binding_region_length	longest_consecutive_pairings	position
hsa-miR-11400	NM_153711	CALHM5	3438	3457	1	1	-0.03	-0.01	19	9	3UTR
hsa-miR-11400	XM_017012678	CALN1	3684	3701	1	1	0.00	0.00	17	11	3UTR
hsa-miR-11400	NM_001363460	CALN1	3164	3181	1	1	0.36	0.48	17	11	3UTR
hsa-miR-11400	XM_005263396	CAMSAP1	5426	5445	1	1	0.00	0.00	19	8	3UTR
hsa-miR-11400	XM_005263397	CAMSAP1	4527	4546	1	1	0.00	0.00	19	8	3UTR
hsa-miR-11400	XM_006721478	CAMTA2	4977	5030	0.974359	1	0.00	0.00	24	11	3UTR
hsa-miR-11400	NM_001171167	CAMTA2	4779	4832	0.974359	1	0.25	0.21	24	11	3UTR
hsa-miR-11400	NM_001171168	CAMTA2	4709	4762	0.974359	1	0.25	0.21	24	11	3UTR
hsa-miR-11400	NM_018448	CAND1	8404	8425	0.969231	1	0.16	0.14	21	16	3UTR
hsa-miR-11400	NM_001748	CAPN2	2535	2559	1	1	0.20	0.34	24	8	3UTR
hsa-miR-11400	NM_001748	CAPN2	2319	2338	1	1	0.43	0.59	19	12	3UTR
hsa-miR-11400	NM_001146068	CAPN2	2431	2455	1	1	0.11	0.41	24	8	3UTR
hsa-miR-11400	NM_001146068	CAPN2	2215	2234	1	1	0.65	0.54	19	12	3UTR
hsa-miR-11400	NM_001385550	CAPRIN2	3112	3144	1	1	3.68	3.13	32	9	3UTR
hsa-miR-11400	NM_014316	CARHSP1	2530	2550	1	1	4.72	2.58	20	9	3UTR
hsa-miR-11400	NM_007359	CASC3	2166	2205	1	1	1.08	0.98	18	10	3UTR
hsa-miR-11400	XM_011543994	CASK	4201	4218	1	1	0.00	0.00	17	16	3UTR

mirnaid	refseqid	genesymbol	start	end	bindingp	seed	phylopstem	phylopflank	binding_region_length	longest_consecutive_pairings	position
hsa-miR-11400	XM_011543995	CASK	4168	4185	1	1	0.00	0.00	17	16	3UTR
hsa-miR-11400	XM_024452473	CASK	3776	3793	1	1	0.00	0.00	17	16	3UTR
hsa-miR-11400	NM_003688	CASK	4170	4187	1	1	0.00	0.00	17	16	3UTR
hsa-miR-11400	NM_001126054	CASK	4101	4118	1	1	0.00	0.00	17	16	3UTR
hsa-miR-11400	NM_001126055	CASK	4098	4115	1	1	0.00	0.00	17	16	3UTR
hsa-miR-11400	NM_020753	CASKIN2	4612	4640	1	1	0.19	0.30	28	9	3UTR
hsa-miR-11400	NM_032983	CASP2	661	683	1	1	2.01	2.88	22	10	3UTR
hsa-miR-11400	NM_001267056	CASP7	1070	1095	1	1	5.04	1.67	25	11	3UTR
hsa-miR-11400	NM_001227	CASP7	981	1006	1	1	3.31	2.99	25	11	3UTR
hsa-miR-11400	NM_000388	CASR	7078	7098	1	1	0.00	0.20	20	11	3UTR
hsa-miR-11400	NM_000388	CASR	7758	7776	1	1	0.09	0.25	18	12	3UTR
hsa-miR-11400	NM_001178065	CASR	7166	7186	1	1	0.00	0.20	20	11	3UTR
hsa-miR-11400	NM_001284212	CAST	2829	2846	1	1	0.42	0.76	17	16	3UTR
hsa-miR-11400	NM_001284213	CAST	2739	2756	1	1	0.42	0.76	17	16	3UTR
hsa-miR-11400	NM_173060	CAST	2889	2906	1	1	0.42	0.76	17	16	3UTR
hsa-miR-11400	NM_001330630	CAST	2790	2807	1	1	0.42	0.76	17	16	3UTR
hsa-miR-11400	NM_001330631	CAST	2916	2933	1	1	0.42	0.76	17	16	3UTR

mirnaid	refseqid	genesymbol	start	end	bindingp	seed	phylopstem	phylopflank	binding_region_length	longest_consecutive_pairings	position
hsa-miR-11400	NM_001330634	CAST	2859	2876	1	1	0.42	0.76	17	16	3UTR
hsa-miR-11400	NM_001042443	CAST	2955	2972	1	1	0.42	0.76	17	16	3UTR
hsa-miR-11400	NM_001042444	CAST	2832	2849	1	1	0.42	0.76	17	16	3UTR
hsa-miR-11400	NM_001042445	CAST	2850	2867	1	1	0.42	0.76	17	16	3UTR
hsa-miR-11400	XM_017007395	CBLB	5413	5434	1	1	0.00	0.00	21	8	3UTR
hsa-miR-11400	NM_012117	CBX5	10384	10404	1	1	1.18	0.89	20	10	3UTR
hsa-miR-11400	NM_001127321	CBX5	10347	10367	1	1	0.61	1.22	20	10	3UTR
hsa-miR-11400	NM_001127322	CBX5	10545	10565	1	1	1.12	1.50	20	10	3UTR
hsa-miR-11400	NM_020649	CBX8	2760	2778	1	1	0.61	1.13	18	17	3UTR
hsa-miR-11400	NM_001282959	CCAR1	4239	4258	1	1	0.07	-0.20	19	15	3UTR
hsa-miR-11400	NM_001282960	CCAR1	4233	4252	1	1	0.07	-0.20	19	15	3UTR
hsa-miR-11400	NM_018237	CCAR1	4278	4297	1	1	0.07	-0.20	19	15	3UTR
hsa-miR-11400	NM_001258395	CCDC103	2008	2039	1	1	4.16	2.80	16	14	3UTR
hsa-miR-11400	NM_001258396	CCDC103	2022	2053	1	1	4.16	2.80	16	14	3UTR
hsa-miR-11400	NM_213607	CCDC103	2038	2069	1	1	4.16	2.80	16	14	3UTR
hsa-miR-11400	NM_001271835	CCDC120	2779	2797	1	1	0.00	0.00	18	10	3UTR
hsa-miR-11400	NM_033626	CCDC120	2720	2738	1	1	0.00	0.00	18	10	3UTR

mirnaid	refseqid	genesymbol	start	end	bindingp	seed	phylopstem	phylopflank	binding_region_length	longest_consecutive_pairings	position
hsa-miR-11400	NM_001304797	CCDC134	5137	5157	1	1	-0.31	-0.09	20	10	3UTR
hsa-miR-11400	NM_024821	CCDC134	5476	5496	1	1	-0.31	-0.09	20	10	3UTR
hsa-miR-11400	NM_138803	CCDC148	2958	2993	1	1	0.53	0.31	16	14	3UTR
hsa-miR-11400	NM_001318335	CCDC157	1658	1706	1	1	-0.04	0.12	48	8	3UTR
hsa-miR-11400	XM_017006554	CCDC174	2029	2051	1	1	0.00	0.00	22	8	3UTR
hsa-miR-11400	NM_018017	CCDC186	6118	6140	1	1	3.47	3.50	22	10	3UTR
hsa-miR-11400	NM_001378188	CCDC187	7403	7422	1	1	0.17	0.40	19	15	3UTR
hsa-miR-11400	NM_001283056	CCDC198	2082	2105	1	1	1.86	0.89	23	8	3UTR
hsa-miR-11400	NM_015439	CCDC28A	951	973	1	1	-0.42	1.04	22	11	3UTR
hsa-miR-11400	NM_001382438	CCDC32	1462	1511	1	1	5.31	4.10	26	8	3UTR
hsa-miR-11400	NM_001143829	CCDC68	1882	1924	1	1	0.14	0.03	31	15	3UTR
hsa-miR-11400	XM_024448693	CCDC82	3164	3182	1	1	0.00	0.00	18	9	3UTR
hsa-miR-11400	XM_005264421	CCDC88A	6342	6377	1	1	0.00	0.00	35	10	3UTR
hsa-miR-11400	XM_011532968	CCDC88A	6201	6236	1	1	0.00	0.00	35	10	3UTR
hsa-miR-11400	NM_053056	CCND1	4053	4071	1	1	1.14	0.94	18	14	3UTR
hsa-miR-11400	NM_001759	CCND2	2442	2461	1	1	0.59	1.02	19	8	3UTR
hsa-miR-11400	NM_004354	CCNG2	5195	5217	1	1	0.01	0.13	22	7	3UTR

mirnaid	refseqid	genesymbol	start	end	bindingp	seed	phylopstem	phylopflank	binding_region_length	longest_consecutive_pairings	position
hsa-miR-11400	NM_001363539	CCNH	1614	1632	1	1	1.86	1.75	18	6	3UTR
hsa-miR-11400	NM_019084	CCNJ	3300	3332	1	1	2.96	2.58	32	8	3UTR
hsa-miR-11400	NM_001134375	CCNJ	3333	3365	1	1	2.96	2.58	32	8	3UTR
hsa-miR-11400	NM_001134376	CCNJ	3297	3329	1	1	2.96	2.58	32	8	3UTR
hsa-miR-11400	NM_014711	CCP110	4394	4413	1	1	-0.14	0.11	19	10	3UTR
hsa-miR-11400	NM_001323572	CCP110	4251	4270	1	1	-0.14	0.11	19	10	3UTR
hsa-miR-11400	NM_001199022	CCP110	4337	4356	1	1	-0.14	0.11	19	10	3UTR
hsa-miR-11400	NM_001145065	CCSER1	5046	5071	1	1	0.09	0.54	25	7	3UTR
hsa-miR-11400	NM_006016	CD164	2716	2735	1	1	0.17	0.97	19	7	3UTR
hsa-miR-11400	NM_001142401	CD164	2677	2696	1	1	0.17	0.97	19	7	3UTR
hsa-miR-11400	NM_001142402	CD164	2659	2678	1	1	0.17	0.97	19	7	3UTR
hsa-miR-11400	NM_001142403	CD164	2120	2139	1	1	0.17	0.97	19	7	3UTR
hsa-miR-11400	NM_001142404	CD164	2024	2043	1	1	0.17	0.97	19	7	3UTR
hsa-miR-11400	XM_011510119	CD1B	1238	1273	1	1	0.00	0.00	18	11	3UTR
hsa-miR-11400	XM_011512194	CD28	1939	1957	1	1	0.00	0.00	18	11	3UTR
hsa-miR-11400	NM_006139	CD28	1940	1958	1	1	0.15	-0.09	18	11	3UTR
hsa-miR-11400	NM_001243078	CD28	1583	1601	1	1	0.00	0.00	18	11	3UTR

mirnaid	refseqid	genesymbol	start	end	bindingp	seed	phylopstem	phylopflank	binding_region_length	longest_consecutive_pairings	position
hsa-miR-11400	NM_006110	CD2BP2	1842	1861	1	1	0.29	-0.31	19	10	3UTR
hsa-miR-11400	NM_001243646	CD2BP2	1972	1991	1	1	-0.07	-0.17	19	10	3UTR
hsa-miR-11400	NM_001775	CD38	5509	5533	1	1	0.23	0.05	24	7	3UTR
hsa-miR-11400	NM_000616	CD4	2143	2167	1	1	-0.79	-0.23	24	10	3UTR
hsa-miR-11400	NM_000074	CD40LG	1615	1632	1	1	0.57	0.40	17	8	3UTR
hsa-miR-11400	NM_203329	CD59	1125	1159	1	1	0.48	-0.10	23	9	3UTR
hsa-miR-11400	NM_203330	CD59	1226	1260	1	1	0.26	-0.02	23	9	3UTR
hsa-miR-11400	NM_001127223	CD59	1231	1265	1	1	0.10	0.06	23	9	3UTR
hsa-miR-11400	NM_001127225	CD59	1124	1158	1	1	0.45	-0.13	23	9	3UTR
hsa-miR-11400	NM_001254750	CD6	2559	2577	1	1	0.22	-0.38	18	15	3UTR
hsa-miR-11400	NM_001254751	CD6	2532	2550	1	1	0.22	-0.38	18	15	3UTR
hsa-miR-11400	NM_006725	CD6	2760	2778	1	1	0.22	-0.38	18	15	3UTR
hsa-miR-11400	NM_012072	CD93	3500	3516	1	1	-0.57	-0.34	16	15	3UTR
hsa-miR-11400	NM_001270436	CDC42EP3	3663	3703	1	1	0.64	1.12	40	7	3UTR
hsa-miR-11400	NM_001270438	CDC42EP3	3695	3735	1	1	0.75	1.69	40	7	3UTR
hsa-miR-11400	NM_006449	CDC42EP3	3777	3817	1	1	0.82	1.23	40	7	3UTR
hsa-miR-11400	NM_020239	CDC42SE1	851	903	1	1	0.14	-0.23	21	8	3UTR

mirnaid	refseqid	genesymbol	start	end	bindingp	seed	phylopstem	phylopflank	binding_region_length	longest_consecutive_pairings	position
hsa-miR-11400	NM_001220488	CDH13	4595	4613	1	1	0.51	0.60	18	7	3UTR
hsa-miR-11400	NM_001220488	CDH13	7213	7230	1	1	0.00	0.00	17	10	3UTR
hsa-miR-11400	NM_001271028	CDH19	4713	4737	1	1	2.23	2.15	24	9	3UTR
hsa-miR-11400	NM_021153	CDH19	5083	5107	1	1	2.23	2.15	24	9	3UTR
hsa-miR-11400	NM_031891	CDH20	3310	3331	1	1	0.32	0.18	21	15	3UTR
hsa-miR-11400	NM_177980	CDH26	4270	4285	1	1	0.00	0.02	15	14	3UTR
hsa-miR-11400	NM_021810	CDH26	2162	2177	1	1	0.00	0.02	15	14	3UTR
hsa-miR-11400	NM_001348204	CDH26	2039	2054	1	1	0.00	0.02	15	14	3UTR
hsa-miR-11400	XM_005255760	CDH8	9152	9195	1	1	0.00	0.00	43	11	3UTR
hsa-miR-11400	NM_001796	CDH8	4722	4741	1	1	0.67	2.59	19	10	3UTR
hsa-miR-11400	NM_033100	CDHR1	4627	4643	1	1	-0.15	0.11	16	15	3UTR
hsa-miR-11400	NM_033018	CDK16	2497	2515	1	1	1.92	1.62	18	14	3UTR
hsa-miR-11400	NM_006201	CDK16	2560	2578	1	1	1.92	1.62	18	14	3UTR
hsa-miR-11400	NM_001170460	CDK16	2396	2414	1	1	2.86	1.21	18	14	3UTR
hsa-miR-11400	XM_017014561	CDK20	1450	1475	1	1	0.00	0.00	25	10	3UTR
hsa-miR-11400	NM_001323289	CDKL5	11690	11714	1	1	0.00	0.05	24	8	3UTR
hsa-miR-11400	NM_001323289	CDKL5	14210	14231	1	1	0.24	0.51	21	8	3UTR

mirnaid	refseqid	genesymbol	start	end	bindingp	seed	phylopstem	phylopflank	binding_region_length	longest_consecutive_pairings	position
hsa-miR-11400	NM_004936	CDKN2B	1482	1499	1	1	-0.38	-0.01	17	16	3UTR
hsa-miR-11400	NM_001263	CDS1	3539	3555	1	1	-0.23	0.01	16	11	3UTR
hsa-miR-11400	NM_001282765	CDV3	873	894	1	1	1.51	0.50	21	14	3UTR
hsa-miR-11400	NM_001134422	CDV3	1158	1179	1	1	1.51	0.50	21	14	3UTR
hsa-miR-11400	NM_001134423	CDV3	715	736	1	1	1.51	0.50	21	14	3UTR
hsa-miR-11400	NM_001804	CDX1	1630	1648	1	1	0.26	0.52	18	9	3UTR
hsa-miR-11400	NM_002483	CEACAM6	1644	1673	1	1	0.05	-0.05	29	9	3UTR
hsa-miR-11400	NM_006561	CELF2	7486	7509	1	1	2.19	2.98	23	10	3UTR
hsa-miR-11400	NM_001172673	CELF5	2310	2339	1	1	-0.82	-0.38	29	9	3UTR
hsa-miR-11400	NM_001810	CENPB	2140	2159	0.978022	1	-0.10	0.15	19	9	3UTR
hsa-miR-11400	NM_018451	CENPJ	4521	4539	1	1	0.22	1.41	18	6	3UTR
hsa-miR-11400	NM_001270473	CENPN	2558	2582	1	1	0.14	0.03	24	12	3UTR
hsa-miR-11400	NM_001270474	CENPN	2516	2540	1	1	0.14	0.03	24	12	3UTR
hsa-miR-11400	NM_001100624	CENPN	2618	2642	1	1	0.14	0.03	24	12	3UTR
hsa-miR-11400	NM_001199803	CENPO	3385	3404	1	1	0.00	0.00	19	7	3UTR
hsa-miR-11400	NM_001012267	CENPP	7871	7890	1	1	-0.51	-0.49	19	10	3UTR
hsa-miR-11400	NM_001040157	CEP44	2345	2369	1	1	0.13	0.67	15	13	3UTR

mirnaid	refseqid	genesymbol	start	end	bindingp	seed	phylopstem	phylopflank	binding_region_length	longest_consecutive_pairings	position
hsa-miR-11400	NM_001145314	CEP44	2552	2574	1	1	0.35	0.33	22	9	3UTR
hsa-miR-11400	NM_016122	CEP83	2924	2938	1	1	3.33	2.98	14	7	3UTR
hsa-miR-11400	NM_001042399	CEP83	2871	2885	1	1	0.62	0.14	14	7	3UTR
hsa-miR-11400	NM_022766	CERK	2488	2514	1	1	-0.39	-0.37	26	10	3UTR
hsa-miR-11400	NM_001164496	CFAP44	9763	9781	1	1	4.03	2.14	18	12	3UTR
hsa-miR-11400	NM_001348523	CFAP92	3393	3419	1	1	0.00	0.00	17	6	3UTR
hsa-miR-11400	NM_021023	CFHR3	1635	1653	1	1	0.34	0.26	18	12	3UTR
hsa-miR-11400	NM_001166624	CFHR3	1452	1470	1	1	0.00	0.00	18	12	3UTR
hsa-miR-11400	NM_003879	CFLAR	5537	5557	1	1	0.04	-0.13	20	16	3UTR
hsa-miR-11400	NM_001351590	CFLAR	6376	6396	1	1	0.04	-0.13	20	16	3UTR
hsa-miR-11400	NM_001127183	CFLAR	5405	5425	1	1	0.04	-0.13	20	16	3UTR
hsa-miR-11400	NM_001202516	CFLAR	5432	5452	1	1	2.10	2.36	20	16	3UTR
hsa-miR-11400	NM_001202517	CFLAR	5119	5139	1	1	-0.26	0.04	20	16	3UTR
hsa-miR-11400	NM_001202518	CFLAR	4764	4807	1	1	0.25	0.00	26	8	3UTR
hsa-miR-11400	XM_011513235	CHCHD6	3742	3759	1	1	0.00	0.00	17	10	3UTR
hsa-miR-11400	NM_001271	CHD2	7772	7795	1	1	-0.44	-0.52	23	8	3UTR
hsa-miR-11400	NM_001161346	CHFR	5436	5455	1	1	0.16	-0.08	19	8	3UTR

mirnaid	refseqid	genesymbol	start	end	bindingp	seed	phylopstem	phylopflank	binding_region_length	longest_consecutive_pairings	position
hsa-miR-11400	NM_023947	CHID1	2007	2030	1	1	2.05	0.59	23	11	3UTR
hsa-miR-11400	NM_001142676	CHID1	2082	2105	1	1	2.09	1.07	23	11	3UTR
hsa-miR-11400	NM_001253387	CHL1	4468	4487	1	1	0.21	0.46	19	11	3UTR
hsa-miR-11400	NM_001253388	CHL1	3976	3995	1	1	0.17	0.64	19	11	3UTR
hsa-miR-11400	NM_006614	CHL1	4516	4535	1	1	0.17	0.64	19	11	3UTR
hsa-miR-11400	NM_000748	CHRNB2	2037	2071	1	1	0.01	-0.48	21	9	3UTR
hsa-miR-11400	NM_005199	CHRNG	2880	2913	1	1	0.20	0.08	21	14	3UTR
hsa-miR-11400	NM_014863	CHST15	3301	3317	1	1	-0.21	0.56	16	15	3UTR
hsa-miR-11400	NM_004273	CHST3	3466	3486	1	1	-0.15	0.10	20	7	3UTR
hsa-miR-11400	NM_021615	CHST6	3988	4009	0.953846	1	-0.13	0.02	21	12	3UTR
hsa-miR-11400	XM_005255955	CHST6	2531	2560	0.969231	1	0.00	0.00	20	18	3UTR
hsa-miR-11400	NM_021615	CHST6	2386	2415	0.969231	1	-0.31	0.03	20	18	3UTR
hsa-miR-11400	NM_001040146	CHTF8	2358	2385	1	1	2.61	1.70	20	11	3UTR
hsa-miR-11400	NM_001277764	CIB1	996	1019	1	1	0.34	0.57	23	14	3UTR
hsa-miR-11400	NM_198491	CIBAR2	1439	1457	1	1	2.61	2.14	18	7	3UTR
hsa-miR-11400	XM_006713717	CIP2A	7447	7461	1	1	0.00	0.00	14	13	3UTR
hsa-miR-11400	NM_033426	CIPC	1528	1548	1	1	-0.09	0.31	20	15	3UTR

mirnaid	refseqid	genesymbol	start	end	bindingp	seed	phylopstem	phylopflank	binding_region_length	longest_consecutive_pairings	position
hsa-miR-11400	NM_015127	CLCC1	2228	2245	1	1	0.13	0.08	17	8	3UTR
hsa-miR-11400	NM_001377458	CLCC1	2539	2556	1	1	0.11	-0.09	17	8	3UTR
hsa-miR-11400	NM_001377460	CLCC1	2383	2400	1	1	0.47	0.48	17	8	3UTR
hsa-miR-11400	NM_001377461	CLCC1	2378	2395	1	1	0.18	0.51	17	8	3UTR
hsa-miR-11400	NM_001377464	CLCC1	2338	2355	1	1	0.38	0.41	17	8	3UTR
hsa-miR-11400	NM_001377467	CLCC1	2489	2506	1	1	-0.06	0.08	17	8	3UTR
hsa-miR-11400	NM_001377469	CLCC1	2276	2293	1	1	0.06	0.10	17	8	3UTR
hsa-miR-11400	NM_001377470	CLCC1	2549	2566	1	1	-0.07	-0.05	17	8	3UTR
hsa-miR-11400	NM_001048210	CLCC1	2368	2385	1	1	0.26	0.39	17	8	3UTR
hsa-miR-11400	NM_173872	CLCN3	4324	4356	1	1	1.73	2.29	32	12	3UTR
hsa-miR-11400	NM_173872	CLCN3	3351	3369	1	1	-0.37	1.17	18	14	3UTR
hsa-miR-11400	NM_001829	CLCN3	4248	4280	1	1	1.73	2.29	32	12	3UTR
hsa-miR-11400	NM_001829	CLCN3	3275	3293	1	1	-0.37	1.17	18	14	3UTR
hsa-miR-11400	NM_001243372	CLCN3	4167	4199	1	1	1.73	2.29	32	12	3UTR
hsa-miR-11400	NM_001243372	CLCN3	3194	3212	1	1	-0.37	1.17	18	14	3UTR
hsa-miR-11400	NM_000084	CLCN5	3712	3736	1	1	1.01	1.24	24	13	3UTR
hsa-miR-11400	NM_000084	CLCN5	3644	3661	1	1	1.51	0.52	17	8	3UTR

mirnaid	refseqid	genesymbol	start	end	bindingp	seed	phylopstem	phylopflank	binding_region_length	longest_consecutive_pairings	position
hsa-miR-11400	NM_001127898	CLCN5	4103	4127	1	1	1.01	1.24	24	13	3UTR
hsa-miR-11400	NM_001127898	CLCN5	4035	4052	1	1	1.51	0.52	17	8	3UTR
hsa-miR-11400	NM_001127899	CLCN5	4362	4386	1	1	1.01	1.24	24	13	3UTR
hsa-miR-11400	NM_001127899	CLCN5	4294	4311	1	1	1.51	0.52	17	8	3UTR
hsa-miR-11400	NM_182848	CLDN10	1721	1748	1	1	-0.36	-0.13	27	9	3UTR
hsa-miR-11400	NM_148960	CLDN19	1036	1058	1	1	-0.20	0.03	22	8	3UTR
hsa-miR-11400	NM_001040199	CLDND1	1686	1702	1	1	2.51	2.71	16	7	3UTR
hsa-miR-11400	NM_001040200	CLDND1	1064	1085	1	1	1.19	0.68	17	7	3UTR
hsa-miR-11400	NM_207390	CLEC17A	2838	2863	1	1	0.00	0.00	25	8	3UTR
hsa-miR-11400	NM_207390	CLEC17A	1989	2016	1	1	0.08	0.04	27	7	3UTR
hsa-miR-11400	NM_001297748	CLEC1A	2029	2082	1	1	2.87	1.59	35	8	3UTR
hsa-miR-11400	NM_016511	CLEC1A	2147	2181	1	1	2.24	1.40	34	8	3UTR
hsa-miR-11400	NM_013269	CLEC2D	1655	1674	1	1	0.33	0.53	19	11	3UTR
hsa-miR-11400	NM_001004419	CLEC2D	1737	1756	1	1	0.33	0.53	19	11	3UTR
hsa-miR-11400	NM_001197317	CLEC2D	1544	1563	1	1	0.33	0.53	19	11	3UTR
hsa-miR-11400	NM_001197318	CLEC2D	1551	1570	1	1	0.33	0.53	19	11	3UTR
hsa-miR-11400	NM_001197319	CLEC2D	1440	1459	1	1	0.33	0.53	19	11	3UTR

mirnaid	refseqid	genesymbol	start	end	bindingp	seed	phylopstem	phylopflank	binding_region_length	longest_consecutive_pairings	position
hsa-miR-11400	NM_018941	CLN8	4725	4750	1	1	-0.05	0.09	25	8	3UTR
hsa-miR-11400	NM_004898	CLOCK	3157	3176	1	1	1.18	1.79	19	15	3UTR
hsa-miR-11400	NM_006012	CLPP	1723	1751	1	1	0.74	0.30	19	15	3UTR
hsa-miR-11400	NM_001256819	CLRN1	2228	2247	1	1	3.03	1.17	19	13	3UTR
hsa-miR-11400	NM_052995	CLRN1	1009	1028	1	1	2.69	1.67	19	13	3UTR
hsa-miR-11400	NM_174878	CLRN1	2056	2075	1	1	3.03	1.17	19	13	3UTR
hsa-miR-11400	XM_005256181	CMIP	2187	2202	1	1	0.00	0.00	15	14	3UTR
hsa-miR-11400	XM_011523353	CMIP	2324	2339	1	1	0.00	0.00	15	14	3UTR
hsa-miR-11400	NM_030629	CMIP	2225	2240	1	1	3.03	0.81	15	14	3UTR
hsa-miR-11400	NM_001142344	CMKLR1	4750	4771	1	1	2.87	2.57	21	11	3UTR
hsa-miR-11400	NM_001142345	CMKLR1	4222	4238	1	1	3.10	2.60	16	9	3UTR
hsa-miR-11400	NM_032359	CMSS1	1506	1525	1	1	-0.01	0.13	19	10	3UTR
hsa-miR-11400	NM_001167924	CMSS1	1390	1409	1	1	0.00	0.00	19	10	3UTR
hsa-miR-11400	XM_011533319	CMTM7	737	758	1	1	0.00	0.00	21	11	3UTR
hsa-miR-11400	NM_001277197	CNIH4	355	376	1	1	0.89	2.36	21	12	3UTR
hsa-miR-11400	NM_014184	CNIH4	496	517	1	1	0.89	2.36	21	12	3UTR
hsa-miR-11400	NM_020348	CNNM1	4256	4281	1	1	0.22	0.01	25	8	3UTR

mirnaid	refseqid	genesymbol	start	end	bindingp	seed	phylopstem	phylopflank	binding_region_length	longest_consecutive_pairings	position
hsa-miR-11400	NM_017546	CNOT11	2009	2029	1	1	0.24	-0.08	20	8	3UTR
hsa-miR-11400	NM_001369813	CNPY1	2175	2193	1	1	0.40	2.13	18	14	3UTR
hsa-miR-11400	NM_001103176	CNPY1	2011	2029	1	1	3.54	2.79	18	14	3UTR
hsa-miR-11400	NM_033181	CNR1	3394	3412	1	1	1.05	0.93	18	7	3UTR
hsa-miR-11400	NM_001365874	CNR1	3561	3579	1	1	1.95	1.83	18	7	3UTR
hsa-miR-11400	NM_173478	CNTD1	1889	1907	1	1	1.12	1.00	18	10	3UTR
hsa-miR-11400	NM_001330222	CNTD1	1671	1689	1	1	1.12	1.00	18	10	3UTR
hsa-miR-11400	NM_033655	CNTNAP3	9089	9110	1	1	0.19	0.81	21	8	3UTR
hsa-miR-11400	NM_001201380	CNTNAP3B	4333	4354	1	1	0.00	0.00	21	7	3UTR
hsa-miR-11400	NM_001008215	COA5	683	707	1	1	0.34	0.07	24	8	3UTR
hsa-miR-11400	NM_023077	COA7	3209	3228	1	1	0.07	0.37	19	10	3UTR
hsa-miR-11400	NM_001287436	COBL	4581	4599	0.953846	1	3.53	2.88	18	8	3UTR
hsa-miR-11400	NM_015198	COBL	4551	4569	0.953846	1	3.53	2.88	18	8	3UTR
hsa-miR-11400	NM_001287436	COBL	5252	5270	1	1	1.54	0.50	18	8	3UTR
hsa-miR-11400	XM_011515240	COBL	4485	4503	1	1	0.00	0.00	18	8	3UTR
hsa-miR-11400	XM_011515240	COBL	5156	5174	1	1	0.00	0.00	18	8	3UTR
hsa-miR-11400	XM_011515241	COBL	3846	3864	1	1	0.00	0.00	18	8	3UTR

mirnaid	refseqid	genesymbol	start	end	bindingp	seed	phylopstem	phylopflank	binding_region_length	longest_consecutive_pairings	position
hsa-miR-11400	XM_011515241	COBL	4517	4535	1	1	0.00	0.00	18	8	3UTR
hsa-miR-11400	NM_015198	COBL	5222	5240	1	1	1.54	0.50	18	8	3UTR
hsa-miR-11400	NM_181733	COG5	3735	3765	1	1	0.00	0.00	16	14	3UTR
hsa-miR-11400	NM_006348	COG5	3798	3828	1	1	3.25	4.24	16	14	3UTR
hsa-miR-11400	NM_032382	COG8	3017	3042	0.961538	1	3.58	3.35	25	8	3UTR
hsa-miR-11400	NM_032382	COG8	4488	4510	1	1	2.36	3.77	22	11	3UTR
hsa-miR-11400	NM_000494	COL17A1	5480	5508	1	1	0.71	1.45	28	10	3UTR
hsa-miR-11400	NM_173465	COL23A1	2132	2152	1	1	4.79	3.20	20	6	3UTR
hsa-miR-11400	XM_011534692	COL23A1	3127	3147	1	1	0.00	0.00	20	6	3UTR
hsa-miR-11400	NM_152890	COL24A1	5839	5860	1	1	2.49	2.40	21	9	3UTR
hsa-miR-11400	NM_017845	COMMD8	882	897	1	1	4.59	2.32	15	14	3UTR
hsa-miR-11400	NM_001001740	COP1	2604	2625	1	1	0.88	0.81	21	5	3UTR
hsa-miR-11400	NM_020441	CORO1B	3296	3314	1	1	5.49	4.85	18	7	3UTR
hsa-miR-11400	NM_001276471	CORO1C	3308	3351	1	1	4.38	4.47	25	8	3UTR
hsa-miR-11400	NM_014325	CORO1C	3177	3220	1	1	3.70	4.69	25	8	3UTR
hsa-miR-11400	NM_001105237	CORO1C	3290	3333	1	1	3.86	4.69	25	8	3UTR
hsa-miR-11400	NM_052820	CORO2A	3622	3635	1	1	1.37	0.94	13	12	3UTR

mirnaid	refseqid	genesymbol	start	end	bindingp	seed	phylopstem	phylopflank	binding_region_length	longest_consecutive_pairings	position
hsa-miR-11400	NM_003389	CORO2A	3809	3822	1	1	1.11	2.02	13	12	3UTR
hsa-miR-11400	NM_003389	CORO2A	2802	2821	1	1	0.01	-0.24	19	10	3UTR
hsa-miR-11400	NM_001297732	COX18	2182	2203	1	1	0.29	0.29	21	12	3UTR
hsa-miR-11400	NM_173827	COX18	2179	2200	1	1	0.38	0.27	21	12	3UTR
hsa-miR-11400	NM_001318794	COX4I1	821	864	1	1	6.35	2.93	15	13	3UTR
hsa-miR-11400	NM_004255	COX5A	1141	1164	1	1	1.13	0.67	23	8	3UTR
hsa-miR-11400	NM_001870	CPA3	1509	1540	1	1	0.58	0.01	18	10	3UTR
hsa-miR-11400	NM_001304	CPD	4410	4431	1	1	2.54	1.61	21	8	3UTR
hsa-miR-11400	NM_001199775	CPD	3768	3796	1	1	2.87	1.65	22	8	3UTR
hsa-miR-11400	NM_001355613	CPHXL	1307	1351	1	1	0.37	-0.06	18	9	3UTR
hsa-miR-11400	NM_001008220	CPLX2	3001	3019	1	1	0.26	-0.02	18	10	3UTR
hsa-miR-11400	NM_001300947	CPSF6	6599	6619	1	1	2.11	3.05	20	11	3UTR
hsa-miR-11400	NM_007007	CPSF6	6488	6508	1	1	2.11	3.05	20	11	3UTR
hsa-miR-11400	NM_032680	CRACR2A	2033	2050	1	1	0.56	0.33	17	8	3UTR
hsa-miR-11400	NM_001320100	CRADD	1037	1066	1	1	2.04	1.05	19	8	3UTR
hsa-miR-11400	NM_001320100	CRADD	924	954	1	1	0.08	1.06	23	7	3UTR
hsa-miR-11400	XM_005251934	CRB2	5547	5566	0.961538	1	0.00	0.00	19	18	3UTR

mirnaid	refseqid	genesymbol	start	end	bindingp	seed	phylopstem	phylopflank	binding_region_length	longest_consecutive_pairings	position
hsa-miR-11400	NM_019060	CRCT1	638	657	1	1	0.62	0.23	19	6	3UTR
hsa-miR-11400	NM_182898	CREB5	5538	5559	1	1	0.32	0.16	21	8	3UTR
hsa-miR-11400	NM_182899	CREB5	5187	5208	1	1	0.32	0.16	21	8	3UTR
hsa-miR-11400	XM_024447005	CREB5	5083	5104	1	1	0.00	0.00	21	8	3UTR
hsa-miR-11400	NM_004904	CREB5	5212	5233	1	1	0.32	0.16	21	8	3UTR
hsa-miR-11400	NM_001011666	CREB5	4742	4763	1	1	0.32	0.16	21	8	3UTR
hsa-miR-11400	NM_001310	CREBL2	2367	2387	1	1	1.12	0.62	20	7	3UTR
hsa-miR-11400	NM_182717	CREM	455	475	1	1	2.58	2.60	20	13	3UTR
hsa-miR-11400	NM_182720	CREM	419	439	1	1	2.58	2.60	20	13	3UTR
hsa-miR-11400	NM_183011	CREM	989	1009	1	1	2.58	2.60	20	13	3UTR
hsa-miR-11400	NM_183012	CREM	800	820	1	1	2.58	2.60	20	13	3UTR
hsa-miR-11400	NM_183013	CREM	1029	1049	1	1	2.58	2.60	20	13	3UTR
hsa-miR-11400	XM_017015723	CREM	898	918	1	1	0.00	0.00	20	13	3UTR
hsa-miR-11400	NM_001352466	CREM	530	550	1	1	2.58	2.60	20	13	3UTR
hsa-miR-11400	NM_001202483	CRHR2	1872	1913	1	1	0.00	0.00	41	9	3UTR
hsa-miR-11400	NM_021151	CROT	2528	2545	1	1	-0.05	0.44	17	16	3UTR
hsa-miR-11400	NM_001143935	CROT	2612	2629	1	1	-0.05	0.44	17	16	3UTR

mirnaid	refseqid	genesymbol	start	end	bindingp	seed	phylopstem	phylopflank	binding_region_length	longest_consecutive_pairings	position
hsa-miR-11400	NM_022769	CRTC3	4341	4373	1	1	-0.55	-0.27	32	10	3UTR
hsa-miR-11400	NM_001042574	CRTC3	4338	4370	1	1	-0.55	-0.27	32	10	3UTR
hsa-miR-11400	NM_000554	CRX	2495	2514	1	1	0.20	-0.14	19	8	3UTR
hsa-miR-11400	NM_015974	CRYL1	1108	1138	1	1	2.67	2.46	21	9	3UTR
hsa-miR-11400	NM_001363647	CRYL1	946	976	1	1	3.92	2.93	21	9	3UTR
hsa-miR-11400	XM_024449014	CSAD	1887	1912	1	1	0.00	0.00	25	8	3UTR
hsa-miR-11400	XM_024449015	CSAD	1873	1898	1	1	0.00	0.00	25	8	3UTR
hsa-miR-11400	NM_139062	CSNK1D	1653	1675	1	1	2.63	1.70	22	12	3UTR
hsa-miR-11400	NM_001893	CSNK1D	1589	1611	1	1	0.55	0.47	22	12	3UTR
hsa-miR-11400	NM_001895	CSNK2A1	3676	3702	0.961538	1	-0.30	0.01	26	10	3UTR
hsa-miR-11400	NM_177559	CSNK2A1	3793	3819	1	1	-0.15	-0.08	26	10	3UTR
hsa-miR-11400	XM_005246865	CSRNP3	9705	9741	1	1	0.00	0.00	24	9	3UTR
hsa-miR-11400	NM_024969	CSRNP3	9882	9918	1	1	0.95	0.64	24	9	3UTR
hsa-miR-11400	NM_001172173	CSRNP3	9932	9968	1	1	0.95	0.64	24	9	3UTR
hsa-miR-11400	NM_015235	CSTF2T	3485	3501	1	1	1.88	3.26	16	15	3UTR
hsa-miR-11400	NM_001083914	CTBP2	6309	6331	0.961538	1	4.77	5.53	22	12	3UTR
hsa-miR-11400	NM_022802	CTBP2	7832	7854	1	1	2.01	1.97	22	12	3UTR

mirnaid	refseqid	genesymbol	start	end	bindingp	seed	phylopstem	phylopflank	binding_region_length	longest_consecutive_pairings	position
hsa-miR-11400	XM_011524010	CTC1	4161	4179	0.991453	1	0.00	0.00	18	15	3UTR
hsa-miR-11400	XM_011524010	CTC1	6416	6439	1	1	0.00	0.00	23	12	3UTR
hsa-miR-11400	NM_025099	CTC1	4243	4261	1	1	-0.36	0.74	18	15	3UTR
hsa-miR-11400	NM_001269055	CTCFL	1627	1652	0.961538	1	4.52	4.07	25	8	3UTR
hsa-miR-11400	NM_016396	CTDSPL2	2670	2692	1	1	1.89	1.89	22	12	3UTR
hsa-miR-11400	NM_005214	CTLA4	1132	1150	1	1	-0.13	0.30	18	8	3UTR
hsa-miR-11400	NM_001037631	CTLA4	1022	1040	1	1	-0.13	0.30	18	8	3UTR
hsa-miR-11400	NM_001290307	CTNNA1	2981	3004	1	1	1.00	0.45	23	8	3UTR
hsa-miR-11400	NM_001290312	CTNNA1	2269	2292	1	1	1.04	0.84	23	8	3UTR
hsa-miR-11400	NM_001903	CTNNA1	2910	2933	1	1	1.04	0.84	23	8	3UTR
hsa-miR-11400	NM_148170	CTSC	3562	3583	1	1	0.09	-0.08	21	8	3UTR
hsa-miR-11400	NM_001114173	CTSC	3518	3539	1	1	-0.15	0.30	21	8	3UTR
hsa-miR-11400	NM_000396	CTSK	1258	1300	1	1	0.84	1.59	25	7	3UTR
hsa-miR-11400	NM_001199739	CTSS	1207	1225	0.974359	1	0.00	0.00	18	10	3UTR
hsa-miR-11400	NM_001352888	CTXND1	4760	4795	1	1	0.08	0.39	19	8	3UTR
hsa-miR-11400	NM_001081	CUBN	11008	11026	1	1	3.88	4.01	18	17	3UTR
hsa-miR-11400	NM_001257197	CUL3	4988	5010	0.961538	1	6.62	5.89	22	8	3UTR

mirnaid	refseqid	genesymbol	start	end	bindingp	seed	phylopstem	phylopflank	binding_region_length	longest_consecutive_pairings	position
hsa-miR-11400	NM_001257198	CUL3	5033	5055	0.961538	1	6.10	5.47	22	8	3UTR
hsa-miR-11400	NM_003590	CUL3	5186	5208	0.961538	1	5.97	5.82	22	8	3UTR
hsa-miR-11400	NM_001257197	CUL3	6228	6257	1	1	0.71	1.18	29	10	3UTR
hsa-miR-11400	NM_001257198	CUL3	6273	6302	1	1	5.98	4.95	29	10	3UTR
hsa-miR-11400	NM_003590	CUL3	6426	6455	1	1	0.71	1.18	29	10	3UTR
hsa-miR-11400	NM_001207064	CXADR	1502	1521	1	1	0.51	0.38	19	7	3UTR
hsa-miR-11400	NM_001207065	CXADR	1297	1316	1	1	0.79	0.35	19	7	3UTR
hsa-miR-11400	NM_001207066	CXADR	1238	1261	1	1	0.39	-0.09	23	11	3UTR
hsa-miR-11400	NM_001511	CXCL1	469	491	1	1	-0.22	-0.45	17	9	3UTR
hsa-miR-11400	NM_001277990	CXCL12	2457	2475	1	1	0.59	1.37	18	11	3UTR
hsa-miR-11400	NM_000609	CXCL12	2867	2885	1	1	0.10	0.56	18	11	3UTR
hsa-miR-11400	NM_002089	CXCL2	466	488	1	1	-0.97	-0.17	17	9	3UTR
hsa-miR-11400	XM_005264809	CXCR6	1489	1525	1	1	0.00	0.00	18	8	3UTR
hsa-miR-11400	XM_011533291	CXCR6	1965	2001	1	1	0.00	0.00	18	8	3UTR
hsa-miR-11400	NM_006564	CXCR6	1432	1468	1	1	-0.15	-0.19	18	8	3UTR
hsa-miR-11400	NM_144970	CXorf38	1461	1486	1	1	0.06	0.08	25	8	3UTR
hsa-miR-11400	XM_017029304	CXorf38	2629	2654	1	1	0.00	0.00	25	8	3UTR

mirnaid	refseqid	genesymbol	start	end	bindingp	seed	phylopstem	phylopflank	binding_region_length	longest_consecutive_pairings	position
hsa-miR-11400	NM_007326	CYB5R3	1225	1243	1	1	0.00	0.00	18	8	3UTR
hsa-miR-11400	NM_001129819	CYB5R3	1470	1488	1	1	0.00	0.00	18	8	3UTR
hsa-miR-11400	NM_001171660	CYB5R3	1358	1376	1	1	-0.06	-0.45	18	8	3UTR
hsa-miR-11400	NM_001171661	CYB5R3	1286	1304	1	1	0.00	0.00	18	8	3UTR
hsa-miR-11400	NM_001319216	CYP1A1	2036	2073	1	1	1.73	3.33	37	12	3UTR
hsa-miR-11400	NM_001319216	CYP1A1	2229	2272	1	1	2.36	1.85	43	9	3UTR
hsa-miR-11400	NM_001319217	CYP1A1	2123	2160	1	1	1.73	3.33	37	12	3UTR
hsa-miR-11400	NM_001319217	CYP1A1	2316	2359	1	1	2.36	1.85	43	9	3UTR
hsa-miR-11400	NM_000499	CYP1A1	2120	2157	1	1	1.69	3.24	37	12	3UTR
hsa-miR-11400	NM_000499	CYP1A1	2313	2356	1	1	2.35	1.97	43	9	3UTR
hsa-miR-11400	NM_001367502	CYP27C1	4097	4116	1	1	1.86	2.36	19	12	3UTR
hsa-miR-11400	NM_001001665	CYP27C1	3704	3723	1	1	2.72	3.11	19	12	3UTR
hsa-miR-11400	NM_183075	CYP2U1	1902	1919	1	1	-0.20	-0.38	17	9	3UTR
hsa-miR-11400	NM_001082	CYP4F2	2006	2053	1	1	0.47	0.55	18	13	3UTR
hsa-miR-11400	NM_004820	CYP7B1	5216	5233	1	1	1.78	1.29	17	16	3UTR
hsa-miR-11400	NM_001305630	CYREN	1064	1083	1	1	0.41	0.26	19	8	3UTR
hsa-miR-11400	NM_030797	CYRIA	1915	1955	0.974359	1	-0.14	0.03	40	11	3UTR

mirnaid	refseqid	genesymbol	start	end	bindingp	seed	phylopstem	phylopflank	binding_region_length	longest_consecutive_pairings	position
hsa-miR-11400	NM_030797	CYRIA	2057	2070	1	1	0.15	0.03	13	12	3UTR
hsa-miR-11400	NM_018659	CYTL1	658	670	1	1	2.67	1.99	12	11	3UTR
hsa-miR-11400	NM_015345	DAAM2	3745	3789	1	1	-0.29	-0.10	22	7	3UTR
hsa-miR-11400	NM_001201427	DAAM2	3697	3718	1	1	-0.26	-0.07	21	7	3UTR
hsa-miR-11400	NM_152654	DAND5	1512	1534	1	1	0.13	0.04	22	7	3UTR
hsa-miR-11400	NM_152654	DAND5	757	776	1	1	-0.39	-0.11	19	13	3UTR
hsa-miR-11400	NM_001363730	DAPK2	3329	3348	1	1	0.11	0.03	19	8	3UTR
hsa-miR-11400	NM_001375658	DAPK3	1840	1867	1	1	3.75	4.79	27	10	3UTR
hsa-miR-11400	NM_001017920	DAPL1	393	403	1	1	0.84	1.97	10	9	3UTR
hsa-miR-11400	NM_024043	DBNDD1	1908	1927	1	1	0.07	0.24	19	8	3UTR
hsa-miR-11400	NM_024043	DBNDD1	1134	1151	1	1	-0.05	-0.24	17	9	3UTR
hsa-miR-11400	NM_001371581	DBNDD1	902	919	1	1	-0.19	-0.16	17	9	3UTR
hsa-miR-11400	NM_001042610	DBNDD1	1889	1908	1	1	0.16	0.36	19	8	3UTR
hsa-miR-11400	NM_001918	DBT	4685	4703	1	1	0.17	0.09	18	14	3UTR
hsa-miR-11400	NM_001918	DBT	4635	4652	1	1	0.10	0.09	17	10	3UTR
hsa-miR-11400	NM_017741	DCAF16	2017	2036	1	1	-0.07	-0.13	19	9	3UTR
hsa-miR-11400	NM_025000	DCAF17	3952	3998	1	1	0.18	0.29	22	9	3UTR

mirnaid	refseqid	genesymbol	start	end	bindingp	seed	phylopstem	phylopflank	binding_region_length	longest_consecutive_pairings	position
hsa-miR-11400	NM_001289076	DCLRE1C	2066	2084	1	1	-0.04	0.03	18	17	3UTR
hsa-miR-11400	NM_001289076	DCLRE1C	4238	4264	1	1	4.18	3.51	26	11	3UTR
hsa-miR-11400	NM_001289077	DCLRE1C	2338	2356	1	1	0.04	-0.15	18	17	3UTR
hsa-miR-11400	NM_001289077	DCLRE1C	4510	4536	1	1	3.21	0.53	26	11	3UTR
hsa-miR-11400	NM_001289078	DCLRE1C	2099	2117	1	1	-0.24	0.18	18	17	3UTR
hsa-miR-11400	NM_001289078	DCLRE1C	4271	4297	1	1	4.07	3.12	26	11	3UTR
hsa-miR-11400	NM_001289079	DCLRE1C	2660	2678	1	1	-0.41	0.03	18	17	3UTR
hsa-miR-11400	NM_001289079	DCLRE1C	4832	4858	1	1	2.89	3.17	26	11	3UTR
hsa-miR-11400	NM_022487	DCLRE1C	2151	2169	1	1	0.07	0.27	18	17	3UTR
hsa-miR-11400	NM_022487	DCLRE1C	4323	4349	1	1	2.17	2.39	26	11	3UTR
hsa-miR-11400	NM_001033855	DCLRE1C	2207	2225	1	1	-0.46	0.06	18	17	3UTR
hsa-miR-11400	NM_001033855	DCLRE1C	4379	4405	1	1	1.47	1.07	26	11	3UTR
hsa-miR-11400	NM_001033857	DCLRE1C	2292	2310	1	1	-0.09	0.01	18	17	3UTR
hsa-miR-11400	NM_001033858	DCLRE1C	2614	2632	1	1	0.21	-0.18	18	17	3UTR
hsa-miR-11400	NM_133507	DCN	425	443	1	1	0.07	-0.02	18	8	3UTR
hsa-miR-11400	NM_001290204	DCP1A	4613	4636	1	1	2.82	1.91	23	8	3UTR
hsa-miR-11400	NM_018403	DCP1A	4727	4750	1	1	2.05	2.48	23	8	3UTR

mirnaid	refseqid	genesymbol	start	end	bindingp	seed	phylopstem	phylopflank	binding_region_length	longest_consecutive_pairings	position
hsa-miR-11400	NM_014026	DCPS	2780	2801	1	1	-0.42	0.00	21	12	3UTR
hsa-miR-11400	NM_004082	DCTN1	4168	4180	1	1	3.45	2.26	12	11	3UTR
hsa-miR-11400	NM_001135040	DCTN1	4093	4105	1	1	0.00	0.00	12	11	3UTR
hsa-miR-11400	NM_001135041	DCTN1	3792	3804	1	1	0.00	0.00	12	11	3UTR
hsa-miR-11400	NM_001190837	DCTN1	4147	4159	1	1	3.45	2.26	12	11	3UTR
hsa-miR-11400	NM_032486	DCTN5	4480	4499	1	1	0.07	0.36	19	9	3UTR
hsa-miR-11400	NM_001199743	DCTN5	4510	4529	1	1	0.00	0.00	19	9	3UTR
hsa-miR-11400	NM_001199743	DCTN5	1979	1998	1	1	0.00	0.00	19	7	3UTR
hsa-miR-11400	NM_020640	DCUN1D1	4928	4967	0.969231	1	4.06	3.19	14	12	3UTR
hsa-miR-11400	NM_001308101	DCUN1D1	4974	5013	1	1	2.04	2.55	14	12	3UTR
hsa-miR-11400	NM_178153	DCX	4221	4263	1	1	0.39	0.31	42	11	3UTR
hsa-miR-11400	NM_001369370	DCX	4312	4354	1	1	0.34	0.30	42	11	3UTR
hsa-miR-11400	NM_001369372	DCX	4224	4266	1	1	0.42	0.36	42	11	3UTR
hsa-miR-11400	NM_012137	DDAH1	1674	1691	1	1	0.27	0.60	17	13	3UTR
hsa-miR-11400	NM_001134445	DDAH1	1590	1607	1	1	0.94	1.00	17	13	3UTR
hsa-miR-11400	NM_001242890	DDC	1306	1328	1	1	0.00	0.00	22	11	3UTR
hsa-miR-11400	NM_004032	DDO	1652	1673	0.961538	1	1.96	3.23	21	9	3UTR

mirnaid	refseqid	genesymbol	start	end	bindingp	seed	phylopstem	phylopflank	binding_region_length	longest_consecutive_pairings	position
hsa-miR-11400	NM_001372108	DDO	1829	1850	0.961538	1	1.96	3.23	21	9	3UTR
hsa-miR-11400	NM_006182	DDR2	6600	6620	1	1	-0.13	-0.02	20	10	3UTR
hsa-miR-11400	NM_001354983	DDR2	7017	7037	1	1	-0.13	-0.02	20	10	3UTR
hsa-miR-11400	NM_001014796	DDR2	6680	6700	1	1	-0.13	-0.02	20	10	3UTR
hsa-miR-11400	NM_020414	DDX24	3736	3761	0.961538	1	5.26	4.83	25	9	3UTR
hsa-miR-11400	NM_013264	DDX25	2933	2948	1	1	-0.40	-0.12	15	8	3UTR
hsa-miR-11400	NM_001330438	DDX25	2940	2978	1	1	-0.22	-0.30	16	8	3UTR
hsa-miR-11400	NM_201224	DDX47	1309	1329	1	1	-0.32	-0.13	20	11	3UTR
hsa-miR-11400	NM_016355	DDX47	1456	1476	1	1	-0.32	-0.13	20	11	3UTR
hsa-miR-11400	NM_001242816	DEF8	1922	1943	1	1	1.47	3.05	21	13	3UTR
hsa-miR-11400	NM_207469	DEFB132	1122	1140	1	1	0.23	0.05	18	15	3UTR
hsa-miR-11400	XM_006714812	DELE1	2671	2692	1	1	0.00	0.00	21	7	3UTR
hsa-miR-11400	NM_198440	DERL3	2812	2836	1	1	5.04	4.21	24	7	3UTR
hsa-miR-11400	NM_001002862	DERL3	2711	2735	1	1	5.14	4.15	24	7	3UTR
hsa-miR-11400	NM_001366604	DERPC	2240	2267	1	1	0.75	1.07	20	11	3UTR
hsa-miR-11400	NM_004401	DFFA	5650	5669	1	1	2.91	1.97	19	7	3UTR
hsa-miR-11400	NM_005137	DGCR2	1997	2015	1	1	0.03	-0.34	18	9	3UTR

mirnaid	refseqid	genesymbol	start	end	bindingp	seed	phylopstem	phylopflank	binding_region_length	longest_consecutive_pairings	position
hsa-miR-11400	NM_001173533	DGCR2	1874	1892	1	1	-0.05	-0.06	18	9	3UTR
hsa-miR-11400	NM_001184781	DGCR2	1988	2006	1	1	-0.32	-0.36	18	9	3UTR
hsa-miR-11400	NM_022720	DGCR8	2761	2782	1	1	0.24	2.05	21	10	3UTR
hsa-miR-11400	NM_152910	DGKH	9522	9546	1	1	2.03	0.54	24	8	3UTR
hsa-miR-11400	NM_178009	DGKH	9653	9677	1	1	2.03	0.54	24	8	3UTR
hsa-miR-11400	NM_001204504	DGKH	9465	9489	1	1	0.00	0.00	24	8	3UTR
hsa-miR-11400	NM_001204506	DGKH	9235	9259	1	1	0.68	0.56	24	8	3UTR
hsa-miR-11400	XM_024448741	DGKZ	3242	3266	1	1	0.00	0.00	24	10	3UTR
hsa-miR-11400	NM_018706	DHTKD1	4194	4213	1	1	0.26	-0.09	19	11	3UTR
hsa-miR-11400	NM_198963	DHX57	4680	4701	1	1	2.06	1.55	21	7	3UTR
hsa-miR-11400	NM_173602	DIP2B	6965	6987	1	1	0.13	0.20	22	10	3UTR
hsa-miR-11400	NM_001252269	DIPK1A	1865	1876	1	1	0.00	0.00	11	10	3UTR
hsa-miR-11400	NM_001044369	DIPK1C	2900	2924	1	1	0.78	0.74	24	9	3UTR
hsa-miR-11400	NM_014953	DIS3	6998	7017	1	1	-0.05	0.18	19	10	3UTR
hsa-miR-11400	NM_001128226	DIS3	6908	6927	1	1	0.58	0.19	19	10	3UTR
hsa-miR-11400	NM_018662	DISC1	2723	2756	0.980769	1	0.35	0.03	19	12	3UTR
hsa-miR-11400	NM_001012957	DISC1	2657	2690	0.980769	1	0.35	0.03	19	12	3UTR

mirnaid	refseqid	genesymbol	start	end	bindingp	seed	phylopstem	phylopflank	binding_region_length	longest_consecutive_pairings	position
hsa-miR-11400	NM_001164540	DISC1	2346	2390	0.980769	1	0.10	0.12	19	12	3UTR
hsa-miR-11400	NM_001164546	DISC1	2265	2285	1	1	0.70	1.09	20	9	3UTR
hsa-miR-11400	NM_001288747	DKC1	2016	2055	1	1	0.09	1.29	17	8	3UTR
hsa-miR-11400	NM_014421	DKK2	2623	2659	1	1	3.73	4.67	20	14	3UTR
hsa-miR-11400	XM_005273810	DLG2	7104	7125	1	1	0.00	0.00	21	9	3UTR
hsa-miR-11400	NM_001206769	DLG2	7467	7488	1	1	0.00	0.00	21	9	3UTR
hsa-miR-11400	XM_006724626	DLG3	4039	4060	1	1	0.00	0.00	21	12	3UTR
hsa-miR-11400	NM_020730	DLG3	3060	3081	1	1	0.09	0.32	21	12	3UTR
hsa-miR-11400	NM_021120	DLG3	4012	4033	1	1	0.09	0.32	21	12	3UTR
hsa-miR-11400	NM_001166278	DLG3	2423	2444	1	1	1.22	0.90	21	12	3UTR
hsa-miR-11400	NM_001317172	DLK1	1148	1172	1	1	0.57	0.40	24	10	3UTR
hsa-miR-11400	NM_003836	DLK1	1367	1391	1	1	0.57	0.40	24	10	3UTR
hsa-miR-11400	NM_001286656	DLK2	1428	1455	1	1	0.44	0.16	22	9	3UTR
hsa-miR-11400	NM_206539	DLK2	1938	1965	1	1	0.30	-0.03	22	9	3UTR
hsa-miR-11400	NM_023932	DLK2	1446	1473	1	1	0.44	0.16	22	9	3UTR
hsa-miR-11400	XM_005267537	DMAC2L	4900	4920	1	1	0.00	0.00	20	11	3UTR
hsa-miR-11400	NM_001382507	DMAC2L	1632	1652	1	1	0.26	0.39	20	11	3UTR

mirnaid	refseqid	genesymbol	start	end	bindingp	seed	phylopstem	phylopflank	binding_region_length	longest_consecutive_pairings	position
hsa-miR-11400	NM_001003803	DMAC2L	2267	2287	1	1	0.26	0.39	20	11	3UTR
hsa-miR-11400	NM_001003805	DMAC2L	2095	2115	1	1	0.26	0.39	20	11	3UTR
hsa-miR-11400	NM_147192	DMBX1	2380	2399	0.974359	1	-0.38	-0.28	19	16	3UTR
hsa-miR-11400	NM_172225	DMBX1	2547	2566	0.974359	1	-0.38	-0.28	19	16	3UTR
hsa-miR-11400	NM_004407	DMP1	1862	1880	1	1	-0.07	0.27	18	11	3UTR
hsa-miR-11400	NM_001079911	DMP1	1814	1832	1	1	-0.07	0.27	18	11	3UTR
hsa-miR-11400	XM_017027125	DMRTC2	1391	1410	1	1	0.00	0.00	19	13	3UTR
hsa-miR-11400	NM_001302816	DMTN	2098	2121	1	1	-0.52	-0.30	23	12	3UTR
hsa-miR-11400	NM_001302817	DMTN	1728	1751	1	1	-0.52	-0.30	23	12	3UTR
hsa-miR-11400	NM_001978	DMTN	2164	2187	1	1	-0.52	-0.30	23	12	3UTR
hsa-miR-11400	NM_001114135	DMTN	1849	1872	1	1	-0.52	-0.30	23	12	3UTR
hsa-miR-11400	NM_001114136	DMTN	1715	1738	1	1	-0.52	-0.30	23	12	3UTR
hsa-miR-11400	NM_001114137	DMTN	1913	1936	1	1	-0.52	-0.30	23	12	3UTR
hsa-miR-11400	NM_001114138	DMTN	1965	1988	1	1	-0.52	-0.30	23	12	3UTR
hsa-miR-11400	NM_001114139	DMTN	1707	1730	1	1	-0.52	-0.30	23	12	3UTR
hsa-miR-11400	NM_001271581	DNAJC10	13804	13822	1	1	1.24	1.33	18	10	3UTR
hsa-miR-11400	NM_018981	DNAJC10	13942	13960	1	1	1.24	1.33	18	10	3UTR

mirnaid	refseqid	genesymbol	start	end	bindingp	seed	phylopstem	phylopflank	binding_region_length	longest_consecutive_pairings	position
hsa-miR-11400	NM_013238	DNAJC15	7124	7141	1	1	-0.38	-0.09	17	8	3UTR
hsa-miR-11400	NM_152686	DNAJC18	4445	4470	1	1	4.07	3.64	25	8	3UTR
hsa-miR-11400	NM_001144766	DNAJC7	1726	1747	1	1	0.51	2.29	21	9	3UTR
hsa-miR-11400	NM_031427	DNAL1	2354	2379	1	1	-0.18	-0.03	25	8	3UTR
hsa-miR-11400	NM_001375	DNASE2	1664	1689	1	1	1.66	1.02	25	8	3UTR
hsa-miR-11400	NM_001080849	DNLZ	1811	1832	1	1	0.11	0.16	21	15	3UTR
hsa-miR-11400	NM_024940	DOCK5	8347	8368	1	1	-0.23	0.17	21	12	3UTR
hsa-miR-11400	NM_203447	DOCK8	7070	7095	0.961538	1	0.15	-0.22	25	13	3UTR
hsa-miR-11400	XM_011518049	DOCK8	5630	5655	0.961538	1	0.00	0.00	25	13	3UTR
hsa-miR-11400	NM_001190458	DOCK8	6761	6786	0.961538	1	0.15	-0.22	25	13	3UTR
hsa-miR-11400	NM_001193536	DOCK8	6857	6882	0.961538	1	0.20	-0.20	25	13	3UTR
hsa-miR-11400	NM_152721	DOK6	1354	1375	1	1	1.00	2.02	21	9	3UTR
hsa-miR-11400	NM_130434	DPP8	4490	4515	0.961538	1	5.97	4.75	18	16	3UTR
hsa-miR-11400	NM_197960	DPP8	4491	4516	0.961538	1	4.82	4.82	18	16	3UTR
hsa-miR-11400	NM_197961	DPP8	4473	4498	1	1	3.51	4.44	18	16	3UTR
hsa-miR-11400	XM_017022378	DPP8	4460	4485	1	1	0.00	0.00	18	16	3UTR
hsa-miR-11400	NM_017743	DPP8	4190	4215	1	1	0.59	0.55	18	16	3UTR

mirnaid	refseqid	genesymbol	start	end	bindingp	seed	phylopstem	phylopflank	binding_region_length	longest_consecutive_pairings	position
hsa-miR-11400	NM_001365987	DPP9	2091	2109	1	1	0.64	0.86	18	7	3UTR
hsa-miR-11400	NM_001384637	DPP9	1913	1931	1	1	0.64	0.86	18	7	3UTR
hsa-miR-11400	NM_173812	DPY19L2	2816	2833	1	1	3.99	3.46	17	10	3UTR
hsa-miR-11400	NM_001253723	DPYSL5	4406	4435	1	1	0.51	0.53	22	9	3UTR
hsa-miR-11400	NM_001253723	DPYSL5	3040	3059	1	1	-0.71	-0.01	19	14	3UTR
hsa-miR-11400	NM_001253723	DPYSL5	5208	5224	1	1	0.00	0.00	16	15	3UTR
hsa-miR-11400	NM_001253724	DPYSL5	3178	3197	1	1	0.37	-0.24	19	14	3UTR
hsa-miR-11400	NM_020134	DPYSL5	2798	2817	1	1	0.42	0.03	19	14	3UTR
hsa-miR-11400	XM_005269005	DRAM1	1717	1742	1	1	0.00	0.00	25	9	3UTR
hsa-miR-11400	NM_001145315	DSN1	1960	1979	1	1	0.33	0.86	19	10	3UTR
hsa-miR-11400	NM_001145318	DSN1	1911	1930	1	1	0.76	0.52	19	10	3UTR
hsa-miR-11400	NM_006870	DSTN	1650	1676	1	1	0.84	1.66	26	16	3UTR
hsa-miR-11400	NM_006870	DSTN	2286	2315	1	1	0.27	0.08	21	12	3UTR
hsa-miR-11400	NM_001011546	DSTN	1782	1808	1	1	0.84	1.66	26	16	3UTR
hsa-miR-11400	NM_001011546	DSTN	2418	2447	1	1	0.27	0.08	21	12	3UTR
hsa-miR-11400	NM_001318043	DTD1	2620	2662	1	1	0.61	0.27	20	9	3UTR
hsa-miR-11400	NM_032978	DTNA	2161	2181	1	1	0.05	0.28	20	8	3UTR

mirnaid	refseqid	genesymbol	start	end	bindingp	seed	phylopstem	phylopflank	binding_region_length	longest_consecutive_pairings	position
hsa-miR-11400	NM_032979	DTNA	2179	2199	1	1	0.05	0.28	20	8	3UTR
hsa-miR-11400	NM_032981	DTNA	1232	1252	1	1	0.05	0.28	20	8	3UTR
hsa-miR-11400	NM_001198941	DTNA	1990	2010	1	1	0.05	0.28	20	8	3UTR
hsa-miR-11400	NM_001198945	DTNA	1587	1620	1	1	0.42	0.13	21	8	3UTR
hsa-miR-11400	NM_020234	DTWD1	10374	10393	1	1	0.11	0.30	19	10	3UTR
hsa-miR-11400	NM_001144955	DTWD1	10246	10265	1	1	0.11	0.30	19	10	3UTR
hsa-miR-11400	NM_001308081	DTWD2	5331	5381	0.974359	1	3.85	3.50	26	8	3UTR
hsa-miR-11400	NM_001276267	DUOXA1	1484	1505	1	1	0.13	-0.03	21	8	3UTR
hsa-miR-11400	NM_001276268	DUOXA1	1143	1164	1	1	0.52	0.43	21	8	3UTR
hsa-miR-11400	XM_006720751	DUOXA1	1304	1325	1	1	0.00	0.00	21	8	3UTR
hsa-miR-11400	NM_181581	DUS4L	1486	1506	1	1	0.01	0.10	20	10	3UTR
hsa-miR-11400	NM_001371366	DUS4L-BCAP29	3285	3303	1	1	0.00	0.00	18	10	3UTR
hsa-miR-11400	NM_001371367	DUS4L-BCAP29	3196	3214	1	1	0.00	0.00	18	10	3UTR
hsa-miR-11400	XM_017016314	DUSP13	805	825	1	1	0.00	0.00	20	9	3UTR
hsa-miR-11400	NM_001007271	DUSP13	868	889	1	1	0.55	1.20	21	9	3UTR
hsa-miR-11400	NM_024025	DUSP26	1635	1685	1	1	0.52	0.18	22	20	3UTR
hsa-miR-11400	NM_001947	DUSP7	3072	3104	1	1	0.64	0.47	32	9	3UTR

mirnaid	refseqid	genesymbol	start	end	bindingp	seed	phylopstem	phylopflank	binding_region_length	longest_consecutive_pairings	position
hsa-miR-11400	NM_004423	DVL3	3526	3552	1	1	0.25	0.29	26	15	3UTR
hsa-miR-11400	XM_005269550	DYDC1	2541	2566	1	1	0.00	0.00	25	8	3UTR
hsa-miR-11400	NM_080677	DYNLL2	860	881	1	1	0.46	0.96	21	8	3UTR
hsa-miR-11400	NM_080677	DYNLL2	3634	3654	1	1	0.19	0.10	20	11	3UTR
hsa-miR-11400	NM_006520	DYNLT3	1628	1658	1	1	0.52	1.03	18	16	3UTR
hsa-miR-11400	NM_001347721	DYRK1A	6477	6499	1	1	0.81	0.01	22	6	3UTR
hsa-miR-11400	NM_001347722	DYRK1A	5734	5756	1	1	0.81	0.01	22	6	3UTR
hsa-miR-11400	NM_001347723	DYRK1A	8374	8399	1	1	0.00	0.00	25	7	3UTR
hsa-miR-11400	NM_001949	E2F3	4378	4397	1	1	3.35	2.99	19	9	3UTR
hsa-miR-11400	NM_001243076	E2F3	3832	3851	1	1	3.33	3.10	19	9	3UTR
hsa-miR-11400	NM_001351378	EDC3	2112	2144	1	1	4.67	4.62	32	8	3UTR
hsa-miR-11400	NM_001142443	EDC3	3759	3778	1	1	-0.42	0.11	19	14	3UTR
hsa-miR-11400	NM_001142443	EDC3	2245	2277	1	1	5.12	4.84	32	8	3UTR
hsa-miR-11400	NM_001142444	EDC3	2030	2062	1	1	1.03	3.66	32	8	3UTR
hsa-miR-11400	NM_001955	EDN1	1717	1738	1	1	0.16	0.50	21	6	3UTR
hsa-miR-11400	NM_000115	EDNRB	2249	2268	1	1	0.18	0.04	19	11	3UTR
hsa-miR-11400	NM_000115	EDNRB	2564	2582	1	1	1.00	0.47	18	12	3UTR

mirnaid	refseqid	genesymbol	start	end	bindingp	seed	phylopstem	phylopflank	binding_region_length	longest_consecutive_pairings	position
hsa-miR-11400	NM_001122659	EDNRB	2275	2294	1	1	0.66	0.07	19	11	3UTR
hsa-miR-11400	NM_001122659	EDNRB	2590	2608	1	1	-0.01	0.76	18	12	3UTR
hsa-miR-11400	NM_001201397	EDNRB	2440	2459	1	1	1.09	1.38	19	11	3UTR
hsa-miR-11400	NM_001201397	EDNRB	2755	2773	1	1	4.83	4.43	18	12	3UTR
hsa-miR-11400	NM_001355529	EFCAB10	754	776	0.987179	1	0.53	1.35	22	7	3UTR
hsa-miR-11400	NM_001962	EFNA5	1941	1973	1	1	1.27	1.83	32	9	3UTR
hsa-miR-11400	NM_001277174	EFS	1469	1503	1	1	1.98	1.03	34	10	3UTR
hsa-miR-11400	NM_032459	EFS	1697	1731	1	1	0.80	0.64	34	10	3UTR
hsa-miR-11400	NM_001357021	EGF	5489	5507	1	1	-0.17	0.01	18	17	3UTR
hsa-miR-11400	NM_001178131	EGF	5811	5829	1	1	-0.17	0.01	18	17	3UTR
hsa-miR-11400	NM_001282444	EHD1	4360	4381	1	1	-0.31	-0.53	21	11	3UTR
hsa-miR-11400	NM_001282445	EHD1	4038	4059	1	1	0.00	0.00	21	11	3UTR
hsa-miR-11400	XM_011519025	EHMT1	4583	4601	1	1	0.00	0.00	18	17	3UTR
hsa-miR-11400	NM_005801	EIF1	2185	2205	1	1	0.05	-0.03	20	8	3UTR
hsa-miR-11400	NM_005801	EIF1	1367	1387	1	1	-0.16	-0.26	20	6	3UTR
hsa-miR-11400	NM_002759	EIF2AK2	3941	3962	1	1	1.20	1.43	21	8	3UTR
hsa-miR-11400	NM_003751	EIF3B	2825	2856	1	1	0.13	0.13	24	12	3UTR

mirnaid	refseqid	genesymbol	start	end	bindingp	seed	phylopstem	phylopflank	binding_region_length	longest_consecutive_pairings	position
hsa-miR-11400	NM_001037283	EIF3B	2900	2931	1	1	0.13	0.13	24	12	3UTR
hsa-miR-11400	NM_003754	EIF3F	1179	1199	1	1	0.27	0.83	20	6	3UTR
hsa-miR-11400	NM_001330202	EIF4E2	1011	1037	0.961538	1	6.78	4.98	26	8	3UTR
hsa-miR-11400	NM_004846	EIF4E2	915	941	1	1	1.42	2.42	26	8	3UTR
hsa-miR-11400	NM_001330201	EIF4E2	780	806	1	1	2.44	2.41	26	8	3UTR
hsa-miR-11400	NM_001134651	EIF4E3	8676	8694	1	1	0.67	0.66	18	8	3UTR
hsa-miR-11400	NM_001291157	EIF4G1	5232	5261	1	1	3.09	1.89	29	15	3UTR
hsa-miR-11400	NM_182917	EIF4G1	5431	5460	1	1	3.09	1.89	29	15	3UTR
hsa-miR-11400	NM_198241	EIF4G1	5172	5201	1	1	3.09	1.89	29	15	3UTR
hsa-miR-11400	NM_198242	EIF4G1	4757	4786	1	1	3.09	1.89	29	15	3UTR
hsa-miR-11400	NM_198244	EIF4G1	4934	4963	1	1	3.09	1.89	29	15	3UTR
hsa-miR-11400	NM_004953	EIF4G1	4845	4874	1	1	3.09	1.89	29	15	3UTR
hsa-miR-11400	NM_001194946	EIF4G1	5193	5222	1	1	3.09	1.89	29	15	3UTR
hsa-miR-11400	NM_018696	ELAC1	1937	1970	1	1	0.47	0.30	18	12	3UTR
hsa-miR-11400	NM_152748	ELAPOR2	2900	2921	1	1	0.50	0.09	21	8	3UTR
hsa-miR-11400	XM_011515921	ELAPOR2	3252	3273	1	1	0.00	0.00	21	8	3UTR
hsa-miR-11400	NM_001257168	ELK1	900	919	1	1	0.00	0.00	19	8	3UTR

mirnaid	refseqid	genesymbol	start	end	bindingp	seed	phylopstem	phylopflank	binding_region_length	longest_consecutive_pairings	position
hsa-miR-11400	NM_005229	ELK1	1672	1691	1	1	0.00	0.00	19	8	3UTR
hsa-miR-11400	NM_001114123	ELK1	1778	1797	1	1	0.00	0.00	19	8	3UTR
hsa-miR-11400	NM_130442	ELMO1	2203	2237	1	1	1.50	0.44	15	13	3UTR
hsa-miR-11400	NM_133171	ELMO2	3599	3614	1	1	-0.25	0.01	15	14	3UTR
hsa-miR-11400	NM_182764	ELMO2	3495	3510	1	1	1.71	2.67	15	14	3UTR
hsa-miR-11400	NM_001318253	ELMO2	3667	3682	1	1	-0.25	0.01	15	14	3UTR
hsa-miR-11400	NM_153702	ELMOD2	1531	1550	1	1	-0.46	0.01	19	11	3UTR
hsa-miR-11400	NM_153702	ELMOD2	3946	3962	1	1	0.81	0.13	16	7	3UTR
hsa-miR-11400	NM_003198	ELOA	3903	3921	1	1	-0.83	-0.07	18	9	3UTR
hsa-miR-11400	NM_017770	ELOVL2	3901	3923	1	1	1.23	1.56	22	5	3UTR
hsa-miR-11400	NM_001297617	ELOVL7	2437	2460	1	1	0.49	0.62	23	10	3UTR
hsa-miR-11400	NM_024930	ELOVL7	2090	2113	1	1	0.24	0.46	23	10	3UTR
hsa-miR-11400	NM_001104558	ELOVL7	2039	2062	1	1	0.56	0.32	23	10	3UTR
hsa-miR-11400	NM_018255	ELP2	7537	7563	1	1	0.05	-0.05	26	8	3UTR
hsa-miR-11400	NM_001242875	ELP2	7732	7758	1	1	0.00	0.00	26	8	3UTR
hsa-miR-11400	NM_001242876	ELP2	7522	7548	1	1	0.00	0.00	26	8	3UTR
hsa-miR-11400	NM_001242877	ELP2	7459	7485	1	1	0.00	0.00	26	8	3UTR

mirnaid	refseqid	genesymbol	start	end	bindingp	seed	phylopstem	phylopflank	binding_region_length	longest_consecutive_pairings	position
hsa-miR-11400	NM_001242878	ELP2	7327	7353	1	1	0.25	0.09	26	8	3UTR
hsa-miR-11400	NM_001242879	ELP2	7327	7353	1	1	0.00	0.00	26	8	3UTR
hsa-miR-11400	NM_001142288	EMC8	651	689	1	1	0.20	-0.05	38	10	3UTR
hsa-miR-11400	NM_016242	EMCN	3099	3124	1	1	2.01	2.10	20	10	3UTR
hsa-miR-11400	NM_001159694	EMCN	3060	3085	1	1	1.51	1.51	20	10	3UTR
hsa-miR-11400	NM_133455	EMID1	1724	1744	0.980769	1	-0.29	-0.22	20	11	3UTR
hsa-miR-11400	XM_005261328	EMID1	1712	1732	0.980769	1	0.00	0.00	20	11	3UTR
hsa-miR-11400	NM_133455	EMID1	2026	2048	1	1	0.00	-0.20	22	10	3UTR
hsa-miR-11400	XM_005261328	EMID1	2014	2036	1	1	0.00	0.00	22	10	3UTR
hsa-miR-11400	XM_011536540	EML1	3203	3235	1	1	0.00	0.00	18	13	3UTR
hsa-miR-11400	NM_004434	EML1	3140	3157	1	1	0.41	-0.04	17	13	3UTR
hsa-miR-11400	NM_001008707	EML1	3197	3214	1	1	0.41	-0.04	17	13	3UTR
hsa-miR-11400	XM_024448315	ENAH	8459	8480	1	1	0.00	0.00	21	9	3UTR
hsa-miR-11400	NM_001008493	ENAH	7801	7822	1	1	0.18	-0.01	21	9	3UTR
hsa-miR-11400	NM_015036	ENDOD1	3396	3418	1	1	0.25	0.19	22	9	3UTR
hsa-miR-11400	NM_001977	ENPEP	6512	6534	1	1	-0.04	0.27	22	14	3UTR
hsa-miR-11400	NM_207045	ENSA	929	952	1	1	1.68	0.90	23	7	3UTR

mirnaid	refseqid	genesymbol	start	end	bindingp	seed	phylopstem	phylopflank	binding_region_length	longest_consecutive_pairings	position
hsa-miR-11400	NM_207046	ENSA	881	904	1	1	0.97	1.56	23	7	3UTR
hsa-miR-11400	NM_004436	ENSA	1001	1024	1	1	2.38	3.63	23	7	3UTR
hsa-miR-11400	NM_001128930	ENTPD4	2351	2376	1	1	1.09	0.11	25	6	3UTR
hsa-miR-11400	XM_024452480	EOLA1	2598	2620	1	1	0.00	0.00	22	7	3UTR
hsa-miR-11400	XM_024452480	EOLA1	3544	3560	1	1	0.00	0.00	16	15	3UTR
hsa-miR-11400	NM_001324276	EOLA1	3579	3595	1	1	0.02	-0.17	16	15	3UTR
hsa-miR-11400	NM_001171909	EOLA1	3545	3561	1	1	0.02	-0.17	16	15	3UTR
hsa-miR-11400	XM_011531181	EOLA2	2605	2627	1	1	0.00	0.00	22	7	3UTR
hsa-miR-11400	XM_011531181	EOLA2	3551	3567	1	1	0.00	0.00	16	15	3UTR
hsa-miR-11400	NM_005233	EPHA3	3524	3559	1	1	0.81	0.34	35	9	3UTR
hsa-miR-11400	NM_001363748	EPHA4	2984	3032	1	1	0.95	1.44	23	11	3UTR
hsa-miR-11400	NM_001309193	EPHB2	9451	9473	1	1	0.09	0.16	22	8	3UTR
hsa-miR-11400	NM_017449	EPHB2	9544	9566	1	1	0.09	0.16	22	8	3UTR
hsa-miR-11400	NM_004442	EPHB2	9548	9570	1	1	0.09	0.16	22	8	3UTR
hsa-miR-11400	XM_011536116	EPM2A	2160	2203	1	1	0.00	0.00	17	8	3UTR
hsa-miR-11400	NM_001360057	EPM2A	2234	2250	1	1	3.34	3.80	16	8	3UTR
hsa-miR-11400	NM_014805	EPM2AIP1	4049	4068	0.961538	1	0.27	1.02	19	13	3UTR

mirnaid	refseqid	genesymbol	start	end	bindingp	seed	phylopstem	phylopflank	binding_region_length	longest_consecutive_pairings	position
hsa-miR-11400	NM_014805	EPM2AIP1	6352	6406	1	1	2.12	1.56	22	11	3UTR
hsa-miR-11400	NM_001042599	ERBB4	4097	4121	1	1	1.82	0.85	24	14	3UTR
hsa-miR-11400	NM_020207	ERCC6L2	9925	9946	0.972222	1	-0.06	-0.08	21	12	3UTR
hsa-miR-11400	NM_182918	ERG	2608	2634	1	1	0.39	0.28	26	6	3UTR
hsa-miR-11400	NM_004449	ERG	4856	4875	1	1	0.48	0.64	19	10	3UTR
hsa-miR-11400	NM_001331025	ERG	2536	2562	1	1	-0.13	0.42	26	6	3UTR
hsa-miR-11400	NM_001136154	ERG	4928	4947	1	1	0.48	0.64	19	10	3UTR
hsa-miR-11400	NM_001243428	ERG	4951	4970	1	1	0.22	0.65	19	10	3UTR
hsa-miR-11400	NM_001243429	ERG	4515	4534	1	1	2.08	3.11	19	10	3UTR
hsa-miR-11400	NM_001100626	ERLIN1	1796	1813	1	1	2.01	2.01	17	10	3UTR
hsa-miR-11400	NM_001433	ERN1	3188	3207	1	1	-0.08	0.06	19	11	3UTR
hsa-miR-11400	NM_001034025	ERP29	479	501	1	1	1.44	2.31	22	6	3UTR
hsa-miR-11400	NM_015051	ERP44	2105	2126	1	1	1.55	1.64	21	12	3UTR
hsa-miR-11400	NM_018948	ERRFI1	2749	2773	1	1	1.47	1.94	24	10	3UTR
hsa-miR-11400	NM_194312	ESPNL	4392	4415	1	1	1.82	1.60	23	9	3UTR
hsa-miR-11400	NM_000125	ESR1	3091	3126	1	1	-0.18	-0.12	24	8	3UTR
hsa-miR-11400	NM_001122741	ESR1	3046	3081	1	1	-0.18	-0.12	24	8	3UTR

mirnaid	refseqid	genesymbol	start	end	bindingp	seed	phylopstem	phylopflank	binding_region_length	longest_consecutive_pairings	position
hsa-miR-11400	NM_001122742	ESR1	3199	3234	1	1	-0.18	-0.12	24	8	3UTR
hsa-miR-11400	NM_001271877	ESR2	1435	1455	0.980769	1	1.74	2.35	20	14	3UTR
hsa-miR-11400	NM_024939	ESRP2	2713	2733	1	1	2.78	2.44	20	7	3UTR
hsa-miR-11400	NM_001365264	ESRP2	2743	2763	1	1	2.78	2.44	20	7	3UTR
hsa-miR-11400	NM_001282450	ESRRA	2489	2508	1	1	0.00	0.00	19	10	3UTR
hsa-miR-11400	NM_001282451	ESRRA	2128	2147	1	1	1.90	2.51	19	10	3UTR
hsa-miR-11400	NM_004451	ESRRA	2131	2150	1	1	2.32	2.36	19	10	3UTR
hsa-miR-11400	NM_001379180	ESRRB	1821	1844	1	1	1.42	1.07	23	11	3UTR
hsa-miR-11400	NM_001243518	ESRRG	2937	2958	1	1	3.65	3.11	21	8	3UTR
hsa-miR-11400	NM_001243519	ESRRG	3087	3108	1	1	3.43	3.35	21	8	3UTR
hsa-miR-11400	NM_031913	ESYT3	4315	4336	1	1	0.02	0.33	21	12	3UTR
hsa-miR-11400	NM_001256302	ETF1	3801	3822	1	1	3.86	2.76	21	8	3UTR
hsa-miR-11400	NM_001282185	ETF1	3551	3572	1	1	0.50	0.31	21	8	3UTR
hsa-miR-11400	NM_004730	ETF1	3602	3623	1	1	3.86	2.76	21	8	3UTR
hsa-miR-11400	NM_005238	ETS1	3098	3120	1	1	0.00	0.00	22	8	3UTR
hsa-miR-11400	NM_001261439	ETV4	1224	1245	1	1	3.24	2.65	21	7	3UTR
hsa-miR-11400	NM_016135	ETV7	1266	1282	1	1	1.03	2.61	16	15	3UTR

mirnaid	refseqid	genesymbol	start	end	bindingp	seed	phylopstem	phylopflank	binding_region_length	longest_consecutive_pairings	position
hsa-miR-11400	NM_001207036	ETV7	1101	1117	1	1	0.00	0.00	16	15	3UTR
hsa-miR-11400	NM_001207037	ETV7	1307	1323	1	1	2.69	2.28	16	15	3UTR
hsa-miR-11400	NM_001207040	ETV7	1130	1146	1	1	1.36	1.25	16	15	3UTR
hsa-miR-11400	NM_001207041	ETV7	965	981	1	1	0.22	0.18	16	15	3UTR
hsa-miR-11400	NM_001308248	EVI5	5182	5204	1	1	4.03	3.53	17	11	3UTR
hsa-miR-11400	NM_005665	EVI5	5149	5171	1	1	2.40	2.99	17	11	3UTR
hsa-miR-11400	XM_005267272	EXOC5	2544	2572	1	1	0.00	0.00	28	8	3UTR
hsa-miR-11400	NM_006544	EXOC5	2465	2493	1	1	0.16	0.65	28	8	3UTR
hsa-miR-11400	NM_001282313	EXOC7	2586	2604	1	1	0.66	0.33	18	6	3UTR
hsa-miR-11400	NM_001308019	EXPH5	6837	6853	1	1	0.79	-0.09	16	15	3UTR
hsa-miR-11400	NM_015065	EXPH5	7055	7071	1	1	-0.31	0.60	16	15	3UTR
hsa-miR-11400	NM_001144763	EXPH5	6914	6930	1	1	1.30	0.05	16	15	3UTR
hsa-miR-11400	XM_017024350	EZH1	2909	2927	1	1	0.00	0.00	18	12	3UTR
hsa-miR-11400	NM_001991	EZH1	3026	3044	1	1	3.21	4.16	18	12	3UTR
hsa-miR-11400	NM_003379	EZR	2404	2444	0.961538	1	4.71	4.17	40	10	3UTR
hsa-miR-11400	NM_001111077	EZR	2387	2427	0.961538	1	5.19	5.03	40	10	3UTR
hsa-miR-11400	NM_001312675	F10	1180	1217	1	1	2.75	2.82	37	8	3UTR

mirnaid	refseqid	genesymbol	start	end	bindingp	seed	phylopstem	phylopflank	binding_region_length	longest_consecutive_pairings	position
hsa-miR-11400	NM_005242	F2RL1	2121	2146	0.961538	1	4.72	4.43	25	8	3UTR
hsa-miR-11400	NM_000130	F5	7754	7773	1	1	3.78	2.65	19	6	3UTR
hsa-miR-11400	NM_001267554	F7	1718	1744	1	1	-0.24	-0.33	26	15	3UTR
hsa-miR-11400	NM_001267554	F7	1832	1866	1	1	-0.02	-0.33	25	15	3UTR
hsa-miR-11400	NM_019616	F7	1904	1930	1	1	-0.18	-0.33	26	15	3UTR
hsa-miR-11400	NM_019616	F7	2028	2052	1	1	-0.17	-0.43	24	15	3UTR
hsa-miR-11400	NM_000131	F7	1973	1999	1	1	-0.18	-0.33	26	15	3UTR
hsa-miR-11400	NM_000131	F7	2097	2121	1	1	-0.17	-0.43	24	15	3UTR
hsa-miR-11400	NM_001256946	FAAP20	1256	1275	1	1	-0.10	-0.28	19	9	3UTR
hsa-miR-11400	NM_000134	FABP2	803	819	0.969231	1	0.35	-0.01	16	13	3UTR
hsa-miR-11400	XM_011545022	FADS1	1910	1931	1	1	0.00	0.00	21	8	3UTR
hsa-miR-11400	NM_013402	FADS1	2182	2203	1	1	0.14	0.12	21	8	3UTR
hsa-miR-11400	XM_017004236	FAHD2A	3003	3024	1	1	0.00	0.00	21	10	3UTR
hsa-miR-11400	XM_005268730	FAIM2	1356	1377	1	1	0.00	0.00	21	17	3UTR
hsa-miR-11400	NM_012306	FAIM2	1536	1557	1	1	-0.01	-0.07	21	17	3UTR
hsa-miR-11400	NM_001282713	FAM107A	2856	2872	1	1	0.76	3.16	16	15	3UTR
hsa-miR-11400	NM_007177	FAM107A	2880	2896	1	1	3.47	1.18	16	15	3UTR

mirnaid	refseqid	genesymbol	start	end	bindingp	seed	phylopstem	phylopflank	binding_region_length	longest_consecutive_pairings	position
hsa-miR-11400	NM_198947	FAM111B	3117	3150	1	1	0.07	0.00	21	9	3UTR
hsa-miR-11400	NM_001142703	FAM111B	2950	2983	1	1	0.07	0.00	21	9	3UTR
hsa-miR-11400	NM_001317994	FAM114A2	4161	4181	1	1	0.97	1.74	20	10	3UTR
hsa-miR-11400	NM_001317995	FAM114A2	3387	3407	1	1	2.60	2.49	20	10	3UTR
hsa-miR-11400	NM_001286379	FAM120B	4267	4285	1	1	-0.40	-0.16	18	17	3UTR
hsa-miR-11400	NM_001286380	FAM120B	4024	4048	1	1	-0.02	-0.30	19	17	3UTR
hsa-miR-11400	NM_001286381	FAM120B	2040	2064	1	1	0.42	-0.43	19	17	3UTR
hsa-miR-11400	NM_032448	FAM120B	3976	4000	1	1	-0.02	-0.30	19	17	3UTR
hsa-miR-11400	NM_024785	FAM124B	2019	2045	0.953846	1	2.97	2.35	21	12	3UTR
hsa-miR-11400	NM_001122779	FAM124B	1894	1920	0.953846	1	2.97	2.35	21	12	3UTR
hsa-miR-11400	NM_024785	FAM124B	2081	2104	1	1	3.28	1.69	23	10	3UTR
hsa-miR-11400	NM_001122779	FAM124B	1956	1979	1	1	3.28	1.69	23	10	3UTR
hsa-miR-11400	NM_032581	FAM126A	3142	3165	1	1	-0.06	0.36	23	10	3UTR
hsa-miR-11400	NM_001363466	FAM126A	3438	3461	1	1	0.19	0.15	23	10	3UTR
hsa-miR-11400	NM_173698	FAM133A	1598	1637	1	1	0.90	1.27	18	7	3UTR
hsa-miR-11400	NM_001171111	FAM133A	1529	1568	1	1	-0.09	0.30	18	7	3UTR
hsa-miR-11400	NM_001040057	FAM133B	1611	1627	1	1	1.40	3.00	16	8	3UTR

mirnaid	refseqid	genesymbol	start	end	bindingp	seed	phylopstem	phylopflank	binding_region_length	longest_consecutive_pairings	position
hsa-miR-11400	NM_001329755	FAM136A	1300	1323	1	1	0.94	1.80	23	9	3UTR
hsa-miR-11400	NM_001265578	FAM13A	2986	3010	1	1	2.29	3.66	24	7	3UTR
hsa-miR-11400	NM_001265579	FAM13A	2944	2968	1	1	2.75	3.20	24	7	3UTR
hsa-miR-11400	NM_001265580	FAM13A	2944	2968	1	1	2.75	3.20	24	7	3UTR
hsa-miR-11400	NM_014883	FAM13A	3999	4023	1	1	1.52	2.98	24	7	3UTR
hsa-miR-11400	NM_001015045	FAM13A	3028	3052	1	1	4.15	5.30	24	7	3UTR
hsa-miR-11400	NM_205548	FAM151B	1280	1326	1	1	-0.05	0.42	20	9	3UTR
hsa-miR-11400	XM_017009358	FAM153A	2322	2353	0.974359	1	0.00	0.00	19	11	3UTR
hsa-miR-11400	XM_011532224	FAM160A1	7275	7293	1	1	0.00	0.00	18	9	3UTR
hsa-miR-11400	NM_001109977	FAM160A1	7776	7794	1	1	0.00	-0.26	18	9	3UTR
hsa-miR-11400	NM_001371529	FAM163B	851	885	1	1	-1.45	-0.61	20	10	3UTR
hsa-miR-11400	NM_053279	FAM167A	2954	2973	1	1	4.73	4.67	19	8	3UTR
hsa-miR-11400	XM_011543837	FAM167A	1813	1833	1	1	0.00	0.00	20	12	3UTR
hsa-miR-11400	XM_011543837	FAM167A	3049	3068	1	1	0.00	0.00	19	8	3UTR
hsa-miR-11400	XM_011543840	FAM167A	1634	1654	1	1	0.00	0.00	20	12	3UTR
hsa-miR-11400	XM_011543840	FAM167A	2870	2889	1	1	0.00	0.00	19	8	3UTR
hsa-miR-11400	NM_182562	FAM169B	1249	1283	1	1	-0.07	-0.27	19	13	3UTR

mirnaid	refseqid	genesymbol	start	end	bindingp	seed	phylopstem	phylopflank	binding_region_length	longest_consecutive_pairings	position
hsa-miR-11400	NM_182562	FAM169B	1289	1323	1	1	-0.11	-0.24	19	13	3UTR
hsa-miR-11400	XM_017001280	FAM177B	2615	2634	1	1	0.00	0.00	19	9	3UTR
hsa-miR-11400	NM_001324080	FAM177B	1344	1363	1	1	0.00	-0.01	19	9	3UTR
hsa-miR-11400	NM_001101376	FAM183A	496	507	1	1	-0.09	-0.10	11	10	3UTR
hsa-miR-11400	NM_207318	FAM199X	1779	1791	1	1	0.20	0.72	12	11	3UTR
hsa-miR-11400	NM_001321920	FAM219B	683	698	1	1	0.30	-0.12	15	14	3UTR
hsa-miR-11400	NM_001321922	FAM219B	1220	1235	1	1	-0.22	-0.19	15	14	3UTR
hsa-miR-11400	NM_001013647	FAM227A	3966	3993	1	1	0.48	0.13	27	10	3UTR
hsa-miR-11400	NM_001384237	FAM237B	1868	1895	1	1	0.48	0.47	16	14	3UTR
hsa-miR-11400	NM_174951	FAM9A	1359	1394	1	1	0.00	0.00	21	8	3UTR
hsa-miR-11400	NM_001171186	FAM9A	1384	1419	1	1	0.00	0.00	21	8	3UTR
hsa-miR-11400	NM_001286839	FARP1	4503	4521	1	1	-0.02	-0.13	18	17	3UTR
hsa-miR-11400	XM_011521046	FARP1	4709	4727	1	1	0.00	0.00	18	17	3UTR
hsa-miR-11400	NM_005766	FARP1	4620	4638	1	1	-0.02	-0.13	18	17	3UTR
hsa-miR-11400	NM_032385	FAXDC2	1649	1670	1	1	0.30	-0.31	21	12	3UTR
hsa-miR-11400	NM_006485	FBLN1	2174	2192	1	1	-0.19	-0.32	18	8	3UTR
hsa-miR-11400	NM_001105079	FBRS	4226	4242	0.980769	1	1.53	0.63	16	15	3UTR

mirnaid	refseqid	genesymbol	start	end	bindingp	seed	phylopstem	phylopflank	binding_region_length	longest_consecutive_pairings	position
hsa-miR-11400	NM_001316939	FBXL12	1741	1761	1	1	-0.30	-0.70	20	10	3UTR
hsa-miR-11400	NM_001316940	FBXL12	1619	1639	1	1	-0.30	-0.70	20	10	3UTR
hsa-miR-11400	NM_017703	FBXL12	1723	1743	1	1	-0.30	-0.70	20	10	3UTR
hsa-miR-11400	NM_001282351	FBXL19	3452	3473	1	1	1.70	1.42	21	12	3UTR
hsa-miR-11400	NM_001382779	FBXL19	4278	4299	1	1	1.70	1.42	21	12	3UTR
hsa-miR-11400	NM_001382781	FBXL19	4149	4170	1	1	1.70	1.42	21	12	3UTR
hsa-miR-11400	NM_001099784	FBXL19	3584	3605	1	1	1.70	1.42	21	12	3UTR
hsa-miR-11400	NM_032875	FBXL20	4520	4539	1	1	-0.12	-0.06	19	14	3UTR
hsa-miR-11400	NM_001370208	FBXL20	4638	4657	1	1	-0.28	0.48	19	14	3UTR
hsa-miR-11400	NM_001184906	FBXL20	4424	4443	1	1	-0.02	-0.18	19	14	3UTR
hsa-miR-11400	NM_001278716	FBXL4	7122	7138	1	1	5.77	4.96	16	15	3UTR
hsa-miR-11400	NM_001278317	FBXL7	2383	2419	1	1	-0.23	-0.11	22	8	3UTR
hsa-miR-11400	XM_011513998	FBXL7	2174	2210	1	1	0.00	0.00	22	8	3UTR
hsa-miR-11400	NM_012304	FBXL7	2646	2667	1	1	-0.28	-0.01	21	8	3UTR
hsa-miR-11400	NM_033624	FBXO21	2173	2206	1	1	0.49	0.61	21	9	3UTR
hsa-miR-11400	XM_017019038	FBXO21	1973	2006	1	1	0.00	0.00	21	9	3UTR
hsa-miR-11400	NM_015002	FBXO21	2152	2185	1	1	0.90	0.90	21	9	3UTR

mirnaid	refseqid	genesymbol	start	end	bindingp	seed	phylopstem	phylopflank	binding_region_length	longest_consecutive_pairings	position
hsa-miR-11400	NM_147188	FBXO22	9527	9547	1	1	0.05	0.09	20	12	3UTR
hsa-miR-11400	NM_183420	FBXO25	4233	4252	1	1	-0.06	-0.44	19	9	3UTR
hsa-miR-11400	NM_183421	FBXO25	4260	4279	1	1	-0.06	-0.44	19	9	3UTR
hsa-miR-11400	NM_012173	FBXO25	4183	4202	1	1	0.00	0.00	19	9	3UTR
hsa-miR-11400	NM_178820	FBXO27	1466	1486	1	1	0.71	1.14	20	10	3UTR
hsa-miR-11400	NM_015176	FBXO28	1147	1165	1	1	0.38	1.59	18	8	3UTR
hsa-miR-11400	NM_001136115	FBXO28	951	969	1	1	0.38	1.59	18	8	3UTR
hsa-miR-11400	NM_001257990	FBXO7	1692	1731	1	1	0.27	0.07	20	10	3UTR
hsa-miR-11400	NM_012179	FBXO7	1875	1914	1	1	0.16	0.11	20	10	3UTR
hsa-miR-11400	NM_001033024	FBXO7	1540	1579	1	1	0.27	0.07	20	10	3UTR
hsa-miR-11400	NM_012180	FBXO8	1452	1478	1	1	5.92	5.35	26	9	3UTR
hsa-miR-11400	NM_032029	FCAMR	1269	1289	1	1	0.51	0.44	20	7	3UTR
hsa-miR-11400	NM_001122979	FCAMR	1186	1206	1	1	0.41	0.09	20	7	3UTR
hsa-miR-11400	NM_015962	FCF1	2972	3027	1	1	0.03	-0.31	26	8	3UTR
hsa-miR-11400	NM_001318508	FCF1	3031	3086	1	1	0.03	-0.31	26	8	3UTR
hsa-miR-11400	NM_001271037	FCGR3B	845	863	1	1	-0.54	-0.20	18	7	3UTR
hsa-miR-11400	XM_005268524	FCHSD1	2140	2160	1	1	0.00	0.00	20	8	3UTR

mirnaid	refseqid	genesymbol	start	end	bindingp	seed	phylopstem	phylopflank	binding_region_length	longest_consecutive_pairings	position
hsa-miR-11400	NM_001142473	FCMR	1948	1991	0.980769	1	-0.43	0.74	20	12	3UTR
hsa-miR-11400	NM_005449	FCMR	2298	2327	1	1	0.27	0.16	20	12	3UTR
hsa-miR-11400	NM_001193338	FCMR	2167	2196	1	1	0.27	0.16	20	12	3UTR
hsa-miR-11400	NM_004109	FDX1	1750	1768	1	1	-0.25	0.05	18	13	3UTR
hsa-miR-11400	NM_001371095	FECH	5910	5946	0.980769	1	0.32	-0.08	20	12	3UTR
hsa-miR-11400	NM_015322	FEM1B	6779	6797	1	1	0.33	0.60	18	14	3UTR
hsa-miR-11400	NM_015322	FEM1B	3058	3076	1	1	0.07	0.11	18	8	3UTR
hsa-miR-11400	NM_001308031	FER	2855	2871	1	1	-0.20	-0.43	16	15	3UTR
hsa-miR-11400	NM_005246	FER	6742	6763	1	1	0.00	0.00	21	8	3UTR
hsa-miR-11400	NM_005246	FER	4201	4217	1	1	0.00	0.00	16	15	3UTR
hsa-miR-11400	NM_001024613	FEZF1	2273	2290	1	1	2.26	1.86	17	13	3UTR
hsa-miR-11400	NM_001160264	FEZF1	1884	1901	1	1	3.16	3.39	17	13	3UTR
hsa-miR-11400	NM_181745	FFAR4	1847	1880	1	1	-0.01	0.09	33	10	3UTR
hsa-miR-11400	NM_001195755	FFAR4	1799	1832	1	1	-0.01	0.09	33	10	3UTR
hsa-miR-11400	NM_033642	FGF13	3876	3896	1	1	-0.13	0.08	20	11	3UTR
hsa-miR-11400	NM_001139498	FGF13	4080	4100	1	1	0.16	0.05	20	11	3UTR
hsa-miR-11400	NM_004115	FGF14	11962	11979	1	1	3.97	1.88	17	16	3UTR

mirnaid	refseqid	genesymbol	start	end	bindingp	seed	phylopstem	phylopflank	binding_region_length	longest_consecutive_pairings	position
hsa-miR-11400	NM_152429	FGFBP3	1249	1268	1	1	-0.33	-0.08	19	10	3UTR
hsa-miR-11400	XM_017013225	FGFR1	4204	4229	1	1	0.00	0.00	25	8	3UTR
hsa-miR-11400	NM_023106	FGFR1	4423	4448	1	1	4.72	3.79	25	8	3UTR
hsa-miR-11400	NM_001174065	FGFR1	4088	4113	1	1	6.54	4.21	25	8	3UTR
hsa-miR-11400	NM_001174067	FGFR1	4248	4273	1	1	3.98	5.15	25	8	3UTR
hsa-miR-11400	XM_024454093	FGFRL1	4168	4186	1	1	0.00	0.00	18	10	3UTR
hsa-miR-11400	NM_021923	FGFRL1	2807	2825	1	1	0.25	0.63	18	10	3UTR
hsa-miR-11400	NM_001370296	FGFRL1	2938	2956	1	1	0.25	0.63	18	10	3UTR
hsa-miR-11400	NM_001004356	FGFRL1	3130	3148	1	1	0.25	0.63	18	10	3UTR
hsa-miR-11400	NM_001450	FHL2	2629	2649	1	1	0.45	-0.04	20	8	3UTR
hsa-miR-11400	NM_054014	FKBP1A	762	780	1	1	0.90	0.93	18	17	3UTR
hsa-miR-11400	NM_004117	FKBP5	3408	3429	1	1	5.68	4.00	21	11	3UTR
hsa-miR-11400	NM_004117	FKBP5	2763	2792	1	1	3.62	3.72	18	11	3UTR
hsa-miR-11400	NM_001145775	FKBP5	3593	3614	1	1	1.66	2.17	21	11	3UTR
hsa-miR-11400	NM_001135212	FKBP7	2422	2442	0.980769	1	0.00	0.00	20	11	3UTR
hsa-miR-11400	NM_006731	FKTN	5123	5141	1	1	0.29	0.21	18	6	3UTR
hsa-miR-11400	NM_006731	FKTN	7045	7062	1	1	-0.07	0.29	17	7	3UTR

mirnaid	refseqid	genesymbol	start	end	bindingp	seed	phylopstem	phylopflank	binding_region_length	longest_consecutive_pairings	position
hsa-miR-11400	NM_001079802	FKTN	5214	5232	1	1	0.29	0.21	18	6	3UTR
hsa-miR-11400	NM_001079802	FKTN	7136	7153	1	1	-0.07	0.29	17	7	3UTR
hsa-miR-11400	NM_001198963	FKTN	2184	2201	1	1	-0.07	0.29	17	7	3UTR
hsa-miR-11400	NM_013231	FLRT2	26216	26238	1	1	-0.19	-0.15	22	12	3UTR
hsa-miR-11400	NM_001346144	FLRT2	26238	26260	1	1	-0.19	-0.15	22	12	3UTR
hsa-miR-11400	NM_001278638	FLT3LG	866	904	1	1	0.40	0.04	20	10	3UTR
hsa-miR-11400	XM_005258681	FLT3LG	842	880	1	1	0.00	0.00	20	10	3UTR
hsa-miR-11400	XM_011526682	FLT3LG	862	900	1	1	0.00	0.00	20	10	3UTR
hsa-miR-11400	NM_001459	FLT3LG	849	887	1	1	0.40	0.04	20	10	3UTR
hsa-miR-11400	NM_001204503	FLT3LG	850	888	1	1	-0.27	0.11	20	10	3UTR
hsa-miR-11400	NM_001277313	FMN1	5599	5650	1	1	-0.88	-0.12	15	13	3UTR
hsa-miR-11400	XM_011521506	FMN1	5356	5407	1	1	0.00	0.00	15	13	3UTR
hsa-miR-11400	XM_017022132	FMN1	5557	5617	1	1	0.00	0.00	15	13	3UTR
hsa-miR-11400	NM_001103184	FMN1	4726	4786	1	1	0.00	0.00	15	13	3UTR
hsa-miR-11400	NM_175736	FMNL3	7759	7780	1	1	1.14	0.56	21	13	3UTR
hsa-miR-11400	NM_198900	FMNL3	7606	7627	1	1	4.25	2.92	21	13	3UTR
hsa-miR-11400	NM_001367835	FMNL3	7874	7895	1	1	1.40	0.66	21	13	3UTR

mirnaid	refseqid	genesymbol	start	end	bindingp	seed	phylopstem	phylopflank	binding_region_length	longest_consecutive_pairings	position
hsa-miR-11400	NM_002023	FMOD	2179	2196	0.969231	1	2.23	3.26	17	11	3UTR
hsa-miR-11400	NM_017737	FNBP1L	3450	3473	0.961538	1	2.39	2.15	21	11	3UTR
hsa-miR-11400	NM_001024948	FNBP1L	4753	4776	0.961538	1	1.81	2.14	21	11	3UTR
hsa-miR-11400	NM_001164473	FNBP1L	3624	3647	0.961538	1	1.81	2.14	21	11	3UTR
hsa-miR-11400	NM_001001343	FNDC9	1179	1205	1	1	0.22	-0.19	26	11	3UTR
hsa-miR-11400	NM_002027	FNTA	1548	1565	1	1	0.13	0.99	17	16	3UTR
hsa-miR-11400	XM_006711977	FOSL2	4664	4684	1	1	0.00	0.00	20	8	3UTR
hsa-miR-11400	NM_004514	FOXK2	2191	2229	1	1	0.51	1.27	20	10	3UTR
hsa-miR-11400	NM_005197	FOXN3	2374	2391	1	1	0.90	0.39	17	16	3UTR
hsa-miR-11400	NM_001085471	FOXN3	2215	2232	1	1	-0.02	-0.06	17	16	3UTR
hsa-miR-11400	NM_001368135	FOXO3B	2002	2031	1	1	1.48	0.90	29	10	3UTR
hsa-miR-11400	NM_003838	FPGT	2758	2782	0.961538	1	0.13	0.18	24	7	3UTR
hsa-miR-11400	NM_001462	FPR2	1281	1303	1	1	-0.55	-0.47	22	12	3UTR
hsa-miR-11400	NM_001005738	FPR2	1203	1225	1	1	-0.55	-0.47	22	12	3UTR
hsa-miR-11400	NM_145246	FRA10AC1	2948	2971	1	1	0.58	-0.06	23	9	3UTR
hsa-miR-11400	NM_032428	FRMPD3	7033	7056	1	1	0.00	0.00	23	13	3UTR
hsa-miR-11400	XM_017029901	FRMPD3	7254	7277	1	1	0.00	0.00	23	13	3UTR

mirnaid	refseqid	genesymbol	start	end	bindingp	seed	phylopstem	phylopflank	binding_region_length	longest_consecutive_pairings	position
hsa-miR-11400	NM_001361041	FRRS1	4306	4332	1	1	0.91	0.20	26	8	3UTR
hsa-miR-11400	NM_014334	FRRS1L	1836	1861	1	1	-0.37	-0.01	25	8	3UTR
hsa-miR-11400	NM_014334	FRRS1L	4883	4901	1	1	0.65	0.21	18	17	3UTR
hsa-miR-11400	NM_006653	FRS3	1884	1899	1	1	3.49	4.68	15	14	3UTR
hsa-miR-11400	NM_015082	FSTL4	5167	5193	1	1	0.51	0.76	26	10	3UTR
hsa-miR-11400	NM_001080432	FTO	11126	11144	0.961538	1	-0.36	-0.31	18	10	3UTR
hsa-miR-11400	XM_017023655	FTO	1519	1536	0.969231	1	0.00	0.00	17	13	3UTR
hsa-miR-11400	NM_023934	FUNDC2	5096	5134	1	1	0.09	0.05	38	11	3UTR
hsa-miR-11400	NM_000149	FUT3	2142	2166	1	1	0.16	-0.22	24	6	3UTR
hsa-miR-11400	NM_001097640	FUT3	1740	1764	1	1	1.57	-0.54	24	6	3UTR
hsa-miR-11400	NM_001097641	FUT3	1601	1625	1	1	-0.20	-0.26	24	6	3UTR
hsa-miR-11400	NM_025129	FUZ	1463	1523	1	1	2.90	1.90	19	10	3UTR
hsa-miR-11400	NM_001363663	FUZ	1326	1386	1	1	1.82	2.61	19	10	3UTR
hsa-miR-11400	NM_001171937	FUZ	1355	1415	1	1	2.90	1.90	19	10	3UTR
hsa-miR-11400	NM_181425	FXN	2892	2922	1	1	-0.24	-0.36	30	12	3UTR
hsa-miR-11400	NM_000144	FXN	2884	2914	1	1	-0.24	-0.36	30	12	3UTR
hsa-miR-11400	XM_005247816	FXR1	2160	2178	1	1	0.00	0.00	18	9	3UTR

mirnaid	refseqid	genesymbol	start	end	bindingp	seed	phylopstem	phylopflank	binding_region_length	longest_consecutive_pairings	position
hsa-miR-11400	NM_001011537	FYTTD1	6711	6735	1	1	0.00	0.00	24	7	3UTR
hsa-miR-11400	NM_145866	FZD3	3086	3122	1	1	0.97	0.70	36	12	3UTR
hsa-miR-11400	NM_017412	FZD3	3132	3168	1	1	0.85	0.70	36	12	3UTR
hsa-miR-11400	NM_001270397	G6PC	1432	1453	1	1	0.48	-0.10	21	12	3UTR
hsa-miR-11400	NM_000151	G6PC	1509	1530	1	1	0.48	-0.10	21	12	3UTR
hsa-miR-11400	NM_080491	GAB2	3251	3273	1	1	-0.26	-0.06	22	16	3UTR
hsa-miR-11400	NM_012296	GAB2	3236	3258	1	1	0.17	-0.17	22	16	3UTR
hsa-miR-11400	XM_011514455	GABBR1	2382	2408	1	1	0.00	0.00	26	8	3UTR
hsa-miR-11400	NM_000811	GABRA6	2243	2261	0.969231	1	1.18	0.44	18	11	3UTR
hsa-miR-11400	NM_001371727	GABRB2	5871	5890	1	1	3.37	3.64	19	8	3UTR
hsa-miR-11400	NM_001191320	GABRB3	3682	3696	1	1	0.75	0.63	14	13	3UTR
hsa-miR-11400	NM_173536	GABRG1	5456	5483	1	1	2.95	1.82	18	16	3UTR
hsa-miR-11400	NM_033223	GABRG3	4796	4816	1	1	-0.71	-0.49	20	13	3UTR
hsa-miR-11400	NM_018558	GABRQ	2408	2427	1	1	0.40	0.39	15	13	3UTR
hsa-miR-11400	NM_207359	GADL1	1677	1694	1	1	-0.30	-0.08	17	13	3UTR
hsa-miR-11400	XM_011536618	GALC	3283	3302	1	1	0.00	0.00	19	10	3UTR
hsa-miR-11400	NM_138801	GALM	1993	2011	1	1	0.60	-0.10	18	8	3UTR

mirnaid	refseqid	genesymbol	start	end	bindingp	seed	phylopstem	phylopflank	binding_region_length	longest_consecutive_pairings	position
hsa-miR-11400	NM_198321	GALNT10	4807	4828	1	1	-0.01	0.06	21	12	3UTR
hsa-miR-11400	NM_054110	GALNT15	3791	3818	1	1	-0.09	-0.13	27	13	3UTR
hsa-miR-11400	NM_001319052	GALNT15	2059	2086	1	1	-0.83	-0.21	27	13	3UTR
hsa-miR-11400	XM_011537007	GALNT16	2059	2078	1	1	0.00	0.00	19	8	3UTR
hsa-miR-11400	XM_011537007	GALNT16	2035	2056	1	1	0.00	0.00	21	8	3UTR
hsa-miR-11400	NM_020692	GALNT16	3872	3889	1	1	0.10	-0.41	17	14	3UTR
hsa-miR-11400	NM_138924	GAMT	1337	1388	0.974359	1	3.79	3.71	26	8	3UTR
hsa-miR-11400	NM_001304428	GAPT	1228	1249	1	1	-0.22	0.06	21	10	3UTR
hsa-miR-11400	NM_014686	GARRE1	4775	4814	1	1	0.16	0.09	21	13	3UTR
hsa-miR-11400	NM_014686	GARRE1	5712	5737	1	1	-0.19	0.13	25	8	3UTR
hsa-miR-11400	NM_016613	GASK1B	2304	2323	1	1	-0.14	0.09	19	7	3UTR
hsa-miR-11400	XM_024452363	GATA1	1127	1144	1	1	0.00	0.00	17	16	3UTR
hsa-miR-11400	NM_002049	GATA1	1318	1335	1	1	0.24	0.60	17	16	3UTR
hsa-miR-11400	NM_032638	GATA2	3147	3169	1	1	0.01	0.22	20	7	3UTR
hsa-miR-11400	NM_001145661	GATA2	3234	3256	1	1	2.42	1.28	20	7	3UTR
hsa-miR-11400	NM_001145662	GATA2	3013	3035	1	1	0.58	1.52	20	7	3UTR
hsa-miR-11400	NM_080473	GATA5	2223	2245	1	1	1.82	0.69	22	8	3UTR

mirnaid	refseqid	genesymbol	start	end	bindingp	seed	phylopstem	phylopflank	binding_region_length	longest_consecutive_pairings	position
hsa-miR-11400	NM_020699	GATAD2B	3944	3959	1	1	0.45	0.23	15	14	3UTR
hsa-miR-11400	NM_020699	GATAD2B	6901	6916	1	1	2.93	4.03	15	14	3UTR
hsa-miR-11400	NM_176818	GATC	3779	3798	1	1	-0.50	-0.14	19	10	3UTR
hsa-miR-11400	NM_176818	GATC	4092	4110	1	1	0.37	0.10	18	8	3UTR
hsa-miR-11400	NM_001005741	GBA	1876	1895	0.961538	1	3.70	1.78	19	12	3UTR
hsa-miR-11400	NM_001171812	GBA	1676	1695	0.961538	1	3.59	2.82	19	12	3UTR
hsa-miR-11400	NM_000157	GBA	2061	2080	1	1	-0.07	0.50	19	11	3UTR
hsa-miR-11400	NM_000157	GBA	1823	1842	1	1	1.99	2.54	19	12	3UTR
hsa-miR-11400	NM_001171812	GBA	1914	1933	1	1	-0.06	0.48	19	11	3UTR
hsa-miR-11400	NM_001282629	GBGT1	1476	1494	1	1	2.36	1.66	18	9	3UTR
hsa-miR-11400	NM_001282632	GBGT1	1444	1462	1	1	1.53	2.38	18	9	3UTR
hsa-miR-11400	NM_021996	GBGT1	1495	1513	1	1	2.36	1.66	18	9	3UTR
hsa-miR-11400	NM_018284	GBP3	2630	2657	1	1	1.54	3.16	27	8	3UTR
hsa-miR-11400	NM_052941	GBP4	5169	5186	1	1	1.75	0.40	17	14	3UTR
hsa-miR-11400	NM_001362929	GDAP1	2209	2231	1	1	-0.14	-0.01	22	12	3UTR
hsa-miR-11400	NM_001362930	GDAP1	2180	2202	1	1	-0.14	-0.01	22	12	3UTR
hsa-miR-11400	NM_001362932	GDAP1	2161	2183	1	1	-0.14	-0.01	22	12	3UTR

mirnaid	refseqid	genesymbol	start	end	bindingp	seed	phylopstem	phylopflank	binding_region_length	longest_consecutive_pairings	position
hsa-miR-11400	NM_001040875	GDAP1	2224	2246	1	1	-0.14	-0.01	22	12	3UTR
hsa-miR-11400	NM_017686	GDAP2	6731	6752	1	1	0.23	0.94	21	7	3UTR
hsa-miR-11400	NM_016641	GDE1	2052	2071	1	1	4.08	3.54	19	10	3UTR
hsa-miR-11400	NM_004962	GDF10	2483	2504	1	1	0.51	0.99	21	10	3UTR
hsa-miR-11400	NM_016204	GDF2	1807	1829	1	1	-0.19	-0.06	22	6	3UTR
hsa-miR-11400	NM_001494	GDI2	2051	2079	1	1	6.73	4.22	28	10	3UTR
hsa-miR-11400	NM_001115156	GDI2	1916	1944	1	1	6.73	4.22	28	10	3UTR
hsa-miR-11400	NM_000514	GDNF	1446	1462	1	1	0.70	-0.03	16	15	3UTR
hsa-miR-11400	NM_001190468	GDNF	1105	1121	1	1	0.07	-0.09	16	15	3UTR
hsa-miR-11400	NM_017856	GEMIN8	1054	1073	0.980769	1	-0.14	-0.04	19	12	3UTR
hsa-miR-11400	NM_001042480	GEMIN8	884	903	0.980769	1	-0.13	-0.03	19	12	3UTR
hsa-miR-11400	NM_001242628	GFOD1	6525	6550	1	1	0.00	0.00	17	15	3UTR
hsa-miR-11400	NM_001242630	GFOD1	6619	6644	1	1	0.00	0.00	17	15	3UTR
hsa-miR-11400	NM_001242630	GFOD1	1194	1215	1	1	-0.32	0.74	21	8	3UTR
hsa-miR-11400	NM_001145453	GFRA1	6859	6907	1	1	1.11	0.73	18	7	3UTR
hsa-miR-11400	XM_006716327	GFRA2	2331	2373	1	1	0.00	0.00	21	9	3UTR
hsa-miR-11400	XM_006716327	GFRA2	1551	1571	1	1	0.00	0.00	20	7	3UTR

mirnaid	refseqid	genesymbol	start	end	bindingp	seed	phylopstem	phylopflank	binding_region_length	longest_consecutive_pairings	position
hsa-miR-11400	NM_001495	GFRA2	2146	2166	1	1	0.48	-0.12	20	7	3UTR
hsa-miR-11400	NM_001165038	GFRA2	2633	2653	1	1	0.16	0.39	20	9	3UTR
hsa-miR-11400	NM_001165038	GFRA2	1831	1851	1	1	4.15	1.87	20	7	3UTR
hsa-miR-11400	NM_001165039	GFRA2	2549	2569	1	1	0.14	0.06	20	9	3UTR
hsa-miR-11400	NM_138619	GGA3	2610	2626	1	1	1.15	2.15	16	15	3UTR
hsa-miR-11400	NM_014001	GGA3	2511	2527	1	1	0.46	1.03	16	15	3UTR
hsa-miR-11400	NM_001172703	GGA3	2633	2649	1	1	1.23	2.90	16	15	3UTR
hsa-miR-11400	NM_015575	GIGYF2	5332	5357	1	1	0.27	0.32	25	7	3UTR
hsa-miR-11400	NM_001103146	GIGYF2	5192	5217	1	1	0.27	0.32	25	7	3UTR
hsa-miR-11400	NM_001103147	GIGYF2	5325	5350	1	1	0.27	0.32	25	7	3UTR
hsa-miR-11400	NM_001103148	GIGYF2	5108	5133	1	1	0.27	0.32	25	7	3UTR
hsa-miR-11400	NM_030772	GJA9	2074	2122	1	1	0.14	0.53	15	7	3UTR
hsa-miR-11400	NM_004004	GJB2	1220	1238	1	1	0.53	-0.25	18	14	3UTR
hsa-miR-11400	XM_011537775	GLIPR1	3449	3470	1	1	0.00	0.00	21	7	3UTR
hsa-miR-11400	NM_006851	GLIPR1	3377	3398	1	1	-0.04	-0.02	21	7	3UTR
hsa-miR-11400	NM_152629	GLIS3	6084	6104	1	1	2.35	2.89	20	7	3UTR
hsa-miR-11400	NM_001256605	GLMP	1192	1213	1	1	2.83	0.42	19	10	3UTR

mirnaid	refseqid	genesymbol	start	end	bindingp	seed	phylopstem	phylopflank	binding_region_length	longest_consecutive_pairings	position
hsa-miR-11400	NM_001256608	GLMP	1193	1214	1	1	2.89	1.13	19	10	3UTR
hsa-miR-11400	NM_144580	GLMP	1450	1471	1	1	2.92	1.07	19	10	3UTR
hsa-miR-11400	XM_005256861	GLP2R	1664	1681	1	1	0.00	0.00	17	8	3UTR
hsa-miR-11400	NM_004246	GLP2R	2115	2132	1	1	0.53	-0.12	17	8	3UTR
hsa-miR-11400	NM_001033044	GLUL	6338	6356	1	1	3.35	3.39	18	14	3UTR
hsa-miR-11400	NM_145262	GLYCTK	3105	3123	1	1	0.05	-0.14	18	11	3UTR
hsa-miR-11400	NM_001144951	GLYCTK	2929	2947	1	1	0.00	0.00	18	11	3UTR
hsa-miR-11400	NM_024482	GMEB1	3912	3929	1	1	0.77	1.06	17	13	3UTR
hsa-miR-11400	NM_006582	GMEB1	3945	3962	1	1	0.77	1.06	17	13	3UTR
hsa-miR-11400	NM_001319674	GMEB1	3915	3932	1	1	0.77	1.06	17	13	3UTR
hsa-miR-11400	NM_001282440	GNA12	2970	2990	1	1	0.31	0.56	20	11	3UTR
hsa-miR-11400	NM_001282441	GNA12	3069	3089	1	1	3.34	2.55	20	11	3UTR
hsa-miR-11400	NM_001282441	GNA12	1740	1762	1	1	0.60	0.00	22	9	3UTR
hsa-miR-11400	NM_001261443	GNAL	1598	1621	1	1	-0.04	0.29	23	10	3UTR
hsa-miR-11400	NM_001261444	GNAL	1001	1024	1	1	-0.04	0.29	23	10	3UTR
hsa-miR-11400	NM_182978	GNAL	1990	2013	1	1	-0.04	0.29	23	10	3UTR
hsa-miR-11400	NM_001369387	GNAL	1808	1831	1	1	-0.04	0.29	23	10	3UTR

mirnaid	refseqid	genesymbol	start	end	bindingp	seed	phylopstem	phylopflank	binding_region_length	longest_consecutive_pairings	position
hsa-miR-11400	NM_001142339	GNAL	1675	1698	1	1	-0.04	0.29	23	10	3UTR
hsa-miR-11400	XM_017014167	GNE	3173	3198	1	1	0.00	0.00	25	8	3UTR
hsa-miR-11400	NM_005476	GNE	3277	3302	1	1	4.06	4.15	25	8	3UTR
hsa-miR-11400	NM_001190388	GNE	3028	3053	1	1	3.71	3.74	25	8	3UTR
hsa-miR-11400	NM_004126	GNG11	2223	2244	1	1	0.26	0.30	21	8	3UTR
hsa-miR-11400	XM_011544167	GNG4	2250	2268	1	1	0.00	0.00	18	8	3UTR
hsa-miR-11400	NM_004485	GNG4	2291	2309	1	1	0.00	0.00	18	8	3UTR
hsa-miR-11400	NM_001098721	GNG4	2403	2421	1	1	0.00	0.00	18	8	3UTR
hsa-miR-11400	NM_021955	GNGT1	502	529	1	1	0.24	0.19	27	9	3UTR
hsa-miR-11400	NM_005275	GNL1	7234	7254	1	1	1.23	0.58	20	12	3UTR
hsa-miR-11400	NM_138335	GNPDA2	1174	1193	1	1	1.06	1.94	19	8	3UTR
hsa-miR-11400	NM_024312	GNPTAB	5444	5470	1	1	0.86	2.16	26	8	3UTR
hsa-miR-11400	NM_000406	GNRHR	1487	1505	1	1	0.54	0.24	18	8	3UTR
hsa-miR-11400	NM_000406	GNRHR	3918	3934	1	1	2.68	2.93	16	15	3UTR
hsa-miR-11400	NM_001012763	GNRHR	1359	1377	1	1	0.09	0.05	18	8	3UTR
hsa-miR-11400	NM_001012763	GNRHR	3790	3806	1	1	3.03	1.91	16	15	3UTR
hsa-miR-11400	NM_004486	GOLGA2	3893	3912	1	1	1.78	2.48	19	9	3UTR

mirnaid	refseqid	genesymbol	start	end	bindingp	seed	phylopstem	phylopflank	binding_region_length	longest_consecutive_pairings	position
hsa-miR-11400	XM_006719736	GOLGA3	6005	6024	1	1	0.00	0.00	19	10	3UTR
hsa-miR-11400	NM_005895	GOLGA3	5965	5984	1	1	2.18	2.34	19	10	3UTR
hsa-miR-11400	NM_004871	GOSR1	5544	5580	1	1	-0.05	0.15	36	10	3UTR
hsa-miR-11400	NM_001007024	GOSR1	5560	5596	1	1	-0.05	0.15	36	10	3UTR
hsa-miR-11400	NM_001007025	GOSR1	5538	5574	1	1	-0.05	0.15	36	10	3UTR
hsa-miR-11400	XM_006722190	GOSR2	1204	1224	1	1	0.00	0.00	20	10	3UTR
hsa-miR-11400	XM_017025389	GOSR2	2544	2585	1	1	0.00	0.00	17	15	3UTR
hsa-miR-11400	XM_017025395	GOSR2	2769	2785	1	1	0.00	0.00	16	15	3UTR
hsa-miR-11400	NM_001321134	GOSR2	2392	2433	1	1	0.09	-0.05	17	15	3UTR
hsa-miR-11400	NM_001330252	GOSR2	2344	2385	1	1	0.09	-0.05	17	15	3UTR
hsa-miR-11400	NM_001353114	GOSR2	2482	2523	1	1	0.09	-0.05	17	15	3UTR
hsa-miR-11400	NM_001353115	GOSR2	2341	2382	1	1	0.09	-0.05	17	15	3UTR
hsa-miR-11400	NM_001363851	GOSR2	2921	2937	1	1	0.11	0.08	16	15	3UTR
hsa-miR-11400	NM_004488	GP5	2143	2160	1	1	-0.07	0.44	17	8	3UTR
hsa-miR-11400	NM_001278505	GPATCH11	768	807	1	1	-0.05	0.38	19	10	3UTR
hsa-miR-11400	NM_174931	GPATCH11	1037	1076	1	1	-0.05	0.38	19	10	3UTR
hsa-miR-11400	XM_017020302	GPC6	3805	3829	1	1	0.00	0.00	24	7	3UTR

mirnaid	refseqid	genesymbol	start	end	bindingp	seed	phylopstem	phylopflank	binding_region_length	longest_consecutive_pairings	position
hsa-miR-11400	NM_005708	GPC6	4826	4850	1	1	0.51	0.45	24	7	3UTR
hsa-miR-11400	XM_005246469	GPD2	3882	3898	1	1	0.00	0.00	16	7	3UTR
hsa-miR-11400	XM_011510978	GPD2	3987	4003	1	1	0.00	0.00	16	7	3UTR
hsa-miR-11400	NM_005278	GPM6B	2656	2676	1	1	0.00	0.00	20	7	3UTR
hsa-miR-11400	NM_001001994	GPM6B	2526	2558	1	1	1.17	1.32	21	7	3UTR
hsa-miR-11400	NM_001001996	GPM6B	2776	2796	1	1	1.21	0.55	20	7	3UTR
hsa-miR-11400	NM_018066	GPN2	1234	1254	1	1	0.05	0.11	20	8	3UTR
hsa-miR-11400	NM_001261454	GPR1	1376	1391	1	1	0.00	0.00	15	6	3UTR
hsa-miR-11400	NM_054021	GPR101	3234	3275	1	1	1.07	0.98	41	13	3UTR
hsa-miR-11400	XM_024449694	GPR135	4558	4576	1	1	0.00	0.00	18	17	3UTR
hsa-miR-11400	NM_001303473	GPR146	1684	1704	1	1	-1.15	-0.82	20	19	3UTR
hsa-miR-11400	NM_138445	GPR146	1685	1705	1	1	-1.15	-0.82	20	19	3UTR
hsa-miR-11400	NM_001038705	GPR149	4640	4665	1	1	1.16	3.67	25	13	3UTR
hsa-miR-11400	NM_001267050	GPR155	4911	4936	0.961538	1	3.82	2.64	25	8	3UTR
hsa-miR-11400	XM_017005795	GPR156	4058	4081	1	1	0.00	0.00	23	8	3UTR
hsa-miR-11400	XM_017005795	GPR156	2975	2992	1	1	0.00	0.00	17	16	3UTR
hsa-miR-11400	XM_017005795	GPR156	3322	3340	1	1	0.00	0.00	18	12	3UTR

mirnaid	refseqid	genesymbol	start	end	bindingp	seed	phylopstem	phylopflank	binding_region_length	longest_consecutive_pairings	position
hsa-miR-11400	NM_001267609	GPR161	4286	4310	1	1	-0.06	-0.19	24	10	3UTR
hsa-miR-11400	NM_001267611	GPR161	3836	3860	1	1	-0.12	-0.12	24	10	3UTR
hsa-miR-11400	NM_001267612	GPR161	4286	4310	1	1	-0.06	-0.19	24	10	3UTR
hsa-miR-11400	NM_180989	GPR180	4056	4091	1	1	0.21	0.12	25	9	3UTR
hsa-miR-11400	NM_005293	GPR20	1439	1455	1	1	-0.29	-0.53	16	15	3UTR
hsa-miR-11400	NM_005282	GPR4	2653	2689	1	1	0.00	0.00	18	10	3UTR
hsa-miR-11400	NM_030784	GPR63	3585	3625	1	1	0.46	0.01	20	11	3UTR
hsa-miR-11400	NM_003608	GPR65	4400	4413	1	1	-0.16	0.19	13	12	3UTR
hsa-miR-11400	NM_001146265	GPR85	2763	2784	1	1	1.26	0.97	17	7	3UTR
hsa-miR-11400	NM_001366261	GPRC5C	3166	3195	1	1	0.21	-0.29	22	8	3UTR
hsa-miR-11400	NM_198281	GPRIN3	13193	13239	1	1	0.31	0.33	24	10	3UTR
hsa-miR-11400	NM_198281	GPRIN3	4750	4766	1	1	0.83	1.14	16	15	3UTR
hsa-miR-11400	XM_017008044	GPRIN3	4758	4774	1	1	0.00	0.00	16	15	3UTR
hsa-miR-11400	NM_201397	GPX1	881	897	1	1	2.30	3.40	16	15	3UTR
hsa-miR-11400	NM_001329502	GPX1	743	759	1	1	2.30	3.31	16	15	3UTR
hsa-miR-11400	NM_001329503	GPX1	621	637	1	1	2.30	3.43	16	15	3UTR
hsa-miR-11400	NM_015696	GPX7	802	841	1	1	0.44	0.36	23	6	3UTR

mirnaid	refseqid	genesymbol	start	end	bindingp	seed	phylopstem	phylopflank	binding_region_length	longest_consecutive_pairings	position
hsa-miR-11400	XM_011542933	GRAMD1B	7791	7810	0.974359	1	0.00	0.00	19	7	3UTR
hsa-miR-11400	XM_017018050	GRAMD1B	7396	7415	0.974359	1	0.00	0.00	19	7	3UTR
hsa-miR-11400	NM_001286563	GRAMD1B	7680	7699	1	1	0.69	0.60	19	7	3UTR
hsa-miR-11400	NM_001286564	GRAMD1B	7379	7398	1	1	0.69	0.60	19	7	3UTR
hsa-miR-11400	NM_020716	GRAMD1B	7659	7678	1	1	0.69	0.60	19	7	3UTR
hsa-miR-11400	NM_001367421	GRAMD1B	7883	7902	1	1	0.69	0.60	19	7	3UTR
hsa-miR-11400	NM_001146319	GRAMD2B	1648	1665	1	1	1.79	0.54	17	13	3UTR
hsa-miR-11400	NM_001291825	GRAP2	2426	2440	1	1	0.00	0.00	14	13	3UTR
hsa-miR-11400	NM_004810	GRAP2	2397	2411	1	1	-0.74	-0.28	14	13	3UTR
hsa-miR-11400	NM_001001550	GRB10	1996	2034	1	1	-0.56	0.08	38	11	3UTR
hsa-miR-11400	NM_013372	GREM1	900	922	1	1	0.00	0.00	22	8	3UTR
hsa-miR-11400	NM_001368719	GREM1	961	983	1	1	1.09	0.46	22	8	3UTR
hsa-miR-11400	NM_001191323	GREM1	777	799	1	1	1.09	0.46	22	8	3UTR
hsa-miR-11400	NM_001258019	GRIA1	3631	3645	1	1	0.80	0.38	14	13	3UTR
hsa-miR-11400	NM_001258021	GRIA1	3709	3723	1	1	0.15	0.33	14	13	3UTR
hsa-miR-11400	NM_001258022	GRIA1	3709	3723	1	1	0.15	0.33	14	13	3UTR
hsa-miR-11400	NM_001258023	GRIA1	3606	3620	1	1	0.15	0.33	14	13	3UTR

mirnaid	refseqid	genesymbol	start	end	bindingp	seed	phylopstem	phylopflank	binding_region_length	longest_consecutive_pairings	position
hsa-miR-11400	NM_000827	GRIA1	3871	3885	1	1	0.15	0.33	14	13	3UTR
hsa-miR-11400	NM_001114183	GRIA1	3871	3885	1	1	0.80	0.38	14	13	3UTR
hsa-miR-11400	NM_001282470	GRIK4	4845	4866	1	1	0.08	1.34	21	7	3UTR
hsa-miR-11400	NM_133445	GRIN3A	7126	7148	1	1	2.16	2.57	22	8	3UTR
hsa-miR-11400	NM_005160	GRK3	6843	6864	1	1	-0.63	-0.51	21	10	3UTR
hsa-miR-11400	NM_000637	GSR	2520	2537	0.961538	1	3.73	3.20	17	9	3UTR
hsa-miR-11400	NM_001195102	GSR	2433	2450	0.961538	1	3.57	3.06	17	9	3UTR
hsa-miR-11400	NM_001195103	GSR	2361	2378	0.961538	1	3.57	3.06	17	9	3UTR
hsa-miR-11400	NM_001195104	GSR	2274	2291	0.961538	1	3.57	3.06	17	9	3UTR
hsa-miR-11400	NM_001322494	GSS	1930	1945	1	1	0.06	-0.07	15	8	3UTR
hsa-miR-11400	NM_001322495	GSS	2692	2707	1	1	0.20	0.03	15	8	3UTR
hsa-miR-11400	NM_001284234	GTDC1	1467	1486	1	1	-0.42	0.17	19	9	3UTR
hsa-miR-11400	NM_001164629	GTDC1	1752	1771	1	1	-0.13	2.09	19	9	3UTR
hsa-miR-11400	NM_001376000	GTF2H2C	2168	2193	1	1	-0.14	0.08	25	8	3UTR
hsa-miR-11400	NM_033107	GTPBP10	2607	2626	1	1	0.00	0.07	19	10	3UTR
hsa-miR-11400	NM_001042717	GTPBP10	2370	2389	1	1	0.00	0.07	19	10	3UTR
hsa-miR-11400	NM_001284256	GUCD1	755	792	0.974359	1	0.27	-0.16	23	9	3UTR

mirnaid	refseqid	genesymbol	start	end	bindingp	seed	phylopstem	phylopflank	binding_region_length	longest_consecutive_pairings	position
hsa-miR-11400	NM_001284251	GUCD1	1087	1124	1	1	-0.34	-0.18	23	9	3UTR
hsa-miR-11400	NM_001284254	GUCD1	994	1031	1	1	-0.57	-0.19	23	9	3UTR
hsa-miR-11400	NM_001284257	GUCD1	752	789	1	1	0.15	-0.20	23	9	3UTR
hsa-miR-11400	NM_031444	GUCD1	997	1034	1	1	-0.61	-0.17	23	9	3UTR
hsa-miR-11400	NM_001256449	GUCY1A1	8228	8247	1	1	-0.51	-0.07	19	18	3UTR
hsa-miR-11400	NM_000856	GUCY1A1	8138	8157	1	1	-0.51	-0.07	19	18	3UTR
hsa-miR-11400	NM_001130682	GUCY1A1	8044	8063	1	1	-0.51	-0.07	19	18	3UTR
hsa-miR-11400	NM_001130683	GUCY1A1	8299	8318	1	1	-0.51	-0.07	19	18	3UTR
hsa-miR-11400	NM_001130684	GUCY1A1	8123	8142	1	1	-0.51	-0.07	19	18	3UTR
hsa-miR-11400	NM_001130685	GUCY1A1	8120	8139	1	1	-0.51	-0.07	19	18	3UTR
hsa-miR-11400	NM_001270781	GZMH	588	637	0.961538	1	-0.03	-0.11	18	8	3UTR
hsa-miR-11400	NM_033423	GZMH	846	895	0.961538	1	-0.03	-0.11	18	8	3UTR
hsa-miR-11400	NM_177925	H2AJ	2953	2972	1	1	0.74	0.13	19	9	3UTR
hsa-miR-11400	NM_198402	HACD2	3058	3076	1	1	1.03	0.76	18	11	3UTR
hsa-miR-11400	NM_001010915	HACD4	7206	7222	1	1	0.01	0.46	16	6	3UTR
hsa-miR-11400	NM_177977	HAP1	2670	2687	1	1	0.14	0.54	17	10	3UTR
hsa-miR-11400	NM_001079870	HAP1	2622	2639	1	1	0.59	0.73	17	10	3UTR

mirnaid	refseqid	genesymbol	start	end	bindingp	seed	phylopstem	phylopflank	binding_region_length	longest_consecutive_pairings	position
hsa-miR-11400	NM_001079871	HAP1	2598	2615	1	1	0.64	0.39	17	10	3UTR
hsa-miR-11400	NM_005328	HAS2	3835	3856	1	1	1.09	0.91	21	7	3UTR
hsa-miR-11400	NM_005329	HAS3	2316	2336	1	1	1.01	1.05	20	8	3UTR
hsa-miR-11400	NM_001199280	HAS3	2295	2315	1	1	1.01	1.05	20	8	3UTR
hsa-miR-11400	NM_001303143	HAUS3	4650	4666	1	1	1.87	1.96	16	13	3UTR
hsa-miR-11400	NM_024511	HAUS3	4379	4395	1	1	1.67	2.41	16	13	3UTR
hsa-miR-11400	NM_021072	HCN1	7529	7545	1	1	3.21	3.48	16	15	3UTR
hsa-miR-11400	XM_005265491	HDAC11	1996	2039	1	1	0.00	0.00	18	10	3UTR
hsa-miR-11400	NM_024827	HDAC11	1940	1983	1	1	-0.30	-0.07	18	10	3UTR
hsa-miR-11400	NM_001330636	HDAC11	1703	1746	1	1	-0.30	-0.07	18	10	3UTR
hsa-miR-11400	NM_001136041	HDAC11	1968	2011	1	1	-0.30	-0.07	18	10	3UTR
hsa-miR-11400	NM_178425	HDAC9	8667	8685	1	1	0.40	0.28	18	9	3UTR
hsa-miR-11400	NM_001321877	HDAC9	8810	8828	1	1	0.40	0.28	18	9	3UTR
hsa-miR-11400	NM_001286451	HDDC3	1368	1393	1	1	3.03	3.19	25	6	3UTR
hsa-miR-11400	NM_001317851	HEMK1	3566	3591	1	1	0.04	0.00	23	10	3UTR
hsa-miR-11400	NM_016173	HEMK1	3525	3550	1	1	0.33	-0.06	23	10	3UTR
hsa-miR-11400	NM_001377421	HEMK1	3496	3521	1	1	0.33	-0.06	23	10	3UTR

mirnaid	refseqid	genesymbol	start	end	bindingp	seed	phylopstem	phylopflank	binding_region_length	longest_consecutive_pairings	position
hsa-miR-11400	NM_152419	HGSNAT	4895	4914	1	1	-0.17	-0.25	19	13	3UTR
hsa-miR-11400	NM_017902	HIF1AN	7661	7686	1	1	-0.04	0.06	25	8	3UTR
hsa-miR-11400	XM_005259153	HIF3A	2232	2252	1	1	0.00	0.00	20	10	3UTR
hsa-miR-11400	NM_005338	HIP1	4693	4734	0.980769	1	-0.01	-0.04	19	10	3UTR
hsa-miR-11400	NM_001243198	HIP1	4540	4581	0.980769	1	0.00	0.00	19	10	3UTR
hsa-miR-11400	NM_001197323	HIRIP3	2050	2076	0.961538	1	-0.38	0.08	14	12	3UTR
hsa-miR-11400	NM_003609	HIRIP3	2509	2535	1	1	1.54	1.71	14	12	3UTR
hsa-miR-11400	NM_001352515	HLCS	5225	5243	1	1	-0.49	-0.30	18	9	3UTR
hsa-miR-11400	NM_001352516	HLCS	5428	5446	1	1	-0.55	-0.16	18	9	3UTR
hsa-miR-11400	NM_000411	HLCS	5371	5389	1	1	-0.11	-0.32	18	9	3UTR
hsa-miR-11400	NM_000411	HLCS	5318	5356	1	1	-0.09	-0.28	24	11	3UTR
hsa-miR-11400	NM_001242784	HLCS	6314	6337	1	1	0.00	0.00	23	11	3UTR
hsa-miR-11400	NM_001242785	HLCS	5528	5546	1	1	-0.39	-0.51	18	9	3UTR
hsa-miR-11400	NM_178582	HM13	2274	2316	0.969231	1	-0.44	0.00	23	11	3UTR
hsa-miR-11400	NM_018200	HMG20A	2021	2039	1	1	1.00	0.86	18	9	3UTR
hsa-miR-11400	XM_024451326	HMG20B	1748	1772	1	1	0.00	0.00	24	9	3UTR
hsa-miR-11400	NM_003483	HMGA2	1359	1377	1	1	4.70	3.73	18	17	3UTR

mirnaid	refseqid	genesymbol	start	end	bindingp	seed	phylopstem	phylopflank	binding_region_length	longest_consecutive_pairings	position
hsa-miR-11400	NM_001313892	HMGB1	4090	4110	0.961538	1	0.90	2.80	20	10	3UTR
hsa-miR-11400	NM_004966	HNRNPF	2211	2229	1	1	3.66	2.94	18	8	3UTR
hsa-miR-11400	NM_001098205	HNRNPF	2245	2263	1	1	2.17	2.55	18	8	3UTR
hsa-miR-11400	NM_001098206	HNRNPF	2176	2194	1	1	1.93	3.91	18	8	3UTR
hsa-miR-11400	NM_001098208	HNRNPF	2294	2312	1	1	0.39	1.20	18	8	3UTR
hsa-miR-11400	NM_004501	HNRNPU	2995	3015	0.953846	1	3.94	2.41	20	8	3UTR
hsa-miR-11400	NM_020834	HOMEZ	3558	3604	1	1	3.47	2.89	19	11	3UTR
hsa-miR-11400	NM_032410	HOOK3	9734	9752	1	1	0.06	0.06	18	8	3UTR
hsa-miR-11400	XM_011529917	HORMAD2	1678	1694	1	1	0.00	0.00	16	7	3UTR
hsa-miR-11400	NM_173860	HOXC12	2143	2178	1	1	0.42	0.11	22	8	3UTR
hsa-miR-11400	NM_173860	HOXC12	1712	1730	1	1	-0.48	0.07	18	14	3UTR
hsa-miR-11400	NM_173860	HOXC12	2558	2595	1	1	0.55	0.13	37	10	3UTR
hsa-miR-11400	NM_016257	HPCAL4	2708	2731	1	1	1.18	0.43	23	6	3UTR
hsa-miR-11400	NM_001166498	HPSE	2731	2756	1	1	-0.06	0.06	25	8	3UTR
hsa-miR-11400	NM_000861	HRH1	4323	4342	1	1	-0.28	-0.09	19	8	3UTR
hsa-miR-11400	NM_001098212	HRH1	4229	4248	1	1	-0.28	-0.09	19	8	3UTR
hsa-miR-11400	NM_001098213	HRH1	4329	4348	1	1	-0.28	-0.09	19	8	3UTR

mirnaid	refseqid	genesymbol	start	end	bindingp	seed	phylopstem	phylopflank	binding_region_length	longest_consecutive_pairings	position
hsa-miR-11400	NM_001537	HSBP1	3276	3310	1	1	-0.75	-0.04	21	19	3UTR
hsa-miR-11400	NM_001318316	HSCB	786	805	1	1	1.82	2.48	19	8	3UTR
hsa-miR-11400	XM_017023732	HSD3B7	1499	1517	1	1	0.00	0.00	18	11	3UTR
hsa-miR-11400	NM_031463	HSDL1	2687	2710	1	1	1.03	2.28	23	8	3UTR
hsa-miR-11400	NM_001146051	HSDL1	2522	2545	1	1	1.49	1.62	23	8	3UTR
hsa-miR-11400	NM_032303	HSDL2	2171	2190	1	1	0.04	0.16	19	11	3UTR
hsa-miR-11400	NM_001195822	HSDL2	1952	1971	1	1	0.73	0.41	19	11	3UTR
hsa-miR-11400	XM_005272316	HSF1	2100	2147	1	1	0.00	0.00	17	15	3UTR
hsa-miR-11400	NM_005526	HSF1	2023	2070	1	1	1.61	0.06	17	15	3UTR
hsa-miR-11400	NM_032855	HSH2D	2256	2281	1	1	-0.20	0.02	25	8	3UTR
hsa-miR-11400	NM_001382417	HSH2D	1842	1867	1	1	-0.20	0.02	25	8	3UTR
hsa-miR-11400	NM_025015	HSPA12A	3439	3457	0.969231	1	0.23	0.82	18	11	3UTR
hsa-miR-11400	XM_005269673	HSPA12A	6572	6590	1	1	0.00	0.00	18	11	3UTR
hsa-miR-11400	XM_011539580	HSPA12A	3603	3621	1	1	0.00	0.00	18	11	3UTR
hsa-miR-11400	XM_017016032	HSPA12A	3197	3215	1	1	0.00	0.00	18	11	3UTR
hsa-miR-11400	NM_001330164	HSPA12A	3547	3565	1	1	0.35	2.02	18	11	3UTR
hsa-miR-11400	NM_001317382	HSPA4L	2820	2844	1	1	1.46	1.98	24	14	3UTR

mirnaid	refseqid	genesymbol	start	end	bindingp	seed	phylopstem	phylopflank	binding_region_length	longest_consecutive_pairings	position
hsa-miR-11400	NM_014278	HSPA4L	2998	3022	1	1	1.46	1.98	24	14	3UTR
hsa-miR-11400	NM_014365	HSPB8	1024	1052	1	1	1.27	0.49	28	10	3UTR
hsa-miR-11400	NM_006410	HTATIP2	1085	1134	1	1	-0.20	0.11	19	6	3UTR
hsa-miR-11400	NM_001098522	HTATIP2	1572	1621	1	1	0.60	-0.05	19	6	3UTR
hsa-miR-11400	NM_002111	HTT	11954	11983	1	1	-0.67	-0.98	20	16	3UTR
hsa-miR-11400	NM_153283	HYAL1	1135	1167	1	1	1.68	2.49	32	10	3UTR
hsa-miR-11400	NM_153285	HYAL1	717	749	1	1	0.61	0.98	32	10	3UTR
hsa-miR-11400	XM_011533668	HYAL1	1800	1832	1	1	0.00	0.00	32	10	3UTR
hsa-miR-11400	NM_016400	HYPK	2317	2332	1	1	0.20	0.07	15	14	3UTR
hsa-miR-11400	NM_001010867	IBA57	5126	5147	1	1	-0.03	0.05	21	10	3UTR
hsa-miR-11400	NM_001288623	ICA1L	7480	7504	1	1	2.69	2.99	24	9	3UTR
hsa-miR-11400	XM_024452060	ICOSLG	1833	1851	1	1	0.00	0.00	18	17	3UTR
hsa-miR-11400	NM_001270930	IFIT1	3160	3184	1	1	0.07	-0.02	24	8	3UTR
hsa-miR-11400	NM_001548	IFIT1	2925	2949	1	1	0.19	0.07	24	8	3UTR
hsa-miR-11400	NM_001547	IFIT2	2118	2140	1	1	-0.09	-0.17	22	12	3UTR
hsa-miR-11400	NM_012420	IFIT5	2486	2507	1	1	0.41	0.28	21	7	3UTR
hsa-miR-11400	NM_006435	IFITM2	635	659	1	1	-1.41	-0.79	10	9	3UTR

mirnaid	refseqid	genesymbol	start	end	bindingp	seed	phylopstem	phylopflank	binding_region_length	longest_consecutive_pairings	position
hsa-miR-11400	NM_001384504	IFNAR1	5615	5655	1	1	0.06	-0.08	24	9	3UTR
hsa-miR-11400	NM_000629	IFNAR1	5533	5573	1	1	0.06	-0.08	24	9	3UTR
hsa-miR-11400	NM_025103	IFT74	4849	4869	1	1	0.08	-0.16	20	7	3UTR
hsa-miR-11400	NM_001099222	IFT74	4908	4928	1	1	0.08	-0.16	20	7	3UTR
hsa-miR-11400	NM_001099223	IFT74	4781	4801	1	1	0.64	0.51	20	7	3UTR
hsa-miR-11400	NM_006546	IGF2BP1	6197	6242	1	1	0.26	1.04	21	9	3UTR
hsa-miR-11400	NM_001160423	IGF2BP1	5780	5825	1	1	0.26	1.04	21	9	3UTR
hsa-miR-11400	NM_001291873	IGF2BP2	2135	2156	1	1	1.41	0.73	21	11	3UTR
hsa-miR-11400	NM_006548	IGF2BP2	1935	1960	1	1	0.75	2.07	25	8	3UTR
hsa-miR-11400	NM_000599	IGFBP5	3601	3630	1	1	1.18	0.79	29	9	3UTR
hsa-miR-11400	NM_000599	IGFBP5	5377	5399	1	1	3.18	2.16	22	8	3UTR
hsa-miR-11400	NM_001002915	IGFL2	828	850	1	1	0.13	-0.12	22	10	3UTR
hsa-miR-11400	NM_001135113	IGFL2	545	567	1	1	0.13	-0.11	22	10	3UTR
hsa-miR-11400	NM_001101372	IGLON5	2732	2766	1	1	0.07	-0.19	28	12	3UTR
hsa-miR-11400	NM_001291837	IKZF1	2052	2098	1	1	-0.31	-0.50	20	15	3UTR
hsa-miR-11400	NM_001291838	IKZF1	1917	1963	1	1	-0.31	-0.50	20	15	3UTR
hsa-miR-11400	NM_001291841	IKZF1	1542	1588	1	1	-0.31	-0.50	20	15	3UTR

mirnaid	refseqid	genesymbol	start	end	bindingp	seed	phylopstem	phylopflank	binding_region_length	longest_consecutive_pairings	position
hsa-miR-11400	NM_001291843	IKZF1	1416	1462	1	1	-0.31	-0.50	20	15	3UTR
hsa-miR-11400	XM_011515075	IKZF1	2074	2120	1	1	0.00	0.00	20	15	3UTR
hsa-miR-11400	NM_006060	IKZF1	2178	2224	1	1	-0.31	-0.50	20	15	3UTR
hsa-miR-11400	NM_001220765	IKZF1	2052	2098	1	1	0.00	0.00	20	15	3UTR
hsa-miR-11400	NM_001220768	IKZF1	1710	1756	1	1	-0.31	-0.50	20	15	3UTR
hsa-miR-11400	NM_001220771	IKZF1	1542	1588	1	1	-0.31	-0.50	20	15	3UTR
hsa-miR-11400	XM_005246384	IKZF2	5675	5707	1	1	0.00	0.00	16	14	3UTR
hsa-miR-11400	NM_016260	IKZF2	5668	5700	1	1	0.31	0.26	16	14	3UTR
hsa-miR-11400	NM_001079526	IKZF2	5637	5669	1	1	0.29	0.32	16	14	3UTR
hsa-miR-11400	NM_001284516	IKZF3	5923	5945	0.953846	1	1.50	0.81	22	10	3UTR
hsa-miR-11400	NM_001372123	IKZF5	3614	3635	1	1	5.56	4.07	21	13	3UTR
hsa-miR-11400	NM_000628	IL10RB	1360	1393	1	1	-0.29	-0.25	17	6	3UTR
hsa-miR-11400	NM_002187	IL12B	2005	2034	1	1	0.35	1.92	29	7	3UTR
hsa-miR-11400	NM_001289905	IL17RA	3195	3220	1	1	-0.13	-0.31	25	8	3UTR
hsa-miR-11400	NM_014339	IL17RA	3297	3322	1	1	-0.12	-0.09	25	8	3UTR
hsa-miR-11400	NM_001364879	IL1RAP	2694	2712	1	1	0.05	0.61	18	14	3UTR
hsa-miR-11400	NM_001167931	IL1RAP	2781	2799	1	1	0.05	0.61	18	14	3UTR

mirnaid	refseqid	genesymbol	start	end	bindingp	seed	phylopstem	phylopflank	binding_region_length	longest_consecutive_pairings	position
hsa-miR-11400	NM_173841	IL1RN	679	697	1	1	0.02	-0.12	18	11	3UTR
hsa-miR-11400	NM_173842	IL1RN	649	667	1	1	0.02	-0.12	18	11	3UTR
hsa-miR-11400	NM_173843	IL1RN	787	805	1	1	0.02	-0.12	18	11	3UTR
hsa-miR-11400	XM_011511121	IL1RN	1216	1234	1	1	0.00	0.00	18	11	3UTR
hsa-miR-11400	NM_000577	IL1RN	616	634	1	1	0.02	-0.12	18	11	3UTR
hsa-miR-11400	NM_001278722	IL20RA	1988	2006	1	1	-0.11	-0.17	18	9	3UTR
hsa-miR-11400	NM_181309	IL22RA2	1983	2022	1	1	1.39	1.29	20	8	3UTR
hsa-miR-11400	NM_181310	IL22RA2	1813	1852	1	1	0.52	0.30	20	8	3UTR
hsa-miR-11400	NM_173170	IL36RN	1980	2011	1	1	-0.14	-0.29	24	9	3UTR
hsa-miR-11400	NM_012275	IL36RN	1918	1949	1	1	-0.14	-0.29	24	9	3UTR
hsa-miR-11400	NM_181359	IL6R	2417	2436	0.955128	1	-0.58	-0.58	19	13	3UTR
hsa-miR-11400	NM_000565	IL6R	2511	2530	0.955128	1	-0.58	-0.58	19	13	3UTR
hsa-miR-11400	XM_005248299	IL7R	4182	4204	1	1	0.00	0.00	16	14	3UTR
hsa-miR-11400	NM_002185	IL7R	4276	4298	1	1	0.05	0.08	16	14	3UTR
hsa-miR-11400	NM_175924	ILDR1	2510	2537	1	1	1.24	1.72	27	8	3UTR
hsa-miR-11400	NM_001199800	ILDR1	2375	2402	1	1	2.35	2.76	27	8	3UTR
hsa-miR-11400	XM_017001256	ILDR2	5236	5254	1	1	0.00	0.00	18	11	3UTR

mirnaid	refseqid	genesymbol	start	end	bindingp	seed	phylopstem	phylopflank	binding_region_length	longest_consecutive_pairings	position
hsa-miR-11400	NM_178511	INAFM1	732	750	1	1	0.43	-0.36	18	8	3UTR
hsa-miR-11400	NM_002193	INHBB	1350	1369	1	1	0.01	0.36	19	15	3UTR
hsa-miR-11400	NM_017759	INO80D	7280	7299	1	1	0.00	0.00	19	12	3UTR
hsa-miR-11400	XM_011539528	INPP5F	4439	4458	1	1	0.00	0.00	19	10	3UTR
hsa-miR-11400	NM_014937	INPP5F	4549	4568	1	1	0.17	-0.14	19	10	3UTR
hsa-miR-11400	NM_001243194	INPP5F	2721	2740	1	1	0.00	0.00	19	10	3UTR
hsa-miR-11400	NM_001135642	INPP5K	1878	1896	1	1	2.03	4.00	18	8	3UTR
hsa-miR-11400	NM_001129891	INSYN2B	5115	5137	1	1	0.82	0.81	22	8	3UTR
hsa-miR-11400	NM_018085	IPO9	7090	7115	1	1	0.16	0.00	25	8	3UTR
hsa-miR-11400	NM_001330619	IQSEC1	6610	6633	0.961538	1	4.51	5.91	23	9	3UTR
hsa-miR-11400	NM_001134382	IQSEC1	6967	6990	0.961538	1	4.71	5.32	23	9	3UTR
hsa-miR-11400	XM_024453846	IQSEC1	6506	6529	1	1	0.00	0.00	23	9	3UTR
hsa-miR-11400	XM_024453846	IQSEC1	6270	6292	1	1	0.00	0.00	22	8	3UTR
hsa-miR-11400	NM_014869	IQSEC1	4785	4808	1	1	3.94	3.48	23	9	3UTR
hsa-miR-11400	NM_001376938	IQSEC1	7151	7174	1	1	3.61	2.16	23	9	3UTR
hsa-miR-11400	NM_001570	IRAK2	1915	1933	1	1	0.20	0.86	18	17	3UTR
hsa-miR-11400	NM_016123	IRAK4	2964	2986	1	1	-0.06	0.10	22	9	3UTR

mirnaid	refseqid	genesymbol	start	end	bindingp	seed	phylopstem	phylopflank	binding_region_length	longest_consecutive_pairings	position
hsa-miR-11400	NM_001114182	IRAK4	3012	3034	1	1	0.00	0.09	22	9	3UTR
hsa-miR-11400	NM_001145257	IRAK4	2818	2840	1	1	0.00	0.09	22	9	3UTR
hsa-miR-11400	NM_001145258	IRAK4	2648	2670	1	1	0.00	0.09	22	9	3UTR
hsa-miR-11400	NM_182972	IRF2BP2	4814	4836	1	1	0.00	0.00	22	9	3UTR
hsa-miR-11400	NM_001077397	IRF2BP2	4113	4135	1	1	1.01	1.41	22	9	3UTR
hsa-miR-11400	NM_002460	IRF4	1519	1539	1	1	-0.58	-0.01	20	14	3UTR
hsa-miR-11400	NM_001370152	ISG20L2	1957	1982	1	1	6.82	2.00	25	8	3UTR
hsa-miR-11400	NM_022334	ITGB1BP1	2959	2977	0.961538	1	-0.05	0.15	18	10	3UTR
hsa-miR-11400	NM_002217	ITIH3	2768	2785	1	1	0.15	0.12	17	10	3UTR
hsa-miR-11400	NM_001001851	ITIH5	2928	2947	1	1	0.26	1.25	19	10	3UTR
hsa-miR-11400	NM_001001851	ITIH5	2997	3022	1	1	0.77	-0.08	25	13	3UTR
hsa-miR-11400	NM_021999	ITM2B	9774	9807	1	1	0.06	-0.19	23	7	3UTR
hsa-miR-11400	NM_001363707	ITPK1	4997	5018	1	1	0.00	0.00	21	8	3UTR
hsa-miR-11400	NM_002223	ITPR2	12137	12159	1	1	2.04	2.24	22	11	3UTR
hsa-miR-11400	NM_001287441	JADE1	2874	2894	1	1	0.19	0.01	20	9	3UTR
hsa-miR-11400	NM_024900	JADE1	2936	2956	1	1	0.19	0.01	20	9	3UTR
hsa-miR-11400	NM_001270407	JAM2	1360	1383	1	1	1.37	1.63	20	11	3UTR

mirnaid	refseqid	genesymbol	start	end	bindingp	seed	phylopstem	phylopflank	binding_region_length	longest_consecutive_pairings	position
hsa-miR-11400	NM_021219	JAM2	1468	1491	1	1	1.25	2.38	20	11	3UTR
hsa-miR-11400	NM_024806	JHY	4249	4274	1	1	1.11	1.21	25	9	3UTR
hsa-miR-11400	NM_016475	JKAMP	1839	1857	1	1	0.08	0.18	18	9	3UTR
hsa-miR-11400	NM_153186	KANK1	4369	4386	0.974359	1	5.44	5.07	17	8	3UTR
hsa-miR-11400	NM_015158	KANK1	4354	4371	1	1	0.25	0.37	17	8	3UTR
hsa-miR-11400	XM_017000485	KANK4	1620	1639	1	1	0.00	0.00	19	13	3UTR
hsa-miR-11400	NM_030929	KAZALD1	1398	1442	0.974359	1	-0.13	-0.35	44	14	3UTR
hsa-miR-11400	XM_005245795	KAZN	4125	4147	1	1	0.00	0.00	22	7	3UTR
hsa-miR-11400	XM_011541396	KCNA2	4889	4909	1	1	0.00	0.00	20	11	3UTR
hsa-miR-11400	XM_011541399	KCNA2	4978	4998	1	1	0.00	0.00	20	11	3UTR
hsa-miR-11400	XM_011541400	KCNA2	4908	4928	1	1	0.00	0.00	20	11	3UTR
hsa-miR-11400	XM_017001213	KCNA2	4870	4890	1	1	0.00	0.00	20	11	3UTR
hsa-miR-11400	NM_004976	KCNC1	1705	1726	1	1	2.42	1.73	21	8	3UTR
hsa-miR-11400	NM_004977	KCNC3	4012	4035	1	1	0.48	0.28	23	7	3UTR
hsa-miR-11400	NM_172198	KCND3	5466	5481	1	1	2.02	3.21	15	14	3UTR
hsa-miR-11400	NM_173092	KCNH6	2904	2928	1	1	0.12	0.05	24	8	3UTR
hsa-miR-11400	NM_144633	KCNH8	4810	4831	1	1	0.19	-0.02	21	8	3UTR

mirnaid	refseqid	genesymbol	start	end	bindingp	seed	phylopstem	phylopflank	binding_region_length	longest_consecutive_pairings	position
hsa-miR-11400	NM_001276435	KCNJ15	7875	7908	1	1	0.00	0.00	28	8	3UTR
hsa-miR-11400	NM_001276436	KCNJ15	7878	7911	1	1	0.00	0.00	28	8	3UTR
hsa-miR-11400	NM_170736	KCNJ15	7744	7777	1	1	0.54	1.03	28	8	3UTR
hsa-miR-11400	NM_170737	KCNJ15	7599	7632	1	1	0.00	0.00	28	8	3UTR
hsa-miR-11400	NM_002243	KCNJ15	7736	7769	1	1	0.54	1.03	28	8	3UTR
hsa-miR-11400	NM_001354169	KCNJ5	3474	3494	1	1	0.11	-0.61	20	12	3UTR
hsa-miR-11400	NM_000890	KCNJ5	3385	3405	1	1	0.11	-0.61	20	12	3UTR
hsa-miR-11400	NM_002240	KCNJ6	12588	12605	1	1	0.09	-0.24	17	16	3UTR
hsa-miR-11400	NM_022055	KCNK12	11797	11817	1	1	6.22	4.17	20	10	3UTR
hsa-miR-11400	XM_005264293	KCNK3	2797	2819	1	1	0.00	0.00	22	10	3UTR
hsa-miR-11400	XM_011517102	KCNK9	1607	1628	1	1	0.00	0.00	21	9	3UTR
hsa-miR-11400	NM_172106	KCNQ2	5929	5949	0.980769	1	-0.84	-1.12	20	10	3UTR
hsa-miR-11400	NM_172107	KCNQ2	5983	6003	0.980769	1	-0.81	-0.94	20	10	3UTR
hsa-miR-11400	NM_172108	KCNQ2	5890	5910	0.980769	1	-1.69	-1.04	20	10	3UTR
hsa-miR-11400	NM_004518	KCNQ2	5899	5919	0.980769	1	-0.55	-1.14	20	10	3UTR
hsa-miR-11400	NM_172106	KCNQ2	3025	3044	1	1	-0.50	-0.22	19	9	3UTR
hsa-miR-11400	NM_172107	KCNQ2	3079	3098	1	1	-0.31	-0.13	19	9	3UTR

mirnaid	refseqid	genesymbol	start	end	bindingp	seed	phylopstem	phylopflank	binding_region_length	longest_consecutive_pairings	position
hsa-miR-11400	NM_172108	KCNQ2	2986	3005	1	1	-0.47	-0.16	19	9	3UTR
hsa-miR-11400	XM_011528811	KCNQ2	5973	5993	1	1	0.00	0.00	20	10	3UTR
hsa-miR-11400	XM_011528811	KCNQ2	6768	6791	1	1	0.00	0.00	16	8	3UTR
hsa-miR-11400	NM_001204824	KCNQ3	6527	6548	1	1	0.00	0.00	21	8	3UTR
hsa-miR-11400	NM_172163	KCNQ4	2531	2546	1	1	0.36	0.27	15	14	3UTR
hsa-miR-11400	XM_017002792	KCNQ4	1709	1724	1	1	0.00	0.00	15	14	3UTR
hsa-miR-11400	NM_004700	KCNQ4	2693	2708	1	1	0.36	0.27	15	14	3UTR
hsa-miR-11400	NM_001272003	KCNT1	6220	6240	1	1	-0.12	-0.25	20	13	3UTR
hsa-miR-11400	NM_020822	KCNT1	6292	6312	1	1	-0.12	-0.25	20	13	3UTR
hsa-miR-11400	NM_001287819	KCNT2	3929	3964	1	1	2.88	3.17	35	10	3UTR
hsa-miR-11400	NM_198503	KCNT2	4001	4036	1	1	2.88	3.17	35	10	3UTR
hsa-miR-11400	NM_001286579	KCTD20	957	978	0.961538	1	0.24	0.67	21	7	3UTR
hsa-miR-11400	NM_173562	KCTD20	1455	1476	0.961538	1	0.24	0.67	21	7	3UTR
hsa-miR-11400	NM_018992	KCTD5	733	754	1	1	1.60	2.68	21	8	3UTR
hsa-miR-11400	NM_006854	KDELR2	1755	1765	1	1	3.08	2.54	10	9	3UTR
hsa-miR-11400	NM_001146695	KDM4C	2831	2851	0.980769	1	-0.20	0.14	20	11	3UTR
hsa-miR-11400	NM_001348716	KDM6B	5809	5860	1	1	1.37	1.28	24	7	3UTR

mirnaid	refseqid	genesymbol	start	end	bindingp	seed	phylopstem	phylopflank	binding_region_length	longest_consecutive_pairings	position
hsa-miR-11400	NM_001080424	KDM6B	5781	5832	1	1	1.10	1.40	24	7	3UTR
hsa-miR-11400	XM_005266677	KDSR	1790	1808	1	1	0.00	0.00	18	8	3UTR
hsa-miR-11400	XM_017004060	KHK	1679	1723	1	1	0.00	0.00	21	9	3UTR
hsa-miR-11400	NM_014743	KIAA0232	5874	5894	1	1	1.53	0.63	20	12	3UTR
hsa-miR-11400	NM_001100590	KIAA0232	5790	5810	1	1	1.44	0.78	20	12	3UTR
hsa-miR-11400	NM_014702	KIAA0408	6113	6161	1	1	3.46	3.40	16	14	3UTR
hsa-miR-11400	XM_006721612	KIAA0753	3201	3222	1	1	0.00	0.00	21	8	3UTR
hsa-miR-11400	NM_014804	KIAA0753	3542	3563	1	1	2.64	2.60	21	8	3UTR
hsa-miR-11400	NM_020444	KIAA1191	1937	1958	1	1	2.21	2.82	21	9	3UTR
hsa-miR-11400	NM_001079684	KIAA1191	1850	1871	1	1	2.08	2.38	21	9	3UTR
hsa-miR-11400	NM_001079685	KIAA1191	1829	1850	1	1	1.90	2.31	21	9	3UTR
hsa-miR-11400	NM_020721	KIAA1210	7578	7599	1	1	0.00	0.00	21	9	3UTR
hsa-miR-11400	XM_011518311	KIAA1958	9665	9684	1	1	0.00	0.00	19	12	3UTR
hsa-miR-11400	NM_006612	KIF1C	4401	4419	1	1	-0.29	-0.42	18	14	3UTR
hsa-miR-11400	NM_004520	KIF2A	6231	6256	1	1	0.00	0.00	25	8	3UTR
hsa-miR-11400	NM_001243953	KIF2A	6174	6199	1	1	0.00	0.00	25	8	3UTR
hsa-miR-11400	NM_004798	KIF3B	3450	3488	1	1	0.34	0.26	38	12	3UTR

mirnaid	refseqid	genesymbol	start	end	bindingp	seed	phylopstem	phylopflank	binding_region_length	longest_consecutive_pairings	position
hsa-miR-11400	NM_004984	KIF5A	5464	5483	1	1	0.37	0.56	19	10	3UTR
hsa-miR-11400	NM_001354705	KIF5A	5197	5216	1	1	0.37	0.56	19	10	3UTR
hsa-miR-11400	NM_001289024	KIF6	1815	1836	1	1	-0.03	0.19	21	9	3UTR
hsa-miR-11400	NM_145027	KIF6	3329	3350	1	1	0.52	-0.04	21	9	3UTR
hsa-miR-11400	NM_182902	KIF9	2797	2819	1	1	1.20	3.27	22	14	3UTR
hsa-miR-11400	NM_001281972	KIR2DS4	1065	1082	1	1	0.08	-0.22	17	6	3UTR
hsa-miR-11400	NM_001286349	KIRREL1	4467	4487	1	1	0.76	0.08	20	10	3UTR
hsa-miR-11400	NM_018240	KIRREL1	4767	4787	1	1	0.05	-0.30	20	10	3UTR
hsa-miR-11400	NM_007249	KLF12	3711	3736	1	1	-0.06	0.36	25	9	3UTR
hsa-miR-11400	NM_007249	KLF12	2658	2688	1	1	0.04	-0.08	22	10	3UTR
hsa-miR-11400	NM_001271865	KLHDC8A	1739	1772	1	1	4.02	3.65	33	8	3UTR
hsa-miR-11400	NM_030624	KLHL15	3462	3487	1	1	1.59	2.53	25	10	3UTR
hsa-miR-11400	NM_032775	KLHL22	2381	2427	1	1	1.51	2.24	21	11	3UTR
hsa-miR-11400	NM_001308112	KLHL28	4745	4770	0.961538	1	4.75	5.02	25	8	3UTR
hsa-miR-11400	NM_001257195	KLHL3	4480	4496	1	1	-0.14	-0.16	16	9	3UTR
hsa-miR-11400	NM_020782	KLHL42	4085	4109	1	1	0.07	-0.07	24	8	3UTR
hsa-miR-11400	NM_001256080	KLK2	2281	2317	1	1	0.04	0.05	19	9	3UTR

mirnaid	refseqid	genesymbol	start	end	bindingp	seed	phylopstem	phylopflank	binding_region_length	longest_consecutive_pairings	position
hsa-miR-11400	NM_002262	KLRD1	13862	13882	1	1	-0.10	0.08	20	11	3UTR
hsa-miR-11400	NM_001351063	KLRD1	13799	13819	1	1	-0.10	0.08	20	11	3UTR
hsa-miR-11400	NM_007360	KLRK1	1195	1235	1	1	0.81	0.22	24	7	3UTR
hsa-miR-11400	NM_001366306	KPNA5	3476	3494	1	1	0.65	0.85	18	13	3UTR
hsa-miR-11400	NM_001366306	KPNA5	9344	9361	1	1	-0.09	0.05	17	12	3UTR
hsa-miR-11400	NM_033360	KRAS	4038	4059	1	1	1.18	1.32	21	9	3UTR
hsa-miR-11400	NM_033360	KRAS	3575	3597	1	1	0.52	0.44	22	8	3UTR
hsa-miR-11400	NM_004985	KRAS	3914	3935	1	1	0.63	1.80	21	9	3UTR
hsa-miR-11400	NM_001039570	KREMEN1	3806	3831	1	1	-0.20	0.15	25	9	3UTR
hsa-miR-11400	NM_001039570	KREMEN1	5182	5204	1	1	0.85	0.13	22	9	3UTR
hsa-miR-11400	NM_007043	KRR1	4341	4360	1	1	-0.35	0.03	19	9	3UTR
hsa-miR-11400	NM_001300810	KRT85	1264	1301	1	1	2.64	2.22	37	8	3UTR
hsa-miR-11400	NM_031957	KRTAP1-5	763	786	1	1	1.55	0.70	23	8	3UTR
hsa-miR-11400	XM_005267161	L3MBTL3	3505	3524	1	1	0.00	0.00	19	11	3UTR
hsa-miR-11400	NM_001365767	L3MBTL4	1852	1874	1	1	-0.04	0.17	22	8	3UTR
hsa-miR-11400	NM_018697	LANCL2	2596	2612	1	1	-0.88	-0.54	16	15	3UTR
hsa-miR-11400	NM_018407	LAPTM4B	1554	1573	1	1	-0.52	-0.71	19	9	3UTR

mirnaid	refseqid	genesymbol	start	end	bindingp	seed	phylopstem	phylopflank	binding_region_length	longest_consecutive_pairings	position
hsa-miR-11400	NM_032239	LARP1B	1762	1782	1	1	1.48	2.12	20	16	3UTR
hsa-miR-11400	NM_052879	LARP4	2583	2600	0.974359	1	2.90	2.85	17	8	3UTR
hsa-miR-11400	NM_199188	LARP4	2580	2597	0.974359	1	2.90	2.85	17	8	3UTR
hsa-miR-11400	NM_199190	LARP4	2370	2387	0.974359	1	2.90	2.85	17	8	3UTR
hsa-miR-11400	NM_001330415	LARP4	2601	2618	0.974359	1	2.90	2.85	17	8	3UTR
hsa-miR-11400	NM_030915	LBH	977	994	1	1	0.59	0.00	17	9	3UTR
hsa-miR-11400	NM_004139	LBP	1482	1517	1	1	-0.04	0.70	21	15	3UTR
hsa-miR-11400	NM_001384302	LCA5L	1411	1425	1	1	3.69	0.53	14	13	3UTR
hsa-miR-11400	NM_001365660	LCORL	2021	2039	1	1	0.68	0.41	18	12	3UTR
hsa-miR-11400	NM_001282719	LDAH	1130	1148	1	1	0.14	0.00	18	13	3UTR
hsa-miR-11400	NM_001282720	LDAH	1112	1130	1	1	0.24	0.00	18	13	3UTR
hsa-miR-11400	NM_001282721	LDAH	1100	1118	1	1	-0.95	0.19	18	13	3UTR
hsa-miR-11400	NM_001282723	LDAH	1021	1039	1	1	-0.15	-0.05	18	13	3UTR
hsa-miR-11400	NM_003893	LDB1	3093	3114	1	1	1.73	1.61	21	10	3UTR
hsa-miR-11400	NM_001013693	LDLRAD2	1594	1638	1	1	0.34	-0.01	37	9	3UTR
hsa-miR-11400	XM_024451250	LDLRAD4	5397	5416	1	1	0.00	0.00	19	12	3UTR
hsa-miR-11400	NM_014319	LEMD3	3482	3508	1	1	1.08	1.98	26	14	3UTR

mirnaid	refseqid	genesymbol	start	end	bindingp	seed	phylopstem	phylopflank	binding_region_length	longest_consecutive_pairings	position
hsa-miR-11400	XM_017026428	LGI4	2105	2127	1	1	0.00	0.00	22	10	3UTR
hsa-miR-11400	NM_005779	LHFPL2	2620	2637	1	1	-0.24	0.23	17	8	3UTR
hsa-miR-11400	NM_198560	LHFPL4	3187	3208	1	1	0.28	-0.17	21	10	3UTR
hsa-miR-11400	NM_005780	LHFPL6	1942	1960	1	1	0.02	-0.30	18	7	3UTR
hsa-miR-11400	NM_001348190	LHX6	1681	1703	1	1	2.70	2.55	22	8	3UTR
hsa-miR-11400	NM_001242333	LHX6	2249	2270	1	1	0.85	0.78	21	9	3UTR
hsa-miR-11400	NM_001278590	LIAS	3378	3403	1	1	-0.12	0.03	25	8	3UTR
hsa-miR-11400	NM_194451	LIAS	3395	3420	1	1	-0.12	0.03	25	8	3UTR
hsa-miR-11400	NM_006859	LIAS	3507	3532	1	1	-0.12	0.03	25	8	3UTR
hsa-miR-11400	NM_001363700	LIAS	3198	3223	1	1	-0.12	0.03	25	8	3UTR
hsa-miR-11400	NM_001113547	LIMA1	3371	3393	1	1	2.18	2.37	22	12	3UTR
hsa-miR-11400	NM_001243775	LIMA1	3127	3167	1	1	3.03	1.71	23	12	3UTR
hsa-miR-11400	NM_024674	LIN28A	1589	1603	1	1	1.16	1.42	14	13	3UTR
hsa-miR-11400	XM_005262750	LIN54	5038	5055	0.961538	1	0.00	0.00	17	7	3UTR
hsa-miR-11400	NM_004664	LIN7A	4224	4239	1	1	0.83	0.59	15	14	3UTR
hsa-miR-11400	NM_001288979	LIPA	1483	1531	1	1	3.17	2.39	19	12	3UTR
hsa-miR-11400	NM_000235	LIPA	1713	1761	1	1	1.65	2.04	19	12	3UTR

mirnaid	refseqid	genesymbol	start	end	bindingp	seed	phylopstem	phylopflank	binding_region_length	longest_consecutive_pairings	position
hsa-miR-11400	NM_001308006	LIPG	4467	4485	1	1	0.11	0.06	18	17	3UTR
hsa-miR-11400	NM_006033	LIPG	4689	4707	1	1	0.11	0.06	18	17	3UTR
hsa-miR-11400	NM_001102469	LIPN	1391	1410	1	1	0.03	0.08	19	13	3UTR
hsa-miR-11400	NM_001136473	LITAF	537	558	1	1	0.35	-0.18	21	11	3UTR
hsa-miR-11400	NM_001136473	LITAF	1923	1937	1	1	3.64	2.05	14	13	3UTR
hsa-miR-11400	NM_001278233	LMCD1	3651	3673	1	1	-0.21	0.00	22	8	3UTR
hsa-miR-11400	NM_001278234	LMCD1	3484	3506	1	1	0.00	0.00	22	8	3UTR
hsa-miR-11400	NM_014583	LMCD1	3740	3762	1	1	-0.21	0.00	22	8	3UTR
hsa-miR-11400	NM_014916	LMTK2	5960	5989	1	1	-0.54	-0.54	20	10	3UTR
hsa-miR-11400	NM_177398	LMX1A	3044	3066	1	1	4.37	3.25	22	8	3UTR
hsa-miR-11400	NM_175920	LNPEP	4895	4915	1	1	0.75	0.70	20	7	3UTR
hsa-miR-11400	NM_005575	LNPEP	4646	4666	1	1	0.75	0.70	20	7	3UTR
hsa-miR-11400	NM_030650	LNPK	6796	6832	1	1	2.06	2.67	36	9	3UTR
hsa-miR-11400	NM_001004352	LOC100506422	2151	2194	1	1	0.51	0.37	21	12	3UTR
hsa-miR-11400	NM_001319657	LOC102724265	1056	1081	1	1	-0.09	-0.18	19	8	3UTR
hsa-miR-11400	NM_001370182	LOC102724488	3999	4022	1	1	0.12	-0.10	23	10	3UTR
hsa-miR-11400	NM_001370184	LOC102724488	4204	4227	1	1	0.14	0.10	23	10	3UTR

mirnaid	refseqid	genesymbol	start	end	bindingp	seed	phylopstem	phylopflank	binding_region_length	longest_consecutive_pairings	position
hsa-miR-11400	XM_005255752	LOC107983990	1124	1173	1	1	0.00	0.00	18	16	3UTR
hsa-miR-11400	NM_002317	LOX	3068	3087	1	1	0.66	0.88	19	11	3UTR
hsa-miR-11400	NM_005296	LPAR4	2352	2376	0.961538	1	0.61	0.40	15	13	3UTR
hsa-miR-11400	NM_001278000	LPAR4	2567	2591	1	1	0.00	0.00	15	13	3UTR
hsa-miR-11400	NM_014646	LPIN2	5767	5787	1	1	3.27	5.42	20	11	3UTR
hsa-miR-11400	NM_005578	LPP	17761	17785	1	1	-0.04	0.19	24	9	3UTR
hsa-miR-11400	NM_001375462	LPP	17763	17787	1	1	-0.04	0.19	24	9	3UTR
hsa-miR-11400	NM_020871	LRCH2	3381	3402	1	1	2.28	2.69	21	8	3UTR
hsa-miR-11400	NM_001243963	LRCH2	3330	3351	1	1	2.28	2.69	21	8	3UTR
hsa-miR-11400	NM_014813	LRIG2	11401	11428	0.961538	1	0.29	0.38	17	15	3UTR
hsa-miR-11400	NM_033300	LRP8	5873	5902	1	1	4.96	3.35	29	9	3UTR
hsa-miR-11400	NM_017522	LRP8	5594	5623	1	1	5.11	3.21	29	9	3UTR
hsa-miR-11400	NM_133259	LRPPRC	5548	5567	1	1	3.81	3.12	19	15	3UTR
hsa-miR-11400	NM_005824	LRRC17	1581	1628	0.953846	1	2.91	2.78	19	17	3UTR
hsa-miR-11400	NM_030626	LRRC27	6686	6710	1	1	0.11	-0.01	24	8	3UTR
hsa-miR-11400	NM_001143757	LRRC27	6966	6990	1	1	0.11	-0.01	24	8	3UTR
hsa-miR-11400	NM_005512	LRRC32	2421	2435	1	1	4.41	2.86	14	13	3UTR

mirnaid	refseqid	genesymbol	start	end	bindingp	seed	phylopstem	phylopflank	binding_region_length	longest_consecutive_pairings	position
hsa-miR-11400	XM_005264850	LRRC3B	2850	2871	1	1	0.00	0.00	21	11	3UTR
hsa-miR-11400	XM_005264850	LRRC3B	3371	3391	1	1	0.00	0.00	20	7	3UTR
hsa-miR-11400	NM_006369	LRRC41	3504	3550	1	1	2.75	2.77	22	10	3UTR
hsa-miR-11400	NM_001005210	LRRC55	3797	3813	1	1	0.03	0.51	16	15	3UTR
hsa-miR-11400	NM_018509	LRRC59	1620	1651	1	1	1.04	1.55	31	8	3UTR
hsa-miR-11400	NM_207387	LRRC75A	2492	2514	1	1	5.19	3.09	22	8	3UTR
hsa-miR-11400	NM_001113567	LRRC75A	2608	2630	1	1	5.29	3.33	22	8	3UTR
hsa-miR-11400	NM_032270	LRRC8C	3468	3488	1	1	0.51	1.14	20	12	3UTR
hsa-miR-11400	NM_001137550	LRRFIP1	3678	3702	1	1	-0.24	-0.29	18	16	3UTR
hsa-miR-11400	NM_020873	LRRN1	5096	5116	1	1	0.02	-0.22	20	9	3UTR
hsa-miR-11400	NM_001039029	LRTM2	2866	2888	1	1	0.77	-0.27	20	14	3UTR
hsa-miR-11400	NM_001163926	LRTM2	2869	2891	1	1	0.77	-0.27	20	14	3UTR
hsa-miR-11400	NM_001145308	LRTOMT	2922	2942	1	1	-0.57	1.86	20	10	3UTR
hsa-miR-11400	NM_152892	LRWD1	2059	2076	1	1	-0.17	0.29	17	16	3UTR
hsa-miR-11400	NM_018385	LSG1	2121	2136	0.961538	1	1.95	3.08	15	14	3UTR
hsa-miR-11400	NM_144703	LSM14B	1705	1750	1	1	1.87	0.73	17	15	3UTR
hsa-miR-11400	NM_001145436	LSS	3025	3071	1	1	1.62	4.05	29	8	3UTR

mirnaid	refseqid	genesymbol	start	end	bindingp	seed	phylopstem	phylopflank	binding_region_length	longest_consecutive_pairings	position
hsa-miR-11400	NM_000595	LTA	1081	1101	1	1	0.38	0.10	20	9	3UTR
hsa-miR-11400	NM_001159740	LTA	1127	1147	1	1	0.38	0.10	20	9	3UTR
hsa-miR-11400	NM_001142546	LUZP1	4260	4281	0.961538	1	0.53	0.31	21	13	3UTR
hsa-miR-11400	XM_011542091	LUZP1	4746	4767	1	1	0.00	0.00	21	13	3UTR
hsa-miR-11400	NM_003695	LY6D	585	627	1	1	0.06	-0.49	18	16	3UTR
hsa-miR-11400	NM_182573	LYPD5	2314	2332	1	1	0.80	0.60	18	12	3UTR
hsa-miR-11400	NM_001031749	LYPD5	2350	2368	1	1	0.89	1.06	18	12	3UTR
hsa-miR-11400	NM_020408	LYRM4	993	1040	1	1	0.50	1.12	21	15	3UTR
hsa-miR-11400	NM_001164841	LYRM4	1145	1165	1	1	-0.38	0.45	20	15	3UTR
hsa-miR-11400	NM_001293735	LYRM7	2677	2695	1	1	-0.16	-0.08	18	17	3UTR
hsa-miR-11400	NM_181705	LYRM7	2759	2777	1	1	-0.16	-0.08	18	17	3UTR
hsa-miR-11400	NM_021020	LZTS1	4654	4673	1	1	2.77	1.70	19	9	3UTR
hsa-miR-11400	XM_005264152	M1AP	1792	1812	1	1	0.00	0.00	20	8	3UTR
hsa-miR-11400	NM_002355	M6PR	1033	1051	1	1	0.34	0.77	18	9	3UTR
hsa-miR-11400	NM_001282921	MAB21L4	1377	1397	1	1	1.94	0.94	20	9	3UTR
hsa-miR-11400	NM_024861	MAB21L4	1320	1340	1	1	0.78	1.05	20	9	3UTR
hsa-miR-11400	NM_182762	MACC1	7443	7464	1	1	2.76	1.95	21	7	3UTR

mirnaid	refseqid	genesymbol	start	end	bindingp	seed	phylopstem	phylopflank	binding_region_length	longest_consecutive_pairings	position
hsa-miR-11400	NM_001304524	MAD1L1	2085	2105	1	1	1.70	0.66	20	15	3UTR
hsa-miR-11400	NM_003550	MAD1L1	2463	2483	1	1	-0.22	0.28	20	15	3UTR
hsa-miR-11400	NM_001013837	MAD1L1	2423	2443	1	1	0.45	1.41	20	15	3UTR
hsa-miR-11400	NM_002358	MAD2L1	3389	3407	1	1	0.13	-0.06	18	11	3UTR
hsa-miR-11400	NM_012323	MAFF	988	1032	1	1	-0.18	-0.35	23	9	3UTR
hsa-miR-11400	NM_001161572	MAFF	1014	1058	1	1	0.34	-0.18	23	9	3UTR
hsa-miR-11400	NM_001161573	MAFF	834	878	1	1	0.00	0.00	23	9	3UTR
hsa-miR-11400	NM_001161574	MAFF	921	965	1	1	0.34	-0.18	23	9	3UTR
hsa-miR-11400	XM_006715773	MAFK	916	937	1	1	0.00	0.00	21	10	3UTR
hsa-miR-11400	XM_011545567	MAGEB17	2324	2346	1	1	0.00	0.00	22	11	3UTR
hsa-miR-11400	NM_177456	MAGEC3	1637	1657	1	1	0.00	0.00	20	6	3UTR
hsa-miR-11400	XM_011531267	MAGEC3	1473	1493	1	1	0.00	0.00	20	6	3UTR
hsa-miR-11400	NM_032509	MAK16	2070	2109	1	1	0.02	0.06	20	10	3UTR
hsa-miR-11400	NM_173844	MALT1	4845	4865	1	1	-0.02	0.28	20	12	3UTR
hsa-miR-11400	NM_006785	MALT1	4878	4898	1	1	-0.02	0.28	20	12	3UTR
hsa-miR-11400	NM_001385185	MAN1C1	1383	1401	1	1	0.31	0.51	18	11	3UTR
hsa-miR-11400	XM_017028006	MANBAL	1633	1673	1	1	0.00	0.00	25	7	3UTR

mirnaid	refseqid	genesymbol	start	end	bindingp	seed	phylopstem	phylopflank	binding_region_length	longest_consecutive_pairings	position
hsa-miR-11400	NM_001369742	MANBAL	756	796	1	1	0.03	0.18	25	7	3UTR
hsa-miR-11400	NM_001376532	MANBAL	807	847	1	1	0.03	0.18	25	7	3UTR
hsa-miR-11400	NM_001003897	MANBAL	451	491	1	1	0.03	0.18	25	7	3UTR
hsa-miR-11400	NM_000240	MAOA	2977	2994	1	1	0.35	0.32	17	8	3UTR
hsa-miR-11400	NM_002758	MAP2K6	10473	10499	1	1	0.10	0.02	21	7	3UTR
hsa-miR-11400	NM_001330450	MAP2K6	10593	10619	1	1	0.10	0.02	21	7	3UTR
hsa-miR-11400	NM_203351	MAP3K3	4224	4249	1	1	0.43	0.41	25	11	3UTR
hsa-miR-11400	NM_002401	MAP3K3	4131	4156	1	1	0.43	0.41	25	11	3UTR
hsa-miR-11400	NM_001330431	MAP3K3	4119	4144	1	1	0.43	0.41	25	11	3UTR
hsa-miR-11400	NM_001363768	MAP3K3	4212	4237	1	1	0.43	0.41	25	11	3UTR
hsa-miR-11400	NM_030885	MAP4	1981	1997	1	1	0.34	1.23	16	15	3UTR
hsa-miR-11400	NM_001270425	MAP4K3	3936	3961	1	1	4.16	3.70	25	10	3UTR
hsa-miR-11400	NM_003618	MAP4K3	3999	4024	1	1	4.16	3.70	25	10	3UTR
hsa-miR-11400	NM_024597	MAP7D3	3984	4004	0.980769	1	1.60	1.50	20	7	3UTR
hsa-miR-11400	NM_002745	MAPK1	4111	4157	1	1	1.97	0.92	16	14	3UTR
hsa-miR-11400	NM_001318067	MAPK10	3519	3547	1	1	0.34	0.42	28	8	3UTR
hsa-miR-11400	NM_138982	MAPK10	5427	5446	1	1	0.18	0.20	19	12	3UTR

mirnaid	refseqid	genesymbol	start	end	bindingp	seed	phylopstem	phylopflank	binding_region_length	longest_consecutive_pairings	position
hsa-miR-11400	XM_005263129	MAPK10	5198	5217	1	1	0.00	0.00	19	12	3UTR
hsa-miR-11400	XM_005263129	MAPK10	3686	3714	1	1	0.00	0.00	28	8	3UTR
hsa-miR-11400	XM_005263130	MAPK10	5193	5212	1	1	0.00	0.00	19	12	3UTR
hsa-miR-11400	XM_005263130	MAPK10	3681	3709	1	1	0.00	0.00	28	8	3UTR
hsa-miR-11400	XM_005263131	MAPK10	5434	5453	1	1	0.00	0.00	19	12	3UTR
hsa-miR-11400	XM_005263131	MAPK10	3922	3950	1	1	0.00	0.00	28	8	3UTR
hsa-miR-11400	XM_005263135	MAPK10	5439	5458	1	1	0.00	0.00	19	12	3UTR
hsa-miR-11400	XM_005263135	MAPK10	3927	3955	1	1	0.00	0.00	28	8	3UTR
hsa-miR-11400	XM_006714268	MAPK10	5363	5382	1	1	0.00	0.00	19	12	3UTR
hsa-miR-11400	XM_006714268	MAPK10	3851	3879	1	1	0.00	0.00	28	8	3UTR
hsa-miR-11400	XM_006714269	MAPK10	5439	5458	1	1	0.00	0.00	19	12	3UTR
hsa-miR-11400	XM_006714269	MAPK10	3927	3955	1	1	0.00	0.00	28	8	3UTR
hsa-miR-11400	XM_011532118	MAPK10	5289	5308	1	1	0.00	0.00	19	12	3UTR
hsa-miR-11400	XM_011532118	MAPK10	3777	3805	1	1	0.00	0.00	28	8	3UTR
hsa-miR-11400	XM_011532120	MAPK10	5259	5278	1	1	0.00	0.00	19	12	3UTR
hsa-miR-11400	XM_011532120	MAPK10	3747	3775	1	1	0.00	0.00	28	8	3UTR
hsa-miR-11400	XM_011532121	MAPK10	5125	5144	1	1	0.00	0.00	19	12	3UTR

mirnaid	refseqid	genesymbol	start	end	bindingp	seed	phylopstem	phylopflank	binding_region_length	longest_consecutive_pairings	position
hsa-miR-11400	XM_011532121	MAPK10	3613	3641	1	1	0.00	0.00	28	8	3UTR
hsa-miR-11400	XM_017008420	MAPK10	5820	5839	1	1	0.00	0.00	19	12	3UTR
hsa-miR-11400	XM_017008420	MAPK10	4308	4336	1	1	0.00	0.00	28	8	3UTR
hsa-miR-11400	XM_017008423	MAPK10	5108	5127	1	1	0.00	0.00	19	12	3UTR
hsa-miR-11400	XM_017008423	MAPK10	3596	3624	1	1	0.00	0.00	28	8	3UTR
hsa-miR-11400	XM_017008427	MAPK10	5455	5474	1	1	0.00	0.00	19	12	3UTR
hsa-miR-11400	XM_017008427	MAPK10	3943	3971	1	1	0.00	0.00	28	8	3UTR
hsa-miR-11400	XM_017008429	MAPK10	5468	5487	1	1	0.00	0.00	19	12	3UTR
hsa-miR-11400	XM_017008429	MAPK10	3956	3984	1	1	0.00	0.00	28	8	3UTR
hsa-miR-11400	XM_017008430	MAPK10	5203	5222	1	1	0.00	0.00	19	12	3UTR
hsa-miR-11400	XM_017008430	MAPK10	3691	3719	1	1	0.00	0.00	28	8	3UTR
hsa-miR-11400	XM_017008433	MAPK10	5383	5402	1	1	0.00	0.00	19	12	3UTR
hsa-miR-11400	XM_017008433	MAPK10	3871	3899	1	1	0.00	0.00	28	8	3UTR
hsa-miR-11400	XM_017008434	MAPK10	5466	5485	1	1	0.00	0.00	19	12	3UTR
hsa-miR-11400	XM_017008434	MAPK10	3954	3982	1	1	0.00	0.00	28	8	3UTR
hsa-miR-11400	XM_017008435	MAPK10	5368	5387	1	1	0.00	0.00	19	12	3UTR
hsa-miR-11400	XM_017008435	MAPK10	3856	3884	1	1	0.00	0.00	28	8	3UTR

mirnaid	refseqid	genesymbol	start	end	bindingp	seed	phylopstem	phylopflank	binding_region_length	longest_consecutive_pairings	position
hsa-miR-11400	XM_017008436	MAPK10	5113	5132	1	1	0.00	0.00	19	12	3UTR
hsa-miR-11400	XM_017008436	MAPK10	3601	3629	1	1	0.00	0.00	28	8	3UTR
hsa-miR-11400	XM_017008437	MAPK10	5130	5149	1	1	0.00	0.00	19	12	3UTR
hsa-miR-11400	XM_017008437	MAPK10	3618	3646	1	1	0.00	0.00	28	8	3UTR
hsa-miR-11400	XM_017008441	MAPK10	5388	5407	1	1	0.00	0.00	19	12	3UTR
hsa-miR-11400	XM_017008441	MAPK10	3876	3904	1	1	0.00	0.00	28	8	3UTR
hsa-miR-11400	XM_017008445	MAPK10	5257	5276	1	1	0.00	0.00	19	12	3UTR
hsa-miR-11400	XM_017008445	MAPK10	3745	3773	1	1	0.00	0.00	28	8	3UTR
hsa-miR-11400	XM_017008451	MAPK10	5262	5281	1	1	0.00	0.00	19	12	3UTR
hsa-miR-11400	XM_017008451	MAPK10	3750	3778	1	1	0.00	0.00	28	8	3UTR
hsa-miR-11400	XM_017008452	MAPK10	5400	5419	1	1	0.00	0.00	19	12	3UTR
hsa-miR-11400	XM_017008452	MAPK10	3888	3916	1	1	0.00	0.00	28	8	3UTR
hsa-miR-11400	XM_024454140	MAPK10	5381	5400	1	1	0.00	0.00	19	12	3UTR
hsa-miR-11400	XM_024454140	MAPK10	3869	3897	1	1	0.00	0.00	28	8	3UTR
hsa-miR-11400	XM_024454141	MAPK10	5899	5918	1	1	0.00	0.00	19	12	3UTR
hsa-miR-11400	XM_024454141	MAPK10	4387	4415	1	1	0.00	0.00	28	8	3UTR
hsa-miR-11400	XM_024454142	MAPK10	5270	5289	1	1	0.00	0.00	19	12	3UTR

mirnaid	refseqid	genesymbol	start	end	bindingp	seed	phylopstem	phylopflank	binding_region_length	longest_consecutive_pairings	position
hsa-miR-11400	XM_024454142	MAPK10	3758	3786	1	1	0.00	0.00	28	8	3UTR
hsa-miR-11400	XM_024454144	MAPK10	5341	5360	1	1	0.00	0.00	19	12	3UTR
hsa-miR-11400	XM_024454144	MAPK10	3829	3857	1	1	0.00	0.00	28	8	3UTR
hsa-miR-11400	XM_024454146	MAPK10	5131	5150	1	1	0.00	0.00	19	12	3UTR
hsa-miR-11400	XM_024454146	MAPK10	3619	3647	1	1	0.00	0.00	28	8	3UTR
hsa-miR-11400	XM_024454147	MAPK10	5099	5118	1	1	0.00	0.00	19	12	3UTR
hsa-miR-11400	XM_024454147	MAPK10	3587	3615	1	1	0.00	0.00	28	8	3UTR
hsa-miR-11400	NM_002753	MAPK10	5202	5221	1	1	0.28	0.90	19	12	3UTR
hsa-miR-11400	NM_001351624	MAPK10	5444	5463	1	1	0.32	0.29	19	12	3UTR
hsa-miR-11400	NM_001363657	MAPK10	5459	5478	1	1	0.04	0.16	19	12	3UTR
hsa-miR-11400	NM_002754	MAPK13	1988	2009	1	1	1.56	0.31	21	8	3UTR
hsa-miR-11400	XM_011532055	MARCHF1	3768	3790	1	1	0.00	0.00	22	9	3UTR
hsa-miR-11400	XM_011532055	MARCHF1	2772	2787	1	1	0.00	0.00	15	8	3UTR
hsa-miR-11400	XM_017008334	MARCHF1	2988	3010	1	1	0.00	0.00	22	9	3UTR
hsa-miR-11400	XM_017008334	MARCHF1	1992	2007	1	1	0.00	0.00	15	8	3UTR
hsa-miR-11400	NM_017923	MARCHF1	1862	1877	1	1	-0.04	0.32	15	8	3UTR
hsa-miR-11400	NM_001166373	MARCHF1	3323	3345	1	1	-0.25	0.19	22	9	3UTR

mirnaid	refseqid	genesymbol	start	end	bindingp	seed	phylopstem	phylopflank	binding_region_length	longest_consecutive_pairings	position
hsa-miR-11400	NM_001166373	MARCHF1	2322	2342	1	1	0.26	-0.09	20	8	3UTR
hsa-miR-11400	NM_001270660	MARCHF6	8310	8328	1	1	0.43	-0.07	18	11	3UTR
hsa-miR-11400	NM_001270661	MARCHF6	8139	8157	1	1	0.43	-0.07	18	11	3UTR
hsa-miR-11400	NM_005885	MARCHF6	8454	8472	1	1	0.43	-0.07	18	11	3UTR
hsa-miR-11400	NM_002377	MAS1	9445	9465	1	1	-0.24	-0.01	20	10	3UTR
hsa-miR-11400	NM_001366704	MAS1	9288	9308	1	1	0.00	0.00	20	10	3UTR
hsa-miR-11400	NM_005911	MAT2A	2319	2336	1	1	5.00	2.39	17	8	3UTR
hsa-miR-11400	NM_020746	MAVS	2737	2757	1	1	-0.53	-0.04	20	14	3UTR
hsa-miR-11400	NM_001206491	MAVS	2562	2582	1	1	0.00	0.00	20	14	3UTR
hsa-miR-11400	NM_001206491	MAVS	3998	4023	1	1	0.00	0.00	25	11	3UTR
hsa-miR-11400	XM_005258271	MBD1	3704	3725	1	1	0.00	0.00	21	11	3UTR
hsa-miR-11400	XM_011525993	MBD1	2129	2150	1	1	0.00	0.00	21	11	3UTR
hsa-miR-11400	XM_017025770	MBD1	3536	3557	1	1	0.00	0.00	21	11	3UTR
hsa-miR-11400	NM_001204137	MBD1	3807	3828	1	1	0.00	0.00	21	11	3UTR
hsa-miR-11400	NM_133486	MBNL3	2717	2754	1	1	2.51	2.78	18	8	3UTR
hsa-miR-11400	XM_024452402	MBNL3	3524	3561	1	1	0.00	0.00	18	8	3UTR
hsa-miR-11400	NM_018388	MBNL3	2748	2785	1	1	0.00	0.00	18	8	3UTR

mirnaid	refseqid	genesymbol	start	end	bindingp	seed	phylopstem	phylopflank	binding_region_length	longest_consecutive_pairings	position
hsa-miR-11400	NM_001170701	MBNL3	2618	2655	1	1	0.88	1.48	18	8	3UTR
hsa-miR-11400	NM_001170702	MBNL3	2582	2619	1	1	1.05	0.87	18	8	3UTR
hsa-miR-11400	NM_001170703	MBNL3	2518	2555	1	1	1.45	1.01	18	8	3UTR
hsa-miR-11400	NM_001170704	MBNL3	2852	2889	1	1	1.66	1.63	18	8	3UTR
hsa-miR-11400	NM_138799	MBOAT2	6283	6311	1	1	3.70	3.39	18	16	3UTR
hsa-miR-11400	NM_005913	MC5R	1431	1457	1	1	-0.02	-0.46	20	8	3UTR
hsa-miR-11400	NM_002387	MCC	5220	5242	1	1	3.82	3.18	22	8	3UTR
hsa-miR-11400	NM_139279	MCFD2	1316	1356	1	1	0.09	0.07	34	10	3UTR
hsa-miR-11400	NM_032503	MCHR2	2220	2245	1	1	0.00	0.00	25	8	3UTR
hsa-miR-11400	NM_001281520	MCM8	4695	4714	1	1	0.00	0.00	19	10	3UTR
hsa-miR-11400	NM_001281520	MCM8	4293	4310	1	1	0.00	0.00	17	8	3UTR
hsa-miR-11400	NM_001281521	MCM8	4761	4780	1	1	0.00	0.00	19	10	3UTR
hsa-miR-11400	NM_001281521	MCM8	4359	4376	1	1	0.00	0.00	17	8	3UTR
hsa-miR-11400	NM_001281522	MCM8	4500	4519	1	1	0.00	0.00	19	10	3UTR
hsa-miR-11400	NM_001281522	MCM8	4098	4115	1	1	0.00	0.00	17	8	3UTR
hsa-miR-11400	NM_032485	MCM8	4641	4660	1	1	0.16	0.08	19	10	3UTR
hsa-miR-11400	NM_182802	MCM8	4647	4666	1	1	0.16	0.08	19	10	3UTR

mirnaid	refseqid	genesymbol	start	end	bindingp	seed	phylopstem	phylopflank	binding_region_length	longest_consecutive_pairings	position
hsa-miR-11400	NM_173518	MCMDC2	4803	4844	1	1	-0.04	-0.17	34	10	3UTR
hsa-miR-11400	NM_001288798	MCRIP1	1099	1117	1	1	0.00	0.00	18	10	3UTR
hsa-miR-11400	NM_001288799	MCRIP1	1162	1180	1	1	0.00	0.00	18	10	3UTR
hsa-miR-11400	NM_207368	MCRIP1	1264	1282	1	1	0.00	0.00	18	10	3UTR
hsa-miR-11400	NM_001093767	MCRIP1	963	981	1	1	0.00	0.00	18	10	3UTR
hsa-miR-11400	NM_001385001	MCTP2	7264	7295	1	1	0.09	0.07	31	9	3UTR
hsa-miR-11400	NM_001385004	MCTP2	7099	7130	1	1	0.09	0.07	31	9	3UTR
hsa-miR-11400	NM_001282940	MDH1B	1761	1782	1	1	1.12	1.33	21	12	3UTR
hsa-miR-11400	NM_001330223	MDH1B	1651	1672	1	1	1.73	1.22	21	12	3UTR
hsa-miR-11400	NM_001039845	MDH1B	1764	1785	1	1	1.12	1.33	21	12	3UTR
hsa-miR-11400	NM_020128	MDM1	913	964	1	1	0.52	0.16	19	9	3UTR
hsa-miR-11400	NM_001278462	MDM2	3498	3518	0.974359	1	1.19	0.47	20	14	3UTR
hsa-miR-11400	NM_002392	MDM2	4265	4285	0.974359	1	1.19	0.47	20	14	3UTR
hsa-miR-11400	NM_001367990	MDM2	4015	4035	0.974359	1	1.19	0.47	20	14	3UTR
hsa-miR-11400	NM_001145339	MDM2	4100	4120	0.974359	1	1.19	0.47	20	14	3UTR
hsa-miR-11400	NM_001145340	MDM2	3420	3440	0.974359	1	1.19	0.47	20	14	3UTR
hsa-miR-11400	NM_001363578	MEA1	968	988	1	1	2.44	1.29	20	10	3UTR

mirnaid	refseqid	genesymbol	start	end	bindingp	seed	phylopstem	phylopflank	binding_region_length	longest_consecutive_pairings	position
hsa-miR-11400	NM_133640	MED22	2021	2045	0.961538	1	0.00	0.00	24	13	3UTR
hsa-miR-11400	NM_133640	MED22	2507	2525	1	1	0.00	0.00	18	8	3UTR
hsa-miR-11400	NM_001319206	MEF2A	2382	2409	1	1	4.20	2.63	27	10	3UTR
hsa-miR-11400	NM_001365208	MEF2A	2358	2385	1	1	4.20	2.63	27	10	3UTR
hsa-miR-11400	NM_001130928	MEF2A	1951	1978	1	1	4.20	2.63	27	10	3UTR
hsa-miR-11400	XM_024446059	MEF2C	1966	1981	1	1	0.00	0.00	15	7	3UTR
hsa-miR-11400	NM_001364356	MEF2C	3220	3238	1	1	4.23	3.81	18	9	3UTR
hsa-miR-11400	NM_001131005	MEF2C	3800	3818	1	1	2.62	2.68	18	9	3UTR
hsa-miR-11400	NM_001193347	MEF2C	3860	3878	1	1	1.02	2.25	18	9	3UTR
hsa-miR-11400	NM_001198536	MEFV	1864	1893	0.961538	1	0.00	-0.16	23	8	3UTR
hsa-miR-11400	NM_001282136	MEI4	4827	4846	1	1	-0.25	-0.17	19	7	3UTR
hsa-miR-11400	NM_001322247	MEI4	4447	4466	1	1	-0.25	-0.17	19	7	3UTR
hsa-miR-11400	NM_002398	MEIS1	4330	4350	1	1	-0.16	0.50	20	10	3UTR
hsa-miR-11400	NM_002399	MEIS2	1676	1698	1	1	7.59	6.58	22	8	3UTR
hsa-miR-11400	NM_013999	MEOX1	1217	1241	0.991453	1	0.10	-0.01	24	7	3UTR
hsa-miR-11400	NM_181725	METTL2A	3162	3187	1	1	-0.20	-0.16	25	8	3UTR
hsa-miR-11400	NM_001330662	METTL6	1328	1377	0.974359	1	3.27	2.80	26	8	3UTR

mirnaid	refseqid	genesymbol	start	end	bindingp	seed	phylopstem	phylopflank	binding_region_length	longest_consecutive_pairings	position
hsa-miR-11400	NM_001301790	METTL6	1259	1282	1	1	0.40	-0.12	23	7	3UTR
hsa-miR-11400	NM_001301790	METTL6	1373	1403	1	1	-0.37	-0.02	30	9	3UTR
hsa-miR-11400	NM_152396	METTL6	1394	1417	1	1	0.09	-0.11	23	7	3UTR
hsa-miR-11400	NM_014033	METTL7A	2349	2369	1	1	-0.29	-0.08	20	7	3UTR
hsa-miR-11400	NM_001009554	MFAP3L	3528	3545	1	1	0.89	0.10	17	10	3UTR
hsa-miR-11400	NM_001009554	MFAP3L	4273	4294	1	1	1.22	0.93	21	11	3UTR
hsa-miR-11400	NM_001242532	MFSD11	1610	1631	1	1	0.00	0.00	21	10	3UTR
hsa-miR-11400	NM_033055	MFSD14A	1971	1994	1	1	1.43	2.30	23	8	3UTR
hsa-miR-11400	NM_001355230	MFSD14C	906	926	1	1	0.02	-0.07	20	7	3UTR
hsa-miR-11400	NM_032718	MFSD9	2392	2414	1	1	0.08	-0.19	22	7	3UTR
hsa-miR-11400	NM_002406	MGAT1	6778	6798	1	1	0.08	0.17	20	7	3UTR
hsa-miR-11400	NM_001114617	MGAT1	3496	3530	1	1	0.00	0.00	22	16	3UTR
hsa-miR-11400	NM_001114617	MGAT1	7178	7198	1	1	0.00	0.00	20	7	3UTR
hsa-miR-11400	NM_001351286	MGAT4C	8184	8207	1	1	0.16	0.05	23	13	3UTR
hsa-miR-11400	NM_001351288	MGAT4C	7797	7820	1	1	0.08	-0.13	23	13	3UTR
hsa-miR-11400	NM_001351288	MGAT4C	16218	16235	1	1	-0.26	-0.13	17	8	3UTR
hsa-miR-11400	NM_001351289	MGAT4C	7893	7916	1	1	0.23	0.04	23	13	3UTR

mirnaid	refseqid	genesymbol	start	end	bindingp	seed	phylopstem	phylopflank	binding_region_length	longest_consecutive_pairings	position
hsa-miR-11400	NM_001351289	MGAT4C	16314	16331	1	1	0.54	-0.03	17	8	3UTR
hsa-miR-11400	NM_001351291	MGAT4C	8917	8940	1	1	0.10	0.13	23	13	3UTR
hsa-miR-11400	NM_001351291	MGAT4C	17338	17355	1	1	0.00	-0.04	17	8	3UTR
hsa-miR-11400	NM_002410	MGAT5	8248	8267	1	1	1.54	2.19	19	14	3UTR
hsa-miR-11400	NM_001371457	MGAT5	8415	8434	1	1	1.54	2.19	19	14	3UTR
hsa-miR-11400	NM_001256585	MGLL	3262	3287	0.953846	1	3.48	3.29	20	8	3UTR
hsa-miR-11400	NM_007283	MGLL	3352	3377	0.953846	1	4.14	2.58	20	8	3UTR
hsa-miR-11400	NM_001003794	MGLL	3247	3272	0.953846	1	4.10	3.28	20	8	3UTR
hsa-miR-11400	XM_017005665	MGLL	3442	3467	1	1	0.00	0.00	20	8	3UTR
hsa-miR-11400	XM_024453334	MGLL	3254	3279	1	1	0.00	0.00	20	8	3UTR
hsa-miR-11400	NM_001363738	MGME1	1227	1245	1	1	-0.06	0.05	18	10	3UTR
hsa-miR-11400	XM_017008212	MGST2	1163	1181	1	1	0.00	0.00	18	9	3UTR
hsa-miR-11400	NM_001204367	MGST2	602	617	1	1	0.24	0.60	15	14	3UTR
hsa-miR-11400	NM_001282667	MICAL2	4510	4526	1	1	-0.08	0.17	16	15	3UTR
hsa-miR-11400	NM_033290	MID1	5527	5546	1	1	4.37	5.34	19	8	3UTR
hsa-miR-11400	NM_001347733	MID1	5771	5790	1	1	5.59	4.97	19	8	3UTR
hsa-miR-11400	NM_000381	MID1	5693	5712	1	1	5.09	5.82	19	8	3UTR

mirnaid	refseqid	genesymbol	start	end	bindingp	seed	phylopstem	phylopflank	binding_region_length	longest_consecutive_pairings	position
hsa-miR-11400	XM_005268205	MIDEAS	6542	6559	1	1	0.00	0.00	17	13	3UTR
hsa-miR-11400	XM_011530237	MIEF1	2907	2954	1	1	0.00	0.00	24	9	3UTR
hsa-miR-11400	NM_019008	MIEF1	2862	2909	1	1	0.00	0.00	24	9	3UTR
hsa-miR-11400	NM_001144900	MIEF2	2117	2179	1	1	0.00	0.00	31	11	3UTR
hsa-miR-11400	NM_032339	MIEN1	1228	1251	1	1	2.75	2.22	23	8	3UTR
hsa-miR-11400	NM_001330206	MIEN1	1307	1330	1	1	2.42	2.51	23	8	3UTR
hsa-miR-11400	NM_001278215	MIER1	739	749	1	1	0.11	0.12	10	9	3UTR
hsa-miR-11400	NM_017550	MIER2	2001	2020	1	1	1.39	2.26	19	11	3UTR
hsa-miR-11400	NM_001297598	MIER3	2553	2600	1	1	1.50	1.55	21	14	3UTR
hsa-miR-11400	NM_001297599	MIER3	2538	2585	1	1	3.96	1.44	21	14	3UTR
hsa-miR-11400	NM_152622	MIER3	2535	2582	1	1	3.43	1.61	21	14	3UTR
hsa-miR-11400	NM_001351281	MINDY4B	1570	1586	1	1	2.96	1.83	16	6	3UTR
hsa-miR-11400	NM_012064	MIP	2194	2228	1	1	4.56	3.02	17	15	3UTR
hsa-miR-11400	XM_024446767	MKLN1	3640	3659	1	1	0.00	0.00	19	15	3UTR
hsa-miR-11400	NM_013255	MKLN1	3416	3435	1	1	-0.44	0.20	19	15	3UTR
hsa-miR-11400	NM_005937	MLLT6	4164	4184	1	1	2.02	0.51	20	13	3UTR
hsa-miR-11400	NM_173470	MMGT1	3371	3387	1	1	-0.25	0.47	16	15	3UTR

mirnaid	refseqid	genesymbol	start	end	bindingp	seed	phylopstem	phylopflank	binding_region_length	longest_consecutive_pairings	position
hsa-miR-11400	NM_018221	MOB1A	1034	1053	1	1	0.28	0.37	19	11	3UTR
hsa-miR-11400	NM_001244766	MOB1B	2869	2888	1	1	0.00	0.00	19	7	3UTR
hsa-miR-11400	NM_145279	MOB3C	1616	1661	1	1	-0.05	0.29	25	9	3UTR
hsa-miR-11400	NM_201403	MOB3C	1655	1679	1	1	-0.01	0.21	24	9	3UTR
hsa-miR-11400	NM_025098	MOGAT2	1528	1550	1	1	0.13	-0.03	22	10	3UTR
hsa-miR-11400	XM_017019041	MON2	10405	10424	1	1	0.00	0.00	19	9	3UTR
hsa-miR-11400	NM_002436	MPP1	1785	1801	1	1	2.93	2.30	16	10	3UTR
hsa-miR-11400	NM_001166461	MPP1	1725	1741	1	1	2.93	2.30	16	10	3UTR
hsa-miR-11400	NM_001166462	MPP1	1934	1950	1	1	2.89	2.25	16	10	3UTR
hsa-miR-11400	NM_033066	MPP4	2112	2133	1	1	2.80	2.29	21	8	3UTR
hsa-miR-11400	XM_017004620	MPP4	3638	3659	1	1	0.00	0.00	21	8	3UTR
hsa-miR-11400	NM_001256550	MPP5	4255	4276	1	1	-0.18	-0.13	21	7	3UTR
hsa-miR-11400	NM_022474	MPP5	4487	4508	1	1	-0.18	-0.13	21	7	3UTR
hsa-miR-11400	NM_173496	MPP7	4093	4112	1	1	4.04	3.68	19	11	3UTR
hsa-miR-11400	NM_001145399	MPPED2	1870	1893	1	1	0.48	0.04	23	7	3UTR
hsa-miR-11400	NM_001145399	MPPED2	2087	2125	1	1	0.18	-0.01	24	8	3UTR
hsa-miR-11400	NM_005797	MPZL2	950	988	1	1	0.19	0.08	19	10	3UTR

mirnaid	refseqid	genesymbol	start	end	bindingp	seed	phylopstem	phylopflank	binding_region_length	longest_consecutive_pairings	position
hsa-miR-11400	NM_001385161	MR1	6869	6886	1	1	-0.05	0.08	17	10	3UTR
hsa-miR-11400	NM_001385164	MR1	6593	6610	1	1	-0.05	0.08	17	10	3UTR
hsa-miR-11400	NM_001531	MR1	7043	7060	1	1	-0.05	0.08	17	10	3UTR
hsa-miR-11400	NM_001194999	MR1	6908	6925	1	1	-0.05	0.08	17	10	3UTR
hsa-miR-11400	NM_001195000	MR1	6767	6784	1	1	-0.05	0.08	17	10	3UTR
hsa-miR-11400	NM_001195035	MR1	6662	6679	1	1	-0.05	0.08	17	10	3UTR
hsa-miR-11400	NM_138409	MRAP2	838	861	1	1	-0.19	-0.07	23	12	3UTR
hsa-miR-11400	NM_012219	MRAS	3537	3566	0.961538	1	-0.15	-0.01	29	11	3UTR
hsa-miR-11400	NM_001085049	MRAS	3048	3077	0.961538	1	-0.15	-0.01	29	11	3UTR
hsa-miR-11400	NM_001252091	MRAS	3326	3355	0.961538	1	-0.15	-0.01	29	11	3UTR
hsa-miR-11400	NM_001252090	MRAS	3019	3048	1	1	0.00	0.00	29	11	3UTR
hsa-miR-11400	NM_001252092	MRAS	2831	2860	1	1	0.00	0.00	29	11	3UTR
hsa-miR-11400	NM_001252093	MRAS	2837	2866	1	1	0.00	0.00	29	11	3UTR
hsa-miR-11400	NM_001039165	MRGPRE	3406	3424	1	1	0.40	-0.14	18	12	3UTR
hsa-miR-11400	NM_145015	MRGPRF	2045	2066	1	1	0.58	-0.09	21	10	3UTR
hsa-miR-11400	NM_001098515	MRGPRF	2056	2077	1	1	0.40	-0.07	21	10	3UTR
hsa-miR-11400	NM_014175	MRPL15	1331	1369	1	1	-0.26	-0.21	21	8	3UTR

mirnaid	refseqid	genesymbol	start	end	bindingp	seed	phylopstem	phylopflank	binding_region_length	longest_consecutive_pairings	position
hsa-miR-11400	NM_145212	MRPL30	1618	1633	1	1	0.84	-0.15	15	8	3UTR
hsa-miR-11400	NM_172177	MRPL42	8460	8481	1	1	-0.07	0.05	21	14	3UTR
hsa-miR-11400	NM_172177	MRPL42	8293	8314	1	1	-0.14	-0.05	21	9	3UTR
hsa-miR-11400	NM_172177	MRPL42	14669	14685	1	1	0.00	0.22	16	15	3UTR
hsa-miR-11400	NM_014050	MRPL42	8457	8478	1	1	-0.07	0.05	21	14	3UTR
hsa-miR-11400	NM_014050	MRPL42	8290	8311	1	1	-0.14	-0.05	21	9	3UTR
hsa-miR-11400	NM_014050	MRPL42	14666	14682	1	1	0.00	0.22	16	15	3UTR
hsa-miR-11400	NM_001193343	MRPS18A	437	467	1	1	0.00	-0.33	18	12	3UTR
hsa-miR-11400	NM_053035	MRPS33	1094	1113	1	1	-0.18	-0.06	19	9	3UTR
hsa-miR-11400	NM_016071	MRPS33	1181	1200	1	1	-0.36	0.08	19	9	3UTR
hsa-miR-11400	NM_138777	MRRF	7183	7213	1	1	-0.16	-0.26	19	11	3UTR
hsa-miR-11400	NM_199177	MRRF	7023	7053	1	1	-0.16	-0.26	19	11	3UTR
hsa-miR-11400	NM_001346343	MRRF	7094	7124	1	1	-0.16	-0.26	19	11	3UTR
hsa-miR-11400	NM_001173512	MRRF	7027	7057	1	1	0.00	0.00	19	11	3UTR
hsa-miR-11400	NM_001278242	MS4A15	1235	1259	1	1	-0.28	-0.04	24	11	3UTR
hsa-miR-11400	NM_152717	MS4A15	1071	1095	1	1	-0.28	-0.04	24	11	3UTR
hsa-miR-11400	NM_001098835	MS4A15	1358	1382	1	1	-0.28	-0.04	24	11	3UTR

mirnaid	refseqid	genesymbol	start	end	bindingp	seed	phylopstem	phylopflank	binding_region_length	longest_consecutive_pairings	position
hsa-miR-11400	NM_001354471	MS4A18	2038	2062	1	1	-0.04	0.23	24	10	3UTR
hsa-miR-11400	XM_005257014	MSI2	4805	4823	1	1	0.00	0.00	18	9	3UTR
hsa-miR-11400	NM_138715	MSR1	2710	2727	1	1	2.19	1.38	17	8	3UTR
hsa-miR-11400	NM_138716	MSR1	2514	2538	1	1	1.70	1.55	18	8	3UTR
hsa-miR-11400	NM_002445	MSR1	2008	2031	1	1	2.61	1.79	23	9	3UTR
hsa-miR-11400	NM_001363744	MSR1	2770	2787	1	1	2.89	2.31	17	8	3UTR
hsa-miR-11400	NM_005259	MSTN	1750	1767	0.961538	1	4.11	2.82	17	9	3UTR
hsa-miR-11400	NM_005259	MSTN	1352	1374	1	1	4.69	4.07	22	6	3UTR
hsa-miR-11400	NM_001282755	MTA3	3975	3993	1	1	-0.16	-0.20	18	9	3UTR
hsa-miR-11400	XM_017004562	MTA3	2471	2492	1	1	0.00	0.00	21	15	3UTR
hsa-miR-11400	NM_178812	MTDH	4084	4104	0.980769	1	0.25	0.17	20	14	3UTR
hsa-miR-11400	NM_001363138	MTDH	3985	4005	0.980769	1	0.25	0.17	20	14	3UTR
hsa-miR-11400	NM_005955	MTF1	2417	2438	1	1	0.30	0.47	21	8	3UTR
hsa-miR-11400	XM_011523285	MTHFSD	1452	1470	1	1	0.00	0.00	18	9	3UTR
hsa-miR-11400	NM_022764	MTHFSD	1411	1429	1	1	0.03	-0.70	18	9	3UTR
hsa-miR-11400	NM_001159377	MTHFSD	1414	1432	1	1	0.03	-0.71	18	9	3UTR
hsa-miR-11400	NM_001159378	MTHFSD	1414	1432	1	1	0.03	-0.71	18	9	3UTR

mirnaid	refseqid	genesymbol	start	end	bindingp	seed	phylopstem	phylopflank	binding_region_length	longest_consecutive_pairings	position
hsa-miR-11400	NM_001159379	MTHFSD	1411	1429	1	1	0.03	-0.70	18	9	3UTR
hsa-miR-11400	XM_011532871	MTIF2	3216	3241	1	1	0.00	0.00	25	10	3UTR
hsa-miR-11400	NM_017762	MTMR10	4616	4642	1	1	0.00	0.00	26	7	3UTR
hsa-miR-11400	NM_001294343	MTMR12	2217	2236	1	1	-0.14	-0.14	19	8	3UTR
hsa-miR-11400	NM_015458	MTMR9	2570	2606	1	1	0.50	0.92	19	13	3UTR
hsa-miR-11400	NM_133645	MTO1	3216	3238	1	1	-0.11	-0.06	22	8	3UTR
hsa-miR-11400	NM_012123	MTO1	3141	3163	1	1	-0.11	-0.06	22	8	3UTR
hsa-miR-11400	NM_001123226	MTO1	3261	3283	1	1	-0.11	-0.06	22	8	3UTR
hsa-miR-11400	XM_011544194	MTR	8453	8474	1	1	0.00	0.00	21	8	3UTR
hsa-miR-11400	NM_015360	MTREX	3702	3718	1	1	2.39	1.51	16	15	3UTR
hsa-miR-11400	NM_001190708	MTRNR2L10	945	964	1	1	-1.88	1.23	19	9	3UTR
hsa-miR-11400	NM_001190472	MTRNR2L3	951	970	1	1	0.22	0.84	19	9	3UTR
hsa-miR-11400	NM_001190476	MTRNR2L4	1585	1604	1	1	-0.10	0.02	19	9	3UTR
hsa-miR-11400	NM_001166393	MTUS1	1948	1979	1	1	0.55	0.33	31	8	3UTR
hsa-miR-11400	NM_001135091	MUC15	1809	1838	1	1	0.00	0.00	15	13	3UTR
hsa-miR-11400	NM_001135092	MUC15	1659	1688	1	1	0.00	0.00	15	13	3UTR
hsa-miR-11400	NM_138297	MUC4	3657	3678	1	1	1.18	0.44	21	15	3UTR

mirnaid	refseqid	genesymbol	start	end	bindingp	seed	phylopstem	phylopflank	binding_region_length	longest_consecutive_pairings	position
hsa-miR-11400	NM_001202514	MXD1	3614	3638	1	1	0.00	0.00	24	11	3UTR
hsa-miR-11400	NM_001020819	MYADM	2645	2667	1	1	-0.02	-0.01	22	6	3UTR
hsa-miR-11400	NM_025107	MYCT1	1336	1357	0.953846	1	-0.22	-0.02	21	12	3UTR
hsa-miR-11400	NM_001371626	MYCT1	776	797	0.953846	1	-0.22	-0.02	21	12	3UTR
hsa-miR-11400	NM_014981	MYH15	6985	7004	1	1	-0.04	0.70	19	10	3UTR
hsa-miR-11400	XM_024450768	MYO1C	4247	4266	1	1	0.00	0.00	19	10	3UTR
hsa-miR-11400	XM_024450769	MYO1C	4041	4060	1	1	0.00	0.00	19	10	3UTR
hsa-miR-11400	NM_004998	MYO1E	4918	4939	1	1	-0.27	2.49	21	10	3UTR
hsa-miR-11400	NM_006901	MYO9A	9486	9505	1	1	0.81	2.88	19	8	3UTR
hsa-miR-11400	NM_153604	MYOCD	3519	3536	1	1	-0.01	0.04	17	8	3UTR
hsa-miR-11400	NM_001146312	MYOCD	3663	3680	1	1	-0.01	0.04	17	8	3UTR
hsa-miR-11400	NM_001122853	MYOZ3	1348	1367	1	1	-0.07	-0.15	19	10	3UTR
hsa-miR-11400	NM_001012643	MYPOP	1726	1746	1	1	2.65	2.13	20	14	3UTR
hsa-miR-11400	NM_052818	N4BP2L1	1677	1699	1	1	0.00	0.33	22	14	3UTR
hsa-miR-11400	NM_001353632	N4BP2L1	1783	1805	1	1	-0.01	0.30	22	14	3UTR
hsa-miR-11400	NM_001079691	N4BP2L1	1669	1691	1	1	0.26	0.26	22	14	3UTR
hsa-miR-11400	NM_014887	N4BP2L2	7339	7358	1	1	2.60	1.46	19	13	3UTR

mirnaid	refseqid	genesymbol	start	end	bindingp	seed	phylopstem	phylopflank	binding_region_length	longest_consecutive_pairings	position
hsa-miR-11400	NM_057175	NAA15	4142	4184	1	1	2.55	2.78	20	15	3UTR
hsa-miR-11400	NM_057175	NAA15	4840	4854	1	1	2.96	1.32	14	13	3UTR
hsa-miR-11400	XM_005263236	NAA15	4145	4187	1	1	0.00	0.00	20	15	3UTR
hsa-miR-11400	XM_005263236	NAA15	4843	4857	1	1	0.00	0.00	14	13	3UTR
hsa-miR-11400	NM_207015	NAALADL2	5076	5094	1	1	-0.63	-0.03	18	7	3UTR
hsa-miR-11400	NM_001031716	NABP1	2055	2073	1	1	1.13	0.46	18	8	3UTR
hsa-miR-11400	NM_004537	NAP1L1	11569	11585	1	1	3.12	2.47	16	6	3UTR
hsa-miR-11400	NM_004537	NAP1L1	8221	8244	1	1	0.68	0.37	23	8	3UTR
hsa-miR-11400	NM_020443	NAV1	11654	11674	1	1	0.17	0.06	20	8	3UTR
hsa-miR-11400	NM_001167738	NAV1	10249	10269	1	1	0.17	0.06	20	8	3UTR
hsa-miR-11400	NM_145117	NAV2	8558	8591	1	1	-0.23	0.62	20	9	3UTR
hsa-miR-11400	NM_182964	NAV2	8567	8600	1	1	-0.23	0.62	20	9	3UTR
hsa-miR-11400	XM_011520444	NAV2	8723	8756	1	1	0.00	0.00	20	9	3UTR
hsa-miR-11400	NM_001111018	NAV2	8163	8196	1	1	-0.23	0.62	20	9	3UTR
hsa-miR-11400	NM_001111019	NAV2	5148	5181	1	1	-0.23	0.62	20	9	3UTR
hsa-miR-11400	NM_001244963	NAV2	8735	8768	1	1	0.00	0.00	20	9	3UTR
hsa-miR-11400	NM_001291571	NBR1	3014	3034	1	1	-0.49	-0.16	20	7	3UTR

mirnaid	refseqid	genesymbol	start	end	bindingp	seed	phylopstem	phylopflank	binding_region_length	longest_consecutive_pairings	position
hsa-miR-11400	NM_002486	NCBP1	4220	4241	1	1	-0.48	-0.01	21	12	3UTR
hsa-miR-11400	XM_011512556	NCBP2	3370	3386	1	1	0.00	0.00	16	15	3UTR
hsa-miR-11400	XM_011512557	NCBP2	3484	3500	1	1	0.00	0.00	16	15	3UTR
hsa-miR-11400	XM_011512558	NCBP2	3409	3425	1	1	0.00	0.00	16	15	3UTR
hsa-miR-11400	NM_001355243	NCBP2AS2	592	619	1	1	0.32	-0.21	22	8	3UTR
hsa-miR-11400	NM_013416	NCF4	1245	1264	1	1	3.86	2.53	19	8	3UTR
hsa-miR-11400	NM_205842	NCKAP1	13200	13224	1	1	0.27	0.18	24	9	3UTR
hsa-miR-11400	NM_013436	NCKAP1	13182	13206	1	1	0.27	0.12	24	9	3UTR
hsa-miR-11400	NM_005337	NCKAP1L	5206	5240	1	1	-0.01	0.22	34	9	3UTR
hsa-miR-11400	NM_005337	NCKAP1L	7857	7877	1	1	-0.02	0.26	20	7	3UTR
hsa-miR-11400	NM_001184976	NCKAP1L	5124	5158	1	1	-0.01	0.22	34	9	3UTR
hsa-miR-11400	NM_001184976	NCKAP1L	7775	7795	1	1	-0.02	0.26	20	7	3UTR
hsa-miR-11400	XM_005264625	NCOA1	4896	4912	1	1	0.00	0.00	16	15	3UTR
hsa-miR-11400	NM_181659	NCOA3	7513	7537	1	1	-0.57	-0.07	24	8	3UTR
hsa-miR-11400	NM_006534	NCOA3	7501	7525	1	1	-0.57	-0.07	24	8	3UTR
hsa-miR-11400	NM_001174088	NCOA3	7486	7510	1	1	-0.57	-0.07	24	8	3UTR
hsa-miR-11400	NM_001202439	NCR3LG1	4874	4894	1	1	0.00	0.00	20	10	3UTR

mirnaid	refseqid	genesymbol	start	end	bindingp	seed	phylopstem	phylopflank	binding_region_length	longest_consecutive_pairings	position
hsa-miR-11400	NM_001128826	NCS1	2178	2196	1	1	-0.55	-0.23	18	11	3UTR
hsa-miR-11400	NM_032013	NDRG3	2828	2847	1	1	3.79	2.67	19	9	3UTR
hsa-miR-11400	XM_006723837	NDRG3	2755	2770	1	1	0.00	0.00	15	7	3UTR
hsa-miR-11400	XM_006723837	NDRG3	2858	2877	1	1	0.00	0.00	19	9	3UTR
hsa-miR-11400	XM_011528928	NDRG3	2574	2589	1	1	0.00	0.00	15	7	3UTR
hsa-miR-11400	XM_011528928	NDRG3	2677	2696	1	1	0.00	0.00	19	9	3UTR
hsa-miR-11400	NM_001242833	NDRG4	1574	1609	1	1	3.12	3.20	22	11	3UTR
hsa-miR-11400	NM_004784	NDST3	3774	3791	1	1	0.44	0.07	17	9	3UTR
hsa-miR-11400	NM_002490	NDUFA6	956	974	1	1	0.00	0.00	18	13	3UTR
hsa-miR-11400	NM_005002	NDUFA9	4675	4700	1	1	-0.23	-0.02	25	8	3UTR
hsa-miR-11400	NM_005002	NDUFA9	2909	2928	1	1	0.71	-0.01	19	15	3UTR
hsa-miR-11400	NM_001199983	NDUFS1	4846	4870	1	1	0.15	0.06	24	11	3UTR
hsa-miR-11400	NM_001199984	NDUFS1	4948	4972	1	1	0.42	0.43	24	11	3UTR
hsa-miR-11400	NM_213569	NEBL	3876	3898	1	1	0.53	0.11	22	9	3UTR
hsa-miR-11400	NM_001377327	NEBL	3840	3862	1	1	-0.48	0.37	22	9	3UTR
hsa-miR-11400	NM_001377328	NEBL	3545	3567	1	1	0.23	0.41	22	9	3UTR
hsa-miR-11400	NM_022351	NECAB1	3980	3998	1	1	0.17	0.36	18	8	3UTR

mirnaid	refseqid	genesymbol	start	end	bindingp	seed	phylopstem	phylopflank	binding_region_length	longest_consecutive_pairings	position
hsa-miR-11400	NM_001135747	NEIL2	1456	1475	1	1	-0.26	-0.20	19	10	3UTR
hsa-miR-11400	NM_003157	NEK4	3040	3063	1	1	0.60	0.60	23	8	3UTR
hsa-miR-11400	NM_001193533	NEK4	2773	2796	1	1	0.50	0.36	23	8	3UTR
hsa-miR-11400	NM_001365552	NEK5	3339	3374	0.961538	1	0.52	-0.01	21	12	3UTR
hsa-miR-11400	NM_001365552	NEK5	2953	2972	1	1	-0.40	-0.35	19	10	3UTR
hsa-miR-11400	NM_015257	NEMP1	4835	4852	1	1	2.58	3.46	17	8	3UTR
hsa-miR-11400	NM_001130963	NEMP1	5054	5071	1	1	1.99	3.55	17	8	3UTR
hsa-miR-11400	NM_002499	NEO1	6493	6513	1	1	-0.41	0.01	20	15	3UTR
hsa-miR-11400	NM_001172623	NEO1	6313	6333	1	1	-0.41	0.01	20	15	3UTR
hsa-miR-11400	NM_001172624	NEO1	6439	6459	1	1	-0.41	0.01	20	15	3UTR
hsa-miR-11400	XM_017023740	NETO2	1744	1755	1	1	0.00	0.00	11	10	3UTR
hsa-miR-11400	NM_000434	NEU1	1306	1324	1	1	-0.13	-0.34	18	11	3UTR
hsa-miR-11400	NM_001308177	NEURL1B	3582	3603	1	1	-0.15	0.20	21	11	3UTR
hsa-miR-11400	NM_001308178	NEURL1B	3408	3429	1	1	-0.15	0.20	21	11	3UTR
hsa-miR-11400	NM_001142651	NEURL1B	4128	4149	1	1	-0.15	0.20	21	11	3UTR
hsa-miR-11400	NM_001285485	NEURL3	908	939	1	1	3.05	2.71	25	8	3UTR
hsa-miR-11400	NM_001285486	NEURL3	1172	1203	1	1	4.63	2.74	25	8	3UTR

mirnaid	refseqid	genesymbol	start	end	bindingp	seed	phylopstem	phylopflank	binding_region_length	longest_consecutive_pairings	position
hsa-miR-11400	NM_144573	NEXN	3059	3091	1	1	0.04	0.15	32	10	3UTR
hsa-miR-11400	NM_000267	NF1	11672	11690	1	1	-0.19	-0.15	18	14	3UTR
hsa-miR-11400	NM_001042492	NF1	11685	11703	1	1	-0.19	-0.15	18	14	3UTR
hsa-miR-11400	NM_173163	NFATC3	6011	6032	1	1	0.50	0.46	21	10	3UTR
hsa-miR-11400	NM_173165	NFATC3	5922	5943	1	1	0.50	0.46	21	10	3UTR
hsa-miR-11400	NM_004555	NFATC3	6026	6047	1	1	0.50	0.46	21	10	3UTR
hsa-miR-11400	NM_001369476	NFIB	1670	1687	0.969231	1	1.89	2.92	17	11	3UTR
hsa-miR-11400	NM_001282787	NFIB	5693	5715	0.974359	1	3.44	2.10	22	9	3UTR
hsa-miR-11400	NM_001369460	NFIB	2546	2586	1	1	4.08	3.80	40	9	3UTR
hsa-miR-11400	NM_001369464	NFIB	2510	2550	1	1	3.44	4.54	40	9	3UTR
hsa-miR-11400	NM_001369466	NFIB	2297	2337	1	1	4.13	4.86	40	9	3UTR
hsa-miR-11400	NM_001369470	NFIB	2232	2272	1	1	3.95	4.89	40	9	3UTR
hsa-miR-11400	NM_001190738	NFIB	2221	2261	1	1	3.62	4.67	40	9	3UTR
hsa-miR-11400	NM_001378599	NFILZ	4664	4682	0.974359	1	0.46	0.11	18	7	3UTR
hsa-miR-11400	NM_001378600	NFILZ	4500	4518	0.974359	1	0.46	0.11	18	7	3UTR
hsa-miR-11400	NM_001378601	NFILZ	4095	4113	0.974359	1	0.46	0.11	18	7	3UTR
hsa-miR-11400	NM_001271043	NFIX	4071	4094	1	1	1.37	1.83	23	11	3UTR

mirnaid	refseqid	genesymbol	start	end	bindingp	seed	phylopstem	phylopflank	binding_region_length	longest_consecutive_pairings	position
hsa-miR-11400	NM_001271044	NFIX	3882	3905	1	1	1.37	1.83	23	11	3UTR
hsa-miR-11400	XM_006722760	NFIX	3915	3938	1	1	0.00	0.00	23	11	3UTR
hsa-miR-11400	NM_002501	NFIX	4209	4232	1	1	1.37	1.83	23	11	3UTR
hsa-miR-11400	NM_001365902	NFIX	4357	4380	1	1	1.37	1.83	23	11	3UTR
hsa-miR-11400	NM_001365984	NFIX	4209	4232	1	1	1.37	1.83	23	11	3UTR
hsa-miR-11400	NM_001365985	NFIX	4061	4084	1	1	1.37	1.83	23	11	3UTR
hsa-miR-11400	NM_001378404	NFIX	4030	4053	1	1	1.37	1.83	23	11	3UTR
hsa-miR-11400	NM_006165	NFRKB	5108	5129	1	1	0.00	0.00	21	10	3UTR
hsa-miR-11400	NM_006165	NFRKB	4900	4930	1	1	0.73	2.12	30	9	3UTR
hsa-miR-11400	NM_001143835	NFRKB	5096	5117	1	1	-0.15	0.20	21	10	3UTR
hsa-miR-11400	NM_021705	NFYA	2789	2809	1	1	0.47	0.27	20	11	3UTR
hsa-miR-11400	NM_002505	NFYA	2876	2896	1	1	0.47	0.27	20	11	3UTR
hsa-miR-11400	NM_001114090	NGEF	2449	2496	1	1	1.88	2.16	47	8	3UTR
hsa-miR-11400	NM_024782	NHEJ1	5611	5650	1	1	0.39	-0.12	17	8	3UTR
hsa-miR-11400	NM_001377498	NHEJ1	5561	5600	1	1	0.59	-0.13	17	8	3UTR
hsa-miR-11400	NM_001144060	NHSL1	5843	5894	1	1	1.99	1.25	51	10	3UTR
hsa-miR-11400	XM_017029476	NHSL2	9369	9390	1	1	0.00	0.00	21	9	3UTR

mirnaid	refseqid	genesymbol	start	end	bindingp	seed	phylopstem	phylopflank	binding_region_length	longest_consecutive_pairings	position
hsa-miR-11400	NM_001322865	NIPAL3	1204	1225	1	1	-0.42	-0.29	21	9	3UTR
hsa-miR-11400	NM_001099287	NIPAL4	2103	2118	1	1	0.29	-0.05	15	7	3UTR
hsa-miR-11400	NM_001172292	NIPAL4	2240	2255	1	1	0.29	-0.05	15	7	3UTR
hsa-miR-11400	NM_020202	NIT2	6317	6336	1	1	-0.26	0.25	19	11	3UTR
hsa-miR-11400	NM_152864	NKAIN4	928	946	1	1	1.63	1.61	18	17	3UTR
hsa-miR-11400	NM_198478	NKPD1	2916	2938	1	1	0.69	0.40	22	13	3UTR
hsa-miR-11400	NM_181303	NLGN3	3104	3154	1	1	0.77	1.22	50	8	3UTR
hsa-miR-11400	NM_181303	NLGN3	2876	2892	1	1	2.14	2.87	16	12	3UTR
hsa-miR-11400	NM_018977	NLGN3	3044	3094	1	1	0.77	1.22	50	8	3UTR
hsa-miR-11400	NM_018977	NLGN3	2816	2832	1	1	2.14	2.87	16	12	3UTR
hsa-miR-11400	NM_001321276	NLGN3	2531	2581	1	1	0.77	1.22	50	8	3UTR
hsa-miR-11400	NM_001321276	NLGN3	2303	2319	1	1	2.14	2.87	16	12	3UTR
hsa-miR-11400	NM_001166660	NLGN3	2984	3034	1	1	0.66	1.35	50	8	3UTR
hsa-miR-11400	NM_001319967	NLRP2B	550	574	1	1	0.00	0.00	24	10	3UTR
hsa-miR-11400	NM_001319967	NLRP2B	2608	2659	1	1	0.00	0.00	22	13	3UTR
hsa-miR-11400	NM_001282143	NLRX1	3252	3278	1	1	-0.19	-0.07	26	10	3UTR
hsa-miR-11400	NM_001282144	NLRX1	3660	3686	1	1	-0.19	-0.07	26	10	3UTR

mirnaid	refseqid	genesymbol	start	end	bindingp	seed	phylopstem	phylopflank	binding_region_length	longest_consecutive_pairings	position
hsa-miR-11400	NM_001282358	NLRX1	3196	3222	1	1	-0.19	-0.07	26	10	3UTR
hsa-miR-11400	NM_024618	NLRX1	3279	3305	1	1	-0.19	-0.07	26	10	3UTR
hsa-miR-11400	XM_017005517	NME6	4090	4110	1	1	0.00	0.00	20	9	3UTR
hsa-miR-11400	XM_017005517	NME6	804	823	1	1	0.00	0.00	19	10	3UTR
hsa-miR-11400	NM_005793	NME6	1043	1062	1	1	-0.02	0.35	19	10	3UTR
hsa-miR-11400	NM_001198546	NOL4	2074	2123	1	1	2.86	3.59	33	8	3UTR
hsa-miR-11400	NM_001198549	NOL4	1370	1419	1	1	2.96	1.53	33	8	3UTR
hsa-miR-11400	NM_001284388	NOLC1	3389	3407	1	1	0.01	-0.04	18	17	3UTR
hsa-miR-11400	NM_001284389	NOLC1	3362	3380	1	1	-0.38	-0.48	18	17	3UTR
hsa-miR-11400	NM_004741	NOLC1	3359	3377	1	1	-0.38	-0.48	18	17	3UTR
hsa-miR-11400	NM_004646	NPHS1	4540	4560	1	1	1.16	1.68	20	9	3UTR
hsa-miR-11400	XM_011521452	NPTN	1256	1275	1	1	0.00	0.00	19	8	3UTR
hsa-miR-11400	NM_001370180	NPY2R	2546	2586	1	1	0.36	0.25	18	16	3UTR
hsa-miR-11400	NM_000910	NPY2R	2550	2590	1	1	0.36	0.25	18	16	3UTR
hsa-miR-11400	XM_005277644	NPY4R2	1904	1928	1	1	0.00	0.00	24	7	3UTR
hsa-miR-11400	XM_006717545	NPY4R2	1748	1772	1	1	0.00	0.00	24	7	3UTR
hsa-miR-11400	XM_006713451	NR1D2	3918	3965	1	1	0.00	0.00	19	9	3UTR

mirnaid	refseqid	genesymbol	start	end	bindingp	seed	phylopstem	phylopflank	binding_region_length	longest_consecutive_pairings	position
hsa-miR-11400	NM_005126	NR1D2	4397	4415	1	1	1.67	2.09	18	9	3UTR
hsa-miR-11400	NM_001206977	NR1H4	2771	2792	1	1	0.00	0.00	21	9	3UTR
hsa-miR-11400	NM_001206978	NR1H4	2328	2346	1	1	0.86	0.69	18	14	3UTR
hsa-miR-11400	NM_001206979	NR1H4	2604	2625	1	1	0.00	0.00	21	9	3UTR
hsa-miR-11400	NM_001206979	NR1H4	2481	2499	1	1	1.33	0.83	18	14	3UTR
hsa-miR-11400	NM_001206992	NR1H4	2267	2288	1	1	0.00	0.00	21	9	3UTR
hsa-miR-11400	NM_033013	NR1I2	2090	2108	1	1	2.94	2.69	18	12	3UTR
hsa-miR-11400	NM_004959	NR5A1	2983	3002	1	1	0.48	0.64	19	9	3UTR
hsa-miR-11400	NM_017970	NRDE2	12734	12759	1	1	5.00	3.81	25	8	3UTR
hsa-miR-11400	NM_017970	NRDE2	7626	7643	1	1	0.48	-0.08	17	11	3UTR
hsa-miR-11400	XM_011544512	NRG1	8198	8217	1	1	0.00	0.00	19	6	3UTR
hsa-miR-11400	NM_138573	NRG4	1603	1625	1	1	0.07	-0.03	22	12	3UTR
hsa-miR-11400	XM_005267709	NRL	1906	1925	1	1	0.00	0.00	19	18	3UTR
hsa-miR-11400	NM_001354768	NRL	1827	1846	1	1	0.00	0.00	19	18	3UTR
hsa-miR-11400	NM_001354769	NRL	2016	2035	1	1	0.00	0.00	19	18	3UTR
hsa-miR-11400	NM_172349	NSD1	11834	11856	1	1	-0.17	0.46	22	10	3UTR
hsa-miR-11400	NM_001365684	NSD1	11711	11733	1	1	0.31	0.14	22	10	3UTR

mirnaid	refseqid	genesymbol	start	end	bindingp	seed	phylopstem	phylopflank	binding_region_length	longest_consecutive_pairings	position
hsa-miR-11400	NM_133334	NSD2	6689	6708	0.980769	1	-0.50	-0.48	19	10	3UTR
hsa-miR-11400	NM_133334	NSD2	3187	3220	1	1	-0.07	-0.18	17	15	3UTR
hsa-miR-11400	XM_006713914	NSD2	7044	7063	1	1	0.00	0.00	19	10	3UTR
hsa-miR-11400	XM_006713914	NSD2	3542	3575	1	1	0.00	0.00	17	15	3UTR
hsa-miR-11400	XM_017008588	NSD2	2958	2977	1	1	0.00	0.00	19	9	3UTR
hsa-miR-11400	NM_015537	NSMF	3480	3499	1	1	-0.36	0.16	19	8	3UTR
hsa-miR-11400	NM_001130969	NSMF	3486	3505	1	1	-0.36	0.16	19	8	3UTR
hsa-miR-11400	NM_001130970	NSMF	3417	3436	1	1	-0.36	0.16	19	8	3UTR
hsa-miR-11400	NM_001130971	NSMF	3411	3430	1	1	-0.36	0.16	19	8	3UTR
hsa-miR-11400	NM_001178064	NSMF	3396	3415	1	1	-0.36	0.16	19	8	3UTR
hsa-miR-11400	NM_018044	NSUN5	1458	1483	1	1	2.24	3.59	25	9	3UTR
hsa-miR-11400	NM_001168348	NSUN5	1344	1369	1	1	4.64	2.30	25	9	3UTR
hsa-miR-11400	NM_001351174	NT5C2	3312	3335	1	1	2.28	2.07	23	8	3UTR
hsa-miR-11400	NM_152729	NT5DC1	6090	6107	1	1	0.14	0.16	17	16	3UTR
hsa-miR-11400	NM_152729	NT5DC1	4630	4664	1	1	0.14	0.25	34	8	3UTR
hsa-miR-11400	NM_001048209	NTM	1300	1350	1	1	0.46	0.98	18	8	3UTR
hsa-miR-11400	NM_032536	NTNG2	4190	4208	1	1	0.41	-0.04	18	17	3UTR

mirnaid	refseqid	genesymbol	start	end	bindingp	seed	phylopstem	phylopflank	binding_region_length	longest_consecutive_pairings	position
hsa-miR-11400	NM_022731	NUCKS1	6070	6089	1	1	3.94	3.97	19	9	3UTR
hsa-miR-11400	NM_152395	NUDT16	3817	3837	1	1	-0.01	0.15	20	12	3UTR
hsa-miR-11400	NM_001171905	NUDT16	3652	3672	1	1	-0.01	0.15	20	12	3UTR
hsa-miR-11400	NM_001171905	NUDT16	1746	1764	1	1	-0.10	-0.40	18	7	3UTR
hsa-miR-11400	NM_198038	NUDT9	1077	1098	1	1	0.11	-0.39	21	8	3UTR
hsa-miR-11400	NM_001248011	NUDT9	1318	1339	1	1	0.00	0.00	21	8	3UTR
hsa-miR-11400	NM_017681	NUP62CL	1414	1431	1	1	1.21	0.66	17	9	3UTR
hsa-miR-11400	NM_206840	NVL	2627	2643	1	1	1.00	2.27	16	15	3UTR
hsa-miR-11400	NM_001243146	NVL	2377	2393	1	1	1.00	2.27	16	15	3UTR
hsa-miR-11400	NM_001081491	NXF1	1168	1200	1	1	3.30	3.55	18	16	3UTR
hsa-miR-11400	NM_145283	NXNL2	1164	1187	1	1	-0.19	0.01	23	10	3UTR
hsa-miR-11400	NM_152745	NXPH1	2316	2335	1	1	0.73	1.72	19	15	3UTR
hsa-miR-11400	NM_018698	NXT2	1167	1187	1	1	0.60	0.33	20	11	3UTR
hsa-miR-11400	XM_005246708	NYAP2	7148	7174	1	1	0.00	0.00	26	8	3UTR
hsa-miR-11400	NM_001032731	OAS2	1536	1573	1	1	0.09	0.02	23	8	3UTR
hsa-miR-11400	NM_001032731	OAS2	1625	1649	1	1	-0.02	0.07	19	7	3UTR
hsa-miR-11400	NM_001300984	OCA2	2675	2693	1	1	0.00	0.00	18	14	3UTR

mirnaid	refseqid	genesymbol	start	end	bindingp	seed	phylopstem	phylopflank	binding_region_length	longest_consecutive_pairings	position
hsa-miR-11400	NM_000275	OCA2	2747	2765	1	1	0.00	0.00	18	14	3UTR
hsa-miR-11400	NM_001164245	ODR4	2809	2827	0.953846	1	0.72	0.15	18	15	3UTR
hsa-miR-11400	NM_001164246	ODR4	2782	2800	0.953846	1	0.72	0.15	18	15	3UTR
hsa-miR-11400	NM_018233	OGFOD1	2538	2563	1	1	-0.02	-0.08	25	8	3UTR
hsa-miR-11400	NM_181672	OGT	3563	3587	1	1	1.55	1.68	24	9	3UTR
hsa-miR-11400	NM_181673	OGT	3533	3557	1	1	1.55	1.68	24	9	3UTR
hsa-miR-11400	NM_004852	ONECUT2	13110	13129	1	1	0.78	0.93	19	10	3UTR
hsa-miR-11400	XM_006723403	OPA3	3490	3521	0.980769	1	0.00	0.00	20	11	3UTR
hsa-miR-11400	NM_025136	OPA3	5197	5222	1	1	-0.19	0.05	25	8	3UTR
hsa-miR-11400	NM_033207	OPALIN	2859	2888	1	1	0.49	1.25	19	6	3UTR
hsa-miR-11400	NM_001040103	OPALIN	2898	2927	1	1	0.98	1.07	19	6	3UTR
hsa-miR-11400	NM_001319105	OPCML	3879	3904	1	1	2.70	1.52	25	8	3UTR
hsa-miR-11400	NM_002547	OPHN1	5083	5101	1	1	1.50	1.82	18	8	3UTR
hsa-miR-11400	NM_001282904	OPRK1	4461	4484	1	1	3.64	2.78	23	10	3UTR
hsa-miR-11400	NM_001200019	OPRL1	1684	1703	1	1	0.00	0.00	19	12	3UTR
hsa-miR-11400	NM_001285526	OPRM1	5904	5928	1	1	0.19	-0.11	24	12	3UTR
hsa-miR-11400	NM_000914	OPRM1	5220	5244	1	1	0.19	-0.11	24	12	3UTR

mirnaid	refseqid	genesymbol	start	end	bindingp	seed	phylopstem	phylopflank	binding_region_length	longest_consecutive_pairings	position
hsa-miR-11400	NM_001145279	OPRM1	5717	5741	1	1	0.19	-0.11	24	12	3UTR
hsa-miR-11400	NM_001145280	OPRM1	5148	5172	1	1	0.19	-0.11	24	12	3UTR
hsa-miR-11400	NM_001145281	OPRM1	4689	4713	1	1	0.19	-0.11	24	12	3UTR
hsa-miR-11400	NM_001145287	OPRM1	5923	5947	1	1	0.19	-0.11	24	12	3UTR
hsa-miR-11400	NM_001386033	OR11G2	3122	3152	1	1	0.00	0.00	24	12	3UTR
hsa-miR-11400	NM_001005503	OR11G2	2495	2525	1	1	0.00	0.00	24	12	3UTR
hsa-miR-11400	NM_014565	OR1A1	2034	2052	1	1	0.45	0.47	18	13	3UTR
hsa-miR-11400	NM_003553	OR1E1	1054	1096	1	1	0.06	0.72	21	10	3UTR
hsa-miR-11400	NM_001004456	OR1M1	2387	2405	1	1	0.00	0.00	18	9	3UTR
hsa-miR-11400	NM_001005287	OR2A1	3965	3988	1	1	-0.02	-0.10	23	15	3UTR
hsa-miR-11400	NM_001013355	OR2G6	4622	4641	1	1	0.00	0.00	19	10	3UTR
hsa-miR-11400	NM_001013355	OR2G6	5163	5185	1	1	0.00	0.00	22	11	3UTR
hsa-miR-11400	NM_017504	OR2M4	5572	5613	1	1	0.00	0.00	21	9	3UTR
hsa-miR-11400	NM_017504	OR2M4	2391	2410	1	1	0.00	0.00	19	8	3UTR
hsa-miR-11400	NM_001004692	OR2T12	3484	3509	1	1	0.00	0.00	25	6	3UTR
hsa-miR-11400	NM_001004136	OR2T2	2896	2919	1	1	0.00	0.00	23	10	3UTR
hsa-miR-11400	NM_001001827	OR2T35	2454	2493	1	1	0.00	0.00	24	10	3UTR

mirnaid	refseqid	genesymbol	start	end	bindingp	seed	phylopstem	phylopflank	binding_region_length	longest_consecutive_pairings	position
hsa-miR-11400	NM_002550	OR3A1	1579	1598	1	1	0.00	0.00	19	7	3UTR
hsa-miR-11400	NM_001386029	OR4K13	2949	2968	1	1	0.00	0.00	19	7	3UTR
hsa-miR-11400	NM_001004714	OR4K13	2653	2672	1	1	0.00	0.00	19	7	3UTR
hsa-miR-11400	NM_001004715	OR4K17	1423	1442	1	1	0.00	0.00	19	13	3UTR
hsa-miR-11400	NM_001004754	OR51I2	1604	1635	1	1	0.00	0.00	31	10	3UTR
hsa-miR-11400	NM_001004754	OR51I2	2410	2435	1	1	0.00	0.00	25	10	3UTR
hsa-miR-11400	NM_001004755	OR51L1	3527	3547	1	1	-0.11	0.04	20	11	3UTR
hsa-miR-11400	NM_001001917	OR56A1	2421	2442	1	1	0.00	0.00	21	8	3UTR
hsa-miR-11400	NM_001001917	OR56A1	4208	4235	1	1	0.00	0.00	15	8	3UTR
hsa-miR-11400	NM_001003443	OR56A3	2466	2505	1	1	0.00	0.00	24	9	3UTR
hsa-miR-11400	NM_001001954	OR5A2	3038	3059	1	1	0.00	0.00	14	12	3UTR
hsa-miR-11400	NM_001004729	OR5AN1	4791	4825	1	1	0.06	0.10	24	9	3UTR
hsa-miR-11400	NM_001005497	OR6C75	2813	2828	1	1	0.00	0.00	15	7	3UTR
hsa-miR-11400	NM_001348233	OR6J1	3308	3326	1	1	0.00	0.00	18	9	3UTR
hsa-miR-11400	NM_001005191	OR7D4	2630	2648	0.991453	1	0.00	0.00	18	6	3UTR
hsa-miR-11400	NM_001005467	OR8B3	1658	1710	1	1	-1.63	0.34	21	8	3UTR
hsa-miR-11400	NM_001005199	OR8H1	1944	1963	1	1	0.00	0.00	19	10	3UTR

mirnaid	refseqid	genesymbol	start	end	bindingp	seed	phylopstem	phylopflank	binding_region_length	longest_consecutive_pairings	position
hsa-miR-11400	NM_001271818	ORAI2	3293	3317	1	1	0.20	0.03	24	10	3UTR
hsa-miR-11400	NM_032831	ORAI2	3161	3185	1	1	-0.01	0.17	24	10	3UTR
hsa-miR-11400	NM_001126340	ORAI2	3270	3294	1	1	-0.01	0.17	24	10	3UTR
hsa-miR-11400	NM_001371387	ORMDL1	3441	3473	1	1	0.67	0.11	23	9	3UTR
hsa-miR-11400	XM_017017164	OSBPL5	3411	3453	1	1	0.00	0.00	42	11	3UTR
hsa-miR-11400	NM_032523	OSBPL6	5423	5455	0.974359	1	-0.35	-0.01	32	8	3UTR
hsa-miR-11400	NM_145739	OSBPL6	5044	5076	0.974359	1	-0.35	-0.01	32	8	3UTR
hsa-miR-11400	XM_017003271	OSBPL6	5323	5355	1	1	0.00	0.00	32	8	3UTR
hsa-miR-11400	NM_001201480	OSBPL6	5498	5530	1	1	0.00	0.00	32	8	3UTR
hsa-miR-11400	NM_001201481	OSBPL6	5330	5362	1	1	0.00	0.00	32	8	3UTR
hsa-miR-11400	NM_138348	OTULIN	7764	7781	0.980769	1	-0.45	-0.01	17	16	3UTR
hsa-miR-11400	NM_002565	P2RY4	1414	1437	1	1	0.00	0.00	23	9	3UTR
hsa-miR-11400	NM_001277204	P2RY6	1797	1841	1	1	-0.16	-0.17	23	10	3UTR
hsa-miR-11400	NM_001277205	P2RY6	1939	1983	1	1	-0.24	-0.15	23	10	3UTR
hsa-miR-11400	NM_001277206	P2RY6	1904	1948	1	1	0.13	-0.15	23	10	3UTR
hsa-miR-11400	NM_001277207	P2RY6	1886	1930	1	1	-0.04	0.13	23	10	3UTR
hsa-miR-11400	NM_176796	P2RY6	1972	2016	1	1	-0.24	-0.15	23	10	3UTR

mirnaid	refseqid	genesymbol	start	end	bindingp	seed	phylopstem	phylopflank	binding_region_length	longest_consecutive_pairings	position
hsa-miR-11400	NM_176797	P2RY6	1812	1856	1	1	-0.24	-0.15	23	10	3UTR
hsa-miR-11400	NM_176798	P2RY6	2090	2134	1	1	-0.16	-0.17	23	10	3UTR
hsa-miR-11400	XM_005274022	P2RY6	3706	3727	1	1	0.00	0.00	21	12	3UTR
hsa-miR-11400	NM_001142599	P4HA2	3636	3656	0.953846	1	1.93	1.93	20	7	3UTR
hsa-miR-11400	NM_001365678	P4HA2	4549	4584	1	1	0.54	0.07	28	9	3UTR
hsa-miR-11400	NM_001142599	P4HA2	4518	4553	1	1	0.01	0.19	28	9	3UTR
hsa-miR-11400	NM_001267803	PAAF1	2223	2246	1	1	0.23	0.29	23	8	3UTR
hsa-miR-11400	NM_001267805	PAAF1	2210	2233	1	1	0.23	0.29	23	8	3UTR
hsa-miR-11400	NM_001267806	PAAF1	2127	2150	1	1	0.00	0.00	23	8	3UTR
hsa-miR-11400	NM_025155	PAAF1	2154	2177	1	1	0.23	0.29	23	8	3UTR
hsa-miR-11400	NM_001363556	PAAF1	2404	2427	1	1	0.00	0.00	23	8	3UTR
hsa-miR-11400	NM_001258345	PACRGL	4182	4201	0.980769	1	-0.56	-0.07	19	12	3UTR
hsa-miR-11400	NM_001258346	PACRGL	3875	3894	0.980769	1	-0.56	-0.07	19	12	3UTR
hsa-miR-11400	NM_001317849	PACRGL	3902	3921	0.980769	1	-0.56	-0.07	19	12	3UTR
hsa-miR-11400	NM_145048	PACRGL	4101	4120	0.980769	1	-0.56	-0.07	19	12	3UTR
hsa-miR-11400	NM_001330748	PACRGL	3733	3752	0.980769	1	-0.56	-0.07	19	12	3UTR
hsa-miR-11400	NM_001130727	PACRGL	3807	3826	0.980769	1	-0.56	-0.07	19	12	3UTR

mirnaid	refseqid	genesymbol	start	end	bindingp	seed	phylopstem	phylopflank	binding_region_length	longest_consecutive_pairings	position
hsa-miR-11400	NM_013358	PADI1	2221	2240	1	1	0.00	0.00	19	12	3UTR
hsa-miR-11400	NM_002571	PAEP	808	834	1	1	-0.31	-0.12	14	12	3UTR
hsa-miR-11400	NM_001018049	PAEP	837	863	1	1	-0.31	-0.12	14	12	3UTR
hsa-miR-11400	XM_006710670	PAFAH2	2087	2114	1	1	0.00	0.00	27	12	3UTR
hsa-miR-11400	NM_002576	PAK1	3230	3243	1	1	-0.23	0.26	13	12	3UTR
hsa-miR-11400	NM_001128620	PAK1	3279	3292	1	1	-0.23	0.26	13	12	3UTR
hsa-miR-11400	NM_002577	PAK2	3047	3095	1	1	0.57	0.59	19	8	3UTR
hsa-miR-11400	XM_017029563	PAK3	5975	5997	1	1	0.00	0.00	22	8	3UTR
hsa-miR-11400	NM_002578	PAK3	5823	5845	1	1	2.82	1.37	22	8	3UTR
hsa-miR-11400	NM_002578	PAK3	8246	8266	1	1	0.77	0.59	20	11	3UTR
hsa-miR-11400	NM_001324328	PAK3	5653	5675	1	1	2.82	1.37	22	8	3UTR
hsa-miR-11400	NM_001324328	PAK3	8076	8096	1	1	0.77	0.59	20	11	3UTR
hsa-miR-11400	NM_001324329	PAK3	5868	5890	1	1	2.82	1.37	22	8	3UTR
hsa-miR-11400	NM_001324329	PAK3	8291	8311	1	1	0.77	0.59	20	11	3UTR
hsa-miR-11400	NM_001128166	PAK3	5578	5600	1	1	1.44	1.01	22	8	3UTR
hsa-miR-11400	NM_001128166	PAK3	8001	8021	1	1	0.63	1.32	20	11	3UTR
hsa-miR-11400	NM_001128167	PAK3	5505	5527	1	1	1.44	1.01	22	8	3UTR

mirnaid	refseqid	genesymbol	start	end	bindingp	seed	phylopstem	phylopflank	binding_region_length	longest_consecutive_pairings	position
hsa-miR-11400	NM_001128167	PAK3	7928	7948	1	1	0.63	1.32	20	11	3UTR
hsa-miR-11400	NM_001128168	PAK3	5934	5956	1	1	2.82	1.37	22	8	3UTR
hsa-miR-11400	NM_001128168	PAK3	8357	8377	1	1	0.77	0.59	20	11	3UTR
hsa-miR-11400	NM_001128172	PAK3	5336	5358	1	1	0.00	0.00	22	8	3UTR
hsa-miR-11400	NM_014431	PALD1	3062	3083	1	1	0.49	-0.10	21	8	3UTR
hsa-miR-11400	NM_053016	PALM2AKAP2	2149	2172	0.974359	1	0.79	1.18	23	9	3UTR
hsa-miR-11400	NM_001037293	PALM2AKAP2	1837	1860	0.974359	1	0.89	1.17	23	9	3UTR
hsa-miR-11400	NM_053016	PALM2AKAP2	4721	4746	1	1	-0.37	0.05	25	8	3UTR
hsa-miR-11400	NM_147150	PALM2AKAP2	5966	5989	1	1	-0.56	-0.37	23	9	3UTR
hsa-miR-11400	NM_007203	PALM2AKAP2	6005	6028	1	1	-0.56	-0.37	23	9	3UTR
hsa-miR-11400	NM_001004065	PALM2AKAP2	5351	5374	1	1	-0.56	-0.37	23	9	3UTR
hsa-miR-11400	NM_001037293	PALM2AKAP2	4409	4434	1	1	-0.45	0.03	25	8	3UTR
hsa-miR-11400	NM_001136562	PALM2AKAP2	5189	5212	1	1	-0.56	-0.37	23	9	3UTR
hsa-miR-11400	NM_001198656	PALM2AKAP2	5390	5413	1	1	-0.56	-0.37	23	9	3UTR
hsa-miR-11400	NM_153640	PANK2	4124	4141	1	1	-0.13	-0.12	17	9	3UTR
hsa-miR-11400	XM_017028079	PANK2	3959	3976	1	1	0.00	0.00	17	9	3UTR
hsa-miR-11400	NM_024960	PANK2	4131	4148	1	1	-0.13	-0.17	17	9	3UTR

mirnaid	refseqid	genesymbol	start	end	bindingp	seed	phylopstem	phylopflank	binding_region_length	longest_consecutive_pairings	position
hsa-miR-11400	NM_024594	PANK3	3231	3250	1	1	0.36	0.00	19	7	3UTR
hsa-miR-11400	NM_022894	PAPOLG	5386	5405	1	1	0.30	0.24	19	16	3UTR
hsa-miR-11400	NM_022894	PAPOLG	3800	3817	1	1	0.47	0.64	17	9	3UTR
hsa-miR-11400	NM_021936	PAPPA2	3855	3874	1	1	0.15	-0.11	19	9	3UTR
hsa-miR-11400	NM_001272107	PAQR6	1578	1606	1	1	3.74	2.93	28	10	3UTR
hsa-miR-11400	NM_198406	PAQR6	1401	1429	1	1	2.07	2.68	28	10	3UTR
hsa-miR-11400	NM_001302769	PARD3B	7082	7111	1	1	0.54	0.16	20	8	3UTR
hsa-miR-11400	NM_057177	PARD3B	6875	6904	1	1	0.54	0.16	20	8	3UTR
hsa-miR-11400	NM_152526	PARD3B	6896	6925	1	1	0.54	0.16	20	8	3UTR
hsa-miR-11400	NM_205863	PARD3B	6779	6808	1	1	0.54	0.16	20	8	3UTR
hsa-miR-11400	NM_001146106	PARP9	2646	2664	1	1	-0.35	0.12	18	17	3UTR
hsa-miR-11400	NM_018222	PARVA	4468	4492	1	1	0.00	0.01	24	12	3UTR
hsa-miR-11400	NM_001280549	PAX5	4061	4081	1	1	0.66	0.33	20	10	3UTR
hsa-miR-11400	NM_001280550	PAX5	3974	3994	1	1	0.04	0.10	20	10	3UTR
hsa-miR-11400	NM_001135254	PAX7	5736	5762	1	1	0.00	0.00	26	12	3UTR
hsa-miR-11400	NM_002585	PBX1	3023	3040	1	1	0.46	-0.04	17	10	3UTR
hsa-miR-11400	NM_001353130	PBX1	2411	2428	1	1	0.46	-0.04	17	10	3UTR

mirnaid	refseqid	genesymbol	start	end	bindingp	seed	phylopstem	phylopflank	binding_region_length	longest_consecutive_pairings	position
hsa-miR-11400	NM_001204961	PBX1	2910	2927	1	1	-0.16	-0.13	17	10	3UTR
hsa-miR-11400	NM_002586	PBX2	1831	1855	1	1	1.52	3.60	24	7	3UTR
hsa-miR-11400	NM_001348244	PCBP3	1571	1590	1	1	-0.03	0.16	19	8	3UTR
hsa-miR-11400	NM_001382278	PCBP3	1628	1647	1	1	-0.03	0.16	19	8	3UTR
hsa-miR-11400	NM_001382279	PCBP3	1700	1719	1	1	-0.03	0.16	19	8	3UTR
hsa-miR-11400	NM_001382287	PCBP3	1561	1580	1	1	-0.03	0.16	19	8	3UTR
hsa-miR-11400	NM_001130141	PCBP3	1553	1572	1	1	-0.03	0.16	19	8	3UTR
hsa-miR-11400	XM_017006918	PCBP4	1696	1713	1	1	0.00	0.00	17	16	3UTR
hsa-miR-11400	XM_017006919	PCBP4	1456	1473	1	1	0.00	0.00	17	16	3UTR
hsa-miR-11400	NM_001318374	PCDH9	16569	16589	1	1	0.33	0.44	20	11	3UTR
hsa-miR-11400	NM_031859	PCDHA10	6859	6883	1	1	0.23	1.09	24	12	3UTR
hsa-miR-11400	NM_031993	PCDHGA1	3577	3593	1	1	0.00	0.00	16	15	3UTR
hsa-miR-11400	NM_001286782	PCMTD1	1564	1588	1	1	0.87	0.60	24	9	3UTR
hsa-miR-11400	NM_020357	PCNP	1933	1951	0.961538	1	1.19	0.90	18	6	3UTR
hsa-miR-11400	NM_006200	PCSK5	4016	4056	1	1	0.31	0.52	27	14	3UTR
hsa-miR-11400	NM_016297	PCYOX1	3109	3128	1	1	0.14	0.09	19	9	3UTR
hsa-miR-11400	NM_002597	PDC	901	923	1	1	4.23	4.46	22	8	3UTR

mirnaid	refseqid	genesymbol	start	end	bindingp	seed	phylopstem	phylopflank	binding_region_length	longest_consecutive_pairings	position
hsa-miR-11400	NM_001267559	PDCD6	310	332	1	1	4.74	4.95	22	8	3UTR
hsa-miR-11400	NM_016953	PDE11A	6151	6168	1	1	0.28	0.52	17	13	3UTR
hsa-miR-11400	NM_001077358	PDE11A	4870	4887	1	1	0.58	0.19	17	13	3UTR
hsa-miR-11400	NM_001288769	PDE1B	1958	1977	0.974359	1	1.57	1.32	19	14	3UTR
hsa-miR-11400	NM_001315534	PDE1B	1908	1927	0.974359	1	1.57	1.32	19	14	3UTR
hsa-miR-11400	NM_000924	PDE1B	2130	2149	0.974359	1	1.57	1.32	19	14	3UTR
hsa-miR-11400	NM_001165975	PDE1B	2005	2024	0.974359	1	1.57	1.32	19	14	3UTR
hsa-miR-11400	NM_001142386	PDK3	1666	1688	1	1	0.26	0.29	22	8	3UTR
hsa-miR-11400	XM_024450296	PDPK1	6214	6261	1	1	0.00	0.00	22	15	3UTR
hsa-miR-11400	NM_198389	PDPN	1794	1827	1	1	0.07	-0.02	33	10	3UTR
hsa-miR-11400	NM_198389	PDPN	2131	2149	1	1	0.57	-0.11	18	8	3UTR
hsa-miR-11400	XM_006710295	PDPN	1531	1564	1	1	0.00	0.00	33	10	3UTR
hsa-miR-11400	NM_006474	PDPN	1725	1758	1	1	0.07	-0.02	33	10	3UTR
hsa-miR-11400	NM_006474	PDPN	2062	2080	1	1	0.57	-0.11	18	8	3UTR
hsa-miR-11400	NM_001006624	PDPN	1636	1669	1	1	0.07	-0.02	33	10	3UTR
hsa-miR-11400	NM_001006624	PDPN	1973	1991	1	1	0.57	-0.11	18	8	3UTR
hsa-miR-11400	NM_001006625	PDPN	1630	1663	1	1	0.07	-0.02	33	10	3UTR

mirnaid	refseqid	genesymbol	start	end	bindingp	seed	phylopstem	phylopflank	binding_region_length	longest_consecutive_pairings	position
hsa-miR-11400	NM_001006625	PDPN	1967	1985	1	1	0.57	-0.11	18	8	3UTR
hsa-miR-11400	XM_005270165	PDZD7	2037	2055	0.961538	1	0.00	0.00	18	8	3UTR
hsa-miR-11400	NM_001297576	PEA15	2643	2688	1	1	0.80	1.62	17	10	3UTR
hsa-miR-11400	NM_001297578	PEA15	2299	2315	1	1	1.80	1.63	16	10	3UTR
hsa-miR-11400	NM_003768	PEA15	2365	2381	1	1	1.80	1.63	16	10	3UTR
hsa-miR-11400	NM_000442	PECAM1	5734	5754	1	1	0.00	0.88	20	11	3UTR
hsa-miR-11400	NM_001282327	PES1	2524	2544	1	1	-0.05	0.20	20	13	3UTR
hsa-miR-11400	NM_001282328	PES1	2571	2591	1	1	-0.05	0.20	20	13	3UTR
hsa-miR-11400	NM_001243225	PES1	2081	2101	1	1	0.00	0.00	20	13	3UTR
hsa-miR-11400	NM_003846	PEX11B	1229	1252	1	1	3.37	3.46	23	10	3UTR
hsa-miR-11400	NM_001270539	PEX11G	581	598	1	1	2.85	3.65	17	16	3UTR
hsa-miR-11400	NM_080662	PEX11G	764	781	1	1	2.85	3.65	17	16	3UTR
hsa-miR-11400	XM_011528428	PEX11G	1026	1043	1	1	0.00	0.00	17	16	3UTR
hsa-miR-11400	NM_002618	PEX13	2157	2207	1	1	-0.08	0.04	26	8	3UTR
hsa-miR-11400	NM_017929	PEX26	15993	16018	1	1	-0.09	-0.13	25	9	3UTR
hsa-miR-11400	NM_001127649	PEX26	16241	16266	1	1	-0.09	-0.13	25	9	3UTR
hsa-miR-11400	NM_001199319	PEX26	15846	15871	1	1	-0.09	-0.13	25	9	3UTR

mirnaid	refseqid	genesymbol	start	end	bindingp	seed	phylopstem	phylopflank	binding_region_length	longest_consecutive_pairings	position
hsa-miR-11400	NM_001256754	PEX5L	7814	7831	1	1	3.02	4.33	17	12	3UTR
hsa-miR-11400	NM_001354735	PFKM	3411	3433	1	1	-0.28	0.25	22	6	3UTR
hsa-miR-11400	NM_001354743	PFKM	3294	3316	1	1	0.00	0.00	22	6	3UTR
hsa-miR-11400	NM_000289	PFKM	3007	3029	1	1	-0.28	0.25	22	6	3UTR
hsa-miR-11400	NM_001166686	PFKM	3393	3415	1	1	-0.28	0.25	22	6	3UTR
hsa-miR-11400	NM_001166687	PFKM	2995	3017	1	1	-0.28	0.25	22	6	3UTR
hsa-miR-11400	NM_001166688	PFKM	3084	3106	1	1	-0.28	0.25	22	6	3UTR
hsa-miR-11400	NM_024989	PGAP1	5601	5626	1	1	0.42	-0.01	25	10	3UTR
hsa-miR-11400	NM_001256238	PGAP2	961	980	1	1	0.33	1.90	19	13	3UTR
hsa-miR-11400	NM_001256239	PGAP2	868	887	1	1	0.33	1.90	19	13	3UTR
hsa-miR-11400	NM_014489	PGAP2	1063	1082	1	1	0.33	1.90	19	13	3UTR
hsa-miR-11400	NM_001346404	PGAP2	967	986	1	1	0.33	1.90	19	13	3UTR
hsa-miR-11400	NM_001258311	PGBD5	10628	10649	1	1	0.42	0.29	21	9	3UTR
hsa-miR-11400	NM_001207012	PGF	1166	1185	1	1	5.91	3.14	19	7	3UTR
hsa-miR-11400	NM_000291	PGK1	1454	1474	1	1	0.84	1.13	20	9	3UTR
hsa-miR-11400	NM_173582	PGM2L1	4841	4860	0.961538	1	0.07	-0.06	19	7	3UTR
hsa-miR-11400	NM_001367286	PGM3	3550	3570	1	1	2.01	1.61	20	9	3UTR

mirnaid	refseqid	genesymbol	start	end	bindingp	seed	phylopstem	phylopflank	binding_region_length	longest_consecutive_pairings	position
hsa-miR-11400	NM_001271161	PGR	6536	6555	1	1	-0.24	-0.06	19	10	3UTR
hsa-miR-11400	NM_001271161	PGR	5982	6009	1	1	0.73	0.21	27	8	3UTR
hsa-miR-11400	NM_001271162	PGR	5598	5617	1	1	0.09	-0.20	19	10	3UTR
hsa-miR-11400	NM_000926	PGR	7038	7065	1	1	0.74	0.27	27	8	3UTR
hsa-miR-11400	XM_005253334	PHC1	4517	4538	1	1	0.00	0.00	21	9	3UTR
hsa-miR-11400	NM_001177996	PHETA1	3160	3181	1	1	0.29	0.38	21	9	3UTR
hsa-miR-11400	NM_001177997	PHETA1	3053	3074	1	1	0.29	0.38	21	9	3UTR
hsa-miR-11400	NM_014660	PHF14	4071	4085	1	1	1.61	1.33	14	13	3UTR
hsa-miR-11400	NM_016621	PHF21A	6209	6235	1	1	2.23	4.01	26	10	3UTR
hsa-miR-11400	NM_001101802	PHF21A	6347	6373	1	1	2.23	4.01	26	10	3UTR
hsa-miR-11400	NM_138415	PHF21B	3474	3492	1	1	1.74	2.36	18	13	3UTR
hsa-miR-11400	NM_015297	PHF24	2205	2221	1	1	-0.26	0.40	16	7	3UTR
hsa-miR-11400	NM_015153	PHF3	11181	11215	1	1	-0.18	-0.34	34	10	3UTR
hsa-miR-11400	NM_001370348	PHF3	11529	11563	1	1	-0.18	-0.34	34	10	3UTR
hsa-miR-11400	NM_032458	PHF6	2952	2976	1	1	1.30	1.85	24	8	3UTR
hsa-miR-11400	NM_001015877	PHF6	2622	2646	1	1	1.30	1.85	24	8	3UTR
hsa-miR-11400	NM_001184896	PHF8	4370	4385	1	1	0.00	0.00	15	14	3UTR

mirnaid	refseqid	genesymbol	start	end	bindingp	seed	phylopstem	phylopflank	binding_region_length	longest_consecutive_pairings	position
hsa-miR-11400	NM_000292	PHKA2	4060	4078	1	1	4.28	5.24	18	10	3UTR
hsa-miR-11400	NM_001258459	PHKG1	1782	1828	1	1	3.98	3.41	46	10	3UTR
hsa-miR-11400	NM_006213	PHKG1	1686	1732	1	1	3.75	3.69	46	10	3UTR
hsa-miR-11400	NM_003924	PHOX2B	1452	1474	1	1	0.29	0.43	22	7	3UTR
hsa-miR-11400	NM_032439	PHYHIPL	2123	2145	1	1	2.82	1.77	22	9	3UTR
hsa-miR-11400	NM_016166	PIAS1	3037	3050	1	1	-0.15	0.73	13	12	3UTR
hsa-miR-11400	NM_001320687	PIAS1	3772	3785	1	1	-0.15	0.73	13	12	3UTR
hsa-miR-11400	NM_004671	PIAS2	8012	8060	1	1	-0.04	0.01	48	8	3UTR
hsa-miR-11400	NM_001324060	PIAS2	1289	1313	1	1	0.43	2.98	24	8	3UTR
hsa-miR-11400	NM_001289053	PIGG	2586	2605	0.961538	1	0.16	-0.55	19	8	3UTR
hsa-miR-11400	NM_001289057	PIGG	2046	2065	0.961538	1	0.16	-0.55	19	8	3UTR
hsa-miR-11400	NM_152671	PIKFYVE	1544	1564	1	1	-0.08	1.69	20	8	3UTR
hsa-miR-11400	NM_001178000	PIKFYVE	1835	1855	1	1	-0.08	1.69	20	8	3UTR
hsa-miR-11400	NM_001326411	PISD	2029	2074	1	1	1.94	3.35	18	8	3UTR
hsa-miR-11400	NM_001284278	PITPNB	1011	1029	1	1	0.54	0.81	18	8	3UTR
hsa-miR-11400	NM_004764	PIWIL1	2861	2881	1	1	-1.09	1.36	20	9	3UTR
hsa-miR-11400	NM_001255975	PIWIL3	3251	3299	1	1	-0.11	-0.17	21	9	3UTR

mirnaid	refseqid	genesymbol	start	end	bindingp	seed	phylopstem	phylopflank	binding_region_length	longest_consecutive_pairings	position
hsa-miR-11400	NM_001008496	PIWIL3	3278	3326	1	1	-0.11	-0.17	21	9	3UTR
hsa-miR-11400	NM_181839	PKIA	1026	1062	1	1	0.77	1.02	36	9	3UTR
hsa-miR-11400	NM_006823	PKIA	1155	1191	1	1	0.77	1.02	36	9	3UTR
hsa-miR-11400	NM_004572	PKP2	2881	2899	0.974359	1	0.10	0.29	18	11	3UTR
hsa-miR-11400	NM_001271814	PLA2G2D	1408	1428	1	1	-0.80	-0.44	20	16	3UTR
hsa-miR-11400	NM_012400	PLA2G2D	1515	1535	1	1	-0.66	-0.66	20	16	3UTR
hsa-miR-11400	NM_014589	PLA2G2E	620	653	1	1	1.21	1.73	33	8	3UTR
hsa-miR-11400	NM_213600	PLA2G4F	3011	3031	1	1	3.15	1.30	20	8	3UTR
hsa-miR-11400	XM_005245892	PLA2G5	1060	1082	1	1	0.00	0.00	22	8	3UTR
hsa-miR-11400	NM_007366	PLA2R1	5987	6009	1	1	0.49	0.15	22	10	3UTR
hsa-miR-11400	XM_011527029	PLAUR	1045	1064	1	1	0.00	0.00	19	10	3UTR
hsa-miR-11400	NM_015184	PLCL2	3812	3832	1	1	0.28	0.56	20	13	3UTR
hsa-miR-11400	NM_019091	PLEKHA3	9480	9499	1	1	0.02	0.18	19	11	3UTR
hsa-miR-11400	NM_001037671	PLEKHG7	1153	1175	1	1	-0.02	0.16	22	12	3UTR
hsa-miR-11400	NM_001178097	PLEKHG7	1664	1686	1	1	0.55	0.63	22	12	3UTR
hsa-miR-11400	NM_020715	PLEKHH1	4603	4621	1	1	-0.15	0.10	18	14	3UTR
hsa-miR-11400	NM_000301	PLG	2855	2874	1	1	-0.10	-0.44	19	8	3UTR

mirnaid	refseqid	genesymbol	start	end	bindingp	seed	phylopstem	phylopflank	binding_region_length	longest_consecutive_pairings	position
hsa-miR-11400	NM_005817	PLIN3	1901	1924	1	1	2.36	1.56	23	8	3UTR
hsa-miR-11400	NM_001164189	PLIN3	1898	1921	1	1	2.36	1.56	23	8	3UTR
hsa-miR-11400	NM_001164194	PLIN3	1865	1888	1	1	2.36	1.56	23	8	3UTR
hsa-miR-11400	NM_020353	PLSCR4	2300	2329	1	1	3.67	1.25	29	8	3UTR
hsa-miR-11400	NM_001128304	PLSCR4	2426	2455	1	1	4.10	2.39	29	8	3UTR
hsa-miR-11400	NM_001128305	PLSCR4	2213	2242	1	1	0.26	2.15	29	8	3UTR
hsa-miR-11400	XM_005250686	PLXNA4	12756	12780	1	1	0.00	0.00	24	11	3UTR
hsa-miR-11400	NM_199169	PMEPA1	4205	4224	1	1	0.39	3.16	19	8	3UTR
hsa-miR-11400	NM_199171	PMEPA1	4236	4255	1	1	4.79	2.67	19	8	3UTR
hsa-miR-11400	NM_015488	PNKD	1649	1669	1	1	-0.08	0.05	20	8	3UTR
hsa-miR-11400	NM_022572	PNKD	1649	1669	1	1	-0.08	0.05	20	8	3UTR
hsa-miR-11400	NM_007257	PNMA2	2917	2965	1	1	0.40	-0.29	17	7	3UTR
hsa-miR-11400	XM_017001691	PNRC2	1134	1175	1	1	0.00	0.00	15	7	3UTR
hsa-miR-11400	NM_017761	PNRC2	1239	1253	1	1	0.93	0.08	14	7	3UTR
hsa-miR-11400	NM_001146254	PODNL1	2889	2911	1	1	0.26	0.60	22	10	3UTR
hsa-miR-11400	NM_001146255	PODNL1	2622	2644	1	1	0.26	0.60	22	10	3UTR
hsa-miR-11400	NM_153705	POGLUT3	2130	2150	0.961538	1	-0.06	0.31	20	15	3UTR

mirnaid	refseqid	genesymbol	start	end	bindingp	seed	phylopstem	phylopflank	binding_region_length	longest_consecutive_pairings	position
hsa-miR-11400	XM_011542621	POGLUT3	2151	2171	1	1	0.00	0.00	20	15	3UTR
hsa-miR-11400	NM_007195	POLI	5788	5803	1	1	-0.07	0.11	15	14	3UTR
hsa-miR-11400	NM_001351613	POLI	5571	5604	1	1	-0.07	0.16	16	14	3UTR
hsa-miR-11400	NM_001351615	POLI	5976	5991	1	1	-0.07	0.11	15	14	3UTR
hsa-miR-11400	XM_006717777	POLL	2164	2187	1	1	0.00	0.00	21	7	3UTR
hsa-miR-11400	XM_011539651	POLL	2203	2226	1	1	0.00	0.00	21	7	3UTR
hsa-miR-11400	XM_011539654	POLL	1948	1971	1	1	0.00	0.00	21	7	3UTR
hsa-miR-11400	XM_024447943	POLL	2443	2466	1	1	0.00	0.00	21	7	3UTR
hsa-miR-11400	NM_001282774	POLR1B	5420	5443	1	1	0.59	0.01	18	10	3UTR
hsa-miR-11400	NM_001002926	POLR1F	2029	2049	1	1	0.15	0.12	20	10	3UTR
hsa-miR-11400	NM_005034	POLR2K	371	391	1	1	0.03	0.45	20	10	3UTR
hsa-miR-11400	NM_001722	POLR3D	3038	3056	1	1	-0.52	-0.05	18	11	3UTR
hsa-miR-11400	NM_001137671	POTEC	3318	3352	1	1	0.00	0.00	21	10	3UTR
hsa-miR-11400	NM_174981	POTED	3308	3342	1	1	-0.12	-0.13	21	10	3UTR
hsa-miR-11400	NM_001145442	POTEM	6024	6045	1	1	-0.07	-0.06	21	9	3UTR
hsa-miR-11400	NM_001173531	POU5F1	1556	1577	1	1	0.17	-0.07	21	7	3UTR
hsa-miR-11400	NM_001354828	PPARGC1A	1720	1744	1	1	0.05	0.74	24	12	3UTR

mirnaid	refseqid	genesymbol	start	end	bindingp	seed	phylopstem	phylopflank	binding_region_length	longest_consecutive_pairings	position
hsa-miR-11400	NM_001368128	PPIAL4H	1289	1323	1	1	-0.22	-0.08	24	9	3UTR
hsa-miR-11400	NM_001286360	PPIL6	2080	2096	1	1	-0.15	0.04	16	9	3UTR
hsa-miR-11400	NM_001286361	PPIL6	1944	1960	1	1	0.09	-0.06	16	9	3UTR
hsa-miR-11400	NM_173672	PPIL6	1622	1649	1	1	-0.35	0.09	17	9	3UTR
hsa-miR-11400	NM_001111298	PPIL6	2254	2270	1	1	0.19	0.10	16	9	3UTR
hsa-miR-11400	NM_177952	PPM1A	1839	1860	1	1	2.42	2.99	21	12	3UTR
hsa-miR-11400	NM_021003	PPM1A	1999	2020	1	1	2.42	2.99	21	12	3UTR
hsa-miR-11400	NM_003620	PPM1D	4285	4303	1	1	-0.08	0.06	18	8	3UTR
hsa-miR-11400	NM_014906	PPM1E	5710	5736	1	1	1.41	1.46	26	11	3UTR
hsa-miR-11400	NM_001271593	PPME1	2237	2257	1	1	0.85	0.76	20	7	3UTR
hsa-miR-11400	NM_016147	PPME1	2195	2215	1	1	0.85	0.76	20	7	3UTR
hsa-miR-11400	NM_001242464	PPP1R1B	992	1011	1	1	2.70	2.61	19	7	3UTR
hsa-miR-11400	NM_033215	PPP1R3F	2510	2524	1	1	0.10	1.17	14	13	3UTR
hsa-miR-11400	NM_001184745	PPP1R3F	1834	1870	1	1	3.08	2.62	28	9	3UTR
hsa-miR-11400	NM_001184745	PPP1R3F	1552	1581	1	1	1.13	1.24	15	13	3UTR
hsa-miR-11400	NM_001271948	PPP2R2B	5710	5741	1	1	0.00	0.00	31	8	3UTR
hsa-miR-11400	NM_181674	PPP2R2B	5360	5391	1	1	0.00	0.00	31	8	3UTR

mirnaid	refseqid	genesymbol	start	end	bindingp	seed	phylopstem	phylopflank	binding_region_length	longest_consecutive_pairings	position
hsa-miR-11400	NM_181675	PPP2R2B	5230	5261	1	1	0.03	-0.06	31	8	3UTR
hsa-miR-11400	NM_181676	PPP2R2B	5166	5197	1	1	0.00	0.00	31	8	3UTR
hsa-miR-11400	NM_001199756	PPP2R5A	2313	2346	1	1	4.89	3.27	33	8	3UTR
hsa-miR-11400	NM_002721	PPP6C	2743	2778	1	1	1.30	1.94	19	17	3UTR
hsa-miR-11400	NM_001123355	PPP6C	2854	2889	1	1	1.93	1.70	19	17	3UTR
hsa-miR-11400	NM_001291380	PRAMEF13	1849	1870	1	1	0.00	0.00	21	9	3UTR
hsa-miR-11400	NM_004905	PRDX6	1292	1312	1	1	0.38	0.07	20	12	3UTR
hsa-miR-11400	XM_017005384	PREPL	4002	4019	1	1	0.00	0.00	17	16	3UTR
hsa-miR-11400	NM_001374276	PREPL	3053	3070	1	1	-0.71	0.52	17	16	3UTR
hsa-miR-11400	NM_001171603	PREPL	3277	3294	1	1	0.27	0.33	17	16	3UTR
hsa-miR-11400	NM_001171606	PREPL	3341	3358	1	1	0.34	0.31	17	16	3UTR
hsa-miR-11400	NM_001110213	PRH2	2185	2215	1	1	-0.31	-0.11	15	13	3UTR
hsa-miR-11400	XM_024449482	PRIMA1	910	926	1	1	0.00	0.00	16	10	3UTR
hsa-miR-11400	NM_006252	PRKAA2	6798	6821	1	1	0.02	0.05	23	8	3UTR
hsa-miR-11400	NM_006252	PRKAA2	9268	9290	1	1	0.30	0.53	22	6	3UTR
hsa-miR-11400	XM_017004343	PRKAG3	2960	2983	1	1	0.00	0.00	23	7	3UTR
hsa-miR-11400	NM_004157	PRKAR2A	4374	4392	1	1	-0.05	0.12	18	17	3UTR

mirnaid	refseqid	genesymbol	start	end	bindingp	seed	phylopstem	phylopflank	binding_region_length	longest_consecutive_pairings	position
hsa-miR-11400	NM_001321983	PRKAR2A	4308	4326	1	1	0.15	0.32	18	17	3UTR
hsa-miR-11400	NM_024653	PRKRIP1	2113	2131	1	1	-0.01	0.00	18	10	3UTR
hsa-miR-11400	NM_006017	PROM1	3450	3493	1	1	3.02	1.86	43	10	3UTR
hsa-miR-11400	NM_001145848	PROM1	3423	3466	1	1	1.01	2.39	43	10	3UTR
hsa-miR-11400	NM_001145849	PROM1	3335	3378	1	1	2.93	3.33	43	10	3UTR
hsa-miR-11400	NM_144707	PROM2	3082	3105	1	1	-0.09	-0.12	23	7	3UTR
hsa-miR-11400	NM_001165978	PROM2	3991	4014	1	1	-0.09	-0.12	23	7	3UTR
hsa-miR-11400	NM_001039887	PROSER3	3741	3761	1	1	0.32	0.11	20	14	3UTR
hsa-miR-11400	NM_001243007	PROX2	3393	3425	1	1	0.00	0.00	21	10	3UTR
hsa-miR-11400	NM_032864	PRPF38A	4078	4099	1	1	0.38	-0.10	21	9	3UTR
hsa-miR-11400	NM_032864	PRPF38A	2969	2994	1	1	0.10	-0.24	25	7	3UTR
hsa-miR-11400	NM_017922	PRPF39	3223	3245	1	1	1.14	0.72	22	10	3UTR
hsa-miR-11400	NM_018304	PRR11	5142	5167	1	1	-0.46	-0.04	25	8	3UTR
hsa-miR-11400	NM_024320	PRR15L	923	943	1	1	-0.11	0.62	20	9	3UTR
hsa-miR-11400	NM_001134659	PRR23A	1698	1723	1	1	-0.30	-0.57	25	8	3UTR
hsa-miR-11400	NM_145270	PRR35	2181	2201	1	1	-0.41	-0.39	20	13	3UTR
hsa-miR-11400	NM_001384818	PRRC2B	10277	10313	1	1	-0.79	-0.14	20	12	3UTR

mirnaid	refseqid	genesymbol	start	end	bindingp	seed	phylopstem	phylopflank	binding_region_length	longest_consecutive_pairings	position
hsa-miR-11400	NM_001384823	PRRC2B	8113	8149	1	1	-0.79	-0.14	20	12	3UTR
hsa-miR-11400	NM_007173	PRSS23	2924	2956	1	1	0.07	-0.08	21	9	3UTR
hsa-miR-11400	NM_032333	PRXL2A	3442	3462	1	1	0.42	0.11	20	8	3UTR
hsa-miR-11400	NM_001243778	PRXL2A	3393	3413	1	1	0.00	0.00	20	8	3UTR
hsa-miR-11400	NM_001243779	PRXL2A	3477	3497	1	1	0.00	0.00	20	8	3UTR
hsa-miR-11400	NM_001243781	PRXL2A	3425	3445	1	1	0.42	0.11	20	8	3UTR
hsa-miR-11400	NM_001085382	PSAPL1	4152	4170	1	1	-0.07	-0.29	18	13	3UTR
hsa-miR-11400	NM_001330524	PSG1	1594	1611	1	1	0.57	0.16	17	12	3UTR
hsa-miR-11400	NM_001184825	PSG1	1873	1890	1	1	0.57	0.16	17	12	3UTR
hsa-miR-11400	NM_001184826	PSG1	1787	1804	1	1	0.57	0.16	17	12	3UTR
hsa-miR-11400	NM_001206650	PSG7	1511	1528	1	1	0.00	0.00	17	12	3UTR
hsa-miR-11400	XM_011526987	PSG8	1632	1649	1	1	0.00	0.00	17	12	3UTR
hsa-miR-11400	NM_001130167	PSG8	1837	1854	1	1	-0.19	-0.01	17	12	3UTR
hsa-miR-11400	NM_001130168	PSG8	1471	1488	1	1	0.34	-0.04	17	12	3UTR
hsa-miR-11400	XM_005259075	PSG9	1797	1821	1	1	0.00	0.00	24	6	3UTR
hsa-miR-11400	XM_017027006	PSG9	1518	1542	1	1	0.00	0.00	24	6	3UTR
hsa-miR-11400	NM_153001	PSMC4	1415	1444	1	1	0.36	0.29	21	13	3UTR

mirnaid	refseqid	genesymbol	start	end	bindingp	seed	phylopstem	phylopflank	binding_region_length	longest_consecutive_pairings	position
hsa-miR-11400	NM_006503	PSMC4	1508	1537	1	1	0.13	0.21	21	13	3UTR
hsa-miR-11400	NM_024946	PSME3IP1	2308	2326	1	1	4.89	4.50	18	9	3UTR
hsa-miR-11400	NM_001354080	PSME3IP1	2526	2544	1	1	3.92	4.28	18	9	3UTR
hsa-miR-11400	NM_001354083	PSME3IP1	2304	2322	1	1	3.97	4.47	18	9	3UTR
hsa-miR-11400	NM_178578	PSMF1	3003	3023	1	1	-0.55	0.29	20	8	3UTR
hsa-miR-11400	NM_006814	PSMF1	3007	3027	1	1	-0.41	0.27	20	8	3UTR
hsa-miR-11400	NM_001323408	PSMF1	1809	1829	1	1	-0.41	0.27	20	8	3UTR
hsa-miR-11400	NM_024430	PSTPIP2	1396	1414	1	1	0.53	0.28	18	14	3UTR
hsa-miR-11400	NM_001300985	PTBP2	9275	9299	1	1	0.18	-0.13	24	9	3UTR
hsa-miR-11400	NM_001300986	PTBP2	9284	9308	1	1	0.18	-0.13	24	9	3UTR
hsa-miR-11400	NM_001300989	PTBP2	9260	9284	1	1	0.18	-0.13	24	9	3UTR
hsa-miR-11400	NM_001284403	PTCD2	9050	9068	1	1	0.05	0.14	18	8	3UTR
hsa-miR-11400	NM_001284404	PTCD2	9180	9198	1	1	0.00	0.00	18	8	3UTR
hsa-miR-11400	NM_001284405	PTCD2	9298	9316	1	1	0.00	0.00	18	8	3UTR
hsa-miR-11400	NM_024754	PTCD2	9377	9395	1	1	0.05	0.14	18	8	3UTR
hsa-miR-11400	NM_173495	PTCHD1	3891	3910	0.961538	1	0.85	0.24	19	7	3UTR
hsa-miR-11400	NM_173495	PTCHD1	4710	4744	1	1	1.05	1.13	34	10	3UTR

mirnaid	refseqid	genesymbol	start	end	bindingp	seed	phylopstem	phylopflank	binding_region_length	longest_consecutive_pairings	position
hsa-miR-11400	NM_001013732	PTCHD4	19531	19549	1	1	1.56	0.18	18	10	3UTR
hsa-miR-11400	NM_001013732	PTCHD4	2755	2773	1	1	-0.22	0.05	18	6	3UTR
hsa-miR-11400	NM_001261836	PTER	3235	3250	1	1	0.86	0.29	15	9	3UTR
hsa-miR-11400	NM_001261838	PTER	2755	2770	1	1	0.86	0.29	15	9	3UTR
hsa-miR-11400	XM_017016930	PTER	3131	3146	1	1	0.00	0.00	15	9	3UTR
hsa-miR-11400	NM_001001484	PTER	3369	3384	1	1	0.86	0.29	15	9	3UTR
hsa-miR-11400	NM_198715	PTGER3	4726	4748	1	1	-0.06	-0.10	22	10	3UTR
hsa-miR-11400	NM_173174	PTK2B	4487	4507	1	1	0.07	-0.05	20	8	3UTR
hsa-miR-11400	NM_173175	PTK2B	3704	3724	1	1	0.07	-0.05	20	8	3UTR
hsa-miR-11400	NM_173176	PTK2B	3842	3862	1	1	0.07	-0.05	20	8	3UTR
hsa-miR-11400	NM_152880	PTK7	3744	3760	1	1	-0.58	0.04	16	15	3UTR
hsa-miR-11400	NM_152881	PTK7	3474	3490	1	1	-0.58	0.04	16	15	3UTR
hsa-miR-11400	NM_152882	PTK7	3696	3712	1	1	-0.58	0.04	16	15	3UTR
hsa-miR-11400	NM_002821	PTK7	3864	3880	1	1	-0.58	0.04	16	15	3UTR
hsa-miR-11400	NM_080391	PTP4A2	3810	3852	1	1	0.77	0.86	42	10	3UTR
hsa-miR-11400	NM_001369859	PTP4A2	3702	3744	1	1	0.13	0.15	42	10	3UTR
hsa-miR-11400	NM_001195100	PTP4A2	3735	3777	1	1	0.77	0.86	42	10	3UTR

mirnaid	refseqid	genesymbol	start	end	bindingp	seed	phylopstem	phylopflank	binding_region_length	longest_consecutive_pairings	position
hsa-miR-11400	XM_017001936	PTPN7	2450	2470	1	1	0.00	0.00	20	11	3UTR
hsa-miR-11400	NM_001199797	PTPN7	2272	2292	1	1	0.00	0.00	20	11	3UTR
hsa-miR-11400	NM_130435	PTPRE	4206	4227	1	1	-0.50	-0.63	21	7	3UTR
hsa-miR-11400	NM_002840	PTPRF	6180	6196	0.961538	1	1.70	1.63	16	15	3UTR
hsa-miR-11400	NM_130440	PTPRF	6153	6169	1	1	1.46	2.14	16	15	3UTR
hsa-miR-11400	NM_130440	PTPRF	7439	7462	1	1	1.54	1.60	23	12	3UTR
hsa-miR-11400	NM_002840	PTPRF	7466	7489	1	1	2.38	1.12	23	12	3UTR
hsa-miR-11400	NM_001199763	PTPRN	3123	3149	1	1	0.00	0.00	20	11	3UTR
hsa-miR-11400	NM_001145026	PTPRQ	7551	7575	1	1	2.81	3.06	24	8	3UTR
hsa-miR-11400	NM_002849	PTPRR	2543	2578	1	1	1.96	3.59	35	8	3UTR
hsa-miR-11400	NM_001271826	PUS7L	9815	9843	0.980769	1	0.28	0.06	20	12	3UTR
hsa-miR-11400	NM_031292	PUS7L	10741	10769	0.980769	1	0.41	0.05	20	12	3UTR
hsa-miR-11400	NM_001098614	PUS7L	10770	10798	0.980769	1	-0.11	0.08	20	12	3UTR
hsa-miR-11400	NM_006505	PVR	1717	1731	1	1	-1.09	-0.74	14	13	3UTR
hsa-miR-11400	NM_001135768	PVR	1582	1596	1	1	-1.09	-0.74	14	13	3UTR
hsa-miR-11400	NM_001135769	PVR	1558	1572	1	1	-1.09	-0.74	14	13	3UTR
hsa-miR-11400	NM_052927	PWWP2A	1778	1799	1	1	0.48	0.61	21	14	3UTR

mirnaid	refseqid	genesymbol	start	end	bindingp	seed	phylopstem	phylopflank	binding_region_length	longest_consecutive_pairings	position
hsa-miR-11400	NM_001130864	PWWP2A	2579	2598	1	1	2.03	2.33	19	9	3UTR
hsa-miR-11400	NM_007238	PXMP4	2571	2594	1	1	-0.11	-0.16	23	7	3UTR
hsa-miR-11400	NM_002862	PYGB	3888	3906	1	1	-0.45	-0.24	18	11	3UTR
hsa-miR-11400	NM_198880	QRICH1	2526	2546	1	1	2.88	3.55	20	7	3UTR
hsa-miR-11400	NM_017730	QRICH1	2586	2606	1	1	2.98	3.20	20	7	3UTR
hsa-miR-11400	NM_001320580	QRICH1	2782	2802	1	1	3.68	3.82	20	7	3UTR
hsa-miR-11400	NM_001076786	QSER1	8331	8352	1	1	0.31	0.26	21	11	3UTR
hsa-miR-11400	NM_001076786	QSER1	7706	7729	1	1	0.39	0.57	23	9	3UTR
hsa-miR-11400	NM_001256835	QTRT2	1671	1702	1	1	0.19	0.24	19	12	3UTR
hsa-miR-11400	NM_001256836	QTRT2	1317	1348	1	1	0.19	0.24	19	12	3UTR
hsa-miR-11400	NM_001256837	QTRT2	1415	1446	1	1	0.19	0.24	19	12	3UTR
hsa-miR-11400	NM_024638	QTRT2	1779	1810	1	1	0.19	0.24	19	12	3UTR
hsa-miR-11400	NM_004663	RAB11A	3421	3435	1	1	0.88	0.40	14	13	3UTR
hsa-miR-11400	NM_004914	RAB36	1666	1686	1	1	-0.48	-0.45	20	10	3UTR
hsa-miR-11400	NM_001349878	RAB36	1799	1819	1	1	-0.48	-0.45	20	10	3UTR
hsa-miR-11400	NM_001330471	RAB37	780	799	1	1	0.71	0.34	19	11	3UTR
hsa-miR-11400	NM_001006638	RAB37	718	737	1	1	0.71	0.34	19	11	3UTR

mirnaid	refseqid	genesymbol	start	end	bindingp	seed	phylopstem	phylopflank	binding_region_length	longest_consecutive_pairings	position
hsa-miR-11400	NM_001163989	RAB37	906	925	1	1	0.71	0.34	19	11	3UTR
hsa-miR-11400	NM_001163990	RAB37	607	626	1	1	0.71	0.34	19	11	3UTR
hsa-miR-11400	NM_002867	RAB3B	8522	8541	1	1	0.17	0.28	19	7	3UTR
hsa-miR-11400	NM_001278402	RAB3IP	6706	6731	1	1	0.36	-0.01	25	9	3UTR
hsa-miR-11400	NM_175623	RAB3IP	7585	7610	1	1	0.36	-0.01	25	9	3UTR
hsa-miR-11400	NM_175624	RAB3IP	7168	7193	1	1	0.36	-0.01	25	9	3UTR
hsa-miR-11400	NM_175625	RAB3IP	7485	7510	1	1	0.36	-0.01	25	9	3UTR
hsa-miR-11400	NM_022456	RAB3IP	7268	7293	1	1	0.36	-0.01	25	9	3UTR
hsa-miR-11400	NM_001024647	RAB3IP	6791	6816	1	1	0.36	-0.01	25	9	3UTR
hsa-miR-11400	NM_006822	RAB40B	3549	3566	1	1	3.57	2.22	17	13	3UTR
hsa-miR-11400	NM_001204885	RAB43	2916	2935	0.969231	1	0.07	0.19	19	10	3UTR
hsa-miR-11400	NM_001204886	RAB43	2953	2972	0.969231	1	0.28	-0.01	19	10	3UTR
hsa-miR-11400	NM_001204884	RAB43	2926	2945	1	1	-0.26	0.11	19	10	3UTR
hsa-miR-11400	NM_001257357	RAB44	3250	3268	1	1	-0.19	-0.29	18	17	3UTR
hsa-miR-11400	NM_016577	RAB6B	2936	2955	1	1	0.80	0.09	19	14	3UTR
hsa-miR-11400	NM_001363953	RAB6B	3032	3051	1	1	0.32	-0.13	19	14	3UTR
hsa-miR-11400	NM_005370	RAB8A	1750	1770	1	1	0.23	-0.08	20	7	3UTR

mirnaid	refseqid	genesymbol	start	end	bindingp	seed	phylopstem	phylopflank	binding_region_length	longest_consecutive_pairings	position
hsa-miR-11400	NM_016370	RAB9B	2578	2594	1	1	1.36	1.35	16	15	3UTR
hsa-miR-11400	NM_012197	RABGAP1	4374	4394	1	1	0.21	0.39	20	14	3UTR
hsa-miR-11400	XM_017002996	RABGAP1L	3483	3504	1	1	0.00	0.00	21	14	3UTR
hsa-miR-11400	NM_001306161	RABL2A	840	861	1	1	-0.25	-0.48	21	12	3UTR
hsa-miR-11400	NM_013412	RABL2A	1025	1046	1	1	-0.25	-0.48	21	12	3UTR
hsa-miR-11400	NM_001354410	RABL2A	1059	1080	1	1	-0.25	-0.48	21	12	3UTR
hsa-miR-11400	NM_001354412	RABL2A	1032	1053	1	1	-0.25	-0.48	21	12	3UTR
hsa-miR-11400	NM_001354421	RABL2A	836	857	1	1	-0.25	-0.48	21	12	3UTR
hsa-miR-11400	NM_007081	RABL2B	1026	1047	1	1	-0.18	-0.39	21	12	3UTR
hsa-miR-11400	NM_001003789	RABL2B	1033	1054	1	1	-0.19	-0.36	21	12	3UTR
hsa-miR-11400	NM_001130921	RABL2B	1281	1302	1	1	-0.15	-0.10	21	12	3UTR
hsa-miR-11400	NM_001130923	RABL2B	1215	1236	1	1	-0.26	-0.30	21	12	3UTR
hsa-miR-11400	XM_011521858	RAD51	1876	1897	1	1	0.00	0.00	21	6	3UTR
hsa-miR-11400	NM_006479	RAD51AP1	1760	1778	1	1	-0.38	0.15	18	8	3UTR
hsa-miR-11400	NM_001130862	RAD51AP1	1811	1829	1	1	-0.38	0.15	18	8	3UTR
hsa-miR-11400	NM_001286531	RAD9B	3570	3590	1	1	0.64	0.19	20	7	3UTR
hsa-miR-11400	NM_001286532	RAD9B	3592	3612	1	1	0.64	0.19	20	7	3UTR

mirnaid	refseqid	genesymbol	start	end	bindingp	seed	phylopstem	phylopflank	binding_region_length	longest_consecutive_pairings	position
hsa-miR-11400	NM_001286535	RAD9B	3599	3619	1	1	0.64	0.19	20	7	3UTR
hsa-miR-11400	NM_001286536	RAD9B	2343	2363	1	1	0.64	0.19	20	7	3UTR
hsa-miR-11400	NM_152442	RAD9B	2659	2679	1	1	0.64	0.19	20	7	3UTR
hsa-miR-11400	NM_000448	RAG1	6058	6080	1	1	0.19	0.57	22	12	3UTR
hsa-miR-11400	NM_001286247	RALGPS2	2403	2431	1	1	2.14	1.88	28	7	3UTR
hsa-miR-11400	NM_152663	RALGPS2	2716	2736	1	1	0.79	0.57	20	12	3UTR
hsa-miR-11400	NM_016732	RALY	2252	2295	1	1	-0.27	-0.11	22	8	3UTR
hsa-miR-11400	NM_007367	RALY	2204	2247	1	1	-0.27	-0.11	22	8	3UTR
hsa-miR-11400	NM_001320239	RANBP10	3286	3313	1	1	1.71	1.12	27	9	3UTR
hsa-miR-11400	XM_011523738	RAP1GAP2	5438	5458	1	1	0.00	0.00	20	12	3UTR
hsa-miR-11400	NM_015085	RAP1GAP2	5373	5393	1	1	0.41	-0.18	20	12	3UTR
hsa-miR-11400	NM_001100398	RAP1GAP2	5328	5348	1	1	0.41	-0.18	20	12	3UTR
hsa-miR-11400	NM_021033	RAP2A	955	973	1	1	1.04	0.91	18	10	3UTR
hsa-miR-11400	NM_006105	RAPGEF3	4197	4219	1	1	1.79	2.32	22	10	3UTR
hsa-miR-11400	NM_001098531	RAPGEF3	4437	4459	1	1	1.65	1.90	22	10	3UTR
hsa-miR-11400	NM_016340	RAPGEF6	8014	8032	1	1	4.36	3.44	18	17	3UTR
hsa-miR-11400	NM_001164386	RAPGEF6	8038	8056	1	1	4.36	3.44	18	17	3UTR

mirnaid	refseqid	genesymbol	start	end	bindingp	seed	phylopstem	phylopflank	binding_region_length	longest_consecutive_pairings	position
hsa-miR-11400	NM_001164387	RAPGEF6	7738	7756	1	1	4.36	3.44	18	17	3UTR
hsa-miR-11400	NM_001164388	RAPGEF6	7723	7741	1	1	4.36	3.44	18	17	3UTR
hsa-miR-11400	XM_011543527	RASA1	3751	3775	1	1	0.00	0.00	24	10	3UTR
hsa-miR-11400	NM_001206957	RASSF1	1267	1312	0.974359	1	0.00	0.00	17	15	3UTR
hsa-miR-11400	NM_170713	RASSF1	1264	1309	1	1	1.89	3.11	17	15	3UTR
hsa-miR-11400	NM_170714	RASSF1	1386	1431	1	1	4.38	3.87	17	15	3UTR
hsa-miR-11400	NM_007182	RASSF1	1374	1419	1	1	4.50	4.29	17	15	3UTR
hsa-miR-11400	XM_024448822	RASSF8	1527	1554	1	1	0.00	0.00	27	10	3UTR
hsa-miR-11400	NM_005447	RASSF9	2115	2147	1	1	0.30	0.32	22	8	3UTR
hsa-miR-11400	NM_001135255	RBBP4	4877	4895	1	1	0.00	0.00	18	14	3UTR
hsa-miR-11400	NM_001135256	RBBP4	4842	4860	1	1	0.05	0.03	18	14	3UTR
hsa-miR-11400	XM_011522546	RBFOX1	2764	2785	0.980769	1	0.00	0.00	21	10	3UTR
hsa-miR-11400	XM_017023318	RBFOX1	4344	4365	0.980769	1	0.00	0.00	21	10	3UTR
hsa-miR-11400	XM_017023320	RBFOX1	4733	4754	0.980769	1	0.00	0.00	21	10	3UTR
hsa-miR-11400	XM_017023322	RBFOX1	7354	7375	0.980769	1	0.00	0.00	21	10	3UTR
hsa-miR-11400	XM_017023330	RBFOX1	4158	4179	0.980769	1	0.00	0.00	21	10	3UTR
hsa-miR-11400	XM_017023332	RBFOX1	2838	2859	0.980769	1	0.00	0.00	21	10	3UTR

mirnaid	refseqid	genesymbol	start	end	bindingp	seed	phylopstem	phylopflank	binding_region_length	longest_consecutive_pairings	position
hsa-miR-11400	XM_017023333	RBFOX1	2667	2688	0.980769	1	0.00	0.00	21	10	3UTR
hsa-miR-11400	XM_017023336	RBFOX1	2720	2741	0.980769	1	0.00	0.00	21	10	3UTR
hsa-miR-11400	XM_024450315	RBFOX1	4620	4641	0.980769	1	0.00	0.00	21	10	3UTR
hsa-miR-11400	NM_145891	RBFOX1	2767	2788	1	1	2.11	1.50	21	10	3UTR
hsa-miR-11400	NM_145892	RBFOX1	2689	2710	1	1	2.11	1.50	21	10	3UTR
hsa-miR-11400	NM_145893	RBFOX1	2820	2841	1	1	2.11	1.50	21	10	3UTR
hsa-miR-11400	NM_018723	RBFOX1	3642	3663	1	1	2.11	1.50	21	10	3UTR
hsa-miR-11400	NM_001142333	RBFOX1	3561	3582	1	1	2.11	1.50	21	10	3UTR
hsa-miR-11400	NM_001142334	RBFOX1	2767	2788	1	1	2.11	1.50	21	10	3UTR
hsa-miR-11400	NM_152838	RBM12	4155	4184	1	1	1.87	2.84	24	11	3UTR
hsa-miR-11400	NM_006047	RBM12	4187	4216	1	1	3.13	3.09	24	11	3UTR
hsa-miR-11400	NM_001198840	RBM12	4102	4131	1	1	2.30	2.75	24	11	3UTR
hsa-miR-11400	NM_001146699	RBM19	3743	3769	1	1	1.89	1.95	26	7	3UTR
hsa-miR-11400	NM_001308044	RBM23	9025	9042	1	1	4.02	3.78	17	9	3UTR
hsa-miR-11400	NM_018107	RBM23	9138	9155	1	1	4.77	3.98	17	9	3UTR
hsa-miR-11400	NM_001077351	RBM23	9186	9203	1	1	3.49	4.02	17	9	3UTR
hsa-miR-11400	NM_001077352	RBM23	9084	9101	1	1	3.60	4.11	17	9	3UTR

mirnaid	refseqid	genesymbol	start	end	bindingp	seed	phylopstem	phylopflank	binding_region_length	longest_consecutive_pairings	position
hsa-miR-11400	NM_153020	RBM24	1025	1043	1	1	3.42	2.55	18	8	3UTR
hsa-miR-11400	NM_001143941	RBM24	1077	1095	1	1	3.42	2.55	18	8	3UTR
hsa-miR-11400	XM_011537044	RBM25	3083	3104	1	1	0.00	0.00	21	10	3UTR
hsa-miR-11400	NM_021239	RBM25	2960	2981	1	1	3.70	3.19	21	10	3UTR
hsa-miR-11400	NM_032120	RBM48	3811	3831	1	1	0.21	-0.16	20	16	3UTR
hsa-miR-11400	XM_024453290	RBM5	5515	5535	1	1	0.00	0.00	20	9	3UTR
hsa-miR-11400	XM_017019778	RBMS2	7267	7285	1	1	0.00	0.00	18	17	3UTR
hsa-miR-11400	XM_017019778	RBMS2	1052	1071	1	1	0.00	0.00	19	10	3UTR
hsa-miR-11400	NM_002898	RBMS2	7964	7982	1	1	-0.33	-0.21	18	17	3UTR
hsa-miR-11400	NM_002898	RBMS2	1749	1768	1	1	-0.13	0.27	19	10	3UTR
hsa-miR-11400	NM_014248	RBX1	919	936	1	1	0.16	0.10	17	10	3UTR
hsa-miR-11400	NM_001100588	RC3H2	7372	7388	1	1	4.10	5.53	16	15	3UTR
hsa-miR-11400	NM_001285389	RCAN1	638	663	1	1	0.75	0.73	18	16	3UTR
hsa-miR-11400	NM_001285393	RCAN1	692	717	1	1	0.57	0.62	18	16	3UTR
hsa-miR-11400	NM_203418	RCAN1	741	766	1	1	0.33	0.21	18	16	3UTR
hsa-miR-11400	XM_017028283	RCAN1	1290	1315	1	1	0.00	0.00	18	16	3UTR
hsa-miR-11400	NM_004414	RCAN1	943	960	1	1	-0.17	0.10	17	16	3UTR

mirnaid	refseqid	genesymbol	start	end	bindingp	seed	phylopstem	phylopflank	binding_region_length	longest_consecutive_pairings	position
hsa-miR-11400	NM_001269	RCC1	1825	1874	1	1	0.25	0.48	19	10	3UTR
hsa-miR-11400	NM_001381866	RCC1	2032	2081	1	1	0.25	0.48	19	10	3UTR
hsa-miR-11400	NM_001048194	RCC1	1918	1967	1	1	0.25	0.48	19	10	3UTR
hsa-miR-11400	NM_001048195	RCC1	1876	1925	1	1	0.25	0.48	19	10	3UTR
hsa-miR-11400	NM_001048199	RCC1	1893	1942	1	1	0.25	0.48	19	10	3UTR
hsa-miR-11400	NM_001260494	RDX	2337	2356	1	1	3.37	2.81	19	9	3UTR
hsa-miR-11400	NM_001003716	RECQL5	2040	2057	0.980769	1	-0.72	-0.09	17	13	3UTR
hsa-miR-11400	NM_004259	RECQL5	3549	3567	1	1	0.36	0.56	18	7	3UTR
hsa-miR-11400	NM_001003716	RECQL5	3495	3554	1	1	1.05	2.00	51	10	3UTR
hsa-miR-11400	NM_005669	REEP5	2887	2909	1	1	2.25	2.63	22	9	3UTR
hsa-miR-11400	NM_001130029	RELL2	1830	1850	1	1	1.14	0.71	20	9	3UTR
hsa-miR-11400	NM_001278792	RFC2	1495	1520	1	1	0.32	0.93	25	10	3UTR
hsa-miR-11400	NM_181471	RFC2	1602	1627	1	1	0.32	0.93	25	10	3UTR
hsa-miR-11400	NM_002914	RFC2	1500	1525	1	1	0.32	0.93	25	10	3UTR
hsa-miR-11400	NM_001017368	RFFL	5548	5568	1	1	0.48	0.59	20	12	3UTR
hsa-miR-11400	NM_182705	RFLNB	821	850	1	1	0.17	-0.05	29	9	3UTR
hsa-miR-11400	NM_144629	RFTN2	4687	4705	1	1	0.56	0.93	18	7	3UTR

mirnaid	refseqid	genesymbol	start	end	bindingp	seed	phylopstem	phylopflank	binding_region_length	longest_consecutive_pairings	position
hsa-miR-11400	XM_017022506	RFX7	7906	7925	1	1	0.00	0.00	19	10	3UTR
hsa-miR-11400	NM_001368073	RFX7	5492	5511	1	1	1.65	1.75	19	10	3UTR
hsa-miR-11400	NM_001166287	RGMA	9199	9219	1	1	0.00	0.00	20	11	3UTR
hsa-miR-11400	NM_001286485	RGS11	1533	1562	1	1	3.27	2.93	15	13	3UTR
hsa-miR-11400	NM_003834	RGS11	1577	1606	1	1	2.83	3.42	15	13	3UTR
hsa-miR-11400	NM_012419	RGS17	4708	4733	1	1	1.93	2.60	25	12	3UTR
hsa-miR-11400	NM_012419	RGS17	2280	2298	1	1	0.36	0.23	18	8	3UTR
hsa-miR-11400	XM_011541891	RHCE	2341	2358	1	1	0.00	0.00	17	9	3UTR
hsa-miR-11400	XM_011541891	RHCE	1963	1980	1	1	0.00	0.00	17	10	3UTR
hsa-miR-11400	NM_004040	RHOB	2158	2178	1	1	2.34	0.95	20	8	3UTR
hsa-miR-11400	NM_021205	RHOU	3502	3522	1	1	0.21	0.27	20	8	3UTR
hsa-miR-11400	XM_017018287	RIC3	6697	6719	1	1	0.00	0.00	22	12	3UTR
hsa-miR-11400	NM_024557	RIC3	3190	3210	1	1	0.13	0.04	20	7	3UTR
hsa-miR-11400	NM_001135109	RIC3	2625	2667	1	1	0.31	0.02	21	7	3UTR
hsa-miR-11400	NM_001206671	RIC3	4006	4023	1	1	2.23	2.16	17	12	3UTR
hsa-miR-11400	NM_001206672	RIC3	2950	2970	1	1	0.00	0.00	20	7	3UTR
hsa-miR-11400	NM_173642	RIMKLA	7782	7804	1	1	0.93	0.17	22	15	3UTR

mirnaid	refseqid	genesymbol	start	end	bindingp	seed	phylopstem	phylopflank	binding_region_length	longest_consecutive_pairings	position
hsa-miR-11400	NM_173642	RIMKLA	1563	1585	1	1	0.03	0.43	22	8	3UTR
hsa-miR-11400	NM_001363559	RIN1	4186	4209	1	1	1.21	0.42	23	9	3UTR
hsa-miR-11400	NM_018343	RIOK2	2469	2494	1	1	2.19	1.68	25	9	3UTR
hsa-miR-11400	NM_001286445	RIPOR2	4094	4119	0.961538	1	4.58	4.29	25	8	3UTR
hsa-miR-11400	NM_014722	RIPOR2	4352	4377	0.961538	1	4.63	4.37	25	8	3UTR
hsa-miR-11400	NM_001346032	RIPOR2	4158	4183	0.961538	1	3.15	5.07	25	8	3UTR
hsa-miR-11400	NM_001286446	RIPOR2	3182	3203	1	1	3.27	3.23	21	9	3UTR
hsa-miR-11400	NM_001290268	RIPOR3	3545	3561	1	1	5.68	3.32	16	8	3UTR
hsa-miR-11400	NM_080829	RIPOR3	3341	3357	1	1	3.87	4.10	16	8	3UTR
hsa-miR-11400	XM_005253972	RITA1	1587	1603	1	1	0.00	0.00	16	9	3UTR
hsa-miR-11400	NM_001288794	RMND5B	2087	2126	1	1	0.00	0.00	23	9	3UTR
hsa-miR-11400	NM_001288795	RMND5B	1936	1975	1	1	0.00	0.00	23	9	3UTR
hsa-miR-11400	NM_001386206	RNASE10	1928	1948	1	1	0.00	0.00	20	7	3UTR
hsa-miR-11400	NM_001012975	RNASE10	1558	1578	1	1	0.00	0.00	20	7	3UTR
hsa-miR-11400	NM_021133	RNASEL	2584	2600	1	1	0.45	0.30	16	11	3UTR
hsa-miR-11400	NM_003730	RNASET2	6539	6564	1	1	-0.24	-0.36	25	8	3UTR
hsa-miR-11400	NM_001254738	RND3	1089	1103	1	1	1.40	1.43	14	10	3UTR

mirnaid	refseqid	genesymbol	start	end	bindingp	seed	phylopstem	phylopflank	binding_region_length	longest_consecutive_pairings	position
hsa-miR-11400	NM_005168	RND3	968	982	1	1	1.42	1.62	14	10	3UTR
hsa-miR-11400	NM_152267	RNF185	1524	1555	1	1	0.06	-0.03	22	10	3UTR
hsa-miR-11400	NM_001135825	RNF185	1356	1387	1	1	0.06	-0.03	22	10	3UTR
hsa-miR-11400	NM_001358699	RNF227	1528	1570	1	1	-0.27	0.03	18	10	3UTR
hsa-miR-11400	NM_001042370	RO60	1988	2008	1	1	0.84	1.40	20	16	3UTR
hsa-miR-11400	NM_001173525	RO60	1999	2019	1	1	0.84	0.79	20	16	3UTR
hsa-miR-11400	NM_001128929	ROBO2	7206	7234	1	1	0.87	0.36	28	9	3UTR
hsa-miR-11400	NM_001301088	ROBO4	4012	4033	1	1	1.24	0.51	21	9	3UTR
hsa-miR-11400	NM_019055	ROBO4	4218	4239	1	1	1.24	0.51	21	9	3UTR
hsa-miR-11400	NM_024813	RPAP2	3377	3395	1	1	0.21	0.04	18	8	3UTR
hsa-miR-11400	NM_003973	RPL14	2082	2119	1	1	-0.15	-0.19	17	15	3UTR
hsa-miR-11400	NM_001034996	RPL14	2193	2230	1	1	-0.15	-0.19	17	15	3UTR
hsa-miR-11400	NM_000983	RPL22	1514	1552	1	1	0.30	0.42	19	11	3UTR
hsa-miR-11400	NM_001098577	RPL31	983	1005	1	1	0.16	0.27	22	8	3UTR
hsa-miR-11400	XM_005260480	RPRD1B	1997	2017	1	1	0.00	0.00	20	12	3UTR
hsa-miR-11400	NM_021215	RPRD1B	1850	1870	1	1	0.55	0.52	20	12	3UTR
hsa-miR-11400	NM_001030009	RPS15A	2044	2062	0.961538	1	5.43	5.66	18	9	3UTR

mirnaid	refseqid	genesymbol	start	end	bindingp	seed	phylopstem	phylopflank	binding_region_length	longest_consecutive_pairings	position
hsa-miR-11400	NM_033022	RPS24	442	456	1	1	1.87	2.68	14	13	3UTR
hsa-miR-11400	NM_001142285	RPS24	1333	1352	1	1	-0.16	-0.22	19	15	3UTR
hsa-miR-11400	NM_004755	RPS6KA5	18022	18043	1	1	-0.52	-0.12	21	9	3UTR
hsa-miR-11400	NM_016052	RRP15	6064	6091	1	1	0.93	1.11	27	8	3UTR
hsa-miR-11400	NM_080657	RSAD2	3227	3248	1	1	-0.29	-0.08	21	10	3UTR
hsa-miR-11400	XM_017001518	RSBN1	8386	8422	1	1	0.00	0.00	36	12	3UTR
hsa-miR-11400	NM_198467	RSBN1L	3797	3815	1	1	0.15	0.44	18	9	3UTR
hsa-miR-11400	NM_001193341	RSPH9	1278	1301	1	1	0.00	0.00	23	8	3UTR
hsa-miR-11400	NM_001282863	RSPO2	1602	1624	1	1	0.66	1.25	22	10	3UTR
hsa-miR-11400	NM_001317942	RSPO2	915	946	1	1	0.17	0.35	23	10	3UTR
hsa-miR-11400	NM_178565	RSPO2	1512	1543	1	1	0.27	0.23	23	10	3UTR
hsa-miR-11400	NM_032784	RSPO3	2780	2816	1	1	1.40	0.94	18	16	3UTR
hsa-miR-11400	NM_032784	RSPO3	2886	2909	1	1	0.03	0.40	23	12	3UTR
hsa-miR-11400	NM_023012	RSRC2	3387	3408	1	1	1.28	1.65	21	8	3UTR
hsa-miR-11400	NM_001130841	RTCA	2373	2395	1	1	0.06	0.02	22	9	3UTR
hsa-miR-11400	NM_001024455	RTL5	3159	3182	1	1	2.20	1.47	23	7	3UTR
hsa-miR-11400	NM_001004312	RTP2	1150	1166	1	1	0.69	0.30	16	13	3UTR

mirnaid	refseqid	genesymbol	start	end	bindingp	seed	phylopstem	phylopflank	binding_region_length	longest_consecutive_pairings	position
hsa-miR-11400	NM_025113	RUBCNL	9735	9755	1	1	0.33	0.79	20	8	3UTR
hsa-miR-11400	NM_001330103	RUFY2	2140	2158	1	1	0.46	0.35	18	12	3UTR
hsa-miR-11400	XM_011529766	RUNX1	5860	5898	1	1	0.00	0.00	25	13	3UTR
hsa-miR-11400	NM_001001890	RUNX1	7065	7103	1	1	0.05	0.66	25	13	3UTR
hsa-miR-11400	NM_175634	RUNX1T1	6978	7000	0.961538	1	6.03	4.85	22	8	3UTR
hsa-miR-11400	NM_175635	RUNX1T1	6479	6501	0.961538	1	5.57	6.81	22	8	3UTR
hsa-miR-11400	NM_175636	RUNX1T1	6593	6615	0.961538	1	6.84	6.14	22	8	3UTR
hsa-miR-11400	NM_004349	RUNX1T1	6365	6387	0.961538	1	5.89	6.70	22	8	3UTR
hsa-miR-11400	NM_001198625	RUNX1T1	6867	6889	0.961538	1	5.40	5.48	22	8	3UTR
hsa-miR-11400	NM_001198626	RUNX1T1	6725	6747	0.961538	1	5.64	6.04	22	8	3UTR
hsa-miR-11400	NM_001198627	RUNX1T1	6785	6807	0.961538	1	5.63	5.86	22	8	3UTR
hsa-miR-11400	NM_001198628	RUNX1T1	6558	6580	0.961538	1	6.63	6.63	22	8	3UTR
hsa-miR-11400	NM_001198631	RUNX1T1	6581	6603	0.961538	1	7.25	6.35	22	8	3UTR
hsa-miR-11400	NM_001198632	RUNX1T1	6643	6665	0.961538	1	5.51	6.36	22	8	3UTR
hsa-miR-11400	NM_001198633	RUNX1T1	6805	6827	0.961538	1	5.59	5.50	22	8	3UTR
hsa-miR-11400	NM_001198634	RUNX1T1	6408	6430	0.961538	1	6.79	6.40	22	8	3UTR
hsa-miR-11400	NM_001198679	RUNX1T1	6552	6574	0.961538	1	6.83	6.57	22	8	3UTR

mirnaid	refseqid	genesymbol	start	end	bindingp	seed	phylopstem	phylopflank	binding_region_length	longest_consecutive_pairings	position
hsa-miR-11400	NM_001024212	S100A13	467	509	1	1	-0.54	-0.11	23	6	3UTR
hsa-miR-11400	NM_176823	S100A7A	1192	1214	1	1	-0.48	-0.77	22	12	3UTR
hsa-miR-11400	NM_199161	SAA1	437	474	1	1	0.09	0.26	22	11	3UTR
hsa-miR-11400	NM_000331	SAA1	584	621	1	1	2.24	1.10	22	11	3UTR
hsa-miR-11400	NM_001178006	SAA1	498	535	1	1	2.24	1.10	22	11	3UTR
hsa-miR-11400	NM_005500	SAE1	1611	1633	1	1	-0.39	0.08	22	8	3UTR
hsa-miR-11400	NM_001145713	SAE1	1396	1418	1	1	-0.39	0.08	22	8	3UTR
hsa-miR-11400	NM_001145714	SAE1	1466	1488	1	1	-0.39	0.08	22	8	3UTR
hsa-miR-11400	NM_001349811	SAMD12	7829	7845	1	1	0.70	2.94	16	7	3UTR
hsa-miR-11400	NM_001349811	SAMD12	2933	2975	1	1	0.32	0.09	18	10	3UTR
hsa-miR-11400	NM_001101676	SAMD12	7859	7875	1	1	3.53	2.65	16	7	3UTR
hsa-miR-11400	NM_001101676	SAMD12	2963	3005	1	1	0.28	0.00	18	10	3UTR
hsa-miR-11400	NM_174920	SAMD14	2579	2597	1	1	-0.36	0.45	18	9	3UTR
hsa-miR-11400	XM_011524490	SAMD14	1404	1422	1	1	0.00	0.00	18	9	3UTR
hsa-miR-11400	XM_017024322	SAMD14	2601	2619	1	1	0.00	0.00	18	9	3UTR
hsa-miR-11400	NM_001304366	SAMD7	2246	2267	1	1	-0.43	-0.11	21	8	3UTR
hsa-miR-11400	NM_182610	SAMD7	2269	2290	1	1	-0.43	-0.11	21	8	3UTR

mirnaid	refseqid	genesymbol	start	end	bindingp	seed	phylopstem	phylopflank	binding_region_length	longest_consecutive_pairings	position
hsa-miR-11400	NM_144660	SAMD8	4665	4691	1	1	0.59	0.34	26	9	3UTR
hsa-miR-11400	NM_001174156	SAMD8	4438	4464	1	1	0.59	0.34	26	9	3UTR
hsa-miR-11400	NM_001363733	SAMHD1	2300	2319	1	1	1.23	4.92	19	7	3UTR
hsa-miR-11400	NM_016103	SAR1B	3524	3546	1	1	0.13	0.29	22	13	3UTR
hsa-miR-11400	NM_001033503	SAR1B	3643	3665	1	1	0.13	0.33	22	13	3UTR
hsa-miR-11400	XM_011537057	SAV1	2344	2365	1	1	0.00	0.00	21	8	3UTR
hsa-miR-11400	NM_006918	SC5D	5399	5418	1	1	-0.23	-0.04	19	7	3UTR
hsa-miR-11400	NM_001024956	SC5D	5580	5599	1	1	0.22	0.02	19	7	3UTR
hsa-miR-11400	NM_004719	SCAF11	5609	5634	1	1	-0.09	0.13	25	8	3UTR
hsa-miR-11400	NM_138967	SCAMP5	1665	1691	1	1	0.05	0.19	26	8	3UTR
hsa-miR-11400	NM_138967	SCAMP5	2138	2178	1	1	0.23	0.48	40	10	3UTR
hsa-miR-11400	NM_001178111	SCAMP5	1748	1774	1	1	0.05	0.19	26	8	3UTR
hsa-miR-11400	NM_001178111	SCAMP5	2221	2261	1	1	0.23	0.48	40	10	3UTR
hsa-miR-11400	NM_005506	SCARB2	2716	2748	1	1	1.08	0.61	32	8	3UTR
hsa-miR-11400	NM_001204255	SCARB2	2287	2319	1	1	0.00	0.00	32	8	3UTR
hsa-miR-11400	NM_001025591	SCGB2B2	1567	1587	1	1	0.04	-0.05	20	10	3UTR
hsa-miR-11400	NM_006746	SCML1	2123	2163	1	1	1.14	0.51	21	10	3UTR

mirnaid	refseqid	genesymbol	start	end	bindingp	seed	phylopstem	phylopflank	binding_region_length	longest_consecutive_pairings	position
hsa-miR-11400	NM_001037535	SCML1	1974	2014	1	1	0.72	0.46	21	10	3UTR
hsa-miR-11400	NM_001037536	SCML1	1971	2011	1	1	1.14	0.51	21	10	3UTR
hsa-miR-11400	NM_001037540	SCML1	2204	2244	1	1	1.14	0.51	21	10	3UTR
hsa-miR-11400	NM_001286408	SCML4	2782	2800	1	1	1.04	0.89	18	9	3UTR
hsa-miR-11400	NM_198081	SCML4	2997	3015	1	1	0.35	0.79	18	9	3UTR
hsa-miR-11400	NM_021007	SCN2A	6987	7030	1	1	0.77	1.82	43	11	3UTR
hsa-miR-11400	NM_001371246	SCN2A	7133	7176	1	1	0.77	1.82	43	11	3UTR
hsa-miR-11400	NM_001371247	SCN2A	6961	7004	1	1	0.77	1.82	43	11	3UTR
hsa-miR-11400	NM_001040142	SCN2A	7133	7176	1	1	0.77	1.82	43	11	3UTR
hsa-miR-11400	NM_001040143	SCN2A	7252	7295	1	1	0.77	1.82	43	11	3UTR
hsa-miR-11400	NM_004588	SCN2B	2965	2990	1	1	0.26	-0.22	25	11	3UTR
hsa-miR-11400	NM_174934	SCN4B	4009	4029	1	1	0.24	1.94	20	8	3UTR
hsa-miR-11400	NM_001142348	SCN4B	3607	3627	1	1	0.49	1.65	20	8	3UTR
hsa-miR-11400	NM_024041	SCNM1	831	847	1	1	0.64	0.30	16	15	3UTR
hsa-miR-11400	NM_001204856	SCNM1	880	896	1	1	0.79	0.19	16	15	3UTR
hsa-miR-11400	NM_032547	SCOC	3709	3728	1	1	0.17	-0.02	19	10	3UTR
hsa-miR-11400	NM_001153484	SCOC	3590	3609	1	1	0.17	-0.02	19	10	3UTR

mirnaid	refseqid	genesymbol	start	end	bindingp	seed	phylopstem	phylopflank	binding_region_length	longest_consecutive_pairings	position
hsa-miR-11400	NM_001278172	SDHC	9224	9254	1	1	0.01	0.00	30	13	3UTR
hsa-miR-11400	NM_001035511	SDHC	9326	9356	1	1	0.01	0.00	30	13	3UTR
hsa-miR-11400	NM_001035512	SDHC	9388	9418	1	1	0.01	0.00	30	13	3UTR
hsa-miR-11400	NM_001035513	SDHC	9331	9361	1	1	0.01	0.00	30	13	3UTR
hsa-miR-11400	NM_001318049	SDR16C5	1760	1783	1	1	0.13	-0.13	17	10	3UTR
hsa-miR-11400	NM_001318050	SDR16C5	1481	1504	1	1	-0.66	-0.65	17	10	3UTR
hsa-miR-11400	NM_138969	SDR16C5	1613	1636	1	1	-0.06	-0.04	17	10	3UTR
hsa-miR-11400	XM_024453705	SEC13	1092	1128	1	1	0.00	0.00	36	12	3UTR
hsa-miR-11400	NM_001291932	SEC14L2	1961	1979	1	1	0.17	0.23	18	17	3UTR
hsa-miR-11400	NM_012429	SEC14L2	2037	2055	1	1	0.17	0.23	18	17	3UTR
hsa-miR-11400	NM_001204204	SEC14L2	1788	1806	1	1	1.13	0.98	18	17	3UTR
hsa-miR-11400	NM_001193336	SEC14L6	1300	1320	1	1	0.04	0.14	20	11	3UTR
hsa-miR-11400	NM_032970	SEC22C	5609	5627	1	1	4.54	3.80	18	9	3UTR
hsa-miR-11400	XM_024453816	SEC22C	5733	5751	1	1	0.00	0.00	18	9	3UTR
hsa-miR-11400	NM_015490	SEC31B	4355	4373	1	1	4.05	4.06	18	7	3UTR
hsa-miR-11400	NM_007214	SEC63	2804	2823	1	1	-0.05	0.17	19	9	3UTR
hsa-miR-11400	NM_000450	SELE	2379	2400	1	1	0.62	0.91	21	8	3UTR

mirnaid	refseqid	genesymbol	start	end	bindingp	seed	phylopstem	phylopflank	binding_region_length	longest_consecutive_pairings	position
hsa-miR-11400	NM_033505	SELENOI	3550	3596	1	1	-0.04	-0.10	20	10	3UTR
hsa-miR-11400	NM_020163	SEMA3G	3443	3465	1	1	2.91	1.88	22	12	3UTR
hsa-miR-11400	NM_001271662	SEMA4F	2655	2672	1	1	0.23	0.07	17	16	3UTR
hsa-miR-11400	XM_011514156	SEMA5A	5901	5919	1	1	0.00	0.00	18	10	3UTR
hsa-miR-11400	NM_001267594	SENP1	3093	3111	1	1	4.63	2.47	18	10	3UTR
hsa-miR-11400	NM_001267595	SENP1	3440	3458	1	1	1.17	2.18	18	10	3UTR
hsa-miR-11400	NM_001308045	SENP5	5910	5932	1	1	0.62	0.82	22	7	3UTR
hsa-miR-11400	NM_001308045	SENP5	3444	3461	1	1	0.00	-0.47	17	9	3UTR
hsa-miR-11400	NM_152699	SENP5	6048	6070	1	1	1.03	0.95	22	7	3UTR
hsa-miR-11400	NM_001166340	SENP8	3834	3859	1	1	0.00	0.00	25	8	3UTR
hsa-miR-11400	NM_001293695	SEPTIN9	2975	2996	1	1	-0.60	-0.86	21	15	3UTR
hsa-miR-11400	NM_001293696	SEPTIN9	2338	2359	1	1	-0.60	-0.86	21	15	3UTR
hsa-miR-11400	NM_001293697	SEPTIN9	2319	2340	1	1	-0.60	-0.86	21	15	3UTR
hsa-miR-11400	NM_001293698	SEPTIN9	2611	2632	1	1	-0.60	-0.86	21	15	3UTR
hsa-miR-11400	NM_006640	SEPTIN9	3750	3771	1	1	-0.78	-0.79	21	15	3UTR
hsa-miR-11400	NM_001113491	SEPTIN9	3032	3053	1	1	-0.60	-0.86	21	15	3UTR
hsa-miR-11400	NM_001113492	SEPTIN9	3252	3273	1	1	-0.60	-0.68	21	15	3UTR

mirnaid	refseqid	genesymbol	start	end	bindingp	seed	phylopstem	phylopflank	binding_region_length	longest_consecutive_pairings	position
hsa-miR-11400	NM_001113493	SEPTIN9	3135	3156	1	1	-0.60	-0.86	21	15	3UTR
hsa-miR-11400	NM_001113494	SEPTIN9	3235	3256	1	1	-0.60	-0.86	21	15	3UTR
hsa-miR-11400	NM_001113495	SEPTIN9	2543	2564	1	1	-0.60	-0.86	21	15	3UTR
hsa-miR-11400	NM_001113496	SEPTIN9	2312	2333	1	1	-0.60	-0.86	21	15	3UTR
hsa-miR-11400	NM_014445	SERP1	1140	1159	1	1	1.36	0.73	19	8	3UTR
hsa-miR-11400	XM_024449352	SERP2	582	596	1	1	0.00	0.00	14	13	3UTR
hsa-miR-11400	XM_005267733	SERPINA10	1613	1629	1	1	0.00	0.00	16	15	3UTR
hsa-miR-11400	XM_017021353	SERPINA10	1812	1828	1	1	0.00	0.00	16	15	3UTR
hsa-miR-11400	NM_001100607	SERPINA10	1525	1541	1	1	-0.31	0.02	16	15	3UTR
hsa-miR-11400	NM_000602	SERPINE1	2535	2555	1	1	1.74	1.96	20	8	3UTR
hsa-miR-11400	NM_013376	SERTAD1	926	943	1	1	0.13	0.43	17	11	3UTR
hsa-miR-11400	NM_203344	SERTAD3	1073	1094	1	1	1.90	1.88	21	12	3UTR
hsa-miR-11400	NM_001271594	SESN3	4072	4089	1	1	0.09	0.38	17	9	3UTR
hsa-miR-11400	NM_001271594	SESN3	4826	4846	1	1	0.64	0.63	20	7	3UTR
hsa-miR-11400	NM_144665	SESN3	4356	4373	1	1	0.86	0.40	17	9	3UTR
hsa-miR-11400	NM_001286752	SETD4	2276	2294	1	1	3.67	1.95	18	8	3UTR
hsa-miR-11400	NM_017438	SETD4	2009	2027	1	1	1.62	2.61	18	8	3UTR

mirnaid	refseqid	genesymbol	start	end	bindingp	seed	phylopstem	phylopflank	binding_region_length	longest_consecutive_pairings	position
hsa-miR-11400	NM_001160305	SETD6	3069	3090	1	1	2.38	2.44	21	7	3UTR
hsa-miR-11400	NM_005877	SF3A1	3441	3468	1	1	6.22	6.17	27	10	3UTR
hsa-miR-11400	NM_005877	SF3A1	3568	3590	1	1	5.67	5.42	22	7	3UTR
hsa-miR-11400	XM_011519611	SFMBT2	6523	6549	1	1	0.00	0.00	26	9	3UTR
hsa-miR-11400	NM_001029880	SFMBT2	6455	6472	1	1	3.26	4.46	17	8	3UTR
hsa-miR-11400	NM_001029880	SFMBT2	6528	6554	1	1	2.44	3.50	26	9	3UTR
hsa-miR-11400	NM_003012	SFRP1	3629	3656	1	1	3.86	4.19	27	9	3UTR
hsa-miR-11400	NM_178858	SFXN2	2256	2289	1	1	0.44	0.16	20	10	3UTR
hsa-miR-11400	NM_000337	SGCD	4582	4626	1	1	-0.08	-0.08	19	8	3UTR
hsa-miR-11400	NM_001128209	SGCD	4536	4580	1	1	-0.08	-0.08	19	8	3UTR
hsa-miR-11400	NM_001308294	SH2B1	1384	1415	1	1	2.25	1.87	31	13	3UTR
hsa-miR-11400	NM_001145797	SH2B1	2502	2533	1	1	2.25	1.87	31	13	3UTR
hsa-miR-11400	NM_001174160	SH2D4A	2313	2330	1	1	-0.04	-0.12	17	10	3UTR
hsa-miR-11400	NM_001145719	SH2D4B	1982	2021	1	1	-0.17	-0.11	39	11	3UTR
hsa-miR-11400	NM_031469	SH3BGRL2	472	497	1	1	1.41	2.11	25	12	3UTR
hsa-miR-11400	NM_001199944	SH3GL1	2226	2250	1	1	0.31	0.80	24	11	3UTR
hsa-miR-11400	NM_014631	SH3PXD2A	4920	4937	1	1	0.00	0.84	17	9	3UTR

mirnaid	refseqid	genesymbol	start	end	bindingp	seed	phylopstem	phylopflank	binding_region_length	longest_consecutive_pairings	position
hsa-miR-11400	NM_024577	SH3TC2	23930	23960	1	1	1.42	1.89	30	16	3UTR
hsa-miR-11400	NM_024577	SH3TC2	5901	5918	1	1	0.12	0.06	17	11	3UTR
hsa-miR-11400	NM_016848	SHC3	8323	8369	1	1	2.71	1.90	46	10	3UTR
hsa-miR-11400	NM_198149	SHISA4	930	949	1	1	0.16	0.34	19	13	3UTR
hsa-miR-11400	NM_001164442	SHISAL2B	707	728	1	1	0.27	0.24	21	9	3UTR
hsa-miR-11400	NM_198849	SIAH3	2618	2636	1	1	-0.28	-0.17	18	15	3UTR
hsa-miR-11400	NM_015191	SIK2	3604	3629	1	1	0.24	0.13	25	8	3UTR
hsa-miR-11400	NM_001102396	SIKE1	4053	4093	1	1	0.43	0.18	18	16	3UTR
hsa-miR-11400	XM_005273213	SIPA1L2	5630	5669	0.974359	1	0.00	0.00	19	10	3UTR
hsa-miR-11400	XM_017001896	SIPA1L2	5683	5722	0.974359	1	0.00	0.00	19	10	3UTR
hsa-miR-11400	XM_017001897	SIPA1L2	5897	5915	0.974359	1	0.00	0.00	18	10	3UTR
hsa-miR-11400	NM_020808	SIPA1L2	5928	5946	1	1	3.22	2.38	18	10	3UTR
hsa-miR-11400	NM_170679	SKP1	8019	8035	0.953846	1	1.66	0.84	16	7	3UTR
hsa-miR-11400	NM_001045557	SLA	1828	1846	1	1	2.02	1.32	18	9	3UTR
hsa-miR-11400	NM_032214	SLA2	1296	1320	1	1	0.21	0.00	24	15	3UTR
hsa-miR-11400	NM_032214	SLA2	2296	2314	1	1	0.02	0.75	18	7	3UTR
hsa-miR-11400	NM_175077	SLA2	1246	1270	1	1	-0.02	0.00	24	15	3UTR

mirnaid	refseqid	genesymbol	start	end	bindingp	seed	phylopstem	phylopflank	binding_region_length	longest_consecutive_pairings	position
hsa-miR-11400	NM_175077	SLA2	2246	2264	1	1	0.02	0.75	18	7	3UTR
hsa-miR-11400	NM_003037	SLAMF1	2889	2912	1	1	-0.63	0.47	17	12	3UTR
hsa-miR-11400	NM_001330754	SLAMF1	2712	2728	1	1	0.99	1.26	16	12	3UTR
hsa-miR-11400	NM_001282588	SLAMF7	1294	1316	1	1	0.01	-0.11	22	10	3UTR
hsa-miR-11400	NM_001282588	SLAMF7	1480	1499	1	1	-0.15	-0.02	19	15	3UTR
hsa-miR-11400	NM_001282589	SLAMF7	1398	1420	1	1	0.01	-0.11	22	10	3UTR
hsa-miR-11400	NM_001282589	SLAMF7	1584	1603	1	1	-0.15	-0.02	19	15	3UTR
hsa-miR-11400	NM_001282590	SLAMF7	1470	1492	1	1	-0.26	-0.11	22	10	3UTR
hsa-miR-11400	NM_001282590	SLAMF7	1656	1675	1	1	-0.44	-0.12	19	15	3UTR
hsa-miR-11400	NM_001282591	SLAMF7	1350	1372	1	1	0.01	-0.11	22	10	3UTR
hsa-miR-11400	NM_001282591	SLAMF7	1536	1555	1	1	-0.15	-0.02	19	15	3UTR
hsa-miR-11400	NM_001282592	SLAMF7	1687	1709	1	1	-0.26	-0.11	22	10	3UTR
hsa-miR-11400	NM_001282592	SLAMF7	1873	1892	1	1	-0.44	-0.12	19	15	3UTR
hsa-miR-11400	NM_001282593	SLAMF7	1246	1268	1	1	0.01	-0.11	22	10	3UTR
hsa-miR-11400	NM_001282593	SLAMF7	1432	1451	1	1	-0.15	-0.02	19	15	3UTR
hsa-miR-11400	NM_001282594	SLAMF7	1509	1531	1	1	0.01	-0.11	22	10	3UTR
hsa-miR-11400	NM_001282594	SLAMF7	1695	1714	1	1	-0.15	-0.02	19	15	3UTR

mirnaid	refseqid	genesymbol	start	end	bindingp	seed	phylopstem	phylopflank	binding_region_length	longest_consecutive_pairings	position
hsa-miR-11400	NM_021181	SLAMF7	1791	1813	1	1	-0.26	-0.11	22	10	3UTR
hsa-miR-11400	NM_021181	SLAMF7	1977	1996	1	1	-0.44	-0.12	19	15	3UTR
hsa-miR-11400	NM_001300842	SLC10A7	1796	1846	1	1	0.60	0.60	20	13	3UTR
hsa-miR-11400	NM_001317816	SLC10A7	1646	1696	1	1	0.89	0.24	20	13	3UTR
hsa-miR-11400	NM_001029998	SLC10A7	1685	1735	1	1	0.10	0.29	20	13	3UTR
hsa-miR-11400	NM_001284510	SLC13A5	2568	2590	1	1	1.08	1.99	22	9	3UTR
hsa-miR-11400	NM_177550	SLC13A5	2697	2719	1	1	0.37	2.33	22	9	3UTR
hsa-miR-11400	NM_001143838	SLC13A5	2559	2581	1	1	0.37	2.33	22	9	3UTR
hsa-miR-11400	NM_001308278	SLC14A1	1668	1697	1	1	-0.77	-0.20	20	18	3UTR
hsa-miR-11400	NM_001308279	SLC14A1	1478	1507	1	1	-0.77	-0.20	20	18	3UTR
hsa-miR-11400	XM_005258333	SLC14A1	1442	1471	1	1	0.00	0.00	20	18	3UTR
hsa-miR-11400	NM_015865	SLC14A1	1840	1869	1	1	-0.77	-0.20	20	18	3UTR
hsa-miR-11400	NM_001128588	SLC14A1	1998	2027	1	1	-0.77	-0.20	20	18	3UTR
hsa-miR-11400	NM_001146036	SLC14A1	1927	1956	1	1	-0.77	-0.20	20	18	3UTR
hsa-miR-11400	NM_001146037	SLC14A1	2149	2178	1	1	-0.77	-0.20	20	18	3UTR
hsa-miR-11400	XM_011510752	SLC16A14	2207	2228	1	1	0.00	0.00	21	9	3UTR
hsa-miR-11400	NM_004207	SLC16A3	1519	1537	1	1	0.49	1.51	18	9	3UTR

mirnaid	refseqid	genesymbol	start	end	bindingp	seed	phylopstem	phylopflank	binding_region_length	longest_consecutive_pairings	position
hsa-miR-11400	NM_001042423	SLC16A3	1582	1600	1	1	0.49	1.51	18	9	3UTR
hsa-miR-11400	NM_001206950	SLC16A3	1565	1583	1	1	2.08	2.53	18	9	3UTR
hsa-miR-11400	NM_001206951	SLC16A3	1546	1574	1	1	2.82	1.22	28	10	3UTR
hsa-miR-11400	NM_001206952	SLC16A3	1523	1541	1	1	1.79	1.30	18	9	3UTR
hsa-miR-11400	NM_139319	SLC17A8	3639	3656	1	1	-0.05	0.23	17	12	3UTR
hsa-miR-11400	NM_001145288	SLC17A8	3489	3506	1	1	-0.05	0.23	17	12	3UTR
hsa-miR-11400	NM_001319667	SLC19A2	2380	2431	1	1	2.44	3.16	19	10	3UTR
hsa-miR-11400	NM_001195728	SLC1A2	3087	3109	1	1	0.88	0.90	22	7	3UTR
hsa-miR-11400	NM_001166696	SLC1A3	864	886	1	1	0.35	0.34	16	14	3UTR
hsa-miR-11400	NM_001307985	SLC22A11	2671	2688	1	1	-0.40	-0.15	17	10	3UTR
hsa-miR-11400	NM_018484	SLC22A11	2995	3012	1	1	-0.40	-0.15	17	10	3UTR
hsa-miR-11400	NM_001286455	SLC22A23	1659	1683	1	1	-0.32	-0.29	24	9	3UTR
hsa-miR-11400	NM_015482	SLC22A23	2609	2633	1	1	-0.51	0.00	24	9	3UTR
hsa-miR-11400	NM_020344	SLC24A2	5019	5041	1	1	0.35	-0.14	22	7	3UTR
hsa-miR-11400	NM_020344	SLC24A2	2372	2393	1	1	-0.10	0.07	21	8	3UTR
hsa-miR-11400	NM_001193288	SLC24A2	4968	4990	1	1	0.05	0.15	22	7	3UTR
hsa-miR-11400	XM_024452474	SLC25A14	953	974	1	1	0.00	0.00	21	12	3UTR

mirnaid	refseqid	genesymbol	start	end	bindingp	seed	phylopstem	phylopflank	binding_region_length	longest_consecutive_pairings	position
hsa-miR-11400	NM_001282727	SLC25A17	927	957	1	1	0.73	0.41	19	9	3UTR
hsa-miR-11400	NM_006358	SLC25A17	1146	1176	1	1	-0.09	-0.08	19	9	3UTR
hsa-miR-11400	NM_024698	SLC25A22	1454	1473	0.953846	1	1.14	1.76	19	10	3UTR
hsa-miR-11400	NM_001191060	SLC25A22	1645	1664	0.953846	1	1.23	2.27	19	10	3UTR
hsa-miR-11400	NM_152333	SLC25A29	2173	2195	1	1	-0.32	-0.15	22	9	3UTR
hsa-miR-11400	NM_001352822	SLC25A29	2293	2315	1	1	-0.32	-0.15	22	9	3UTR
hsa-miR-11400	NM_001352823	SLC25A29	2379	2401	1	1	-0.32	-0.15	22	9	3UTR
hsa-miR-11400	NM_001039355	SLC25A29	2217	2239	1	1	-0.32	-0.15	22	9	3UTR
hsa-miR-11400	NM_018155	SLC25A36	1203	1224	1	1	1.63	1.51	21	8	3UTR
hsa-miR-11400	NM_001104647	SLC25A36	1206	1227	1	1	1.63	1.51	21	8	3UTR
hsa-miR-11400	NM_001286184	SLC25A44	1650	1671	1	1	0.23	0.28	21	9	3UTR
hsa-miR-11400	NM_014655	SLC25A44	1626	1647	1	1	0.23	0.28	21	9	3UTR
hsa-miR-11400	NM_001012755	SLC25A53	3404	3421	1	1	2.71	1.12	17	8	3UTR
hsa-miR-11400	NM_001636	SLC25A6	1374	1386	1	1	-0.34	0.28	12	11	3UTR
hsa-miR-11400	NM_145176	SLC2A12	5044	5060	1	1	1.41	2.97	16	15	3UTR
hsa-miR-11400	NM_006931	SLC2A3	2168	2186	1	1	0.36	0.92	18	11	3UTR
hsa-miR-11400	NM_006345	SLC30A9	4906	4921	1	1	-0.02	-0.04	15	12	3UTR

mirnaid	refseqid	genesymbol	start	end	bindingp	seed	phylopstem	phylopflank	binding_region_length	longest_consecutive_pairings	position
hsa-miR-11400	NM_004733	SLC33A1	9003	9018	0.961538	1	0.00	0.24	15	14	3UTR
hsa-miR-11400	NM_001271684	SLC35A3	2842	2861	1	1	0.04	-0.01	19	10	3UTR
hsa-miR-11400	NM_001271685	SLC35A3	3056	3075	1	1	0.04	-0.01	19	10	3UTR
hsa-miR-11400	XM_005270691	SLC35A3	4789	4808	1	1	0.00	0.00	19	10	3UTR
hsa-miR-11400	XM_011541136	SLC35A3	3219	3238	1	1	0.00	0.00	19	10	3UTR
hsa-miR-11400	XM_017000869	SLC35A3	4567	4586	1	1	0.00	0.00	19	10	3UTR
hsa-miR-11400	XM_017000870	SLC35A3	2926	2945	1	1	0.00	0.00	19	10	3UTR
hsa-miR-11400	XM_017000871	SLC35A3	4433	4452	1	1	0.00	0.00	19	10	3UTR
hsa-miR-11400	NM_012243	SLC35A3	3095	3114	1	1	0.04	-0.01	19	10	3UTR
hsa-miR-11400	NM_017945	SLC35A5	3893	3913	1	1	0.04	-0.02	20	11	3UTR
hsa-miR-11400	NM_001348910	SLC35A5	3421	3441	1	1	0.04	-0.02	20	11	3UTR
hsa-miR-11400	NM_001286511	SLC35B2	1864	1879	1	1	1.40	1.09	15	9	3UTR
hsa-miR-11400	NM_001286512	SLC35B2	1849	1864	1	1	1.19	1.89	15	9	3UTR
hsa-miR-11400	NM_001286513	SLC35B2	1716	1731	1	1	2.20	1.24	15	9	3UTR
hsa-miR-11400	NM_001286517	SLC35B2	1677	1692	1	1	4.02	2.27	15	9	3UTR
hsa-miR-11400	NM_001286519	SLC35B2	1522	1537	1	1	5.63	2.40	15	9	3UTR
hsa-miR-11400	NM_178148	SLC35B2	1871	1886	1	1	2.20	1.24	15	9	3UTR

mirnaid	refseqid	genesymbol	start	end	bindingp	seed	phylopstem	phylopflank	binding_region_length	longest_consecutive_pairings	position
hsa-miR-11400	NM_025181	SLC35F5	4930	4948	1	1	-0.39	0.22	18	12	3UTR
hsa-miR-11400	XM_005268386	SLC36A1	3046	3068	1	1	0.00	0.00	22	8	3UTR
hsa-miR-11400	NM_001278387	SLC38A1	4892	4911	1	1	0.23	1.14	19	15	3UTR
hsa-miR-11400	NM_001278388	SLC38A1	4654	4673	1	1	-0.24	0.08	19	15	3UTR
hsa-miR-11400	NM_030674	SLC38A1	4769	4788	1	1	1.74	0.77	19	15	3UTR
hsa-miR-11400	NM_001077484	SLC38A1	4430	4449	1	1	-0.29	-0.18	19	15	3UTR
hsa-miR-11400	NM_015359	SLC39A14	3878	3903	1	1	0.32	-0.17	25	13	3UTR
hsa-miR-11400	NM_001128431	SLC39A14	3878	3903	1	1	0.32	-0.17	25	13	3UTR
hsa-miR-11400	NM_001135153	SLC39A14	3947	3972	1	1	0.00	0.00	25	13	3UTR
hsa-miR-11400	NM_001198810	SLC43A1	1913	1931	1	1	3.15	2.85	18	8	3UTR
hsa-miR-11400	NM_001198810	SLC43A1	2189	2237	1	1	7.78	3.82	16	8	3UTR
hsa-miR-11400	NM_199329	SLC43A3	1914	1943	1	1	3.83	3.10	29	8	3UTR
hsa-miR-11400	NM_017611	SLC43A3	1892	1921	1	1	2.63	3.55	29	8	3UTR
hsa-miR-11400	NM_014096	SLC43A3	2090	2119	1	1	3.04	1.95	29	8	3UTR
hsa-miR-11400	NM_001286730	SLC44A1	9404	9426	1	1	1.46	2.23	22	9	3UTR
hsa-miR-11400	NM_080546	SLC44A1	10275	10297	1	1	1.46	2.23	22	9	3UTR
hsa-miR-11400	NM_001012509	SLC45A2	1869	1917	1	1	0.21	0.64	19	14	3UTR

mirnaid	refseqid	genesymbol	start	end	bindingp	seed	phylopstem	phylopflank	binding_region_length	longest_consecutive_pairings	position
hsa-miR-11400	NM_001012509	SLC45A2	2335	2353	1	1	3.94	3.73	18	12	3UTR
hsa-miR-11400	NM_003759	SLC4A4	3736	3757	1	1	0.64	0.60	21	8	3UTR
hsa-miR-11400	NM_001098484	SLC4A4	3783	3804	1	1	0.64	0.60	21	8	3UTR
hsa-miR-11400	NM_001370086	SLC52A3	2260	2284	1	1	2.45	5.16	18	7	3UTR
hsa-miR-11400	NM_000343	SLC5A1	2201	2219	1	1	-0.33	0.30	18	8	3UTR
hsa-miR-11400	NM_018057	SLC6A15	3852	3873	1	1	0.38	0.37	21	11	3UTR
hsa-miR-11400	NM_001044	SLC6A3	3757	3774	1	1	-0.35	-0.16	17	16	3UTR
hsa-miR-11400	NM_001045	SLC6A4	2323	2339	1	1	-0.25	0.28	16	15	3UTR
hsa-miR-11400	NM_014228	SLC6A7	2290	2307	1	1	-0.57	0.02	17	16	3UTR
hsa-miR-11400	NM_003983	SLC7A6	2318	2339	1	1	0.60	0.39	21	11	3UTR
hsa-miR-11400	NM_001076785	SLC7A6	2405	2426	1	1	0.60	0.39	21	11	3UTR
hsa-miR-11400	NM_032178	SLC7A6OS	3745	3765	1	1	2.79	0.46	20	13	3UTR
hsa-miR-11400	XM_006712083	SLC8A1	4605	4630	0.961538	1	0.00	0.00	25	8	3UTR
hsa-miR-11400	XM_011533054	SLC8A1	4522	4547	0.961538	1	0.00	0.00	25	8	3UTR
hsa-miR-11400	NM_001351494	SLC8A1	4583	4608	1	1	0.33	0.09	25	8	3UTR
hsa-miR-11400	NM_134431	SLCO1A2	5914	5936	1	1	2.89	2.96	22	15	3UTR
hsa-miR-11400	NM_134431	SLCO1A2	3988	4019	1	1	0.12	0.25	31	11	3UTR

mirnaid	refseqid	genesymbol	start	end	bindingp	seed	phylopstem	phylopflank	binding_region_length	longest_consecutive_pairings	position
hsa-miR-11400	XM_011520818	SLCO1A2	5342	5364	1	1	0.00	0.00	22	15	3UTR
hsa-miR-11400	XM_011520818	SLCO1A2	4433	4453	1	1	0.00	0.00	20	8	3UTR
hsa-miR-11400	XM_011520818	SLCO1A2	3416	3447	1	1	0.00	0.00	31	11	3UTR
hsa-miR-11400	NM_030958	SLCO5A1	3271	3313	1	1	0.03	0.17	20	11	3UTR
hsa-miR-11400	NM_001146008	SLCO5A1	3267	3309	1	1	-0.03	0.19	20	11	3UTR
hsa-miR-11400	NM_001146009	SLCO5A1	2981	3023	1	1	0.40	0.48	20	11	3UTR
hsa-miR-11400	NM_152270	SLFN11	4036	4056	1	1	3.22	0.39	20	9	3UTR
hsa-miR-11400	NM_001104590	SLFN11	4143	4163	1	1	0.10	0.52	20	9	3UTR
hsa-miR-11400	NM_144682	SLFN13	4375	4395	1	1	0.05	0.02	20	9	3UTR
hsa-miR-11400	NM_144682	SLFN13	3925	3950	1	1	-0.02	-0.02	25	8	3UTR
hsa-miR-11400	XM_005257922	SLFN13	3977	4002	1	1	0.00	0.00	25	8	3UTR
hsa-miR-11400	XM_005257922	SLFN13	3909	3928	1	1	0.00	0.00	19	10	3UTR
hsa-miR-11400	XM_011524383	SLFN13	3402	3422	1	1	0.00	0.00	20	9	3UTR
hsa-miR-11400	XM_011524383	SLFN13	2952	2977	1	1	0.00	0.00	25	8	3UTR
hsa-miR-11400	NM_001271946	SLIT3	6993	7015	0.961538	1	3.25	4.16	22	10	3UTR
hsa-miR-11400	NM_003062	SLIT3	6972	6994	0.961538	1	3.25	4.16	22	10	3UTR
hsa-miR-11400	XM_017009779	SLIT3	6365	6387	1	1	0.00	0.00	22	10	3UTR

mirnaid	refseqid	genesymbol	start	end	bindingp	seed	phylopstem	phylopflank	binding_region_length	longest_consecutive_pairings	position
hsa-miR-11400	NM_052910	SLITRK1	3395	3430	1	1	3.37	3.45	21	11	3UTR
hsa-miR-11400	NM_005901	SMAD2	24222	24242	1	1	1.72	1.05	20	16	3UTR
hsa-miR-11400	NM_005901	SMAD2	29286	29308	1	1	0.05	0.15	22	9	3UTR
hsa-miR-11400	NM_001003652	SMAD2	23996	24016	1	1	0.01	-0.01	20	16	3UTR
hsa-miR-11400	NM_001003652	SMAD2	29060	29082	1	1	0.28	0.43	22	9	3UTR
hsa-miR-11400	NM_001135937	SMAD2	23906	23926	1	1	0.00	0.00	20	16	3UTR
hsa-miR-11400	NM_001135937	SMAD2	28970	28992	1	1	0.00	0.00	22	9	3UTR
hsa-miR-11400	NM_001145104	SMAD3	5082	5100	1	1	-0.18	0.02	18	8	3UTR
hsa-miR-11400	XM_024446047	SMAD5	3445	3463	1	1	0.00	0.00	18	10	3UTR
hsa-miR-11400	NM_005903	SMAD5	2515	2533	1	1	0.99	1.03	18	10	3UTR
hsa-miR-11400	NM_001001420	SMAD5	2440	2458	1	1	0.99	1.03	18	10	3UTR
hsa-miR-11400	NM_001317946	SMARCB1	4494	4511	1	1	-0.06	-0.46	17	16	3UTR
hsa-miR-11400	NM_001007468	SMARCB1	4440	4457	1	1	-0.06	-0.46	17	16	3UTR
hsa-miR-11400	NM_001077657	SMCO1	914	942	1	1	0.33	0.06	28	6	3UTR
hsa-miR-11400	NM_138428	SMIM12	2886	2916	1	1	-0.10	-0.20	17	15	3UTR
hsa-miR-11400	XM_005270403	SMIM12	3436	3466	1	1	0.00	0.00	17	15	3UTR
hsa-miR-11400	NM_001320261	SMIM12	2911	2941	1	1	-0.24	-0.17	17	15	3UTR

mirnaid	refseqid	genesymbol	start	end	bindingp	seed	phylopstem	phylopflank	binding_region_length	longest_consecutive_pairings	position
hsa-miR-11400	NM_001164824	SMIM12	3203	3233	1	1	-0.46	0.10	17	15	3UTR
hsa-miR-11400	NM_001164825	SMIM12	3108	3138	1	1	-0.71	0.13	17	15	3UTR
hsa-miR-11400	NM_024104	SMIM7	762	789	1	1	-0.18	-0.19	17	7	3UTR
hsa-miR-11400	NM_001297715	SMN1	1251	1273	1	1	-0.18	-0.14	22	8	3UTR
hsa-miR-11400	XM_011543597	SMN1	989	1011	1	1	0.00	0.00	22	8	3UTR
hsa-miR-11400	NM_022874	SMN1	1209	1231	1	1	-0.18	-0.14	22	8	3UTR
hsa-miR-11400	NM_000344	SMN1	1159	1181	1	1	3.32	2.97	22	8	3UTR
hsa-miR-11400	XM_011543600	SMN2	989	1011	1	1	0.00	0.00	22	8	3UTR
hsa-miR-11400	NM_017411	SMN2	1159	1181	1	1	-0.18	-0.14	22	8	3UTR
hsa-miR-11400	NM_022875	SMN2	1105	1127	1	1	-0.18	-0.14	22	8	3UTR
hsa-miR-11400	NM_022876	SMN2	1209	1231	1	1	-0.18	-0.14	22	8	3UTR
hsa-miR-11400	NM_005871	SMNDC1	2945	2965	1	1	0.30	0.63	20	9	3UTR
hsa-miR-11400	NM_018667	SMPD3	2495	2517	1	1	2.62	2.48	22	7	3UTR
hsa-miR-11400	NM_004782	SNAP29	2673	2701	1	1	-0.54	-0.17	28	10	3UTR
hsa-miR-11400	XM_017015056	SNAPC3	3598	3635	1	1	0.00	0.00	16	11	3UTR
hsa-miR-11400	NM_000345	SNCA	2411	2429	0.961538	1	2.42	1.87	18	10	3UTR
hsa-miR-11400	NM_001375290	SNCA	2212	2236	1	1	2.55	1.95	19	10	3UTR

mirnaid	refseqid	genesymbol	start	end	bindingp	seed	phylopstem	phylopflank	binding_region_length	longest_consecutive_pairings	position
hsa-miR-11400	NM_001146054	SNCA	2630	2648	1	1	0.90	2.81	18	10	3UTR
hsa-miR-11400	NM_001146055	SNCA	2258	2282	1	1	2.66	2.37	19	10	3UTR
hsa-miR-11400	NM_024700	SNIP1	2784	2807	1	1	1.07	1.10	23	8	3UTR
hsa-miR-11400	NM_006938	SNRPD1	2024	2049	1	1	-0.10	0.04	25	8	3UTR
hsa-miR-11400	NM_006938	SNRPD1	825	859	1	1	0.12	0.12	34	11	3UTR
hsa-miR-11400	NM_021021	SNTB1	2176	2198	1	1	0.18	0.25	22	9	3UTR
hsa-miR-11400	NM_006750	SNTB2	7513	7532	1	1	-0.52	-0.01	19	10	3UTR
hsa-miR-11400	NM_005008	SNU13	1483	1514	1	1	0.28	-0.03	31	9	3UTR
hsa-miR-11400	NM_148955	SNX1	4122	4140	1	1	0.63	0.25	18	8	3UTR
hsa-miR-11400	XM_005257262	SNX11	1918	1938	0.961538	1	0.00	0.00	20	6	3UTR
hsa-miR-11400	XM_011524697	SNX11	1960	1980	0.961538	1	0.00	0.00	20	6	3UTR
hsa-miR-11400	NM_001256185	SNX12	982	1003	1	1	0.43	0.68	21	13	3UTR
hsa-miR-11400	NM_001256188	SNX12	832	853	1	1	1.27	0.74	21	13	3UTR
hsa-miR-11400	NM_001347927	SNX19	10418	10448	1	1	-0.70	-0.42	22	11	3UTR
hsa-miR-11400	NM_001144972	SNX20	1109	1127	1	1	-0.57	-0.27	18	14	3UTR
hsa-miR-11400	XM_017022581	SNX22	2622	2642	0.961538	1	0.00	0.00	20	14	3UTR
hsa-miR-11400	NM_024798	SNX22	2682	2702	1	1	-0.36	-0.15	20	14	3UTR

mirnaid	refseqid	genesymbol	start	end	bindingp	seed	phylopstem	phylopflank	binding_region_length	longest_consecutive_pairings	position
hsa-miR-11400	NM_030918	SNX27	5492	5513	1	1	-0.53	-0.23	21	11	3UTR
hsa-miR-11400	XM_005245510	SNX27	4311	4332	1	1	0.00	0.00	21	11	3UTR
hsa-miR-11400	XM_017002417	SNX27	5057	5078	1	1	0.00	0.00	21	11	3UTR
hsa-miR-11400	NM_001330723	SNX27	4715	4736	1	1	-0.53	-0.23	21	11	3UTR
hsa-miR-11400	NM_152238	SNX7	1422	1441	1	1	0.65	0.88	19	8	3UTR
hsa-miR-11400	NM_015976	SNX7	1587	1606	1	1	0.65	0.88	19	8	3UTR
hsa-miR-11400	XM_017026086	SOCS6	4569	4593	1	1	0.00	0.00	17	15	3UTR
hsa-miR-11400	NM_004232	SOCS6	4419	4443	1	1	0.01	0.04	17	15	3UTR
hsa-miR-11400	NM_001322814	SOD2	11732	11750	0.961538	1	-0.32	0.01	18	8	3UTR
hsa-miR-11400	NM_001322814	SOD2	4911	4930	1	1	0.13	-0.02	19	12	3UTR
hsa-miR-11400	NM_001322815	SOD2	4848	4867	1	1	-0.12	0.02	19	12	3UTR
hsa-miR-11400	NM_000636	SOD2	5028	5047	1	1	0.13	0.01	19	12	3UTR
hsa-miR-11400	NM_002959	SORT1	2959	3009	1	1	1.05	1.06	24	8	3UTR
hsa-miR-11400	NM_001205228	SORT1	2733	2783	1	1	0.00	0.00	24	8	3UTR
hsa-miR-11400	NM_025237	SOST	894	942	1	1	-0.37	0.01	21	8	3UTR
hsa-miR-11400	NM_007084	SOX21	1464	1485	1	1	1.22	0.30	21	7	3UTR
hsa-miR-11400	NM_001261414	SOX5	2774	2791	1	1	3.96	1.74	17	11	3UTR

mirnaid	refseqid	genesymbol	start	end	bindingp	seed	phylopstem	phylopflank	binding_region_length	longest_consecutive_pairings	position
hsa-miR-11400	NM_001261415	SOX5	2667	2684	1	1	3.39	3.10	17	11	3UTR
hsa-miR-11400	NM_178010	SOX5	1522	1539	1	1	0.51	1.10	17	11	3UTR
hsa-miR-11400	NM_006940	SOX5	2715	2732	1	1	3.45	2.57	17	11	3UTR
hsa-miR-11400	NM_001330785	SOX5	2610	2627	1	1	2.27	4.31	17	11	3UTR
hsa-miR-11400	NM_138473	SP1	4821	4846	1	1	0.33	0.74	25	10	3UTR
hsa-miR-11400	NM_003109	SP1	4739	4764	1	1	0.33	0.74	25	10	3UTR
hsa-miR-11400	NM_001080391	SP100	4593	4626	1	1	0.08	-0.03	33	8	3UTR
hsa-miR-11400	NM_199262	SP6	1268	1286	1	1	-0.36	-0.25	18	11	3UTR
hsa-miR-11400	NM_182700	SP8	3129	3144	1	1	2.18	1.27	15	9	3UTR
hsa-miR-11400	NM_198956	SP8	3225	3240	1	1	2.26	1.31	15	9	3UTR
hsa-miR-11400	NM_058206	SPAG11B	317	360	1	1	1.44	0.30	21	10	3UTR
hsa-miR-11400	NM_138796	SPATA17	2371	2394	1	1	0.01	-0.07	23	7	3UTR
hsa-miR-11400	NM_001145197	SPATA31D4	4057	4074	1	1	-0.71	-0.79	17	9	3UTR
hsa-miR-11400	NM_001353486	SPATA6L	2290	2310	1	1	0.18	0.83	20	6	3UTR
hsa-miR-11400	NM_198572	SPATC1	2046	2066	1	1	-0.68	-0.17	20	10	3UTR
hsa-miR-11400	NM_001134374	SPATC1	1906	1926	1	1	-0.68	-0.17	20	10	3UTR
hsa-miR-11400	NM_145026	SPATS1	2782	2811	1	1	0.10	-0.09	21	11	3UTR

mirnaid	refseqid	genesymbol	start	end	bindingp	seed	phylopstem	phylopflank	binding_region_length	longest_consecutive_pairings	position
hsa-miR-11400	NM_001372081	SPATS1	2790	2819	1	1	0.10	-0.09	21	11	3UTR
hsa-miR-11400	NM_014752	SPCS2	1742	1782	1	1	0.21	0.21	20	7	3UTR
hsa-miR-11400	NM_152904	SPECC1	3977	3997	0.980769	1	-0.82	-0.20	20	9	3UTR
hsa-miR-11400	NM_001033554	SPECC1	3808	3828	0.980769	1	-0.82	-0.20	20	9	3UTR
hsa-miR-11400	NM_001243438	SPECC1	3817	3837	1	1	0.00	0.00	20	9	3UTR
hsa-miR-11400	NM_020148	SPIRE1	2909	2928	1	1	1.40	0.55	19	6	3UTR
hsa-miR-11400	NM_001128626	SPIRE1	2951	2970	1	1	0.27	0.72	19	6	3UTR
hsa-miR-11400	NM_001030288	SPN	6839	6864	0.961538	1	-0.05	-0.07	25	8	3UTR
hsa-miR-11400	NM_001030288	SPN	3908	3926	1	1	-0.04	-0.01	18	6	3UTR
hsa-miR-11400	XM_011532018	SPOCK3	1896	1921	1	1	0.00	0.00	25	7	3UTR
hsa-miR-11400	XM_017008257	SPOCK3	1820	1845	1	1	0.00	0.00	25	7	3UTR
hsa-miR-11400	XM_017008258	SPOCK3	1960	1985	1	1	0.00	0.00	25	7	3UTR
hsa-miR-11400	NM_001251967	SPOCK3	1544	1569	1	1	0.00	0.00	25	7	3UTR
hsa-miR-11400	NM_032802	SPPL2A	6633	6654	1	1	2.25	2.19	21	8	3UTR
hsa-miR-11400	NM_001042522	SPRED3	4485	4506	1	1	1.40	1.48	21	11	3UTR
hsa-miR-11400	NM_024738	SPRING1	8131	8148	1	1	4.01	3.69	17	11	3UTR
hsa-miR-11400	NM_001304990	SPRY3	5261	5282	1	1	0.29	0.15	21	12	3UTR

mirnaid	refseqid	genesymbol	start	end	bindingp	seed	phylopstem	phylopflank	binding_region_length	longest_consecutive_pairings	position
hsa-miR-11400	NM_005840	SPRY3	5405	5426	1	1	0.00	0.00	21	12	3UTR
hsa-miR-11400	NM_032840	SPRYD3	2571	2596	0.961538	1	4.17	4.37	25	11	3UTR
hsa-miR-11400	NM_207344	SPRYD4	10252	10291	1	1	0.59	2.56	22	10	3UTR
hsa-miR-11400	NM_025106	SPSB1	1407	1437	1	1	0.54	-0.02	30	10	3UTR
hsa-miR-11400	NM_001355436	SPTB	7632	7653	1	1	1.14	2.15	21	10	3UTR
hsa-miR-11400	NM_001024858	SPTB	7751	7772	1	1	3.93	1.51	21	10	3UTR
hsa-miR-11400	NM_178324	SPTLC1	870	895	1	1	4.02	3.49	25	8	3UTR
hsa-miR-11400	XM_024453379	SPTSSB	794	835	0.980769	1	0.00	0.00	20	11	3UTR
hsa-miR-11400	NM_001040100	SPTSSB	1206	1225	1	1	0.30	0.39	19	11	3UTR
hsa-miR-11400	NM_030572	SPX	669	689	0.961538	1	0.63	0.15	20	11	3UTR
hsa-miR-11400	NM_032567	SPZ1	1735	1778	1	1	0.10	-0.13	14	6	3UTR
hsa-miR-11400	NM_198291	SRC	4407	4428	1	1	0.04	-0.28	21	10	3UTR
hsa-miR-11400	NM_173829	SREK1IP1	3744	3774	1	1	0.60	0.28	30	11	3UTR
hsa-miR-11400	NM_173829	SREK1IP1	6177	6206	1	1	0.36	1.09	29	8	3UTR
hsa-miR-11400	XM_017023528	SRL	3127	3143	1	1	0.00	0.00	16	11	3UTR
hsa-miR-11400	NM_003137	SRPK1	3374	3399	1	1	2.15	2.38	25	12	3UTR
hsa-miR-11400	NM_003139	SRPRA	2438	2453	1	1	4.13	3.88	15	14	3UTR

mirnaid	refseqid	genesymbol	start	end	bindingp	seed	phylopstem	phylopflank	binding_region_length	longest_consecutive_pairings	position
hsa-miR-11400	NM_001177842	SRPRA	2354	2369	1	1	1.12	2.25	15	14	3UTR
hsa-miR-11400	NM_001013694	SRRD	1579	1598	1	1	3.67	4.04	19	9	3UTR
hsa-miR-11400	NM_006275	SRSF6	4185	4213	1	1	0.13	-0.15	28	10	3UTR
hsa-miR-11400	NM_001256733	SSBP2	3309	3328	1	1	0.03	0.04	19	10	3UTR
hsa-miR-11400	NM_001256734	SSBP2	3303	3322	1	1	0.02	0.03	19	10	3UTR
hsa-miR-11400	NM_001256735	SSBP2	3279	3298	1	1	0.01	0.04	19	10	3UTR
hsa-miR-11400	NM_001256736	SSBP2	3180	3199	1	1	0.00	0.00	19	10	3UTR
hsa-miR-11400	XM_017009309	SSBP2	3536	3555	1	1	0.00	0.00	19	10	3UTR
hsa-miR-11400	NM_018984	SSH1	12327	12352	0.961538	1	4.58	4.82	25	8	3UTR
hsa-miR-11400	NM_005086	SSPN	3392	3405	1	1	1.89	1.93	13	12	3UTR
hsa-miR-11400	NM_001135823	SSPN	3209	3222	1	1	1.89	1.93	13	12	3UTR
hsa-miR-11400	NM_001292008	SSR1	3898	3921	1	1	0.06	0.12	23	7	3UTR
hsa-miR-11400	NM_003144	SSR1	4102	4125	1	1	0.16	-0.05	23	7	3UTR
hsa-miR-11400	NM_001308197	SSR3	2655	2687	1	1	0.05	0.01	22	17	3UTR
hsa-miR-11400	NM_001308197	SSR3	2965	2982	1	1	0.29	0.64	17	13	3UTR
hsa-miR-11400	NM_001308204	SSR3	2564	2596	1	1	0.16	-0.08	22	17	3UTR
hsa-miR-11400	NM_001308204	SSR3	2874	2891	1	1	0.27	0.34	17	13	3UTR

mirnaid	refseqid	genesymbol	start	end	bindingp	seed	phylopstem	phylopflank	binding_region_length	longest_consecutive_pairings	position
hsa-miR-11400	NM_001308205	SSR3	2588	2620	1	1	0.05	0.05	22	17	3UTR
hsa-miR-11400	NM_001308205	SSR3	2898	2915	1	1	0.38	0.50	17	13	3UTR
hsa-miR-11400	NM_007107	SSR3	2616	2648	1	1	-0.05	0.04	22	17	3UTR
hsa-miR-11400	NM_152996	ST6GALNAC3	4548	4571	1	1	1.10	0.79	23	7	3UTR
hsa-miR-11400	NM_001286999	ST6GALNAC6	1630	1664	1	1	1.88	3.38	19	8	3UTR
hsa-miR-11400	NM_001287001	ST6GALNAC6	1665	1683	1	1	1.81	2.85	18	8	3UTR
hsa-miR-11400	NM_013443	ST6GALNAC6	1634	1668	1	1	1.88	3.38	19	8	3UTR
hsa-miR-11400	NM_003034	ST8SIA1	7730	7750	1	1	0.96	0.59	20	8	3UTR
hsa-miR-11400	NM_006011	ST8SIA2	2797	2814	1	1	0.10	0.57	17	9	3UTR
hsa-miR-11400	NM_001330416	ST8SIA2	2734	2751	1	1	0.55	0.49	17	9	3UTR
hsa-miR-11400	NM_001307987	ST8SIA5	3841	3865	1	1	-0.38	0.00	24	9	3UTR
hsa-miR-11400	NM_013305	ST8SIA5	3934	3958	1	1	0.41	0.04	24	9	3UTR
hsa-miR-11400	NM_181900	STARD5	1678	1709	1	1	0.33	-0.26	18	16	3UTR
hsa-miR-11400	NM_181900	STARD5	4709	4729	1	1	3.03	2.38	20	9	3UTR
hsa-miR-11400	NM_001178080	STAT6	2803	2829	1	1	2.74	3.55	26	8	3UTR
hsa-miR-11400	NM_001205316	STEAP4	8248	8266	1	1	0.00	0.00	18	12	3UTR
hsa-miR-11400	NM_001382581	STIM1	2773	2809	1	1	0.74	0.67	21	7	3UTR

mirnaid	refseqid	genesymbol	start	end	bindingp	seed	phylopstem	phylopflank	binding_region_length	longest_consecutive_pairings	position
hsa-miR-11400	NM_001271979	STK25	2008	2026	1	1	-0.23	-0.23	18	9	3UTR
hsa-miR-11400	NM_001271980	STK25	1946	1964	1	1	0.23	-0.22	18	9	3UTR
hsa-miR-11400	NM_001282305	STK25	2317	2335	1	1	-0.42	-0.26	18	9	3UTR
hsa-miR-11400	NM_007271	STK38	2696	2712	1	1	4.56	5.40	16	15	3UTR
hsa-miR-11400	NM_001256674	STOML1	5541	5562	1	1	2.81	3.10	21	12	3UTR
hsa-miR-11400	NM_001256675	STOML1	5481	5502	1	1	3.23	3.45	21	12	3UTR
hsa-miR-11400	NM_004809	STOML1	5694	5715	1	1	3.23	3.45	21	12	3UTR
hsa-miR-11400	NM_007178	STRAP	1406	1441	1	1	2.51	1.33	21	7	3UTR
hsa-miR-11400	XM_017026717	STRN4	2329	2378	1	1	0.00	0.00	19	8	3UTR
hsa-miR-11400	NM_003764	STX11	3487	3504	1	1	0.17	-0.07	17	8	3UTR
hsa-miR-11400	NM_003763	STX16	1718	1734	1	1	0.49	0.03	16	15	3UTR
hsa-miR-11400	NM_001001433	STX16	1781	1797	1	1	0.49	0.03	16	15	3UTR
hsa-miR-11400	NM_001134772	STX16	1769	1785	1	1	0.49	0.03	16	15	3UTR
hsa-miR-11400	NM_001134773	STX16	1730	1746	1	1	0.00	0.00	16	15	3UTR
hsa-miR-11400	NM_001204868	STX16	1143	1159	1	1	0.95	1.26	16	15	3UTR
hsa-miR-11400	NM_004603	STX1A	1112	1132	1	1	1.26	0.25	20	7	3UTR
hsa-miR-11400	NM_052874	STX1B	3069	3102	1	1	0.00	0.13	22	12	3UTR

mirnaid	refseqid	genesymbol	start	end	bindingp	seed	phylopstem	phylopflank	binding_region_length	longest_consecutive_pairings	position
hsa-miR-11400	NM_001244666	STX5	1475	1491	0.961538	1	0.00	0.00	16	15	3UTR
hsa-miR-11400	NM_139244	STXBP5	6640	6690	1	1	1.54	0.78	28	11	3UTR
hsa-miR-11400	NM_001127715	STXBP5	6748	6798	1	1	1.54	0.78	28	11	3UTR
hsa-miR-11400	NM_001304477	STXBP6	2585	2603	1	1	0.23	0.99	18	8	3UTR
hsa-miR-11400	XM_017021239	STXBP6	2649	2667	1	1	0.00	0.00	18	8	3UTR
hsa-miR-11400	XM_024451308	SUGP2	5766	5789	0.961538	1	0.00	0.00	23	13	3UTR
hsa-miR-11400	NM_001352071	SUGP2	5179	5202	1	1	0.74	1.21	23	13	3UTR
hsa-miR-11400	NM_001017392	SUGP2	5177	5200	1	1	0.97	1.28	23	13	3UTR
hsa-miR-11400	XM_006716442	SULF1	2000	2026	1	1	0.00	0.00	26	8	3UTR
hsa-miR-11400	NM_006753	SURF6	3393	3413	1	1	2.17	2.98	20	11	3UTR
hsa-miR-11400	XM_017022761	SV2B	11089	11111	1	1	0.00	0.00	22	15	3UTR
hsa-miR-11400	NM_033025	SYDE1	2760	2800	1	1	-0.14	0.19	40	12	3UTR
hsa-miR-11400	NM_003177	SYK	2800	2819	1	1	-0.54	-0.77	19	9	3UTR
hsa-miR-11400	NM_001135052	SYK	2731	2750	1	1	0.00	0.00	19	9	3UTR
hsa-miR-11400	NM_001174167	SYK	2885	2904	1	1	-0.54	-0.77	19	9	3UTR
hsa-miR-11400	NM_001174168	SYK	3062	3081	1	1	-0.54	-0.77	19	9	3UTR
hsa-miR-11400	NM_003178	SYN2	2821	2842	1	1	-0.10	0.00	21	7	3UTR

mirnaid	refseqid	genesymbol	start	end	bindingp	seed	phylopstem	phylopflank	binding_region_length	longest_consecutive_pairings	position
hsa-miR-11400	NM_003490	SYN3	6449	6472	1	1	4.09	3.70	23	8	3UTR
hsa-miR-11400	XM_005267200	SYNJ2	4798	4831	1	1	0.00	0.00	24	14	3UTR
hsa-miR-11400	XM_006715592	SYNJ2	6250	6283	1	1	0.00	0.00	24	14	3UTR
hsa-miR-11400	NM_003898	SYNJ2	6093	6126	1	1	-0.40	-0.17	24	14	3UTR
hsa-miR-11400	NM_001178088	SYNJ2	5978	6011	1	1	-0.40	-0.17	24	14	3UTR
hsa-miR-11400	NM_018373	SYNJ2BP	3180	3200	1	1	0.04	0.21	20	13	3UTR
hsa-miR-11400	NM_018373	SYNJ2BP	5252	5274	1	1	-0.06	0.11	22	7	3UTR
hsa-miR-11400	NM_144642	SYNPR	1835	1860	1	1	0.83	0.43	25	8	3UTR
hsa-miR-11400	NM_001130003	SYNPR	1987	2012	1	1	0.83	0.43	25	8	3UTR
hsa-miR-11400	NM_080550	SYNRG	7128	7153	1	1	4.40	4.68	25	8	3UTR
hsa-miR-11400	NM_198882	SYNRG	7233	7258	1	1	4.53	4.81	25	8	3UTR
hsa-miR-11400	NM_001163545	SYNRG	7161	7186	1	1	4.51	4.93	25	8	3UTR
hsa-miR-11400	NM_001163546	SYNRG	6993	7018	1	1	4.40	4.68	25	8	3UTR
hsa-miR-11400	NM_001163547	SYNRG	6780	6805	1	1	4.53	4.81	25	8	3UTR
hsa-miR-11400	NM_020826	SYT13	4895	4913	1	1	0.57	1.85	18	10	3UTR
hsa-miR-11400	NM_031912	SYT15	3295	3318	1	1	-0.18	0.02	23	10	3UTR
hsa-miR-11400	XM_024448228	SYT15	4200	4223	1	1	0.00	0.00	23	10	3UTR

mirnaid	refseqid	genesymbol	start	end	bindingp	seed	phylopstem	phylopflank	binding_region_length	longest_consecutive_pairings	position
hsa-miR-11400	NM_001367656	SYT16	5314	5339	1	1	0.69	0.62	25	12	3UTR
hsa-miR-11400	NM_001308157	SYT17	1691	1711	1	1	-0.09	2.27	20	8	3UTR
hsa-miR-11400	NM_016524	SYT17	1833	1853	1	1	-0.09	2.27	20	8	3UTR
hsa-miR-11400	NM_001253772	SYT6	1744	1765	1	1	-0.32	0.18	21	13	3UTR
hsa-miR-11400	NM_205848	SYT6	1624	1645	1	1	0.68	0.84	21	13	3UTR
hsa-miR-11400	NM_001366223	SYT6	1641	1662	1	1	1.25	0.60	21	13	3UTR
hsa-miR-11400	NM_001366225	SYT6	1723	1744	1	1	-0.02	0.03	21	13	3UTR
hsa-miR-11400	NM_175733	SYT9	2654	2674	1	1	0.19	0.54	20	8	3UTR
hsa-miR-11400	XM_011519906	SYT9	2240	2259	1	1	0.00	0.00	19	8	3UTR
hsa-miR-11400	NM_006283	TACC1	4859	4878	1	1	0.32	-0.04	19	9	3UTR
hsa-miR-11400	NM_001352786	TACC1	5054	5073	1	1	0.32	-0.04	19	9	3UTR
hsa-miR-11400	NM_001352792	TACC1	4438	4457	1	1	0.32	-0.04	19	9	3UTR
hsa-miR-11400	NM_001352798	TACC1	4405	4424	1	1	0.32	-0.04	19	9	3UTR
hsa-miR-11400	NM_001352799	TACC1	4351	4370	1	1	0.32	-0.04	19	9	3UTR
hsa-miR-11400	NM_001122824	TACC1	3629	3648	1	1	0.32	-0.04	19	9	3UTR
hsa-miR-11400	NM_001146216	TACC1	4441	4460	1	1	0.32	-0.04	19	9	3UTR
hsa-miR-11400	NM_139353	TAF1C	3226	3246	1	1	2.03	0.89	20	15	3UTR

mirnaid	refseqid	genesymbol	start	end	bindingp	seed	phylopstem	phylopflank	binding_region_length	longest_consecutive_pairings	position
hsa-miR-11400	NM_005679	TAF1C	3468	3488	1	1	0.72	0.66	20	15	3UTR
hsa-miR-11400	NM_001243156	TAF1C	3390	3410	1	1	0.72	0.66	20	15	3UTR
hsa-miR-11400	NM_001243157	TAF1C	3024	3044	1	1	0.00	0.00	20	15	3UTR
hsa-miR-11400	NM_001243158	TAF1C	2885	2905	1	1	1.23	0.55	20	15	3UTR
hsa-miR-11400	NM_001243159	TAF1C	3357	3377	1	1	2.03	0.89	20	15	3UTR
hsa-miR-11400	XM_005273099	TAF5L	2311	2333	1	1	0.00	0.00	22	9	3UTR
hsa-miR-11400	NM_025185	TANC2	7836	7854	0.980769	1	-0.31	0.10	18	11	3UTR
hsa-miR-11400	XM_006721811	TANC2	5514	5533	1	1	0.00	0.00	19	8	3UTR
hsa-miR-11400	NM_153365	TAPT1	4050	4071	1	1	4.15	4.56	21	11	3UTR
hsa-miR-11400	NM_001271845	TBC1D16	3331	3352	1	1	-0.38	-0.01	21	10	3UTR
hsa-miR-11400	NM_001292054	TBC1D19	2339	2355	1	1	0.65	0.29	16	13	3UTR
hsa-miR-11400	NM_018317	TBC1D19	2534	2550	1	1	0.71	0.13	16	13	3UTR
hsa-miR-11400	NM_178571	TBC1D26	1125	1147	1	1	0.32	0.57	22	6	3UTR
hsa-miR-11400	XM_017020882	TBC1D4	3354	3372	1	1	0.00	0.00	18	8	3UTR
hsa-miR-11400	NM_001349077	TBC1D5	4006	4030	1	1	1.13	1.68	24	9	3UTR
hsa-miR-11400	NM_001134381	TBC1D5	2739	2755	1	1	-0.02	0.02	16	15	3UTR
hsa-miR-11400	NM_198868	TBC1D9B	5022	5041	0.974359	1	0.00	0.00	19	15	3UTR

mirnaid	refseqid	genesymbol	start	end	bindingp	seed	phylopstem	phylopflank	binding_region_length	longest_consecutive_pairings	position
hsa-miR-11400	NM_152715	TBCEL	4689	4714	0.980769	1	-0.57	0.12	20	13	3UTR
hsa-miR-11400	NM_001130047	TBCEL	4534	4577	1	1	-0.31	-0.02	20	13	3UTR
hsa-miR-11400	NM_199047	TBPL2	1181	1198	1	1	0.53	0.42	17	8	3UTR
hsa-miR-11400	NM_001080508	TBX18	2439	2458	1	1	0.72	0.26	19	8	3UTR
hsa-miR-11400	NM_001282913	TCAIM	2351	2373	1	1	0.02	-0.14	22	7	3UTR
hsa-miR-11400	NM_001282913	TCAIM	2902	2913	1	1	-0.29	-0.81	11	10	3UTR
hsa-miR-11400	NM_173826	TCAIM	2229	2251	1	1	0.02	-0.14	22	7	3UTR
hsa-miR-11400	NM_173826	TCAIM	2741	2791	1	1	-0.52	-0.51	12	10	3UTR
hsa-miR-11400	NM_153035	TCEANC2	4726	4744	1	1	0.43	0.24	18	11	3UTR
hsa-miR-11400	XM_005256298	TCF25	2230	2253	1	1	0.00	0.00	23	10	3UTR
hsa-miR-11400	XM_017023054	TCF25	2373	2396	1	1	0.00	0.00	23	10	3UTR
hsa-miR-11400	XM_005266752	TCF4	2690	2710	1	1	0.00	0.00	20	8	3UTR
hsa-miR-11400	XM_017025956	TCF4	2101	2121	1	1	0.00	0.00	20	8	3UTR
hsa-miR-11400	NM_001243227	TCF4	2722	2742	1	1	-0.39	0.94	20	8	3UTR
hsa-miR-11400	NM_001243230	TCF4	2340	2360	1	1	0.00	0.00	20	8	3UTR
hsa-miR-11400	NM_001243233	TCF4	2081	2101	1	1	0.00	0.00	20	8	3UTR
hsa-miR-11400	NM_001243236	TCF4	1963	1983	1	1	0.00	0.00	20	8	3UTR

mirnaid	refseqid	genesymbol	start	end	bindingp	seed	phylopstem	phylopflank	binding_region_length	longest_consecutive_pairings	position
hsa-miR-11400	NM_021966	TCL1A	781	802	1	1	0.08	-0.29	16	14	3UTR
hsa-miR-11400	NM_001098725	TCL1A	776	797	1	1	-0.26	-0.37	16	14	3UTR
hsa-miR-11400	XM_011520207	TCP11L1	5914	5936	1	1	0.00	0.00	22	9	3UTR
hsa-miR-11400	NM_001368158	TCP11X1	1669	1690	1	1	-0.07	0.37	21	14	3UTR
hsa-miR-11400	NM_001277423	TCP11X2	1669	1690	1	1	0.00	0.00	21	14	3UTR
hsa-miR-11400	NM_153046	TDRD9	4729	4749	1	1	0.10	0.53	18	9	3UTR
hsa-miR-11400	NM_015395	TECPR1	4916	4939	1	1	0.19	0.10	17	15	3UTR
hsa-miR-11400	NM_053285	TEKT1	2806	2834	1	1	2.46	2.91	28	10	3UTR
hsa-miR-11400	NM_001080428	TENM2	7809	7835	1	1	3.01	2.97	26	8	3UTR
hsa-miR-11400	XM_024454343	TENT4A	3328	3348	1	1	0.00	0.00	20	11	3UTR
hsa-miR-11400	NM_017633	TENT5A	3865	3883	1	1	0.87	0.96	18	6	3UTR
hsa-miR-11400	NM_004923	TESMIN	1823	1862	1	1	1.15	0.77	16	14	3UTR
hsa-miR-11400	NM_001039656	TESMIN	2842	2859	1	1	2.49	1.31	17	8	3UTR
hsa-miR-11400	NM_017628	TET2	6630	6661	1	1	0.23	0.05	31	10	3UTR
hsa-miR-11400	NM_001288732	TEX2	3875	3897	1	1	4.52	3.31	22	10	3UTR
hsa-miR-11400	XM_011524998	TEX2	3948	3970	1	1	0.00	0.00	22	10	3UTR
hsa-miR-11400	NM_018469	TEX2	3896	3918	1	1	4.52	3.31	22	10	3UTR

mirnaid	refseqid	genesymbol	start	end	bindingp	seed	phylopstem	phylopflank	binding_region_length	longest_consecutive_pairings	position
hsa-miR-11400	NM_144582	TEX261	1341	1363	1	1	0.63	0.23	22	9	3UTR
hsa-miR-11400	NM_001351123	TEX49	534	560	1	1	0.01	0.30	21	7	3UTR
hsa-miR-11400	NM_014553	TFCP2L1	6461	6482	1	1	-0.34	-0.10	21	8	3UTR
hsa-miR-11400	NM_006521	TFE3	2125	2139	1	1	3.30	3.93	14	13	3UTR
hsa-miR-11400	NM_012252	TFEC	3679	3699	1	1	-0.27	-0.20	20	10	3UTR
hsa-miR-11400	NM_001018058	TFEC	3592	3612	1	1	0.03	-0.03	20	10	3UTR
hsa-miR-11400	NM_001008697	TFIP11	3302	3321	1	1	0.37	1.87	19	15	3UTR
hsa-miR-11400	NM_006287	TFPI	2677	2721	1	1	-0.14	0.26	19	17	3UTR
hsa-miR-11400	NM_001329240	TFPI	2708	2752	1	1	0.41	0.38	19	17	3UTR
hsa-miR-11400	NM_000660	TGFB1	2317	2337	1	1	0.71	0.40	20	16	3UTR
hsa-miR-11400	NM_000660	TGFB1	2611	2660	1	1	0.55	0.62	49	9	3UTR
hsa-miR-11400	NM_001199515	TGIF2	3284	3300	0.974359	1	3.43	1.70	16	9	3UTR
hsa-miR-11400	NM_001368096	TGOLN2	1547	1570	1	1	-0.02	0.04	23	9	3UTR
hsa-miR-11400	NM_001206844	TGOLN2	1356	1379	1	1	0.36	0.88	23	9	3UTR
hsa-miR-11400	NM_020147	THAP10	1340	1364	1	1	1.00	0.59	17	10	3UTR
hsa-miR-11400	XM_005262774	THAP6	1794	1817	1	1	0.00	0.00	23	13	3UTR
hsa-miR-11400	NM_001289997	THPO	1347	1366	1	1	1.56	2.17	19	12	3UTR

mirnaid	refseqid	genesymbol	start	end	bindingp	seed	phylopstem	phylopflank	binding_region_length	longest_consecutive_pairings	position
hsa-miR-11400	NM_000460	THPO	1462	1481	1	1	1.75	2.24	19	12	3UTR
hsa-miR-11400	NM_001177597	THPO	1451	1470	1	1	1.56	2.17	19	12	3UTR
hsa-miR-11400	NM_001177598	THPO	1446	1465	1	1	1.56	2.17	19	12	3UTR
hsa-miR-11400	NM_001252634	THRB	5246	5262	1	1	0.02	0.22	16	15	3UTR
hsa-miR-11400	NM_001354712	THRB	5157	5173	1	1	0.20	0.34	16	15	3UTR
hsa-miR-11400	NM_001374822	THRB	4947	4963	1	1	0.19	0.38	16	15	3UTR
hsa-miR-11400	NM_001374823	THRB	5079	5095	1	1	0.21	0.05	16	15	3UTR
hsa-miR-11400	NM_001374824	THRB	5252	5268	1	1	-0.22	0.22	16	15	3UTR
hsa-miR-11400	NM_001374825	THRB	4959	4975	1	1	0.43	0.50	16	15	3UTR
hsa-miR-11400	NM_000461	THRB	5011	5027	1	1	0.21	0.17	16	15	3UTR
hsa-miR-11400	NM_001128176	THRB	5122	5138	1	1	0.25	0.21	16	15	3UTR
hsa-miR-11400	NM_001316349	THSD7B	5333	5358	1	1	2.39	1.47	25	8	3UTR
hsa-miR-11400	NM_001099221	TIFAB	2044	2062	1	1	0.64	-0.22	18	11	3UTR
hsa-miR-11400	NM_030953	TIGD6	3099	3117	1	1	1.28	0.58	18	8	3UTR
hsa-miR-11400	NM_173799	TIGIT	1687	1706	1	1	-0.51	-0.14	19	9	3UTR
hsa-miR-11400	XM_024453388	TIGIT	11508	11528	1	1	0.00	0.00	20	7	3UTR
hsa-miR-11400	NM_004085	TIMM8A	1113	1139	1	1	1.14	2.13	16	14	3UTR

mirnaid	refseqid	genesymbol	start	end	bindingp	seed	phylopstem	phylopflank	binding_region_length	longest_consecutive_pairings	position
hsa-miR-11400	NM_001145951	TIMM8A	2854	2880	1	1	1.14	2.13	16	14	3UTR
hsa-miR-11400	NM_152902	TIPRL	1222	1238	1	1	-0.19	0.22	16	10	3UTR
hsa-miR-11400	NM_001258028	TKT	2524	2545	1	1	0.00	0.00	21	7	3UTR
hsa-miR-11400	NM_001135055	TKT	2359	2380	1	1	0.00	0.00	21	7	3UTR
hsa-miR-11400	XM_006721671	TLCD1	897	924	1	1	0.00	0.00	17	15	3UTR
hsa-miR-11400	NM_152487	TLCD4	1423	1448	1	1	0.16	0.08	25	8	3UTR
hsa-miR-11400	NM_001130	TLE5	938	980	1	1	3.61	2.58	42	10	3UTR
hsa-miR-11400	NM_138554	TLR4	3734	3756	1	1	-0.23	-0.20	22	6	3UTR
hsa-miR-11400	NM_138554	TLR4	11834	11855	1	1	0.46	0.97	21	13	3UTR
hsa-miR-11400	NM_003266	TLR4	3854	3876	1	1	-0.23	-0.20	22	6	3UTR
hsa-miR-11400	NM_003266	TLR4	11954	11975	1	1	0.46	0.97	21	13	3UTR
hsa-miR-11400	NM_006068	TLR6	3900	3922	1	1	3.09	1.14	22	9	3UTR
hsa-miR-11400	NM_006068	TLR6	3586	3610	1	1	2.39	3.58	24	10	3UTR
hsa-miR-11400	NM_138636	TLR8	3834	3855	1	1	0.00	0.00	21	11	3UTR
hsa-miR-11400	NM_138786	TM4SF18	3132	3153	1	1	2.76	2.48	21	11	3UTR
hsa-miR-11400	NM_001184723	TM4SF18	3047	3068	1	1	1.74	2.47	21	11	3UTR
hsa-miR-11400	XM_011539977	TM9SF3	6032	6068	1	1	0.00	0.00	22	8	3UTR

mirnaid	refseqid	genesymbol	start	end	bindingp	seed	phylopstem	phylopflank	binding_region_length	longest_consecutive_pairings	position
hsa-miR-11400	NM_020123	TM9SF3	5863	5899	1	1	0.68	1.30	22	8	3UTR
hsa-miR-11400	NM_001330376	TMED3	1533	1553	1	1	0.17	0.13	20	9	3UTR
hsa-miR-11400	NM_024056	TMEM106C	855	868	1	1	0.94	1.51	13	12	3UTR
hsa-miR-11400	NM_183065	TMEM107	1650	1668	1	1	-0.08	-0.02	18	9	3UTR
hsa-miR-11400	NM_001351278	TMEM107	1647	1665	1	1	-0.11	0.00	18	9	3UTR
hsa-miR-11400	NM_152913	TMEM130	2042	2071	1	1	1.11	1.76	29	9	3UTR
hsa-miR-11400	NM_001134450	TMEM130	2078	2107	1	1	1.11	1.76	29	9	3UTR
hsa-miR-11400	NM_001134451	TMEM130	1736	1765	1	1	1.31	1.73	29	9	3UTR
hsa-miR-11400	NM_001286219	TMEM132B	8206	8225	0.961538	1	0.07	-0.26	19	7	3UTR
hsa-miR-11400	NM_052907	TMEM132B	9459	9478	0.961538	1	0.07	-0.26	19	7	3UTR
hsa-miR-11400	NM_025124	TMEM134	1192	1213	1	1	0.18	0.14	21	11	3UTR
hsa-miR-11400	NM_001078650	TMEM134	1147	1168	1	1	0.14	0.01	21	11	3UTR
hsa-miR-11400	NM_022918	TMEM135	7670	7691	1	1	-0.36	0.04	21	12	3UTR
hsa-miR-11400	NM_001168724	TMEM135	7604	7625	1	1	-0.36	0.04	21	12	3UTR
hsa-miR-11400	XM_011531716	TMEM154	1033	1056	1	1	0.00	0.00	17	15	3UTR
hsa-miR-11400	NM_024943	TMEM156	1092	1134	1	1	0.41	1.07	21	15	3UTR
hsa-miR-11400	XM_005262205	TMEM164	4833	4849	1	1	0.00	0.00	16	10	3UTR

mirnaid	refseqid	genesymbol	start	end	bindingp	seed	phylopstem	phylopflank	binding_region_length	longest_consecutive_pairings	position
hsa-miR-11400	NM_022484	TMEM168	5061	5086	1	1	3.79	4.03	25	8	3UTR
hsa-miR-11400	NM_020823	TMEM181	4875	4896	1	1	-0.04	0.32	21	10	3UTR
hsa-miR-11400	NM_138391	TMEM183A	2891	2917	1	1	0.79	0.11	18	8	3UTR
hsa-miR-11400	NM_018279	TMEM19	3988	4008	1	1	-0.11	-0.15	20	7	3UTR
hsa-miR-11400	NM_001100389	TMEM192	3668	3693	1	1	0.26	0.18	25	8	3UTR
hsa-miR-11400	NM_001363562	TMEM196	1463	1495	1	1	-0.02	0.19	23	9	3UTR
hsa-miR-11400	NM_001318217	TMEM208	741	788	1	1	0.40	2.58	16	14	3UTR
hsa-miR-11400	NM_014187	TMEM208	624	671	1	1	0.40	2.58	16	14	3UTR
hsa-miR-11400	XM_017001812	TMEM234	2436	2457	1	1	0.00	0.00	21	13	3UTR
hsa-miR-11400	XM_024448414	TMEM234	2312	2333	1	1	0.00	0.00	21	13	3UTR
hsa-miR-11400	NM_032933	TMEM241	2812	2832	1	1	2.25	2.76	20	7	3UTR
hsa-miR-11400	NM_032933	TMEM241	2610	2633	1	1	2.66	2.47	23	8	3UTR
hsa-miR-11400	XM_024448724	TMEM25	1821	1858	1	1	0.00	0.00	20	10	3UTR
hsa-miR-11400	NM_001270367	TMEM254	1088	1103	1	1	-0.10	-0.32	15	8	3UTR
hsa-miR-11400	NM_017938	TMEM255A	1885	1903	1	1	0.75	0.54	18	9	3UTR
hsa-miR-11400	NM_001104544	TMEM255A	1813	1831	1	1	0.70	0.74	18	9	3UTR
hsa-miR-11400	NM_001104545	TMEM255A	1561	1579	1	1	0.34	1.10	18	9	3UTR

mirnaid	refseqid	genesymbol	start	end	bindingp	seed	phylopstem	phylopflank	binding_region_length	longest_consecutive_pairings	position
hsa-miR-11400	NM_001351003	TMEM272	1412	1435	1	1	-0.20	-0.15	18	10	3UTR
hsa-miR-11400	NM_001288743	TMEM273	822	870	0.974359	1	-0.10	-0.12	19	13	3UTR
hsa-miR-11400	XM_005248116	TMEM33	1630	1665	1	1	0.00	0.00	19	8	3UTR
hsa-miR-11400	XM_005248117	TMEM33	1590	1608	1	1	0.00	0.00	18	8	3UTR
hsa-miR-11400	XM_011510659	TMEM37	947	986	1	1	0.00	0.00	39	10	3UTR
hsa-miR-11400	NM_001331211	TMEM45B	947	984	1	1	1.03	0.62	37	11	3UTR
hsa-miR-11400	NM_153022	TMEM52B	1593	1611	1	1	0.01	-0.10	18	12	3UTR
hsa-miR-11400	NM_001079815	TMEM52B	1527	1545	1	1	0.01	-0.10	18	12	3UTR
hsa-miR-11400	NM_001286660	TMEM68	2026	2044	1	1	2.16	2.34	18	7	3UTR
hsa-miR-11400	NM_001345926	TMEM72	1733	1752	1	1	-0.20	-0.45	19	8	3UTR
hsa-miR-11400	NM_001123376	TMEM72	1800	1819	1	1	-0.63	-0.56	19	8	3UTR
hsa-miR-11400	NM_015497	TMEM87A	2420	2459	0.961538	1	1.81	2.66	24	8	3UTR
hsa-miR-11400	NM_001286487	TMEM87A	2510	2549	1	1	3.01	1.94	24	8	3UTR
hsa-miR-11400	NM_001110503	TMEM87A	1619	1635	1	1	-0.02	-0.02	16	12	3UTR
hsa-miR-11400	NM_032824	TMEM87B	3096	3115	1	1	1.19	0.22	19	7	3UTR
hsa-miR-11400	NM_001329914	TMEM87B	3096	3115	1	1	1.19	0.22	19	7	3UTR
hsa-miR-11400	NM_001301746	TMEM98	2568	2586	1	1	0.00	0.00	18	9	3UTR

mirnaid	refseqid	genesymbol	start	end	bindingp	seed	phylopstem	phylopflank	binding_region_length	longest_consecutive_pairings	position
hsa-miR-11400	NM_001033504	TMEM98	2420	2438	1	1	0.00	0.00	18	9	3UTR
hsa-miR-11400	NM_182606	TMPRSS11A	2792	2807	1	1	0.56	1.17	15	14	3UTR
hsa-miR-11400	NM_001114387	TMPRSS11A	2783	2798	1	1	0.54	0.99	15	14	3UTR
hsa-miR-11400	NM_001290096	TMPRSS4	1683	1707	1	1	0.26	-0.01	19	8	3UTR
hsa-miR-11400	NM_019894	TMPRSS4	1768	1792	1	1	0.04	0.03	19	8	3UTR
hsa-miR-11400	NM_001083947	TMPRSS4	1753	1777	1	1	0.26	-0.01	19	8	3UTR
hsa-miR-11400	NM_001173551	TMPRSS4	1762	1786	1	1	0.26	-0.01	19	8	3UTR
hsa-miR-11400	NM_001173552	TMPRSS4	1648	1672	1	1	0.26	-0.01	19	8	3UTR
hsa-miR-11400	NM_152588	TMTC2	5256	5275	1	1	1.74	1.00	19	14	3UTR
hsa-miR-11400	NM_177441	TMUB2	1687	1712	1	1	0.44	0.63	25	10	3UTR
hsa-miR-11400	NM_024107	TMUB2	2051	2071	1	1	3.47	2.43	20	8	3UTR
hsa-miR-11400	NM_001330235	TMUB2	1344	1369	1	1	0.44	0.63	25	10	3UTR
hsa-miR-11400	NM_001353177	TMUB2	1324	1349	1	1	0.44	0.63	25	10	3UTR
hsa-miR-11400	NM_001353182	TMUB2	1257	1282	1	1	0.44	0.63	25	10	3UTR
hsa-miR-11400	NM_001076674	TMUB2	1265	1290	1	1	0.44	0.63	25	10	3UTR
hsa-miR-11400	NM_000594	TNF	1281	1298	1	1	-0.27	0.26	17	9	3UTR
hsa-miR-11400	XM_011537114	TNFAIP2	2874	2901	1	1	0.00	0.00	27	11	3UTR

mirnaid	refseqid	genesymbol	start	end	bindingp	seed	phylopstem	phylopflank	binding_region_length	longest_consecutive_pairings	position
hsa-miR-11400	NM_024575	TNFAIP8L2	956	1003	1	1	1.72	0.45	14	12	3UTR
hsa-miR-11400	NM_003840	TNFRSF10D	2262	2303	1	1	0.00	-0.24	17	15	3UTR
hsa-miR-11400	NM_001039664	TNFRSF25	628	650	1	1	0.44	0.01	22	8	3UTR
hsa-miR-11400	NM_001190943	TNFSF10	491	506	1	1	0.16	0.08	15	14	3UTR
hsa-miR-11400	NM_006573	TNFSF13B	1216	1233	1	1	1.09	1.49	17	7	3UTR
hsa-miR-11400	NM_001145645	TNFSF13B	1248	1265	1	1	1.09	1.49	17	7	3UTR
hsa-miR-11400	NM_001297562	TNFSF4	2712	2749	1	1	0.14	0.31	32	10	3UTR
hsa-miR-11400	NM_003326	TNFSF4	2798	2835	1	1	1.21	0.26	32	10	3UTR
hsa-miR-11400	NM_003281	TNNI1	3434	3474	1	1	0.34	0.24	40	10	3UTR
hsa-miR-11400	XM_017002219	TNR	7015	7035	1	1	0.00	0.00	20	12	3UTR
hsa-miR-11400	NM_003285	TNR	6629	6649	1	1	0.71	0.79	20	12	3UTR
hsa-miR-11400	NM_018996	TNRC6C	8502	8520	1	1	-0.43	-0.45	18	8	3UTR
hsa-miR-11400	NM_001142640	TNRC6C	8610	8628	1	1	-0.43	-0.45	18	8	3UTR
hsa-miR-11400	XM_017004813	TNS1	8489	8512	1	1	0.00	0.00	23	6	3UTR
hsa-miR-11400	NM_032865	TNS4	3412	3431	1	1	0.45	1.94	19	10	3UTR
hsa-miR-11400	NM_001267578	TOR1AIP1	2924	2949	1	1	-0.30	-0.03	25	8	3UTR
hsa-miR-11400	NM_015602	TOR1AIP1	2921	2946	1	1	-0.30	-0.03	25	8	3UTR

mirnaid	refseqid	genesymbol	start	end	bindingp	seed	phylopstem	phylopflank	binding_region_length	longest_consecutive_pairings	position
hsa-miR-11400	NM_032883	TOX2	2024	2045	1	1	-0.11	0.69	21	11	3UTR
hsa-miR-11400	NM_001098796	TOX2	1899	1920	1	1	-0.11	0.69	21	11	3UTR
hsa-miR-11400	NM_001098797	TOX2	1959	1980	1	1	-0.11	0.69	21	11	3UTR
hsa-miR-11400	NM_001098798	TOX2	1882	1903	1	1	-0.11	0.69	21	11	3UTR
hsa-miR-11400	NM_001251964	TP53AIP1	994	1008	1	1	-0.53	0.05	14	8	3UTR
hsa-miR-11400	NM_001258320	TP53I11	2054	2074	1	1	-0.24	-0.34	20	8	3UTR
hsa-miR-11400	NM_001258324	TP53I11	1910	1930	1	1	-0.41	-0.48	20	8	3UTR
hsa-miR-11400	NM_001204184	TP73	3174	3196	1	1	0.00	0.00	22	7	3UTR
hsa-miR-11400	NM_139075	TPCN2	4756	4774	0.980769	1	0.15	-0.49	18	11	3UTR
hsa-miR-11400	NM_139075	TPCN2	3472	3488	1	1	-0.16	-0.28	16	9	3UTR
hsa-miR-11400	NM_199359	TPD52L2	788	809	1	1	0.51	0.56	21	11	3UTR
hsa-miR-11400	NM_199360	TPD52L2	917	938	1	1	0.51	0.56	21	11	3UTR
hsa-miR-11400	NM_199361	TPD52L2	857	878	1	1	0.51	0.56	21	11	3UTR
hsa-miR-11400	NM_199362	TPD52L2	890	911	1	1	0.51	0.56	21	11	3UTR
hsa-miR-11400	NM_199363	TPD52L2	830	851	1	1	0.51	0.56	21	11	3UTR
hsa-miR-11400	NM_003288	TPD52L2	848	869	1	1	0.51	0.56	21	11	3UTR
hsa-miR-11400	NM_001243892	TPD52L2	719	740	1	1	0.51	0.56	21	11	3UTR

mirnaid	refseqid	genesymbol	start	end	bindingp	seed	phylopstem	phylopflank	binding_region_length	longest_consecutive_pairings	position
hsa-miR-11400	NM_001243894	TPD52L2	686	707	1	1	0.00	0.00	21	11	3UTR
hsa-miR-11400	NM_004179	TPH1	3612	3644	1	1	5.74	5.46	32	8	3UTR
hsa-miR-11400	NM_001301227	TPM2	1036	1073	0.974359	1	6.21	3.10	37	9	3UTR
hsa-miR-11400	NM_003289	TPM2	1036	1073	0.974359	1	6.49	2.50	37	9	3UTR
hsa-miR-11400	NM_001043352	TPM3	1841	1864	1	1	0.81	0.50	23	8	3UTR
hsa-miR-11400	NM_001043353	TPM3	1841	1864	1	1	0.81	0.50	23	8	3UTR
hsa-miR-11400	NM_003292	TPR	9396	9418	1	1	3.32	1.75	22	11	3UTR
hsa-miR-11400	NM_003292	TPR	8403	8423	1	1	2.38	3.02	20	10	3UTR
hsa-miR-11400	NM_001136053	TPRA1	3682	3700	1	1	0.33	-0.03	18	13	3UTR
hsa-miR-11400	NM_001142646	TPRA1	3518	3536	1	1	0.33	-0.03	18	13	3UTR
hsa-miR-11400	NM_001286272	TPT1	3657	3679	1	1	-0.01	1.35	22	6	3UTR
hsa-miR-11400	NM_001286273	TPT1	3418	3440	1	1	1.09	1.25	22	6	3UTR
hsa-miR-11400	NM_003295	TPT1	3492	3514	1	1	2.04	0.93	22	6	3UTR
hsa-miR-11400	NM_005658	TRAF1	3785	3829	1	1	1.70	1.25	44	12	3UTR
hsa-miR-11400	NM_001190945	TRAF1	3735	3779	1	1	1.21	1.15	44	12	3UTR
hsa-miR-11400	NM_001190947	TRAF1	3160	3204	1	1	2.81	2.84	44	12	3UTR
hsa-miR-11400	NM_004620	TRAF6	5069	5089	1	1	0.01	0.28	20	15	3UTR

mirnaid	refseqid	genesymbol	start	end	bindingp	seed	phylopstem	phylopflank	binding_region_length	longest_consecutive_pairings	position
hsa-miR-11400	NM_004620	TRAF6	2663	2679	1	1	-0.01	-0.09	16	15	3UTR
hsa-miR-11400	NM_001265609	TRAK1	2408	2423	0.980769	1	1.02	0.83	15	14	3UTR
hsa-miR-11400	NM_014965	TRAK1	2324	2339	0.980769	1	1.02	0.83	15	14	3UTR
hsa-miR-11400	NM_015049	TRAK2	3364	3389	1	1	2.61	2.58	25	12	3UTR
hsa-miR-11400	NM_003274	TRAPPC10	4081	4111	1	1	0.82	0.40	30	10	3UTR
hsa-miR-11400	NM_177452	TRAPPC6B	851	871	1	1	-0.27	0.23	20	7	3UTR
hsa-miR-11400	NM_001079537	TRAPPC6B	935	955	1	1	-0.11	0.16	20	7	3UTR
hsa-miR-11400	NM_198153	TREML4	2077	2095	1	1	-0.17	-0.37	18	10	3UTR
hsa-miR-11400	NM_013381	TRHDE	7632	7678	1	1	-0.69	-0.01	18	16	3UTR
hsa-miR-11400	NM_052828	TRIM10	2624	2641	0.974359	1	1.41	0.48	17	14	3UTR
hsa-miR-11400	NM_006778	TRIM10	3163	3180	0.974359	1	1.52	0.58	17	14	3UTR
hsa-miR-11400	XM_006714158	TRIM2	5370	5392	1	1	0.00	0.00	22	7	3UTR
hsa-miR-11400	XM_006714160	TRIM2	6170	6192	1	1	0.00	0.00	22	7	3UTR
hsa-miR-11400	XM_006714161	TRIM2	6167	6189	1	1	0.00	0.00	22	7	3UTR
hsa-miR-11400	XM_017007944	TRIM2	6158	6180	1	1	0.00	0.00	22	7	3UTR
hsa-miR-11400	XM_017007946	TRIM2	5475	5497	1	1	0.00	0.00	22	7	3UTR
hsa-miR-11400	XM_017007947	TRIM2	5472	5494	1	1	0.00	0.00	22	7	3UTR

mirnaid	refseqid	genesymbol	start	end	bindingp	seed	phylopstem	phylopflank	binding_region_length	longest_consecutive_pairings	position
hsa-miR-11400	XM_017007948	TRIM2	5425	5447	1	1	0.00	0.00	22	7	3UTR
hsa-miR-11400	XM_017007950	TRIM2	5878	5900	1	1	0.00	0.00	22	7	3UTR
hsa-miR-11400	NM_015271	TRIM2	3575	3596	1	1	0.66	1.79	21	12	3UTR
hsa-miR-11400	NM_001351054	TRIM2	3698	3719	1	1	0.66	1.79	21	12	3UTR
hsa-miR-11400	NM_001351055	TRIM2	3695	3716	1	1	0.66	1.79	21	12	3UTR
hsa-miR-11400	NM_001351056	TRIM2	3745	3766	1	1	0.66	1.79	21	12	3UTR
hsa-miR-11400	NM_001375488	TRIM2	3668	3689	1	1	0.66	1.79	21	12	3UTR
hsa-miR-11400	NM_001375489	TRIM2	3665	3686	1	1	0.66	1.79	21	12	3UTR
hsa-miR-11400	NM_001375490	TRIM2	3518	3539	1	1	0.66	1.79	21	12	3UTR
hsa-miR-11400	NM_001375491	TRIM2	3515	3536	1	1	0.66	1.79	21	12	3UTR
hsa-miR-11400	NM_001375512	TRIM2	4369	4390	1	1	0.66	1.79	21	12	3UTR
hsa-miR-11400	NM_001375513	TRIM2	3733	3754	1	1	0.66	1.79	21	12	3UTR
hsa-miR-11400	NM_001375514	TRIM2	3738	3759	1	1	0.66	1.79	21	12	3UTR
hsa-miR-11400	NM_001375515	TRIM2	3650	3671	1	1	0.66	1.79	21	12	3UTR
hsa-miR-11400	NM_001375516	TRIM2	3660	3681	1	1	0.66	1.79	21	12	3UTR
hsa-miR-11400	NM_001375517	TRIM2	3574	3595	1	1	0.66	1.79	21	12	3UTR
hsa-miR-11400	NM_001375519	TRIM2	3242	3263	1	1	0.66	1.79	21	12	3UTR

mirnaid	refseqid	genesymbol	start	end	bindingp	seed	phylopstem	phylopflank	binding_region_length	longest_consecutive_pairings	position
hsa-miR-11400	NM_001375520	TRIM2	3239	3260	1	1	0.66	1.79	21	12	3UTR
hsa-miR-11400	NM_001375522	TRIM2	3522	3543	1	1	0.66	1.79	21	12	3UTR
hsa-miR-11400	NM_001375525	TRIM2	3465	3486	1	1	0.66	1.79	21	12	3UTR
hsa-miR-11400	NM_001130067	TRIM2	3647	3668	1	1	0.66	1.79	21	12	3UTR
hsa-miR-11400	NM_015905	TRIM24	6624	6644	1	1	-0.55	-0.03	20	15	3UTR
hsa-miR-11400	NM_003852	TRIM24	6522	6542	1	1	-0.55	-0.03	20	15	3UTR
hsa-miR-11400	XM_005249374	TRIM26	2661	2681	1	1	0.00	0.00	20	10	3UTR
hsa-miR-11400	NM_003449	TRIM26	2777	2797	1	1	0.62	1.09	20	10	3UTR
hsa-miR-11400	NM_001242783	TRIM26	2556	2576	1	1	0.00	0.00	20	10	3UTR
hsa-miR-11400	NM_001330382	TRIM29	1604	1625	1	1	3.77	2.74	21	8	3UTR
hsa-miR-11400	NM_012210	TRIM32	2688	2704	1	1	0.00	0.39	16	11	3UTR
hsa-miR-11400	NM_001099679	TRIM32	2685	2701	1	1	0.00	0.39	16	11	3UTR
hsa-miR-11400	NM_172016	TRIM39	2243	2285	1	1	0.97	0.21	20	14	3UTR
hsa-miR-11400	NM_021253	TRIM39	2394	2436	1	1	0.87	0.18	20	14	3UTR
hsa-miR-11400	NM_001369521	TRIM39	2304	2346	1	1	0.87	0.18	20	14	3UTR
hsa-miR-11400	NM_001369523	TRIM39	2349	2391	1	1	0.88	0.18	20	14	3UTR
hsa-miR-11400	NM_033091	TRIM4	1820	1842	1	1	0.74	0.55	22	6	3UTR

mirnaid	refseqid	genesymbol	start	end	bindingp	seed	phylopstem	phylopflank	binding_region_length	longest_consecutive_pairings	position
hsa-miR-11400	NM_033452	TRIM47	2157	2177	1	1	1.94	1.76	20	9	3UTR
hsa-miR-11400	XM_011544192	TRIM67	8051	8077	1	1	0.00	0.00	26	9	3UTR
hsa-miR-11400	NM_030912	TRIM8	1988	2009	1	1	1.14	1.08	21	17	3UTR
hsa-miR-11400	XM_011536389	TRIM9	5682	5704	1	1	0.00	0.00	22	11	3UTR
hsa-miR-11400	NM_007032	TRIOBP	4483	4503	1	1	0.00	0.00	20	10	3UTR
hsa-miR-11400	NM_001039141	TRIOBP	9829	9849	1	1	-0.26	-0.41	20	10	3UTR
hsa-miR-11400	NM_004239	TRIP11	7504	7529	1	1	2.15	2.06	25	8	3UTR
hsa-miR-11400	NM_182984	TRMT2A	2134	2175	0.980769	1	2.63	2.65	23	8	3UTR
hsa-miR-11400	NM_012471	TRPC5	7561	7580	1	1	0.00	0.00	19	10	3UTR
hsa-miR-11400	NM_019841	TRPV5	2860	2879	1	1	-0.48	-0.09	19	11	3UTR
hsa-miR-11400	NM_015679	TRUB2	4312	4336	1	1	-0.03	-0.17	24	12	3UTR
hsa-miR-11400	NM_021055	TSC2	5586	5627	1	1	0.00	0.00	22	8	3UTR
hsa-miR-11400	NM_001318827	TSC2	5406	5447	1	1	0.00	0.00	22	8	3UTR
hsa-miR-11400	NM_001318829	TSC2	5350	5391	1	1	0.00	0.00	22	8	3UTR
hsa-miR-11400	NM_001318832	TSC2	5505	5546	1	1	0.00	0.00	22	8	3UTR
hsa-miR-11400	NM_001363528	TSC2	5517	5558	1	1	0.00	0.00	22	8	3UTR
hsa-miR-11400	NM_001077183	TSC2	5514	5555	1	1	0.00	0.00	22	8	3UTR

mirnaid	refseqid	genesymbol	start	end	bindingp	seed	phylopstem	phylopflank	binding_region_length	longest_consecutive_pairings	position
hsa-miR-11400	NM_001114382	TSC2	5646	5687	1	1	0.00	0.00	22	8	3UTR
hsa-miR-11400	XM_005262100	TSC22D3	1284	1362	1	1	0.00	0.00	22	10	3UTR
hsa-miR-11400	NM_001318468	TSC22D3	1395	1473	1	1	0.00	0.00	22	10	3UTR
hsa-miR-11400	NM_001318470	TSC22D3	1381	1459	1	1	0.00	0.00	22	10	3UTR
hsa-miR-11400	NM_173485	TSHZ2	7416	7436	1	1	0.36	0.97	20	9	3UTR
hsa-miR-11400	NM_001193421	TSHZ2	6834	6854	1	1	0.36	0.97	20	9	3UTR
hsa-miR-11400	NM_130783	TSPAN18	1742	1760	1	1	-0.20	-0.41	18	10	3UTR
hsa-miR-11400	XM_006718373	TSPAN18	1673	1691	1	1	0.00	0.00	18	10	3UTR
hsa-miR-11400	XM_011520459	TSPAN18	1574	1592	1	1	0.00	0.00	18	10	3UTR
hsa-miR-11400	NM_178562	TSPAN33	1273	1293	1	1	-0.12	0.27	20	13	3UTR
hsa-miR-11400	XM_017011367	TTBK1	2892	2917	1	1	0.00	0.00	25	7	3UTR
hsa-miR-11400	XM_017011367	TTBK1	5111	5131	1	1	0.00	0.00	20	8	3UTR
hsa-miR-11400	NM_001114108	TTC22	2368	2386	1	1	4.24	3.29	18	7	3UTR
hsa-miR-11400	NM_001297662	TTC39A	1695	1720	1	1	4.13	2.43	25	8	3UTR
hsa-miR-11400	NM_001168342	TTC39B	3382	3401	1	1	-0.05	-0.37	19	18	3UTR
hsa-miR-11400	XM_011533000	TTC7A	2290	2314	1	1	0.00	0.00	24	8	3UTR
hsa-miR-11400	XM_011533000	TTC7A	2787	2806	1	1	0.00	0.00	19	11	3UTR

mirnaid	refseqid	genesymbol	start	end	bindingp	seed	phylopstem	phylopflank	binding_region_length	longest_consecutive_pairings	position
hsa-miR-11400	XM_024453013	TTC7A	1893	1917	1	1	0.00	0.00	24	8	3UTR
hsa-miR-11400	XM_024453013	TTC7A	2390	2409	1	1	0.00	0.00	19	11	3UTR
hsa-miR-11400	NM_001010854	TTC7B	7665	7683	1	1	-0.24	-0.07	18	10	3UTR
hsa-miR-11400	NM_001139442	TTLL11	3594	3631	1	1	0.05	-0.42	37	10	3UTR
hsa-miR-11400	NM_001367620	TTLL9	1754	1773	1	1	0.09	0.05	19	9	3UTR
hsa-miR-11400	NM_001008409	TTLL9	2303	2322	1	1	0.09	0.05	19	9	3UTR
hsa-miR-11400	NM_001261839	TTPAL	5960	5989	1	1	0.80	1.44	21	16	3UTR
hsa-miR-11400	NM_024331	TTPAL	6086	6115	1	1	0.80	1.44	21	16	3UTR
hsa-miR-11400	NM_001039199	TTPAL	6062	6091	1	1	0.80	1.44	21	16	3UTR
hsa-miR-11400	NM_001286414	TUBGCP4	5017	5036	1	1	3.38	2.67	19	8	3UTR
hsa-miR-11400	NM_014444	TUBGCP4	5014	5033	1	1	3.38	2.67	19	8	3UTR
hsa-miR-11400	NM_052903	TUBGCP5	3213	3236	0.953846	1	4.64	3.26	23	9	3UTR
hsa-miR-11400	XM_017013861	TUSC3	3579	3600	1	1	0.00	0.00	21	12	3UTR
hsa-miR-11400	XM_006720944	TVP23A	1092	1127	1	1	0.00	0.00	19	17	3UTR
hsa-miR-11400	XM_017023651	TVP23A	1410	1428	1	1	0.00	0.00	18	7	3UTR
hsa-miR-11400	NM_175852	TXLNA	3787	3806	1	1	-0.10	0.14	19	15	3UTR
hsa-miR-11400	NM_001376857	TXLNA	3934	3953	1	1	-0.10	0.14	19	15	3UTR

mirnaid	refseqid	genesymbol	start	end	bindingp	seed	phylopstem	phylopflank	binding_region_length	longest_consecutive_pairings	position
hsa-miR-11400	NM_153235	TXLNB	4013	4030	1	1	4.20	3.21	17	10	3UTR
hsa-miR-11400	NM_032731	TXNDC17	917	957	1	1	-0.06	-0.18	17	15	3UTR
hsa-miR-11400	NM_001313972	TXNIP	2323	2356	1	1	1.36	2.09	33	10	3UTR
hsa-miR-11400	NM_001305563	TXNL4A	1936	1957	1	1	-0.08	-0.16	21	9	3UTR
hsa-miR-11400	NM_006701	TXNL4A	2117	2138	1	1	-0.18	-0.18	21	9	3UTR
hsa-miR-11400	NM_001330264	TYRO3	5033	5058	1	1	0.00	0.00	25	8	3UTR
hsa-miR-11400	NM_138467	TYW3	1728	1746	0.980769	1	-0.20	-0.12	18	11	3UTR
hsa-miR-11400	NM_001162916	TYW3	1629	1647	0.980769	1	-0.20	-0.12	18	11	3UTR
hsa-miR-11400	NM_001039693	TYW5	3883	3902	1	1	0.39	0.50	19	9	3UTR
hsa-miR-11400	NM_001039693	TYW5	2814	2848	1	1	0.69	0.19	34	10	3UTR
hsa-miR-11400	NM_024818	UBA5	1809	1840	1	1	-0.08	-0.04	31	10	3UTR
hsa-miR-11400	NM_001320210	UBA5	2159	2190	1	1	0.21	0.06	31	10	3UTR
hsa-miR-11400	NM_018227	UBA6	5494	5513	1	1	0.70	0.80	19	12	3UTR
hsa-miR-11400	NM_016172	UBAC1	1542	1560	0.961538	1	2.61	2.93	18	9	3UTR
hsa-miR-11400	XM_005265431	UBE2E1	1348	1375	1	1	0.00	0.00	21	12	3UTR
hsa-miR-11400	NM_001202476	UBE2E1	1361	1372	1	1	0.60	-1.38	11	10	3UTR
hsa-miR-11400	XM_024450432	UBE2I	1831	1867	1	1	0.00	0.00	18	8	3UTR

mirnaid	refseqid	genesymbol	start	end	bindingp	seed	phylopstem	phylopflank	binding_region_length	longest_consecutive_pairings	position
hsa-miR-11400	NM_152489	UBE2U	2872	2893	0.980769	1	0.68	-0.11	21	11	3UTR
hsa-miR-11400	NM_199144	UBE2V1	2054	2099	1	1	2.20	2.99	38	8	3UTR
hsa-miR-11400	NM_003350	UBE2V2	3573	3590	1	1	0.16	0.24	17	16	3UTR
hsa-miR-11400	NM_018299	UBE2W	1154	1176	1	1	0.88	0.29	22	9	3UTR
hsa-miR-11400	NM_001001481	UBE2W	1187	1209	1	1	0.95	0.25	22	9	3UTR
hsa-miR-11400	XM_005263422	UBE4B	5798	5817	0.974359	1	0.00	0.00	19	8	3UTR
hsa-miR-11400	NM_001330350	UBIAD1	3310	3326	1	1	-0.16	-0.07	16	15	3UTR
hsa-miR-11400	NM_203412	UBL4B	1434	1451	1	1	-0.14	-0.26	17	8	3UTR
hsa-miR-11400	NM_199415	UBOX5	3512	3537	1	1	4.25	2.11	25	8	3UTR
hsa-miR-11400	NM_014948	UBOX5	3674	3699	1	1	4.25	2.11	25	8	3UTR
hsa-miR-11400	NM_024954	UBTD1	1471	1503	1	1	0.98	0.37	24	8	3UTR
hsa-miR-11400	XM_006722059	UBTF	4466	4489	1	1	0.00	0.00	23	8	3UTR
hsa-miR-11400	NM_014233	UBTF	4577	4600	1	1	0.08	0.47	23	8	3UTR
hsa-miR-11400	NM_001076684	UBTF	4466	4489	1	1	0.08	0.47	23	8	3UTR
hsa-miR-11400	NM_152376	UBXN10	4025	4048	1	1	-0.14	-0.03	23	8	3UTR
hsa-miR-11400	NM_004181	UCHL1	858	900	1	1	0.42	0.77	18	16	3UTR
hsa-miR-11400	NM_003356	UCP3	2042	2062	1	1	2.00	2.73	20	16	3UTR

mirnaid	refseqid	genesymbol	start	end	bindingp	seed	phylopstem	phylopflank	binding_region_length	longest_consecutive_pairings	position
hsa-miR-11400	NM_003360	UGT8	3437	3453	1	1	0.43	0.47	16	15	3UTR
hsa-miR-11400	NM_001128174	UGT8	3093	3109	1	1	0.43	0.47	16	15	3UTR
hsa-miR-11400	NM_144624	UHMK1	5221	5239	1	1	0.27	0.34	18	17	3UTR
hsa-miR-11400	NM_175866	UHMK1	5297	5315	1	1	0.27	0.34	18	17	3UTR
hsa-miR-11400	NM_001184763	UHMK1	4962	4987	1	1	0.42	0.32	19	17	3UTR
hsa-miR-11400	NM_001006947	UHRF1BP1L	1850	1877	1	1	0.41	0.53	27	10	3UTR
hsa-miR-11400	NM_152896	UHRF2	3116	3148	1	1	1.46	2.07	32	8	3UTR
hsa-miR-11400	NM_001080533	UNC119B	826	848	1	1	-0.22	0.47	22	11	3UTR
hsa-miR-11400	XM_011527811	UNC13A	8527	8550	1	1	0.00	0.00	23	10	3UTR
hsa-miR-11400	NM_001080421	UNC13A	8451	8474	1	1	3.35	3.33	23	10	3UTR
hsa-miR-11400	NM_001297549	UPF1	4704	4727	1	1	-0.53	-0.34	23	10	3UTR
hsa-miR-11400	NM_002911	UPF1	4671	4694	1	1	-0.53	-0.34	23	10	3UTR
hsa-miR-11400	NM_013387	UQCR10	609	630	1	1	0.25	0.55	17	8	3UTR
hsa-miR-11400	NM_001003684	UQCR10	668	689	1	1	0.25	0.55	17	8	3UTR
hsa-miR-11400	NM_003365	UQCRC1	1486	1505	1	1	1.91	1.06	19	11	3UTR
hsa-miR-11400	NM_001290075	URGCP	3000	3032	1	1	3.13	1.61	32	8	3UTR
hsa-miR-11400	NM_017920	URGCP	2978	3010	1	1	2.57	2.12	32	8	3UTR

mirnaid	refseqid	genesymbol	start	end	bindingp	seed	phylopstem	phylopflank	binding_region_length	longest_consecutive_pairings	position
hsa-miR-11400	XM_005270140	UROS	2320	2354	1	1	0.00	0.00	18	16	3UTR
hsa-miR-11400	XM_005270140	UROS	2566	2584	1	1	0.00	0.00	18	10	3UTR
hsa-miR-11400	NM_024598	USB1	2083	2100	1	1	-0.56	-0.03	17	16	3UTR
hsa-miR-11400	NM_001330568	USB1	1989	2006	1	1	-0.56	-0.03	17	16	3UTR
hsa-miR-11400	NM_001195302	USB1	2029	2046	1	1	-0.56	-0.03	17	16	3UTR
hsa-miR-11400	XM_005259197	USF2	1288	1310	1	1	0.00	0.00	22	9	3UTR
hsa-miR-11400	NM_182488	USP12	1494	1537	1	1	1.40	2.09	16	14	3UTR
hsa-miR-11400	NM_005151	USP14	4834	4852	0.961538	1	0.81	1.33	18	9	3UTR
hsa-miR-11400	NM_001037334	USP14	4729	4747	0.961538	1	0.81	1.33	18	9	3UTR
hsa-miR-11400	NM_005151	USP14	3121	3138	1	1	-0.07	0.12	17	16	3UTR
hsa-miR-11400	NM_001037334	USP14	3016	3033	1	1	-0.07	0.12	17	16	3UTR
hsa-miR-11400	NM_006313	USP15	12415	12435	1	1	-0.09	0.04	20	14	3UTR
hsa-miR-11400	NM_006313	USP15	10482	10500	1	1	0.21	0.03	18	9	3UTR
hsa-miR-11400	NM_001252078	USP15	12502	12522	1	1	-0.09	0.04	20	14	3UTR
hsa-miR-11400	NM_001252078	USP15	10569	10587	1	1	0.21	0.03	18	9	3UTR
hsa-miR-11400	NM_022832	USP46	6480	6506	1	1	1.56	1.31	26	9	3UTR
hsa-miR-11400	NM_001384542	USP49	2569	2586	1	1	0.33	0.42	17	8	3UTR

mirnaid	refseqid	genesymbol	start	end	bindingp	seed	phylopstem	phylopflank	binding_region_length	longest_consecutive_pairings	position
hsa-miR-11400	NM_001080491	USP6NL	10307	10329	1	1	0.00	0.00	22	9	3UTR
hsa-miR-11400	NM_014388	UTP25	6459	6497	1	1	-0.32	0.00	38	9	3UTR
hsa-miR-11400	NM_003762	VAMP4	1045	1060	1	1	0.31	0.04	15	11	3UTR
hsa-miR-11400	NM_001185127	VAMP4	1042	1057	1	1	0.02	0.07	15	11	3UTR
hsa-miR-11400	NM_001287044	VEGFA	722	744	1	1	0.25	1.89	22	8	3UTR
hsa-miR-11400	NM_001128220	VGLL4	2056	2082	1	1	0.14	2.27	26	13	3UTR
hsa-miR-11400	NM_001128221	VGLL4	2179	2205	1	1	0.36	2.87	26	13	3UTR
hsa-miR-11400	NM_004624	VIPR1	1612	1635	1	1	-0.72	-0.41	23	13	3UTR
hsa-miR-11400	NM_001251882	VIPR1	2020	2043	1	1	0.00	0.00	23	13	3UTR
hsa-miR-11400	NM_001251883	VIPR1	1249	1272	1	1	-0.68	-0.46	23	13	3UTR
hsa-miR-11400	NM_001251884	VIPR1	1503	1526	1	1	-0.68	-0.46	23	13	3UTR
hsa-miR-11400	NM_001128159	VPS53	10823	10844	1	1	0.00	0.00	21	8	3UTR
hsa-miR-11400	NM_001128159	VPS53	10685	10706	1	1	0.00	0.00	21	8	3UTR
hsa-miR-11400	NM_001288838	VRK2	2107	2126	1	1	0.86	0.49	19	15	3UTR
hsa-miR-11400	NM_007268	VSIG4	1640	1657	1	1	0.06	1.13	17	13	3UTR
hsa-miR-11400	NM_001100431	VSIG4	1358	1375	1	1	0.06	1.13	17	13	3UTR
hsa-miR-11400	NM_022153	VSIR	1412	1431	1	1	0.95	1.17	19	15	3UTR

mirnaid	refseqid	genesymbol	start	end	bindingp	seed	phylopstem	phylopflank	binding_region_length	longest_consecutive_pairings	position
hsa-miR-11400	NM_001253849	VTCN1	2221	2236	1	1	2.12	2.23	15	14	3UTR
hsa-miR-11400	NM_001253850	VTCN1	1758	1773	1	1	0.81	1.67	15	14	3UTR
hsa-miR-11400	NM_024626	VTCN1	2106	2121	1	1	1.87	2.37	15	14	3UTR
hsa-miR-11400	NM_006370	VTI1B	1582	1600	1	1	0.29	0.18	18	12	3UTR
hsa-miR-11400	NM_001039500	VWA5B1	4274	4294	1	1	-0.54	-0.21	20	9	3UTR
hsa-miR-11400	NM_198570	VWC2	5463	5481	1	1	-0.19	-0.29	18	7	3UTR
hsa-miR-11400	NM_182905	WASHC1	1591	1607	1	1	2.44	3.25	16	15	3UTR
hsa-miR-11400	NM_007187	WBP4	1564	1593	1	1	1.95	0.93	20	9	3UTR
hsa-miR-11400	NM_052950	WDFY2	5907	5928	1	1	0.01	-0.30	21	9	3UTR
hsa-miR-11400	NM_001242414	WDR20	878	907	1	1	0.15	-0.08	29	9	3UTR
hsa-miR-11400	NM_025160	WDR26	4794	4816	1	1	4.59	6.05	22	8	3UTR
hsa-miR-11400	NM_025160	WDR26	3440	3456	1	1	1.59	1.47	16	11	3UTR
hsa-miR-11400	NM_001379403	WDR26	4794	4816	1	1	4.59	6.05	22	8	3UTR
hsa-miR-11400	NM_001379403	WDR26	3440	3456	1	1	1.59	1.47	16	11	3UTR
hsa-miR-11400	NM_001006622	WDR33	1739	1761	1	1	2.59	2.27	22	11	3UTR
hsa-miR-11400	NM_172005	WFDC13	615	634	1	1	0.17	0.33	19	10	3UTR
hsa-miR-11400	NM_024911	WLS	2439	2458	1	1	3.84	4.55	19	11	3UTR

mirnaid	refseqid	genesymbol	start	end	bindingp	seed	phylopstem	phylopflank	binding_region_length	longest_consecutive_pairings	position
hsa-miR-11400	NM_001193334	WLS	2189	2208	1	1	6.24	2.75	19	11	3UTR
hsa-miR-11400	NM_024494	WNT2B	2556	2577	1	1	0.24	0.37	21	11	3UTR
hsa-miR-11400	NM_030753	WNT3	1232	1254	1	1	-0.46	0.20	22	8	3UTR
hsa-miR-11400	NM_030761	WNT4	3692	3713	0.980769	1	5.04	2.11	21	8	3UTR
hsa-miR-11400	NM_001256105	WNT5A	4323	4344	1	1	2.53	1.61	21	7	3UTR
hsa-miR-11400	NM_001256105	WNT5A	2451	2469	1	1	0.59	0.33	18	10	3UTR
hsa-miR-11400	NM_003392	WNT5A	2710	2728	1	1	0.08	0.07	18	10	3UTR
hsa-miR-11400	NM_003396	WNT9B	2444	2466	1	1	-0.57	-0.14	20	12	3UTR
hsa-miR-11400	NM_001320458	WNT9B	2007	2055	1	1	-0.12	0.03	23	11	3UTR
hsa-miR-11400	NM_001270453	WWP2	3444	3463	1	1	-0.40	-0.61	19	18	3UTR
hsa-miR-11400	NM_001270454	WWP2	3706	3725	1	1	-0.40	-0.61	19	18	3UTR
hsa-miR-11400	NM_199424	WWP2	2843	2862	1	1	-0.40	-0.61	19	18	3UTR
hsa-miR-11400	NM_001024644	XCR1	4238	4257	1	1	1.69	2.42	19	9	3UTR
hsa-miR-11400	XM_011533097	XPO1	3293	3309	1	1	0.00	0.00	16	7	3UTR
hsa-miR-11400	NM_005431	XRCC2	2729	2753	1	1	0.03	0.08	24	9	3UTR
hsa-miR-11400	XM_017005750	XXYLT1	6240	6259	1	1	0.00	0.00	19	10	3UTR
hsa-miR-11400	NM_001190980	YAF2	517	537	1	1	-1.27	0.22	20	6	3UTR

mirnaid	refseqid	genesymbol	start	end	bindingp	seed	phylopstem	phylopflank	binding_region_length	longest_consecutive_pairings	position
hsa-miR-11400	NM_006555	YKT6	1374	1391	1	1	-0.52	-0.52	17	11	3UTR
hsa-miR-11400	NM_001363678	YKT6	1272	1289	1	1	-0.52	-0.52	17	11	3UTR
hsa-miR-11400	XM_017024621	YPEL2	3238	3271	1	1	0.00	0.00	24	9	3UTR
hsa-miR-11400	XM_017024621	YPEL2	2355	2374	1	1	0.00	0.00	19	6	3UTR
hsa-miR-11400	NM_001005404	YPEL2	3257	3290	1	1	0.00	0.00	24	9	3UTR
hsa-miR-11400	NM_133370	YTHDC1	3421	3437	1	1	3.02	1.65	16	15	3UTR
hsa-miR-11400	NM_001330698	YTHDC1	3499	3515	1	1	1.44	2.12	16	15	3UTR
hsa-miR-11400	NM_001031732	YTHDC1	3475	3491	1	1	1.84	2.02	16	15	3UTR
hsa-miR-11400	NM_012479	YWHAG	1855	1886	1	1	1.14	1.95	21	7	3UTR
hsa-miR-11400	NM_014838	ZBED4	4654	4675	1	1	0.83	0.59	21	9	3UTR
hsa-miR-11400	XM_017021095	ZBTB1	3686	3704	1	1	0.00	0.00	18	9	3UTR
hsa-miR-11400	NM_006006	ZBTB16	6246	6264	1	1	0.14	-0.18	18	9	3UTR
hsa-miR-11400	NM_001018011	ZBTB16	6152	6170	1	1	0.14	-0.18	18	9	3UTR
hsa-miR-11400	NM_205768	ZBTB18	3160	3180	1	1	3.38	2.83	20	7	3UTR
hsa-miR-11400	NM_001098402	ZBTB21	6438	6458	1	1	1.47	1.48	20	12	3UTR
hsa-miR-11400	NM_001098403	ZBTB21	5835	5855	1	1	1.47	1.48	20	12	3UTR
hsa-miR-11400	NM_001376164	ZBTB38	4897	4913	1	1	0.89	0.21	16	8	3UTR

mirnaid	refseqid	genesymbol	start	end	bindingp	seed	phylopstem	phylopflank	binding_region_length	longest_consecutive_pairings	position
hsa-miR-11400	NM_001376166	ZBTB38	4904	4920	1	1	0.89	0.21	16	8	3UTR
hsa-miR-11400	NM_001376179	ZBTB38	5175	5191	1	1	0.89	0.21	16	8	3UTR
hsa-miR-11400	NM_014870	ZBTB40	7934	7951	0.961538	1	0.23	0.28	17	13	3UTR
hsa-miR-11400	NM_001330398	ZBTB40	7598	7615	0.961538	1	0.23	0.28	17	13	3UTR
hsa-miR-11400	NM_001083621	ZBTB40	8222	8239	0.961538	1	0.23	0.28	17	13	3UTR
hsa-miR-11400	NM_014007	ZBTB43	1706	1726	1	1	3.02	2.26	20	8	3UTR
hsa-miR-11400	XM_005260198	ZBTB46	3411	3429	0.991453	1	0.00	0.00	18	8	3UTR
hsa-miR-11400	NM_152735	ZBTB9	2603	2621	1	1	0.04	0.11	18	6	3UTR
hsa-miR-11400	NM_001010888	ZC3H12B	6704	6726	1	1	0.00	0.00	22	10	3UTR
hsa-miR-11400	NM_207660	ZC3H14	9308	9327	1	1	-0.12	-0.13	19	10	3UTR
hsa-miR-11400	NM_207661	ZC3H14	9362	9381	1	1	0.00	0.00	19	10	3UTR
hsa-miR-11400	NM_207661	ZC3H14	6947	6964	1	1	0.00	0.00	17	16	3UTR
hsa-miR-11400	NM_207662	ZC3H14	8699	8718	1	1	-0.12	-0.13	19	10	3UTR
hsa-miR-11400	NM_024824	ZC3H14	9779	9798	1	1	-0.12	-0.13	19	10	3UTR
hsa-miR-11400	NM_001326295	ZC3H14	9386	9405	1	1	-0.12	-0.13	19	10	3UTR
hsa-miR-11400	NM_001326307	ZC3H14	9704	9723	1	1	-0.12	-0.13	19	10	3UTR
hsa-miR-11400	NM_001326311	ZC3H14	9193	9212	1	1	-0.12	-0.13	19	10	3UTR

mirnaid	refseqid	genesymbol	start	end	bindingp	seed	phylopstem	phylopflank	binding_region_length	longest_consecutive_pairings	position
hsa-miR-11400	NM_001160104	ZC3H14	9761	9780	1	1	-0.12	-0.13	19	10	3UTR
hsa-miR-11400	NM_001294340	ZC3H18	3145	3165	1	1	1.96	2.22	20	8	3UTR
hsa-miR-11400	NM_144604	ZC3H18	3073	3093	1	1	1.96	2.22	20	8	3UTR
hsa-miR-11400	XM_017026531	ZC3H4	5280	5298	1	1	0.00	0.00	18	9	3UTR
hsa-miR-11400	NM_015168	ZC3H4	5523	5541	1	1	3.34	2.89	18	9	3UTR
hsa-miR-11400	NM_198581	ZC3H6	11335	11365	1	1	0.49	0.22	30	10	3UTR
hsa-miR-11400	NM_018684	ZC4H2	2597	2618	1	1	4.49	4.20	21	10	3UTR
hsa-miR-11400	NM_001178032	ZC4H2	2839	2860	1	1	0.05	0.12	21	10	3UTR
hsa-miR-11400	NM_001178033	ZC4H2	2434	2455	1	1	4.53	4.61	21	10	3UTR
hsa-miR-11400	NM_001300817	ZCCHC10	1600	1622	0.961538	1	3.21	1.47	22	12	3UTR
hsa-miR-11400	NM_001300819	ZCCHC10	1510	1532	0.961538	1	0.49	0.95	22	12	3UTR
hsa-miR-11400	NM_001300822	ZCCHC10	1646	1668	0.961538	1	2.80	3.28	22	12	3UTR
hsa-miR-11400	NM_001308130	ZCCHC10	1580	1602	0.961538	1	0.75	1.89	22	12	3UTR
hsa-miR-11400	NM_017665	ZCCHC10	1552	1574	0.961538	1	0.04	1.35	22	12	3UTR
hsa-miR-11400	XM_017005759	ZCWPW2	4458	4483	1	1	0.00	0.00	25	12	3UTR
hsa-miR-11400	NM_001330059	ZDHHC20	2018	2050	1	1	0.28	0.13	23	9	3UTR
hsa-miR-11400	NM_174976	ZDHHC22	2064	2083	1	1	0.53	-0.01	19	7	3UTR

mirnaid	refseqid	genesymbol	start	end	bindingp	seed	phylopstem	phylopflank	binding_region_length	longest_consecutive_pairings	position
hsa-miR-11400	NM_001349377	ZDHHC3	3477	3498	1	1	0.00	0.00	21	11	3UTR
hsa-miR-11400	NM_001135179	ZDHHC3	12181	12202	1	1	0.00	0.00	21	11	3UTR
hsa-miR-11400	NM_001135179	ZDHHC3	5973	6006	1	1	0.00	0.00	33	8	3UTR
hsa-miR-11400	XM_017016564	ZDHHC6	2060	2103	1	1	0.00	0.00	43	10	3UTR
hsa-miR-11400	NM_006336	ZER1	4029	4050	1	1	0.35	0.43	21	8	3UTR
hsa-miR-11400	NM_001278243	ZFAND5	939	964	1	1	0.01	0.12	19	12	3UTR
hsa-miR-11400	NM_020917	ZFP14	3659	3677	1	1	-0.01	-0.11	18	17	3UTR
hsa-miR-11400	NM_153018	ZFP3	2530	2555	1	1	-0.26	-0.01	24	9	3UTR
hsa-miR-11400	NM_053023	ZFP91	2907	2933	1	1	0.96	1.53	26	9	3UTR
hsa-miR-11400	NM_001369702	ZFY	4587	4605	1	1	2.40	2.59	18	9	3UTR
hsa-miR-11400	NM_001145276	ZFY	4014	4032	1	1	2.40	2.59	18	9	3UTR
hsa-miR-11400	NM_001284236	ZFYVE16	8160	8179	1	1	-0.07	0.01	19	9	3UTR
hsa-miR-11400	NM_014733	ZFYVE16	8106	8125	1	1	-0.07	0.01	19	9	3UTR
hsa-miR-11400	NM_001105251	ZFYVE16	8234	8253	1	1	-0.07	0.01	19	9	3UTR
hsa-miR-11400	NM_152338	ZG16	1846	1894	1	1	0.08	0.01	48	8	3UTR
hsa-miR-11400	NM_024645	ZMAT4	2142	2161	1	1	5.48	4.51	19	9	3UTR
hsa-miR-11400	NM_001135731	ZMAT4	1914	1933	1	1	5.48	4.51	19	9	3UTR

mirnaid	refseqid	genesymbol	start	end	bindingp	seed	phylopstem	phylopflank	binding_region_length	longest_consecutive_pairings	position
hsa-miR-11400	NM_001289088	ZMYM1	3949	3972	1	1	-0.04	0.10	23	8	3UTR
hsa-miR-11400	NM_001289090	ZMYM1	3689	3712	1	1	0.06	0.10	23	8	3UTR
hsa-miR-11400	XM_011542166	ZMYM1	4021	4044	1	1	0.00	0.00	23	8	3UTR
hsa-miR-11400	NM_024772	ZMYM1	3641	3664	1	1	-0.04	0.10	23	8	3UTR
hsa-miR-11400	NM_001319955	ZMYM1	3471	3494	1	1	0.06	0.10	23	8	3UTR
hsa-miR-11400	NM_001008727	ZNF121	4841	4859	1	1	0.04	0.00	18	14	3UTR
hsa-miR-11400	NM_007147	ZNF175	3833	3851	1	1	0.18	0.04	18	8	3UTR
hsa-miR-11400	NM_007150	ZNF185	3369	3390	1	1	0.00	0.00	21	7	3UTR
hsa-miR-11400	NM_001178106	ZNF185	3465	3486	1	1	0.00	0.00	21	7	3UTR
hsa-miR-11400	NM_001178107	ZNF185	3378	3399	1	1	0.00	0.00	21	7	3UTR
hsa-miR-11400	NM_001178108	ZNF185	3372	3393	1	1	0.00	0.00	21	7	3UTR
hsa-miR-11400	NM_001178110	ZNF185	3192	3213	1	1	0.00	0.00	21	7	3UTR
hsa-miR-11400	NM_001178113	ZNF185	2944	2965	1	1	0.07	-0.07	21	7	3UTR
hsa-miR-11400	NM_001178115	ZNF185	2273	2294	1	1	0.06	-0.13	21	7	3UTR
hsa-miR-11400	NM_001098507	ZNF207	6116	6138	1	1	-0.45	-0.21	22	12	3UTR
hsa-miR-11400	NM_001329974	ZNF208	704	721	0.974359	1	-0.11	-0.13	17	8	3UTR
hsa-miR-11400	NM_001267597	ZNF248	4481	4500	1	1	1.85	1.34	19	11	3UTR

mirnaid	refseqid	genesymbol	start	end	bindingp	seed	phylopstem	phylopflank	binding_region_length	longest_consecutive_pairings	position
hsa-miR-11400	NM_001267605	ZNF248	3096	3115	1	1	-0.07	0.06	19	11	3UTR
hsa-miR-11400	NM_001267606	ZNF248	3297	3319	1	1	-0.07	0.18	22	8	3UTR
hsa-miR-11400	NM_001267606	ZNF248	3845	3864	1	1	-0.07	0.06	19	11	3UTR
hsa-miR-11400	NM_021045	ZNF248	4808	4827	1	1	-0.07	0.06	19	11	3UTR
hsa-miR-11400	NM_001352478	ZNF248	2769	2788	1	1	1.85	1.34	19	11	3UTR
hsa-miR-11400	NM_145011	ZNF25	1654	1677	1	1	-0.23	-0.07	23	9	3UTR
hsa-miR-11400	XM_017023889	ZNF276	2539	2562	1	1	0.00	0.00	23	8	3UTR
hsa-miR-11400	XM_017023889	ZNF276	2376	2398	1	1	0.00	0.00	22	10	3UTR
hsa-miR-11400	NM_001351777	ZNF320	4669	4689	1	1	0.31	0.23	20	7	3UTR
hsa-miR-11400	NM_001351777	ZNF320	678	706	1	1	0.09	-0.32	17	12	3UTR
hsa-miR-11400	NM_001253800	ZNF331	3487	3508	1	1	0.01	-0.04	21	9	3UTR
hsa-miR-11400	NM_001253801	ZNF331	3450	3471	1	1	0.01	-0.04	21	9	3UTR
hsa-miR-11400	NM_001317113	ZNF331	3410	3431	1	1	0.01	-0.04	21	9	3UTR
hsa-miR-11400	NM_001317116	ZNF331	3317	3338	1	1	0.01	-0.04	21	9	3UTR
hsa-miR-11400	NM_001317119	ZNF331	3530	3551	1	1	0.01	-0.04	21	9	3UTR
hsa-miR-11400	NM_001079906	ZNF331	3474	3495	1	1	0.01	-0.04	21	9	3UTR
hsa-miR-11400	NM_001079907	ZNF331	3560	3581	1	1	0.01	-0.04	21	9	3UTR

mirnaid	refseqid	genesymbol	start	end	bindingp	seed	phylopstem	phylopflank	binding_region_length	longest_consecutive_pairings	position
hsa-miR-11400	NM_015655	ZNF337	2593	2611	0.980769	1	-2.98	0.49	18	17	3UTR
hsa-miR-11400	NM_001290261	ZNF337	2989	3007	1	1	1.05	0.26	18	17	3UTR
hsa-miR-11400	NM_001172674	ZNF347	6274	6299	1	1	0.00	0.00	25	8	3UTR
hsa-miR-11400	NM_014951	ZNF365	3332	3349	1	1	0.31	0.52	17	16	3UTR
hsa-miR-11400	NM_021188	ZNF410	1703	1720	1	1	0.58	0.63	17	9	3UTR
hsa-miR-11400	NM_001242924	ZNF410	1872	1889	1	1	0.58	0.63	17	9	3UTR
hsa-miR-11400	NM_001242927	ZNF410	1484	1501	1	1	0.58	0.63	17	9	3UTR
hsa-miR-11400	NM_001242928	ZNF410	1796	1813	1	1	0.58	0.63	17	9	3UTR
hsa-miR-11400	NM_001146175	ZNF414	1350	1373	1	1	0.68	1.44	23	7	3UTR
hsa-miR-11400	NM_001300883	ZNF426	6602	6624	1	1	0.89	0.07	22	13	3UTR
hsa-miR-11400	NM_024106	ZNF426	6465	6487	1	1	-0.59	-0.46	22	13	3UTR
hsa-miR-11400	NM_014650	ZNF432	2345	2365	1	1	0.29	-0.07	20	7	3UTR
hsa-miR-11400	NM_001322285	ZNF432	2222	2242	1	1	-0.02	0.11	20	7	3UTR
hsa-miR-11400	NM_030634	ZNF436	1866	1885	1	1	0.73	0.83	19	8	3UTR
hsa-miR-11400	NM_152355	ZNF441	3908	3947	1	1	0.31	0.08	39	10	3UTR
hsa-miR-11400	NM_001297623	ZNF461	2912	2943	1	1	0.94	0.21	22	10	3UTR
hsa-miR-11400	NM_153257	ZNF461	2981	3012	1	1	0.00	-0.10	22	10	3UTR

mirnaid	refseqid	genesymbol	start	end	bindingp	seed	phylopstem	phylopflank	binding_region_length	longest_consecutive_pairings	position
hsa-miR-11400	NM_001308424	ZNF473	2906	2931	1	1	-0.48	-0.31	25	8	3UTR
hsa-miR-11400	NM_015428	ZNF473	3106	3131	1	1	-0.48	-0.31	25	8	3UTR
hsa-miR-11400	NM_001006656	ZNF473	2984	3009	1	1	-0.48	-0.31	25	8	3UTR
hsa-miR-11400	NM_001007169	ZNF483	1517	1536	1	1	-0.29	-0.09	19	10	3UTR
hsa-miR-11400	NM_001355444	ZNF487	1326	1349	1	1	1.06	-0.05	23	8	3UTR
hsa-miR-11400	NM_001355445	ZNF487	1262	1285	1	1	1.06	-0.05	23	8	3UTR
hsa-miR-11400	NM_153034	ZNF488	1442	1461	0.953846	1	-0.46	-0.27	19	13	3UTR
hsa-miR-11400	NM_001314059	ZNF510	4215	4232	1	1	-0.40	-0.17	17	12	3UTR
hsa-miR-11400	NM_001314059	ZNF510	2778	2796	1	1	0.12	0.05	18	12	3UTR
hsa-miR-11400	NM_014930	ZNF510	3763	3780	1	1	-0.43	-0.23	17	12	3UTR
hsa-miR-11400	NM_001297763	ZNF546	5638	5669	1	1	-0.26	0.10	19	14	3UTR
hsa-miR-11400	NM_178544	ZNF546	5716	5747	1	1	-0.26	0.10	19	14	3UTR
hsa-miR-11400	NM_001277090	ZNF550	1967	1985	1	1	0.02	-0.59	18	15	3UTR
hsa-miR-11400	NM_001277091	ZNF550	1967	1985	1	1	0.12	-0.70	18	15	3UTR
hsa-miR-11400	NM_024762	ZNF552	1723	1748	1	1	-1.83	-0.57	25	8	3UTR
hsa-miR-11400	NM_152791	ZNF555	6827	6847	1	1	-0.14	-0.62	20	7	3UTR
hsa-miR-11400	NM_001172775	ZNF555	6824	6844	1	1	-0.14	-0.62	20	7	3UTR

mirnaid	refseqid	genesymbol	start	end	bindingp	seed	phylopstem	phylopflank	binding_region_length	longest_consecutive_pairings	position
hsa-miR-11400	NM_032838	ZNF566	2878	2894	1	1	0.13	0.40	16	12	3UTR
hsa-miR-11400	XM_006723447	ZNF566	3287	3303	1	1	0.00	0.00	16	12	3UTR
hsa-miR-11400	XM_011527428	ZNF566	3049	3065	1	1	0.00	0.00	16	12	3UTR
hsa-miR-11400	NM_001145345	ZNF566	2943	2959	1	1	0.25	0.22	16	12	3UTR
hsa-miR-11400	NM_001159860	ZNF583	2516	2538	1	1	0.00	0.00	17	9	3UTR
hsa-miR-11400	NM_001288800	ZNF585A	5060	5082	1	1	-0.28	0.00	22	8	3UTR
hsa-miR-11400	NM_152279	ZNF585B	5693	5709	1	1	-0.44	0.17	16	10	3UTR
hsa-miR-11400	NM_001204814	ZNF586	2037	2058	1	1	3.65	1.97	21	12	3UTR
hsa-miR-11400	NM_178167	ZNF598	3070	3088	1	1	2.08	2.53	18	15	3UTR
hsa-miR-11400	NM_015042	ZNF609	5712	5733	1	1	0.24	0.77	21	10	3UTR
hsa-miR-11400	NM_001287245	ZNF621	966	991	1	1	0.11	-0.06	25	8	3UTR
hsa-miR-11400	NM_198484	ZNF621	5076	5110	1	1	-0.02	0.05	34	10	3UTR
hsa-miR-11400	NM_198484	ZNF621	7045	7070	1	1	0.18	0.02	25	8	3UTR
hsa-miR-11400	NM_198484	ZNF621	2320	2339	1	1	0.46	0.07	19	11	3UTR
hsa-miR-11400	NM_001098414	ZNF621	4840	4874	1	1	-0.02	0.05	34	10	3UTR
hsa-miR-11400	NM_001098414	ZNF621	6809	6834	1	1	0.18	0.02	25	8	3UTR
hsa-miR-11400	NM_001098414	ZNF621	2084	2103	1	1	0.46	0.07	19	11	3UTR

mirnaid	refseqid	genesymbol	start	end	bindingp	seed	phylopstem	phylopflank	binding_region_length	longest_consecutive_pairings	position
hsa-miR-11400	NM_001080417	ZNF629	4706	4725	1	1	0.15	1.35	19	13	3UTR
hsa-miR-11400	NM_138494	ZNF655	3277	3328	1	1	0.97	0.29	26	9	3UTR
hsa-miR-11400	NM_001009960	ZNF655	3324	3375	1	1	0.97	0.29	26	9	3UTR
hsa-miR-11400	NM_001083956	ZNF655	3382	3433	1	1	0.55	0.25	26	9	3UTR
hsa-miR-11400	NM_001085368	ZNF655	3429	3480	1	1	0.97	0.29	26	9	3UTR
hsa-miR-11400	NM_001355197	ZNF66	4230	4260	1	1	0.14	-0.08	30	11	3UTR
hsa-miR-11400	NM_138447	ZNF689	2991	3012	1	1	0.70	1.06	21	8	3UTR
hsa-miR-11400	XM_011527092	ZNF701	4951	4972	1	1	0.00	0.00	21	8	3UTR
hsa-miR-11400	XM_011527092	ZNF701	4200	4236	1	1	0.00	0.00	36	8	3UTR
hsa-miR-11400	XM_011527092	ZNF701	3066	3100	1	1	0.00	0.00	34	8	3UTR
hsa-miR-11400	NM_018260	ZNF701	4863	4884	1	1	-0.16	0.17	21	8	3UTR
hsa-miR-11400	NM_001172655	ZNF701	5171	5192	1	1	-0.16	0.17	21	8	3UTR
hsa-miR-11400	NM_001033723	ZNF704	11553	11599	1	1	0.14	0.04	21	14	3UTR
hsa-miR-11400	NM_001164457	ZNF705G	2848	2876	1	1	1.43	-0.11	23	9	3UTR
hsa-miR-11400	NM_001159279	ZNF716	4890	4906	1	1	-0.32	-0.06	16	8	3UTR
hsa-miR-11400	NM_001159522	ZNF727	4361	4379	1	1	-0.14	0.03	18	14	3UTR
hsa-miR-11400	NM_001159293	ZNF737	4024	4045	1	1	0.36	0.29	21	14	3UTR

mirnaid	refseqid	genesymbol	start	end	bindingp	seed	phylopstem	phylopflank	binding_region_length	longest_consecutive_pairings	position
hsa-miR-11400	NM_001004304	ZNF740	6746	6783	1	1	1.24	0.90	20	18	3UTR
hsa-miR-11400	NM_001004304	ZNF740	6764	6783	1	1	3.24	0.84	19	18	3UTR
hsa-miR-11400	NM_007131	ZNF75D	5256	5275	1	1	-0.60	-0.63	19	13	3UTR
hsa-miR-11400	NM_001185063	ZNF75D	2629	2648	1	1	0.00	0.00	19	13	3UTR
hsa-miR-11400	NM_001304335	ZNF773	4914	4969	1	1	0.11	0.03	18	12	3UTR
hsa-miR-11400	NM_001304337	ZNF773	4869	4924	1	1	0.11	0.03	18	12	3UTR
hsa-miR-11400	XM_017023015	ZNF778	2681	2697	0.953846	1	0.00	0.00	16	10	3UTR
hsa-miR-11400	NM_001142579	ZNF780A	1361	1387	1	1	0.00	0.00	26	11	3UTR
hsa-miR-11400	NM_001001662	ZNF782	3643	3678	1	1	1.19	1.29	35	12	3UTR
hsa-miR-11400	NM_001195220	ZNF783	2056	2074	1	1	-0.02	-0.47	18	17	3UTR
hsa-miR-11400	NM_153358	ZNF791	6097	6114	1	1	-0.46	0.05	17	9	3UTR
hsa-miR-11400	NM_001355461	ZNF806	560	596	1	1	-0.01	0.01	36	8	3UTR
hsa-miR-11400	XM_011543900	ZNF81	2539	2561	0.961538	1	0.00	0.00	22	9	3UTR
hsa-miR-11400	NM_007137	ZNF81	2420	2442	0.961538	1	-0.12	0.14	22	9	3UTR
hsa-miR-11400	NM_007137	ZNF81	9437	9470	0.980769	1	0.49	0.22	20	13	3UTR
hsa-miR-11400	NM_001037232	ZNF829	3735	3758	1	1	0.84	0.73	23	9	3UTR
hsa-miR-11400	NM_001171979	ZNF829	3698	3721	1	1	1.06	-0.34	23	9	3UTR

mirnaid	refseqid	genesymbol	start	end	bindingp	seed	phylopstem	phylopflank	binding_region_length	longest_consecutive_pairings	position
hsa-miR-11400	NM_021035	ZNFX1	6702	6720	1	1	1.89	1.87	18	9	3UTR
hsa-miR-11400	NM_017953	ZNHIT6	4089	4110	1	1	-0.29	-0.10	21	8	3UTR
hsa-miR-11400	NM_001170670	ZNHIT6	3972	3993	1	1	0.03	0.12	21	8	3UTR
hsa-miR-11400	NM_147128	ZNRF2	1580	1600	1	1	1.07	1.60	20	8	3UTR
hsa-miR-11400	NM_147128	ZNRF2	1627	1672	1	1	1.65	0.92	45	11	3UTR
hsa-miR-11400	NM_001286568	ZRANB3	4231	4280	0.974359	1	3.31	2.99	26	8	3UTR
hsa-miR-11400	NM_001286569	ZRANB3	5829	5860	1	1	3.44	3.10	31	8	3UTR
hsa-miR-11400	XM_017011528	ZSCAN12	2017	2054	1	1	0.00	0.00	19	7	3UTR
hsa-miR-11400	NM_001287821	ZWILCH	2097	2122	1	1	3.52	2.91	25	10	3UTR
hsa-miR-11400	NM_001287822	ZWILCH	2001	2026	1	1	2.17	1.35	25	10	3UTR
hsa-miR-11400	NM_001287823	ZWILCH	1950	1975	1	1	3.52	2.91	25	10	3UTR
hsa-miR-11400	NM_017975	ZWILCH	2002	2027	1	1	3.52	2.91	25	10	3UTR

Supplementary Table S8. KEGG pathway enrichment analysis for miR-11400 predicted target genes.

nGenes[a]	Enrichment FDR [b]	Pathway Genes	Fold Enrichment [c]	Pathway	Genes
33	0.0025	157	2.246374085	Hippo signaling pathway	RASSF1 CSNK1D CTNNA1 AMOT DLG2 DLG3 DVL3 BBC3 CRB2 BIRC3 SMAD2 SMAD3 SERPINE1 WNT4 PPP2R2B MOB1A CCND1 SAV1 MPP5 BMPR1A BMPR2 TGFB1 ACTG1 TP73 WNT3 WNT5A WNT2B WNT9B YWHAG FZD3 CCND2 BTRC MOB1B
25	0.0213	128	2.087362283	Lysosome	AP3S2 CTSC AP1S3 HGSNAT CTSK CTSS DNASE2 GGA3 GALC GBA AP3M1 HYAL1 LIPA M6PR NEU1 ACP2 ACP5 LAPTM4B SORT1 PSAPL1 GNPTAB CD164 SCARB2 LITAF ENTPD4
20	0.0213	93	2.298342987	TGF-beta signaling pathway	CDKN2B DCN FMOD GREM1 AMHR2 INHBB SMAD2 SMAD3 SMAD5 NEO1 MAPK1 RGMA TGIF2 SKP1 BMPR1A BMPR2 SP1 TGFB1 TNF ZFYVE16
33	0.0213	181	1.948512328	Axon guidance	SEMA4F EFNA5 EPHA3 EPHA4 EPHB2 NGEF KRAS NEO1 NFATC3 PAK1 PAK2 PAK3 PDPK1 WNT4 SSH1 ENAH MAPK1 DPYSL5 SEMA3G RGMA RASA1 ROBO2 CXCL12 SLIT3 BMPR2 SRC TRPC5 WNT5A FZD3 NTNG2 SEMA5A PLXNA4B LRIG2
12	0.0213	41	3.127988748	Bladder cancer	RASSF1 DAPK3 E2F3 EGF DAPK2 KRAS MDM2 MAPK1 CCND1 SRC VEGFA RPS6KA5
10	0.0258	33	3.238574209	SNARE interactions in vesicular transport	VTI1B YKT6 STX1B STX1A STX5 VAMP4 STX16 STX11 SNAP29 GOSR1
19	0.0258	89	2.281557336	GABAergic synapse	ADCY1 ABAT GABBR1 GABRA6 GABRB2 GABRB3 GABRG1 GABRG3 PIG59 GNG4 GNG11 GNGT1 KCNJ6 GABRQ TRAK2 SRC CACNA1S SLC38A1 HAP1
34	0.0258	202	1.798851615	Proteoglycans in cancer	HPSE DCN ELK1 ERBB4 AKT2 ESR1 FGFR1 MRAS ANK3 IL12B ITPR2 KRAS SMAD2 MDM2 PAK1 PDPK1 PLAUR WNT4 MAPK1 MAPK13 CCND1 RDX SRC TGFB1 TLR4 ACTG1 TNF VEGFA EZR WNT3 WNT5A WNT2B WNT9B FZD3

[a] number of genes enriched in the pathway

[b] FDR is adjusted from the hypergeometric test. Fold Enrichment indicates how drastically genes of a certain pathway is overrepresented.

[c] Fold Enrichment is defined as the percentage of genes in the list belonging to a pathway, divided by the corresponding percentage in the background

Supplementary Table S9. Characteristics of the retrospective study used in the validation analysis.

	**MPM cases***	**Controls****
**N (%)**	30 (60)	20 (40)
**GENDER**		
M (%)	22 (73)	20 (100)
F (%)	8 (27)	0 (0)
**AGE** (mean±sd)	72± 10	65±4

*MPM enrolled at Azienda Ospedaliero-Universitaria Maggiore della Carità (Novara) ; ** Cancer-free individuals enrolled at the Occupational Medicine Department of the University of Genoa and at the Oncology Department of the Villa Scassi Hospital, Genova, Italy

References

1. Pesch B, Gawrych K, Rabstein S, Weiss T, Casjens S, Rihs HP, et al. N-acetyltransferase 2 phenotype, occupation, and bladder cancer risk: results from the EPIC cohort. Cancer Epidemiol Biomarkers Prev. 2013 Nov;22(11):2055-65.
2. Livak KJ, Schmittgen TD. Analysis of relative gene expression data using real-time quantitative PCR and the 2(-Delta Delta C(T)) Method. Methods. 2001 Dec;25(4):402-8.
